# DNA barcoding of Afrotropical nose flies (Diptera, Calliphoridae, Rhiniinae): species identification, female-male morphotype association, and reference library development

**DOI:** 10.3897/zookeys.1284.189450

**Published:** 2026-07-03

**Authors:** Arianna Thomas-Cabianca, Kurt Jordaens, Eliana Buenaventura, Arn Rytter-Jensen, Anabel Martínez-Sánchez, Georg Goergen, Santos Rojo, Pierfilippo Cerretti, Thomas Pape

**Affiliations:** 1 Senckenberg Natural History Collections Dresden, Dresden, Germany Royal Museum for Central Africa, Department of Biology and JEMU Tervuren Belgium; 2 Department of Environmental Sciences & Natural Resources, University of Alicante, San Vicente del Raspeig, Alicante, Spain Senckenberg Natural History Collections Dresden Dresden Germany; 3 Royal Museum for Central Africa, Department of Biology and JEMU, Tervuren, Belgium Department of Biology and Biotechnologies “Charles Darwin”, Sapienza University of Rome Rome Italy; 4 Rigshospitalet, Copenhagen University Hospital, Copenhagen, Denmark Natural History Museum of Denmark, University of Copenhagen Copenhagen Denmark; 5 Natural History Museum of Denmark, University of Copenhagen, Copenhagen, Denmark International Institute of Tropical Agriculture, Biodiversity Centre Cotonou Benin; 6 Department of Biology and Biotechnologies “Charles Darwin”, Sapienza University of Rome, Rome, Italy Rigshospitalet, Copenhagen University Hospital Copenhagen Denmark; 7 International Institute of Tropical Agriculture, Biodiversity Centre, Cotonou, Benin Department of Environmental Sciences & Natural Resources, University of Alicante Alicante Spain

**Keywords:** COI reference library, Cytochrome c oxidase I, mitochondrial gene, Rhiniines, sex linkage

## Abstract

DNA barcoding offers a practical approach to species identification, especially in morphologically complex taxa such as Rhiniinae (Diptera, Calliphoridae). The most extensive COI barcode dataset for the subfamily to date was assembled, combining 216 newly generated sequences with more than 1,150 publicly available records from BOLD Systems and GenBank, with emphasis on Afrotropical taxa. Distance-based analyses, Neighbour-Joining (NJ) and Maximum Likelihood (ML) clustering, were evaluated, as well as automated barcode clustering approaches (ABGD and ASAP), for identifying morphospecies within this group. These analyses recognized 79 putative Afrotropical species, with most morphospecies forming well-supported barcode clusters congruent with morphological identifications. Several lineages, however, including species of *Fainia*, *Rhinia*, *Cosmina*, *Stomorhina*, and *Rhyncomya*, displayed low interspecific COI divergence, indicating that COI alone offers limited resolution. Yet, NJ and ML clustering helped highlight problematic taxa and identify previously unrecognized lineages. Public data on BOLD Systems comprised 957 BINs identified to subfamily level. Integrating ML, ABGD, ASAP, and voucher-based assessment allowed the assignment of 749 unnamed BINs to 11 species names, 193 BINs to eight genus names within 24 morphospecies, 12 BINs remain as Rhiniinae, and three represent non-Rhiniinae. The analyses also allowed linking 31 Afrotropical female morphotypes to their conspecific male morphotypes. This study establishes the first quality-controlled COI reference library for Afrotropical Rhiniinae and provides a framework for future taxonomic, ecological, and molecular investigations.

## Introduction

Rhiniinae (nose flies) were recently reinstated as a subfamily of Calliphoridae (Kutty et al. 2010; Lehrer 2011; Pape et al. 2011; Singh and Wells 2013; Marinho et al. 2017; Cerretti et al. 2017, 2019; Kutty et al. 2019; Yan et al. 2021; Beza‐Beza et al. 2026) and are divided into a monophyletic tribe, Rhiniini, and a paraphyletic tribe, Cosminini (Malloch 1926; Kurahashi and Kirk-Spriggs 2006; Buenaventura et al. 2020). Rhiniinae are distributed in the Australasian, Afrotropical, Oriental, and Palaearctic regions (with one species introduced to Bermuda), with nearly 400 described species across 30 genera *sensu*Pape et al. (2011). The Afrotropical region contains the largest diversity with approximately 150 described species in 16 genera (Pont 1980; Kurahashi and Kirk-Spriggs 2006; Lehrer 2011; Rognes 2013; Rognes et al. in press).

The biology and ecology of most Rhiniinae remain poorly known. Several species have been described as termitophilous, yet the nature of their association with termites remains unclear (Ferrar 1987; Arce et al. 2019). Recently, Schär et al. (2025) reported *Rhyncomya* Robineau-Desvoidy, 1830 larvae inhabiting the nests of the harvester termite *Anacanthotermes ochraceus* (Burmeister, 1839) (Blattodea, Hodotermitidae) in southern Morocco. These larvae exhibit morphological and chemical adaptations that enable them to integrate into termite colonies undetected, providing the first clear evidence of highly specialized termitophily in Rhiniinae and highlighting a remarkable case of evolutionary adaptation to a concealed ecological niche. Observations of females ovipositing in soils rich in organic matter (Cuthbertson 1933; Peris 1952b; Thomas-Cabianca et al. 2023) suggest that nose flies also exploit other concealed habitats. This association with concealed habitats may explain why Rhiniinae immature stages have remained elusive for so long. To date, *Stomorhina lunata* (Fabricius, 1805) is the only Rhiniinae species for which larval predation has been documented, with larvae reported to prey and develop on grasshopper oothecae (Greathead 1962). In addition, an ectoparasitic association has been reported between species of *Stomorhina* Rondani, 1861 and Himalayan ants of the genus *Myrmica* Latreille, 1804, providing further evidence that some Rhiniinae may exploit social insects or their nests during larval development (Bharti and Bharti 2016).

Current taxonomic knowledge of Afrotropical Rhiniinae relies largely on older literature (Peris 1951, 1952a, 1952b, 1956, 1960; Zumpt 1957, 1958, 1962a, 1962b, 1967; Zumpt and Stimie 1965; Zumpt and Argo 1978), which, while foundational, lacks the detail and accuracy expected by modern standards. The most recent extensive work was conducted by A.Z. Lehrer, who described 43 species, seven genera, and two subfamilies of Afrotropical nose flies between 1970 and 2012 (Verves and Khrokalo 2020). However, his work is widely considered to require careful revision (e.g., Rognes 2005, 2011). Overall, of the 16 Afrotropical genera of Rhiniinae, only three have been revised recently: *Thoracites* Brauer & Bergenstamm, 1891 (Kurahashi 2001), *Pseudorhyncomyia* Peris, 1952 (Rognes 2013), and *Fainia* Zumpt, 1958 (Thomas-Cabianca et al. 2021); except for Schär et al. (2025) and Li et al. (2026), no molecular studies have been conducted within Rhiniinae, and the relationships among its genera and species remain unresolved. Morphological identification of nose flies remains challenging and relies strongly on the morphology of male terminalia. Females are even harder to identify and often cannot be linked to males with certainty. This is particularly evident in the two most diverse genera, *Rhyncomya* and *Isomyia* Walker, 1859, which are arranged into species groups based on morphology and in which most females remain unidentified to species level (Peris 1952b; Zumpt 1958; Kurahashi and Kirk-Spriggs 2006). Therefore, the identification and taxonomy of nose flies would greatly benefit from integrating molecular data with morphological data, especially for morphologically intricate or insufficiently studied taxa (Hebert et al. 2003a; Jordaens et al. 2013a; Zhang et al. 2016).

DNA barcoding is widely used for species identification and is notably effective in groups where morphological identification is challenging. Several studies have successfully used DNA barcoding to distinguish species within Calliphoridae, and related families such as Fanniidae, Mesembrinellidae, Polleniidae and Sarcophagidae (Jordaens et al. 2013a, 2013b; Grzywacz et al. 2017; Yusseff-Vanegas and Agnarsson 2017; Buenaventura et al. 2018; Whitworth and Yusseff-Vanegas 2019; García-Ruilova et al. 2020; Moreno et al. 2020; Szpila et al. 2023, 2025; Parmar et al. 2026). DNA barcoding has also proven valuable for linking life stages, associating sexes, and discovering cryptic diversity (Hebert et al. 2003a, 2003b, 2004; Grzywacz et al. 2021; Krčmar et al. 2022; Posada-López et al. 2023; Pramual et al. 2023). Although many cytochrome *c* oxidase subunit I (COI) DNA barcode sequences for Rhiniinae are available in BOLD and GenBank repositories, most lack species- or genus-level identifications, hampering testing the accuracy of DNA barcoding as an identification tool in this subfamily.

In this study, we used COI DNA barcode sequences of Afrotropical Rhiniinae to: (1) evaluate the use of DNA barcoding to test morphology-based Rhiniinae species identifications, (2) link female morphotypes with their conspecific male morphotypes, (3) identify unnamed DNA barcode sequences of Rhiniinae in BOLD Systems and GenBank, and (4) generate a curated DNA barcode library for Afrotropical Rhiniinae.

## Materials and methods

### Samples and morphological identifications

A total of 216 adult specimens of Afrotropical Rhiniinae specimens were studied (Table 1), either dry-pinned or preserved in 70–96% ethanol. We used the taxonomical identification keys in Kurahashi (2001), Kurahashi and Kirk-Spriggs (2006), Lehrer (2007), Peris (1952b, 1992), Rognes (1991, 1998, 2013), Thomas-Cabianca et al. (2021), Zumpt (1956, 1958, 1962a, 1962b, 1972a, 1972b, 1974, 1981), and Zumpt and Stimie (1965) to identify the specimens.

**Table 1.** Details of the Afrotropical species and specimens of Rhiniinae studied, including their molecular identification. **Species**: **sp** = corresponding morphospecies name; **cf** = conspecific species name proposed (to be confirmed); **Sex**: **F** = female; **M** = male; **Consr**: specimen preservation method (**E** = ethanol, **P** = pinned); **Year**: year when the specimen was collected; **Tissue**: targeted tissue for the DNA extraction (**Ab** = abdomen, **Le** = leg/s, **Th** = thorax, **WB** = whole body); **Country**: country of collection; **Species code**: specimen identifiers (in all the analyses); **Museum ID**: acronyms of the specimens repositories, and specimen catalog numbers when available; **Code**: researcher code that indicates which researcher was responsible for the DNA extraction of the specimen; **AC**: accession number of the sequence in GenBank www.ncbi.nlm.nih.gov (**ATC** = Arianna Thomas-Cabianca, **EB** = Eliana Buenaventura and **KJ** = Kurt Jordaens).

**Species**	**Sex**	**Consr**	**Year**	**Tissue**	**Country**	**Species code**	**Museum ID**	C**ode**	**GenBank AC**
***Albaredaya malgache***	F	E	2017	Ab	Madagascar	A3_Albaredaya_malgache_F	ZMUC	ATC	PZ426670
***Albaredaya malgache***	F	E	2017	Ab	Madagascar	K5_Albaredaya_malgache_F	ZMUC	ATC	PZ426713
***Cosmina aenea***	M	P	2012	Ab	Namibia	C3_Cosmina_aenea_M	BMSA (D) 47655	ATC	PZ426680
***Cosmina aenea***	F	P	2012	Ab	Namibia	C4_Cosmina_aenea_F	BMSA (D) 47684	ATC	PZ426681
***Cosmina fuscipennis***	F	E	2017	WB	South Africa	USA01_Cosmina_fuscipennis_F	RMCA 006	EB	PZ426748
***Cosmina fuscipennis***	M	E	2017	Le	South Africa	USA02_Cosmina_fuscipennis_M	RMCA 005	EB	PZ426749
***Cosmina gracilis***	M	E	2018	Ab	Namibia	A8_Cosmina_gracilis_M	ZMUC	ATC	PZ426672
***Cosmina margaritae***	F	E	2015	Ab	Tanzania	A5_Cosmina_margaritae_F	ZMUC	ATC	PZ426671
***Cosmina* sp. 1**	F	E	-	Le	South Africa	C20_Cosmina_sp1_cf_fuscipennis_F	ZMUC	ATC	PZ426679
***Cosmina* sp. 2**	M	P	2007	Ab	Madagascar	C11_Cosmina_sp2_M	AMSA 106	ATC	PZ426676
***Cosmina* sp. 3**	F	P	2007	Ab	Madagascar	C10_Cosmina_sp3_F	AMSA 105	ATC	PZ426675
***Cosmina* sp. 5**	F	P	2006	Ab	Kenya	C18_Cosmina_sp5_F	MZSUR 07	ATC	PZ426677
***Cosmina* sp. 6**	M	P	2011	Ab	Kenya	C19_Cosmina_sp6_M	BMSA (D) 32932	ATC	PZ426678
***Cosmina* sp. 9**	M	P	2007	Ab	Madagascar	C8_Cosmina_sp9_M	AMSA 100	ATC	PZ426682
***Eurhyncomyia diversicolor***	M	E	2006	Ab	Mozambique	E1_Eurhyncomyia_diversicolor_M	ZMUC	ATC	PZ426683
***Eurhyncomyia diversicolor***	F	E	2006	Ab	Mozambique	E2_Eurhyncomyia_diversicolor_F	ZMUC	ATC	PZ426684
***Fainia albitarsis***	M	P	2013	Ab	Tanzania	F20_Fainia_albitarsis_M	ZMUC	ATC	PZ426686
***Fainia albitarsis***	F	E	2014	Ab	Tanzania	K3_Fainia_albitarsis_F	ZMUC	ATC	PZ426711
***Fainia albitarsis***	F	P	2013	Ab	Tanzania	K7_Fainia_albitarsis_F	ZMUC	ATC	PZ426714
***Fainia albitarsis***	M	E	2017	WB	Kenya	USA03_Fainia_albitarsis_M	CEUA 011	EB	PZ426750
***Fainia albitarsis***	F	E	2017	Le	Kenya	USA04_Fainia_albitarsis_F	CEUA 003	EB	PZ426751
***Fainia elongata***	F	P	2010	Ab	DR Congo	F2_Fainia_elongata_F	BMSA (D) (BECE 03372)	ATC	PZ426685
***Fainia elongata***	M	P	2010	Ab	Malawi	F5_Fainia_elongata_M	BMSA (D) (BECE 03371)	ATC	PZ426688
***Fainia inexpectata***	F	P	2016	Ab	Malawi	F3_Fainia_inexpectata_F	BMSA (D) 92318	ATC	PZ426687
***Fainia inexpectata***	M	P	2006	Th	Kenya	F6_Fainia_inexpectata_M	MZSUR 01	ATC	PZ426689
***Isomyia cuthbertsoni***	M	P	2009	Ab	South Africa	I4_Isomyia_cuthbertsoni_M	BMSA (D) 17166	ATC	PZ426706
***Isomyia darwini***	F	P	2009	Ab	South Africa	I11_Isomyia_darwini_F	BMSA (D) 15543	ATC	PZ426697
***Isomyia distinguenda***	F	P	2016	Ab	Malawi	I17_Isomyia_distinguenda_F	BMSA (D) 92320	ATC	PZ426701
***Isomyia distinguenda***	F	P	2016	Ab	Malawi	I19_Isomyia_distinguenda_F	BMSA (D) 92327	ATC	PZ426703
***Isomyia distinguenda***	M	P	2009	Ab	South Africa	I8_Isomyia_distinguenda_M	BMSA (D) 13696	ATC	PZ426709
***Isomyia dubiosa***	F	P	2016	Ab	Togo	I1_Isomyia_dubiosa_F	BMSA (D) 84374	ATC	PZ426695
***Isomyia dubiosa***	F	P	2016	Ab	Togo	I12_Isomyia_dubiosa_F	BMSA (D) 84368	ATC	PZ426698
***Isomyia dubiosa***	F	P	2016	Ab	Togo	I2_Isomyia_dubiosa_F	BMSA (D) 84375	ATC	PZ426704
***Isomyia eos***	F	P	2009	Ab	South Africa	I10_Isomyia_eos_F	BMSA (D) 13717	ATC	PZ426696
***Isomyia faini***	M	P	2016	Ab	Togo	I18_Isomyia_faini_M	BMSA (D) 84370	ATC	PZ426702
***Isomyia natalensis***	F	E	2019	Le	South Africa	1306B05_Isomyia_natalensis_F	1306B05 RMCA	KJ	PZ426645
***Isomyia natalensis***	F	E	2019	Le	South Africa	1306C05_Isomyia_natalensis_F	1306C05 RMCA	KJ	PZ426651
***Isomyia natalensis***	F	E	2019	Le	South Africa	1306D04_Isomyia_natalensis_F	1306D04 RMCA	KJ	PZ426655
***Isomyia natalensis***	F	E	2019	Le	South Africa	1306F01_Isomyia_natalensis_F	1306F01 RMCA	KJ	PZ426661
***Isomyia natalensis***	F	P	2015	Le	South Africa	USA05_Isomyia_nataliensis_F	CEUA 031	EB	PZ426752
***Isomyia natalensis***	M	P	2015	WB	South Africa	USA06_Isomyia_nataliensis_M	CEUA 004	EB	PZ426753
***Isomyia natalensis***	M	P		Le	South Africa	USA08_Isomyia_natalensis_F	NMSA-DIP 80117	EB	PZ426755
***Isomyia pubera***	M	E	2014	Le	South Africa	I5_Isomyia_pubera_M	ZMUC	ATC	PZ426707
***Isomyia pubera***	F	E	2019	Le	South Africa	1306A06_Isomyia_pubera_F	1306A06 RMCA	KJ	PZ426639
***Isomyia pubera***	F	P	2008	Le	South Africa	USA07_Isomyia_pubera_F	NMSA-DIP 80123	EB	PZ426754
***Isomyia* sp. 1**	F	P	2008	Ab	Uganda	I14_Isomyia_sp1_F	SAMC DIP A015260	ATC	PZ426699
***Isomyia* sp. 2**	M	P	2013	Ab	Cameroon	I15_Isomyia_sp2_M	BMSA (D) 50778	ATC	PZ426700
***Isomyia* sp. 3**	F	P	2010	Ab	South Africa	I6_Isomyia_sp3_cf_cuthbertsoni_F	BMSA (D) 31132	ATC	PZ426708
***Isomyia* sp. 3**	F	P	2009	Ab	South Africa	I9_Isomyia_sp3_cf_cuthbertsoni_F	BMSA (D) 16928	ATC	PZ426710
***Isomyia tristis***	M	E	2019	Le	South Africa	1282A03_Isomyia_tristis_M	1282A03 RMCA	KJ	PZ426619
***Isomyia tristis***	F	E	2019	Le	South Africa	1282A04_Isomyia_tristis_F	1282A04 RMCA	KJ	PZ426620
***Isomyia tristis***	F	E	2019	Le	South Africa	1282B01_Isomyia_tristis_F	1282B01 RMCA	KJ	PZ426623
***Isomyia tristis***	F	E	2019	Le	South Africa	1282B04_Isomyia_tristis_F	1282B04 RMCA	KJ	PZ426624
***Isomyia tristis***	F	E	2019	Le	South Africa	1282B05_Isomyia_trisits_F	1282B05 RMCA	KJ	PZ426625
***Isomyia tristis***	M	E	2019	Le	South Africa	1306A07_Isomyia_tristis_F	1306A07 RMCA	KJ	PZ426640
***Isomyia tristis***	F	E	2019	Le	South Africa	1306B03_Isomyia_trisits_F	1306B03 RMCA	KJ	PZ426643
***Isomyia tristis***	F	E	2019	Le	South Africa	1306B04_Isomyia_tristis_F	1306B04 RMCA	KJ	PZ426644
***Isomyia tristis***	F	E	2019	Le	South Africa	1306C04_Isomyia_tristis_F	1306C04 RMCA	KJ	PZ426650
***Isomyia tristis***	M	E	2019	Le	South Africa	1306C06_Isomyia_tristis_M	1306C06 RMCA	KJ	PZ426652
***Isomyia tristis***	M	E	2019	Le	South Africa	1306C08_Isomyia_tristis_M	1306C08 RMCA	KJ	PZ426653
***Isomyia tristis***	F	E	2019	Le	South Africa	1306F03_Isomyia_tristis_F	1306F03 RMCA	KJ	PZ426662
***Isomyia tristis***	F	E	2019	Le	South Africa	1306F04_Isomyia_tristis_F	1306F04 RMCA	KJ	PZ426663
***Isomyia tristis***	M	E	2014	Le	South Africa	I20B_Isomyia_tristis_M	ZMUC	ATC	PZ426705
***Isomyia tristis***	M	P	2015	Le	South Africa	USA09_Isomyia_tristis_M	CEUA 010	EB	PZ426756
***Isomyia tristis***	F	P	2015	Le	South Africa	USA10_Isomyia_tristis_F	CEUA 011	EB	PZ426757
***Rhinia apicalis***	M	E	2019	Le	Ghana	1279A02_Rhinia_apicalis_M	1279A02 RMCA	KJ	PZ426584
***Rhinia apicalis***	M	E	2019	Le	Togo	1279A03_Rhinia_apicalis_M	1279A03 RMCA	KJ	PZ426585
***Rhinia apicalis***	M	E	2019	Le	Togo	1279C04_Rhinia_apicalis_M	1279C04 RMCA	KJ	PZ426598
***Rhinia apicalis***	M	E	2019	Le	Togo	1279C07_Rhinia_apicalis_M	1279C07 RMCA	KJ	PZ426601
***Rhinia apicalis***	M	E	2019	Le	Ghana	1279E03_Rhinia_apicalis_M	1279E03 RMCA	KJ	PZ426608
***Rhinia apicalis***	F	P	2012	Ab	Namibia	R12_Rhinia_apicalis_F	BMSA (D) 33375	ATC	PZ426715
***Rhinia apicalis***	M	P	2016	Ab	Togo	R18_Rhinia_apicalis_M	BMSA (D) 84399	ATC	PZ426716
***Rhinia apicalis***	F	P	2012	Ab	Namibia	R24_Rhinia_apicalis_F	ZMUC	ATC	PZ426718
***Rhinia apicalis***	F	P	2017	Le	Kenya	USA11_Rhinia_apicalis_F	CEUA 001	EB	PZ426758
**Rhinia cf. apicalis**	F	E	2019	Le	Togo	1279A04_Rhinia_cf_apicalis_F	1279A04 RMCA	KJ	PZ426586
**Rhinia cf. apicalis**	F	E	2019	Le	Togo	1279A05_Rhinia_cf_apicalis_F	1279A05 RMCA	KJ	PZ426587
**Rhinia cf. apicalis**	F	E	2019	Le	Togo	1279A06_Rhinia_cf_apicalis_F	1279A06 RMCA	KJ	PZ426588
**Rhinia cf. apicalis**	F	E	2019	Le	Togo	1279B01_Rhinia_cf_apicalis_F	1279B01 RMCA	KJ	PZ426590
**Rhinia cf. apicalis**	F	E	2019	Le	Togo	1279B03_Rhinia_cf_apicalis_F	1279B03 RMCA	KJ	*
**Rhinia cf. apicalis**	F	E	2019	Le	Togo	1279B06_Rhinia_cf_apicalis_F	1279B06 RMCA	KJ	PZ426592
**Rhinia cf. apicalis**	F	E	2019	Le	Togo	1279B08_Rhinia_cf_apicalis_F	1279B08 RMCA	KJ	PZ426594
**Rhinia cf. apicalis**	F	E	2019	Le	Togo	1279C02_Rhinia_cf_apicalis_F	1279C02 RMCA	KJ	PZ426596
**Rhinia cf. apicalis**	F	E	2019	Le	Togo	1279C03_Rhinia_cf_apicalis_F	1279C03 RMCA	KJ	PZ426597
**Rhinia cf. apicalis**	F	E	2019	Le	Togo	1279C08_Rhinia_cf_apicalis_F	1279C08 RMCA	KJ	PZ426602
**Rhinia cf. apicalis**	F	E	2019	Le	Togo	1279E02_Rhinia_cf_apicalis_F	1279E02 RMCA	KJ	PZ426607
**Rhinia cf. apicalis**	F	E	2019	Le	Ghana	1279E04_Rhinia_cf_apicalis_F	1279E04 RMCA	KJ	PZ426609
**Rhinia cf. apicalis**	F	E	2019	Le	Ghana	1279E08_Rhinia_cf_apicalis_F	1279E08 RMCA	KJ	PZ426611
**Rhinia cf. apicalis**	F	E	2019	Le	Ghana	1279F02_Rhinia_cf_apicalis_F	1279F02 RMCA	KJ	PZ426613
**Rhinia cf. apicalis**	M	E	2019	Le	Ghana	1279F06_Rhinia_apicalis_M	1279F06 RMCA	KJ	PZ426617
**Rhinia cf. apicalis**	M	E	2019	Le	South Africa	1282A08_Rhinia_cf_apicalis_M	1282A08 RMCA	KJ	PZ426622
**Rhinia cf. apicalis**	M	E	2019	Le	South Africa	1282B06_Rhinia_apicalis_M	1282B06 RMCA	KJ	PZ426626
***Rhinia* sp. 1**	M	E	2019	Le	Togo	1279B02_Rhinia_sp1_M	1279B02 RMCA	KJ	PZ426591
***Rhinia* sp. 2**	F	P	2011	Ab	Burundi	R2_Rhinia_sp2_F	MZSUR 03	ATC	PZ426717
***Rhinia* sp. 3**	M	E	2019	Le	Togo	1279B07_Rhinia_sp3_M	1279B07 RMCA	KJ	PZ426593
***Rhinia* sp. 4**	F	E	2019	Le	Ghana	1279F04_Rhinia_sp4_F	1279F04 RMCA	KJ	PZ426615
***Rhinia* sp. 5**	M	E	2019	Le	South Africa	1282C07_Rhinia_sp5_M	1282C07 RMCA	KJ	PZ426630
***Rhinia* sp. 6**	F	E	2019	Le	Ghana	1279F05_Rhinia_sp6_F	1279F05 RMCA	KJ	PZ426616
***Rhinia* sp. 7**	F	E	2019	Le	Ghana	1279F03_Rhinia_sp7_F	1279F03 RMCA	KJ	PZ426614
***Rhinia* sp. 8**	M	E	2019	Le	Togo	1279C05_Rhinia_sp8_M	1279C05 RMCA	KJ	PZ426599
***Rhinia* sp. 9**	F	E	2012	Ab	Tanzania	K4_Rhinia_sp9_F	ZMUC	ATC	PZ426712
***Rhinia coxendix***	M	P	2010	Ab	DR Congo	R3_Rhinia_coxendix_M	BMSA (BECE) (D) 02554	ATC	PZ426719
***Rhyncomya cassotis***	M	P	2016	Ab	Malawi	Y35_Rhyncomya_cassotis_M	BMSA (D) 92323	ATC	PZ426778
***Rhyncomya cassotis***	F	P	2016	Ab	Malawi	Y38_Rhyncomya_cassotis_F	BMSA (D) 92510	ATC	PZ426781
***Rhyncomya cassotis***	F	P	2012	Ab	Namibia	Y39_Rhyncomya_cassotis_F	BMSA (D) 33391	ATC	PZ426782
***Rhyncomya dasyops***	F	P	2006	Ab	South Africa	Y47_Rhyncomya_dasyops_F	NMSA-DIP 19541	ATC	PZ426787
***Rhyncomya disclusa***	M	E	2019	Le	South Africa	1306A08_Rhyncomya_disclusa_M	1306A08 RMCA	KJ	PZ426641
***Rhyncomya disclusa***	F	E	2019	Le	South Africa	1306E03_Rhyncomya_disclusa_F	1306E03 RMCA	KJ	PZ426658
***Rhyncomya disclusa***	F	E	2019	Le	South Africa	1306E04_Rhyncomya_disclusa_F	1306E04 RMCA	KJ	PZ426659
***Rhyncomya disclusa***	F	E	2019	Le	South Africa	1306E05_Rhyncomya_disclusa_F	1306E05 RMCA	KJ	PZ426660
***Rhyncomya forcipata***	F	E	2016	Ab	South Africa	Y15_Rhyncomya_forcipata_F	SAMC-DIP-A015262	ATC	PZ426768
***Rhyncomya forcipata***	F	P	2014	Ab	South Africa	Y27_Rhyncomya_forcipata_F	BMSA (D) 62658	ATC	PZ426774
***Rhyncomya forcipata***	F	P	2009	Ab	South Africa	Y28_Rhyncomya_forcipata_F	BMSA (D) 16949	ATC	PZ426775
***Rhyncomya forcipata***	M	P	2012	Ab	Namibia	Y29_Rhyncomya_forcipata_M	BMSA (D) 48387	ATC	PZ426776
***Rhyncomya interclusa***	M	P	2012	Ab	South Africa	Y69_Rhyncomya_interclusa_M	BMSA (D) 41201	ATC	PZ426796
***Rhyncomya interclusa***	M	E	2004	Ab	South Africa	A9_Rhyncomya_interclusa_M	ZMUC	ATC	PZ426673
***Rhyncomya maculata***	M	P	2007	Ab	South Africa	Y20_Rhyncomya_maculata_M	MZSUR 10	ATC	PZ426769
***Rhyncomya minutalis***	F	E	2017	Le	South Africa	USA12_Rhyncomya_minutalis_F	RMCA 003	EB	PZ426759
***Rhyncomya minutalis***	F	E	2015	Ab	South Africa	Y32_Rhyncomya_minutalis_F	SAMC-DIP-A015272	ATC	PZ426777
***Rhyncomya paratristis***	F	P	2014	Ab	South Africa	Y46_Rhyncomya_paratristis_F	BMSA (D) 66026	ATC	PZ426786
***Rhyncomya pruinosa***	M	P	2009	Ab	South Africa	Y23_Rhyncomya_pruinosa_M	BMSA (D) 16807	ATC	PZ426770
***Rhyncomya pruinosa***	M	P	2016	Ab	Malawi	Y24_Rhyncomya_pruinosa_M	BMSA (D) 90551	ATC	PZ426771
***Rhyncomya pruinosa***	F	P	2011	Ab	Kenya	Y25_Rhyncomya_pruinosa_F	BMSA (D) 32933	ATC	PZ426772
***Rhyncomya pruinosa***	F	P	2014	Ab	South Africa	Y26_Rhyncomya_pruinosa_F	BMSA (D) 62625	ATC	PZ426773
***Rhyncomya* sp. 1**	F	P	2013	Ab	Cameroon	Y41_Rhyncomya_sp1_F	BMSA (D) 53937	ATC	PZ426784
***Rhyncomya* sp. 3**	F	P	2009	Ab	South Africa	Y57_Rhyncomya_sp3_F	BMSA (D) 13680	ATC	PZ426789
***Rhyncomya* sp. 3**	F	P	2009	Ab	South Africa	Y40_Rhyncomya_sp3_F	BMSA (D) 16901	ATC	PZ426783
***Rhyncomya* sp. 6**	M	P	2016	Ab	Togo	Y64_Rhyncomya_sp6_M	BMSA (D) 84379	ATC	PZ426793
***Rhyncomya* sp. 6**	F	P	2016	Ab	Togo	Y65_Rhyncomya_sp6_F	BMSA (D) 84376	ATC	PZ426794
***Rhyncomya* sp. 7**	M	P	2012	Ab	South Africa	Y67_Rhyncomya_sp7_M	NMSA 014	ATC	PZ426795
***Rhyncomya* sp. 8**	F	P	2012	Ab	Namibia	Y44_Rhyncomya_sp8_F	BMSA (D) 47698	ATC	PZ426785
***Rhyncomya* sp. 11**	M	P	2016	Ab	Togo	Y36_Rhyncomya_sp11_cf_cassotis_M	BMSA (D) 84398	ATC	PZ426779
***Rhyncomya* sp. 12**	M	P	2012	Ab	Zambia	Y37_Rhyncomya_sp12_cf_cassotis_M	BMSA (D) 48787	ATC	PZ426780
***Rhyncomya* sp. 14**	F	E	2009	Ab	Tanzania	A10_Rhyncomya_sp14_F	ZMUC	ATC	PZ426668
***Rhyncomya* sp. 15**	F	E	1998	Ab	South Africa	Y6_Rhyncomya_sp15_F	SAMC-DIP-A015239	ATC	PZ426792
***Rhyncomya* sp. 16**	M	E	2015	Ab	South Africa	Y13_Rhyncomya_sp16_cf_minutalis_M	SAMC-DIP-A015286	ATC	PZ426767
***Rhyncomya* sp. 19**	F	P	2013	Ab	Cameroon	Y56_Rhyncomya_sp19_F	BMSA (D) 52204	ATC	PZ426788
***Rhyncomya soyauxi***	F	E	2017	WB	Kenya	USA13_Rhyncomya_soyauxi_F	CEUA 009	EB	PZ426760
***Rhyncomya soyauxi***	M	E	2017	Le	Kenya	USA14_Rhyncomya_soyauxi_M	CEUA 033	EB	PZ426761
***Rhyncomya soyauxi***	F	E	2017	Le	Kenya	USA15_Rhyncomya_soyauxi_F	CEUA 034	EB	PZ426762
***Rhyncomya soyauxi***	M	E	2017	Le	Kenya	USA16_Rhyncomya_soyauxi_M	CEUA 006	EB	PZ426763
***Rhyncomya trispina***	M	P	2012	Ab	Namibia	Y58_Rhyncomya_trispina_M	BMSA (D) 33393	ATC	PZ426790
***Rhyncomya trispina***	F	P	2009	Ab	South Africa	Y59_Rhyncomya_trispina_F	BMSA (D) 13699	ATC	PZ426791
***Stegosoma bowdeni***	M	P	2016	Ab	Togo	G1_Stegosoma_bowdeni_M	BMSA (D) 84384	ATC	PZ426690
***Stegosoma bowdeni***	F	P	2016	Ab	Togo	G3_Stegosoma_bowdeni_F	BMSA (D) 84385	ATC	PZ426691
***Stegosoma* sp. 1**	F	P	2010	Ab	DR Congo	G8_Stegosoma_sp1_F	BMSA (D) (BECE 02553)	ATC	PZ426694
***Stegosoma vinculatum***	M	E	2019	Le	Ghana	1279E07_Stegosoma_vinculatum_M	1279E07 RMCA	KJ	PZ426610
***Stegosoma vinculatum***	F	E	2019	Le	Ghana	1279F01_Stegosoma_vinculatum_F	1279F01 RMCA	KJ	PZ426612
***Stegosoma vinculatum***	F	P	1993	Ab	South Africa	G5_Stegosoma_vinculatum_F	BMSA (D) 00706	ATC	PZ426692
***Stegosoma vinculatum***	M	P	2010	Ab	South Africa	G7_Stegosoma_vinculatum_M	BMSA (D) 29274	ATC	PZ426693
***Stegosoma wellmani***	M	E	2019	Le	Togo	1279C01_Stegosoma_wellmani_M	1279C01 RMCA	KJ	PZ426595
***Stomorhina apta***	F	P	2010	Ab	Burundi	S19_Stomorhina_apta_F	BMSA (D) 24761	ATC	PZ426724
***Stomorhina apta***	F	P	2010	Ab	Burundi	S20_Stomorhina_apta_F	BMSA (D) 24762	ATC	PZ426726
***Stomorhina armatipes***	M	P	2015	Ab	South Africa	S9_Stomorhina_armatipes_M	CEUA 005	ATC	PZ426743
***Stomorhina chapini***	M	P	2009	Ab	South Africa	S4_Stomorhina_chapini_M	BMSA (D) 19118	ATC	PZ426736
***Stomorhina chapini***	F	P	2010	Ab	DR Congo	S2_Stomorhina_chapini_F	BMSA (D) (BECE 00890)	ATC	PZ426725
***Stomorhina chapini***	F	E	2019	Le	Togo	1279A07_Stomorhina_chapini_F	1279A07 RMCA	KJ	PZ426589
***Stomorhina chapini***	F	P	2009	Ab	South Africa	S3_Stomorhina_chapini_F	BMSA (D) 16483	ATC	PZ426733
***Stomorhina cribrata***	F	E	2019	Le	Ghana	1279A01_Stomorhina_cribrata_F	1279A01 RMCA	KJ	PZ426583
***Stomorhina cribrata***	M	E	2019	Le	Togo	1279C06_Stomorhina_cribrata_M	1279C06 RMCA	KJ	PZ426600
***Stomorhina cribrata***	F	E	2019	Le	Togo	1279D01_Stomorhina_cribrata_F	1279D01 RMCA	KJ	PZ426603
***Stomorhina cribrata***	M	E	2019	Le	Togo	1279D02_Stomorhina_cribrata_M	1279D02 RMCA	KJ	PZ426604
***Stomorhina cribrata***	F	E	2019	Le	Togo	1279E01_Stomorhina_cribrata_F	1279E01 RMCA	KJ	PZ426606
***Stomorhina cribrata***	M	E	2019	Le	South Africa	1282C02_Stomorhina_cribrata_M	1282C02 RMCA	KJ	PZ426628
***Stomorhina cribrata***	F	E	2019	Le	South Africa	1306B06_Stomorhina_cribrata_F	1306B06 RMCA	KJ	PZ426646
***Stomorhina cribrata***	M	E	2019	Le	South Africa	1306C03_Stomorhina_cribrata_M	1306C03 RMCA	KJ	PZ426649
***Stomorhina cribrata***	F	E	2019	Le	South Africa	1306E02_Stomorhina_cribrata_F	1306E02 RMCA	KJ	PZ426657
***Stomorhina cribrata***	F	E	2019	Le	South Africa	1306F05_Stomorhina_cribrata_F	1306F05 RMCA	KJ	PZ426664
***Stomorhina cribrata***	F	E	2019	Le	South Africa	1306F06_Stomorhina_cribrata_F	1306F06 RMCA	KJ	PZ426665
***Stomorhina cribrata***	F	E	2019	Le	South Africa	1306F07_Stomorhina_cribrata_F	1306F07 RMCA	KJ	PZ426666
***Stomorhina cribrata***	F	E	2019	Le	South Africa	1306F08_Stomorhina_cribrata_F	1306F08 RMCA	KJ	PZ426667
***Stomorhina cribrata***	M	P	2013	Ab	Tanzania	S45_Stomorhina_cribrata_M	ZMUC	ATC	PZ426739
***Stomorhina guttata***	F	P	2008	Ab	South Africa	S16_Stomorhina_guttata_F	BMSA (D) 01831	ATC	PZ426722
***Stomorhina guttata***	M	P	2010	Th	South Africa	S17_Stomorhina_guttata_M	BMSA (D) 29275	ATC	PZ426723
***Stomorhina guttata***	F	E	2019	Le	South Africa	1306B08_Stomorhina_guttata_F	1306B08 RMCA	KJ	PZ426647
***Stomorhina lunata***	M	E	2019	Le	South Africa	1282A01_Stomorhina_lunata_M	1282A01 RMCA	KJ	PZ426618
***Stomorhina lunata***	F	E	2019	Le	South Africa	1282A06_Stomorhina_lunata_F	1282A06 RMCA	KJ	PZ426621
***Stomorhina lunata***	M	E	2019	Le	South Africa	1282C01_Stomorhina_lunata_M	1282C01 RMCA	KJ	PZ426627
***Stomorhina lunata***	M	E	2019	Le	South Africa	1282C03_Stomorhina_lunata_M	1282C03 RMCA	KJ	PZ426629
***Stomorhina lunata***	F	E	2019	Le	South Africa	1282C08_Stomorhina_lunata_F	1282C08 RMCA	KJ	PZ426631
***Stomorhina lunata***	M	E	2019	Le	South Africa	1282D02_Stomorhina_lunata_M	1282D02 RMCA	KJ	PZ426633
***Stomorhina lunata***	F	E	2019	Le	South Africa	1299A01_Stomorhina_lunata_F	1299A01 RMCA	KJ	PZ426635
***Stomorhina lunata***	F	E	2019	Le	South Africa	1299A02_Stomorhina_lunata_F	1299A02 RMCA	KJ	PZ426636
***Stomorhina lunata***	F	E	2019	Le	South Africa	1299A03_Stomorhina_lunata_F	1299A03 RMCA	KJ	PZ426637
***Stomorhina lunata***	F	E	2019	Le	South Africa	1306A01_Stomorhina_lunata_F	1306A01 RMCA	KJ	PZ426638
***Stomorhina lunata***	F	E	2019	Le	South Africa	1306B01_Stomorhina_lunata_F	1306B01 RMCA	KJ	PZ426642
***Stomorhina lunata***	M	E	2019	Le	South Africa	1306C01_Stomorhina_lunata_M	1306C01 RMCA	KJ	PZ426648
***Stomorhina lunata***	F	E	2019	Le	South Africa	1306D03_Stormohina_lunata_F	1306D03 RMCA	KJ	PZ426654
***Stomorhina lunata***	F	E	2019	Le	South Africa	1306D05_Stomorhina_lunata_F	1306D05 RMCA	KJ	PZ426656
***Stomorhina lunata***	F	P	2016	Ab	Mauritius	S21_Stomorhina_lunata_F	BMSA (D) 87119	ATC	PZ426727
***Stomorhina lunata***	M	P	2016	Ab	Mauritius	S22_Stomorhina_lunata_M	BMSA (D) 86965	ATC	PZ426728
***Stomorhina lunata***	M	P	2016	Ab	Malawi	S23_Stomorhina_lunata_M	BMSA (D) 90545	ATC	PZ426729
***Stomorhina lunata***	F	P	2016	Ab	Malawi	S24_Stomorhina_lunata_F	BMSA (D) 90549	ATC	PZ426730
***Stomorhina lunata***	M	P	2015	Ab	South Africa	S26_Stomorhina_lunata_M	CEUA 023	ATC	PZ426731
***Stomorhina lunata***	F	P	2015	Ab	South Africa	S7_Stomorhina_lunata_F	CEUA 003	ATC	PZ426741
***Stomorhina lunata***	F	P	2015	Le	South Africa	USA18_Stomorhina_lunata_F	CEUA 027	EB	PZ426764
***Stomorhina lunata***	M	P	2015	WB	South Africa	USA19_Stomorhina_lunata_M	CEUA 024	EB	PZ426765
***Stomorhina malobana***	M	P	2016	Ab	Malawi	S31_Stomorhina_malobana_M	BMSA (D) 90534	ATC	PZ426735
***Stomorhina rugosa***	M	E	2019	Le	Togo	1279D05_Stomorhina_rugosa_M	1279D05 RMCA	KJ	PZ426605
***Stomorhina rugosa***	M	E	2019	Le	South Africa	1282D01_Stomorhina_rugosa_M	1282D01 RMCA	KJ	PZ426632
***Stomorhina rugosa***	F	E	2019	Le	South Africa	1282D03_Stomorhina_rugosa_F	1282D03 RMCA	KJ	PZ426634
***Stomorhina rugosa***	M	P	2016	Ab	Malawi	S13_Stomorhina_rugosa_M	BMSA (D) 92522	ATC	PZ426720
***Stomorhina rugosa***	F	P	?2017	Ab	Ethiopia	S14_Stomorhina_rugosa_F	NMSA 010	ATC	PZ426721
***Stomorhina* sp. 1**	F	P	2015	Ab	South Africa	S5_Stomorhina_sp1_cf_armatipes_F	CEUA 030	ATC	PZ426740
***Stomorhina* sp. 1**	F	P	2015	Ab	South Africa	S8_Stomorhina_sp1_cf_armatipes_F	CEUA 032	ATC	PZ426742
***Stomorhina* sp. 2**	F	P	2016	Ab	Malawi	S28_Stomorhina_sp2_cf_malobana_F	BMSA (D) 90539	ATC	PZ426732
***Stomorhina* sp. 2**	F	P	2016	Ab	Malawi	S30_Stomorhina_sp2_cf_malobana_F	BMSA (D) 91878	ATC	PZ426734
***Stomorhina* sp. 2**	F	E	2007	Ab	Tanzania	S40_Stomorhina_sp2_cf_malobana_F	ZMUC	ATC	PZ426737
***Stomorhina* sp. 2**	F	E	2007	Ab	Tanzania	S42_Stomorhina_sp2_cf_malobana_F	ZMUC	ATC	PZ426738
***Thoracites petersiana***	F	P	2009	Ab	South Africa	T6_Thoracites_petersiana_F	BMSA (D) 17221	ATC	PZ426746
***Thoracites petersiana***	M	P	2009	Ab	South Africa	T7_Thoracites_petersiana_M	BMSA (D) 13702	ATC	PZ426747
***Thoracites* sp. 1**	M	E	2016	Ab	South Africa	T1_Thoracites_sp1_M	SAMC-DIP-A014257	ATC	PZ426744
***Thoracites* sp. 1**	F	E	2016	Ab	South Africa	T3_Thoracites_sp1_F	SAMC-DIP-A015258	ATC	PZ426745
***Trichoberia* sp. 1**	M	P	2007	Ab	South Africa	B1_Trichoberia_sp1_M	NMSA-DIP 84380	ATC	PZ426674
***Vanemdenia africana***	M	E	2011	Ab	?Tanzania	A12_Vanemdenia_africana_F	ZMUC	ATC	PZ426669
***Zumba antennalis***	F	E	2016	Ab	South Africa	Y10_Zumba_antennalis_F	SAMC-DIP-A015288	ATC	PZ426766
***Zumba antennalis***	F	E	2016	Ab	South Africa	Z2_Zumba_antennalis_F	SAMC-DIP-A015280	ATC	PZ426797

*Specimen 1279B03 was included in the alignment and analyses but was not deposited in GenBank because the sequence contained an internal stop codon that could not be verified against the original chromatogram.

Male and female terminalia were dissected after DNA extraction, taking advantage of tissue softening by proteinase K digestion. Abdomens were rinsed in 70% ethanol, dissected, and preserved in 96% ethanol. Terminalia were stored in microvials with glycerin for subsequent morphological studies. Microvials were either pinned with their respective dry specimens or stored in the same container as their ethanol-preserved specimen. Detached abdomens were dried, glued on cardboard, and pinned with their respective specimens. This method avoids the use of KOH and acetic acid (CH3–COOH), preserving specimens for future morphological studies. Before the abdomens were detached, specimens were photographed as described in Thomas-Cabianca et al. (2021).

### DNA extraction, PCR amplification, and sequencing

DNA extraction, amplification, purification, and sequencing were carried out by ATC, EB, and KJ. For ATC and EB samples, muscle tissue from the abdomen, thorax, and/or legs was used (Table 1). Genomic DNA was extracted by proteinase K digestion using the DNeasy Blood & Tissue Kit (Qiagen), following the manufacturer’s protocol, but to maximize DNA yield the Proteinase K digestion ran for 48 h at 56 °C and DNA was eluted twice in 50 μL (total volume 100 μL) as described in Buenaventura (2021). DNA concentrations were quantified with the Qubit 2.0 dsDNA HS (ThermoFisher) using 2 µL of sample. The 658 bp mitochondrial cytochrome *c* oxidase subunit I (COI) fragment (DNA barcode) (Hebert et al. 2003b) was amplified using primers TY-J-1460 and C1-N-2191 (Simon et al. 1994; Bernasconi et al. 2000, Suppl. material 1).

PCR reactions for ATC samples (25 µL) contained 4 µL DNA template, 4 µL HOT FIREPol Blend Master Mix (Solis BioDyne), 0.5 µL of each primer, and 16 µL distilled, autoclaved water. Cycling conditions: initial denaturation at 95 °C for 2 min; 29 cycles of 94 °C for 30 s, annealing at 47.5 °C for 30 s, extension at 72 °C for 2 min; and final extension at 72 °C for 8 min. PCR reactions for EB samples (15 µL) contained 1 µL DNA template, 7.5 µL GoTaq Green Master Mix (Promega), 0.4 µL of each primer, and 5.7 µL distilled, autoclaved water. Cycling conditions: initial denaturation at 95 °C for 5 min; 40–50 cycles of 95 °C for 60 s, annealing at 45 °C for 60 s, extension at 72 °C for 2 min; and final extension at 72 °C for 10 min (Suppl. material 1).

PCR products were electrophoresed in 2% agarose gels stained with GelRed® (Merck; 1 µL/10 mL gel), with 3 µL of sample and 2 µL of a 100 bp ladder (Invitrogen), run at 115 V for 35 min. Samples with clear single bands (650–750 bp) were sequenced directly; those with polymorphic bands were gel-extracted prior to sequencing. For ATC, PCR products were processed at the Centre for GeoGenetics, University of Copenhagen (Denmark). The PCR clean-up and sequencing were performed by Macrogen Europe BV (Amsterdam, Netherlands). For EB, PCR products were sequenced on an ABI 3100 genetic analyzer using Big Dye Cycle Sequencing chemistry at the Laboratory of Analytical Biology, National Museum of Natural History, Smithsonian Institution (USA).

For KJ samples (Table 1) the protocol from Jordaens et al. (2015) was followed. Briefly, genomic DNA was extracted from a single leg using the NucleoSpin Tissue Kit (Macherey-Nagel), following manufacturer instructions. PCR reactions (25 µL) contained 1.5 mM MgCl_2_ in 1× PCR buffer (Invitrogen), 0.2 mM of each dNTP, 0.2 µM of each primer, and 0.5 U Taq polymerase (Invitrogen). COI was amplified using primers LCO1490 and HCO2198 (Folmer et al. 1994, Suppl. material 1). PCR cycling conditions were an initial denaturation at 95 °C for 5 min followed by 35 cycles of 95 °C for 45 s, annealing at 45 °C for 45 s, extension at 72 °C for 1.5 min; and a final extension at 72 °C for 5 min (Suppl. material 1). PCR products were purified using ExoSap (Invitrogen). Bi-directional sequencing was done at Macrogen (The Netherlands).

### DNA barcode alignment

Sequences were edited and assembled using De Novo Assembly with ‘High Sensitivity/Medium’ parameters in Geneious v. 9.0.5 (Biomatters Ltd.) (Kearse et al. 2012), with ends trimmed at an error probability limit of 0.05. Alignments were generated using MUSCLE in MEGA-X v. 10.0.5 (Kumar et al. 2018) and translated to amino acids for error checking.

The dataset included 216 newly generated COI DNA barcode sequences (>500 bp) from 13 of the 16 Afrotropical genera. No barcodes were obtained for *Pararhyncomyia* Becker, 1910, *Perisiella* Zumpt, 1958, and *Pseudorhyncomyia*. One sequence was shorter than 600 bp (A5 – *Cosmina margaritae* Peris, 1952), and four sequences were obtained from reverse reads only (K5 – *Albaredaya malgache* Peris, 1956; E2 – *Eurhyncomyia diversicolor* (Bigot, 1888); I12 – *Isomyia dubiosa* (Villeneuve, 1917); I8 – *I. distinguenda* (Villeneuve, 1917)). In addition, 60 RhiniinaeCOI DNA barcodes were downloaded from GenBank (Suppl. material 2) and 1,112 from BOLD Systems (Suppl. material 3). Four calyptrate species were used as outgroups: Anthomyiidae: *Hylemya alcathoe* (Walker, 1849); Calliphoridae: *Cordylobia anthropophaga* (Blanchard & Berenger-Feraud, 1872) – FR719158 (McDonagh and Stevens 2011), *Lucilia sericata* (Meigen, 1826) – KY416613 (Buenaventura and Pape 2017); and Sarcophagidae: *Oxysarcodexia trivialis* (Wulp, 1896). The 1,112 COI DNA barcodes downloaded from BOLD Systems included 957 identified only at the subfamily level (BINs), 14 at the genus level (*Rhinia* Robineau-Desvoidy, 1830 (5), *Stomorhina* (9)), and 141 at the species level (*Rhinia apicalis* (Wiedemann, 1830) (3), *Rh. soyauxi* Karsch, 1886 (113), *Stegosoma vinculatum* Loew, 1863 (1), *Stomorhina discolor* (Fabricius, 1794) (23), *St. lunata* (1)).

GenBank sequences (accessed on 20 November 2023; https://www.ncbi.nlm.nih.gov/genbank/) were obtained via BLAST searches (program megablast) using *Stomorhina lunata* (MN868825) as a query, filtered for “Rhiniinae”. BOLD Systems sequences (accessed on 21 November 2023; https://portal.boldsystems.org/) were downloaded using the “Calliphoridae-Rhiniinae” filter. Only sequences >500 bp were retained.

### DNA barcode analysis

Two final COI DNA barcode alignments (~660 bp) were generated. A first alignment contained only the newly generated DNA barcodes and the four outgroups (*n* = 220, Suppl. material 11). The second alignment comprised all Rhiniinae sequences from BOLD Systems, GenBank, the newly generated sequences (1,388 sequences), and the four outgroups (*n* = 1,392, Suppl. material 12). Identical sequences were removed with DAMBE v. 7 (Xia 2018). Neighbour-Joining (NJ) and Maximum Likelihood (ML) trees (Guindon and Gascuel 2003) were constructed for each alignment. NJ trees were reconstructed in MEGA-X v. 10.0.5 under the Kimura 2-parameter (K2P) model with 500 bootstrap (BS) replicates. ML analyses used codon-position partitioning with models selected by jModelTest v. 2.1.10 (Posada 2008; Darriba et al. 2012): GTR+I+G (1^st^ position, AICc = 44779.001), TN93+G+I (2^nd^ position, AICc = 44857.605), GTR+G (3^rd^ position, AICc = 44996.942). ML analyses were run in RAxML (Stamatakis 2014) with 100 replicates; node support was assessed with 300 bootstrap pseudo-replicates with search reps set to one. A consensus tree was built in RAxML, combining the bootstrap trees and plotting them on the best tree. The NJ and ML analyses were used as exploratory tools to visualize clustering patterns and assess reciprocal clustering of morpho­species for identification purposes only. Clusters with bootstrap values ≥70% were considered supported and BS < 70% as unsupported.

Species clusters from NJ and ML analyses were compared with those inferred using the Automatic Barcode Gap Definition (ABGD) (Puillandre et al. 2012; https://bioinfo.mnhn.fr/abi/public/abgd/abgdweb.html) and the Assemble Species by Automatic Partitioning (ASAP) (Puillandre et al. 2021; https://bioinfo.mnhn.fr/abi/public/asap/). Only ingroup species were included. ABGD and ASAP analyses were performed via the SPART-Explorer web interface https://spartexplorer.mnhn.fr/ (Bruy et al. 2024). For the ABGD analysis, we used the K80 model with default parameter values, except for the relative gap width (X), which was set to 0.90 (instead of the default 1.50) when higher values failed to detect more than one group (“barcode gap distance” is abbreviated as BG). The ASAP analysis was run with default settings under the JC69 (Jukes-Cantor), K80 (Kimura), and Simple Distance models. Results from the K80 model were selected for comparison. Automated clustering approaches (ABGD and ASAP) were applied to further explore barcode clustering patterns and the consistency of identification. Despite their usefulness for exploring clustering structure, neither ABGD nor ASAP was treated here as a formal species delimitation method, as both rely on a single mitochondrial marker and are sensitive to parameter choice, sampling density, and database composition (Collins and Cruickshank 2013; Phillips et al. 2022).

Pairwise K2P-based genetic distances were used as a quantitative model to evaluate COI DNA barcode performance, following standard practice in DNA barcoding studies (Buenaventura et al. 2018). Pairwise uncorrected K2P-distances within and among species were calculated in MEGA-X v. 10.0.5 with 500 bootstrap replicates.

Species are treated here as morphospecies defined by adult morphology, and molecular results are interpreted strictly in terms of identification success or failure, following established DNA barcoding approaches (Hebert et al. 2003a, 2003b; Meier et al. 2006; Buenaventura et al. 2018; Phillips et al. 2022). Public sequences that did not cluster with voucher-based species from this study were provisionally assigned to genus or species based on specimen photographs and associated metadata provided by the source author in the BOLD Systems. Trees were edited in FigTree v. 1.4.4 (Rambaut 2010) and Inkscape 1.4-beta (Inkscape Project 2025). All sequences, identifications, and metadata are deposited in GenBank (accession numbers: PZ426583–PZ426797).

## Results

**Figure 1.**
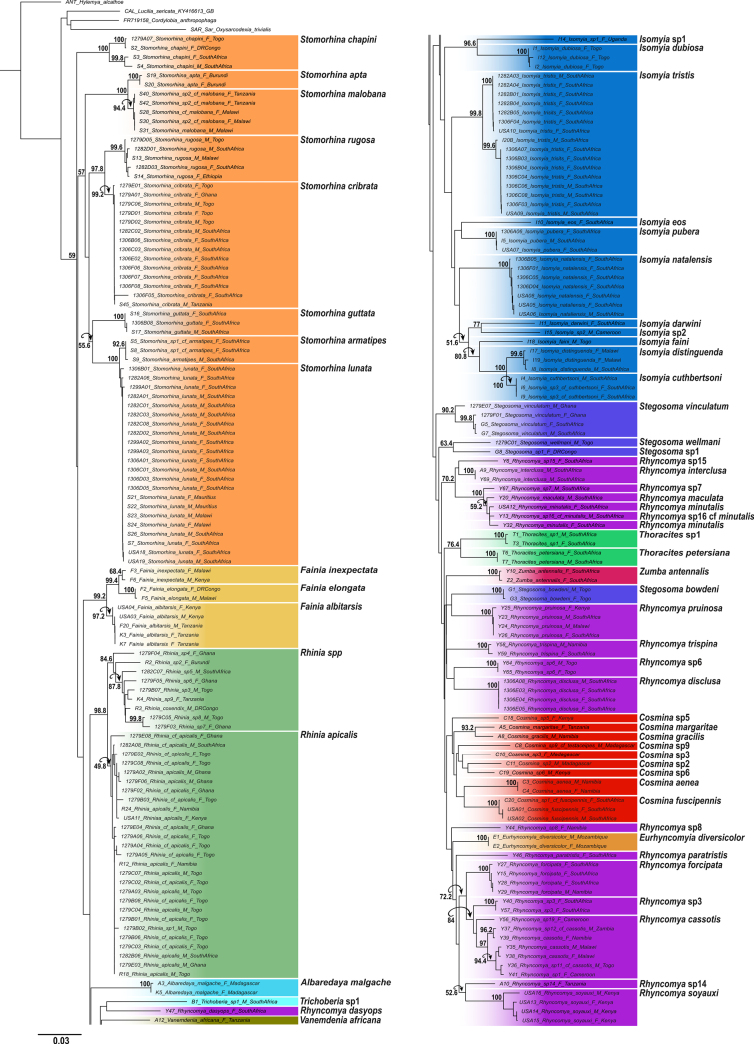
Neighbor-Joining (NJ) tree inferred using the Kimura 2-parameter (K2P) model based on 216 COI DNA barcode sequences of Afrotropical Rhiniinae species and four outgroups. The species *Hylemya alcathoe* (Walker, 1849) (Anthomyiidae) was used to root the tree. Numbers above branches indicate bootstrap (BS) support (500 replicates). Only support values ≥ 50% are shown. Species clusters are highlighted in different colours and labeled with species names.

**Figure 2.**
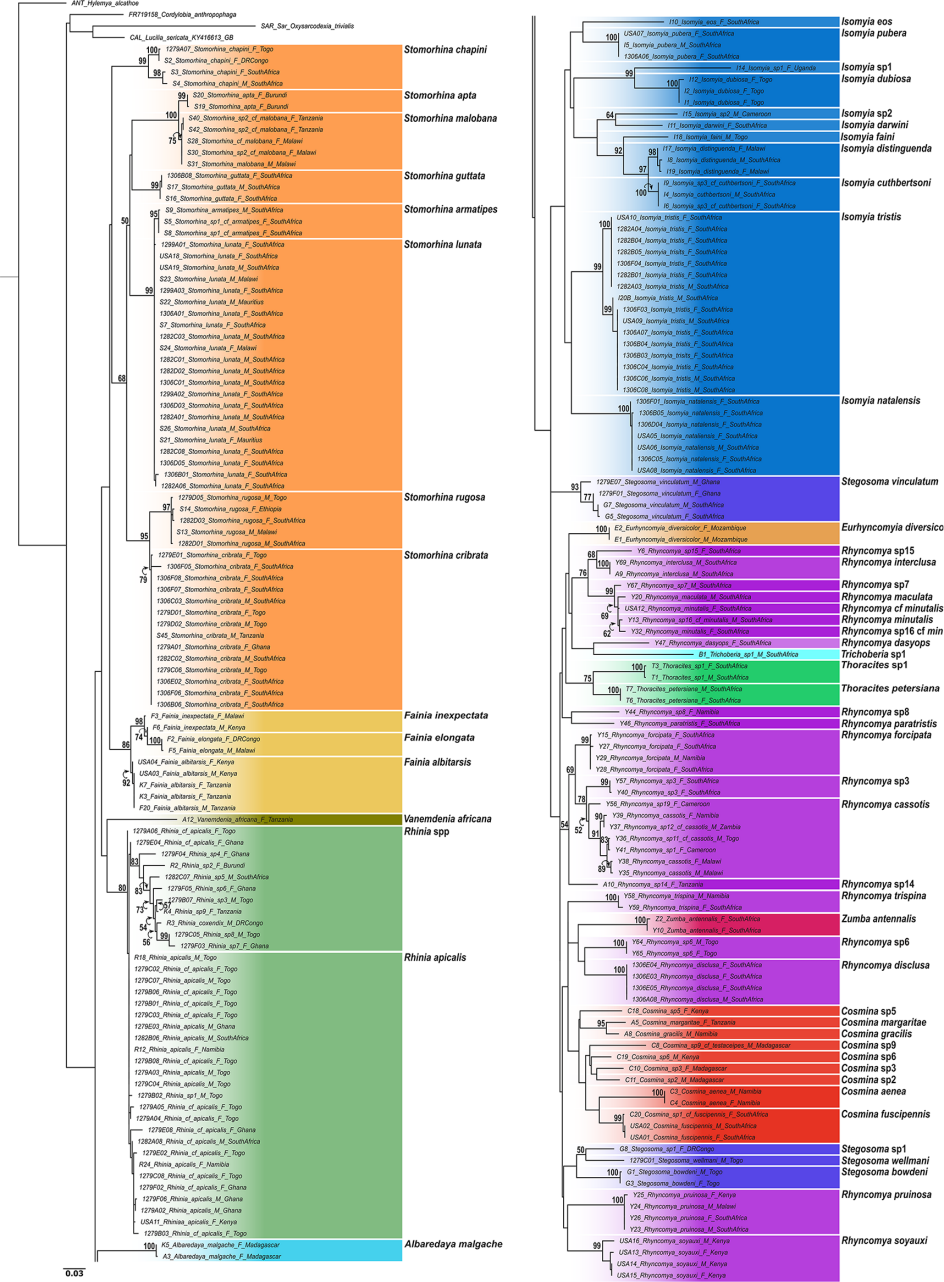
Maximum Likelihood (ML) tree inferred using the GTR+I+G codon-position partitioning model based on 216 COI DNA barcode sequences of Afrotropical Rhiniinae species and four outgroups. The species *Hylemya alcathoe* (Walker, 1849) (Anthomyiidae) was used to root the tree. Numbers above branches indicate bootstrap (BS) support (100 replicates). Only support values ≥ 50% are shown. Species clusters are highlighted in different colours and labelled with species names.

**Figure 3.**
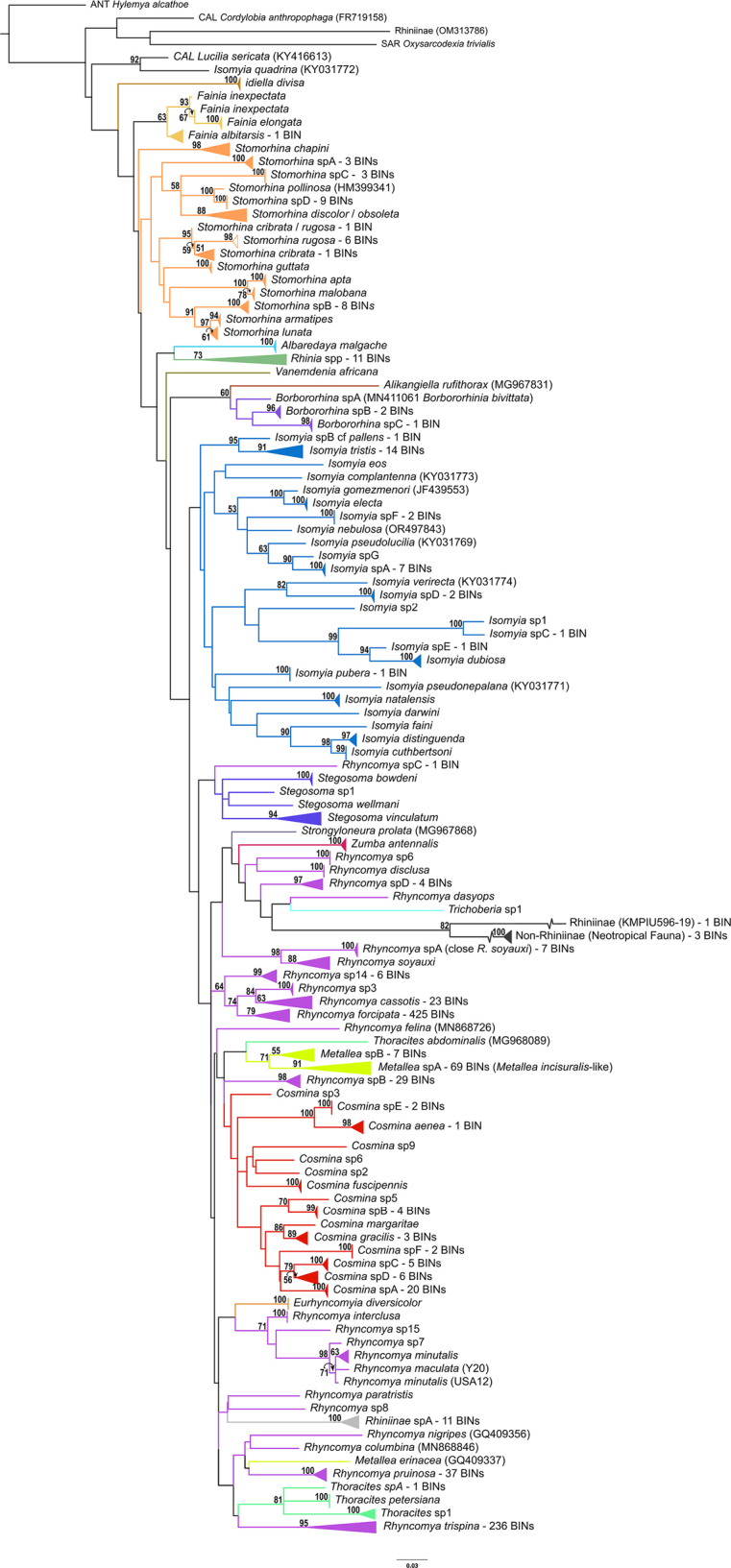
Maximum Likelihood (ML) tree inferred using the GTR+I+G codon-position partitioning model based on 1,388 COI DNA barcode sequences of Rhiniinae species and four outgroups. *Hylemya alcathoe* (Walker, 1849) (Anthomyiinae) was used to root the tree. Numbers above branches indicate bootstrap (BS) support (100 replicates). Only support values ≥ 50% are shown. Species clusters are highlighted in different colours and labelled with species names. Morphospecies in which “sp” is followed by a number (e.g., *Thoracites* sp1) refer to morphospecies that were delimited and identified by us based on examined material and subsequently revised. In contrast, morphospecies in which “sp” is followed by a letter (e.g., *Stomorhina* spB) correspond to entities delimited based exclusively on COI sequences retrieved from BOLD Systems.

We obtained new COI DNA barcode sequences from 216 Rhiniinae specimens. Of these, 156 specimens were assigned to 44 species names, while the remaining 60 specimens could only be identified to genus level and were grouped into 36 morphospecies. In total, 79 putative species were recognized. The dataset comprised 133 females and 83 males, and allowed the successful association of 31 female morphotypes with their conspecific males morphotypes (Figs 1, 2). The analyses of the 216 newly generated barcodes, together with the 1,172 sequences obtained from public repositories (totalling 1,388 Rhiniinae DNA barcodes), allowed the confirmation of putative species identifications (Fig. 3, Suppl. material 10) and the assignment of species or genus names to the majority of the 957 available BINs identified only at the subfamily level in BOLD Systems (see Suppl. material 3, 6, 13).

### DNA barcoding analysis of the Afrotropical Rhiniinae

Based on the NJ and ML analysis, we clustered *Isomyia* and *Rhyncomya* in the following putative species groups: *Rhyncomya*-forcipata group (NJBS = 69%; MLBS =72.2%, excluding *Rhyncomya trispina* Villeneuve, 1929), *-maculata* group (NJBS = 70.2%; MLBS = 76%), *-pruinosa* group (NJ*-*MLBS = 100%), *-soyauxi* group (as *pictifacies*; NJBS = 99%; MLBS = 100%), and *Isomyia*-distinguenda group (NJBS = 80.8%; MLBS = 92%, excluding *Isomyia darwini* (Curran, 1938)) (Figs 1, 2, Suppl. material 4, 5). Also, the species of *Albaredaya* Peris, 1956 (NJ-MLBS = 100%), *Fainia* (NJBS = 86%; MLBS = 99.2%), *Rhinia* (NJBS = 80%; MLBS = 98.8%), and *Thoracites* (NJBS = 75%; MLBS = 76.4%) were retained in supported clusters. (Figs 1, 2, Suppl. material 4, 5). Clustering of species of the other genera showed no support: *Cosmina* Robineau-Desvoidy, 1830 (NJBS = 11.2%; MLBS = 32%), *Isomyia* (NJBS = 16%; MLBS = 23%), and *Stomorhina* (NJBS = 35%; MLBS = 57%) (Figs 1, 2, Suppl. material 4, 5). Species of *Rhyncomya* and *Stegosoma* Loew, 1863 did not cluster in a single group. For *Vanemdenia* Peris, 1951, *Trichoberia* Townsend, 1933, *Eurhyncomyia* Malloch, 1926, and *Zumba* Peris, 1951, we had DNA barcodes for only a single species. The largest genetic interspecific K2P-distances were found between *Trichoberia* sp. (K2P = 0.0867–0.1376) and all other species, while the smallest were between morphologically similar species, with K2P-distance values that ranged from 0.005 to 0.026 (Table 2, Suppl. material 9).

**Table 2.** Interspecific Kimura 2-parameter (K2P) genetic distances among morphologically similar species of the genera *Cosmina* Robineau-Desvoidy, 1830; *Fainia* Zumpt, 1958; *Isomyia* Walker, 1859; *Rhinia* Robineau-Desvoidy, 1830; *Rhyncomya* Robineau-Desvoidy, 1830; and *Stomorhina* Rondani, 1861.

**Species**	**Inter-specific K-distance**
*Cosmina margaritae* + *C. gracilis*	0.02613661
*Fainia albitarsis* + *F. elongata* + *F. inexpectata*	0.01690651–0.02704600
*Isomyia. cuthbertsoni* + *I. distinguenda*	0.02157076
*Rhinia apicalis* + *R. Coxendix*	0.02186814
*Rhyncomya minutalis* + *Rh. maculata* + *Rhyncomya* sp7	0.01124392–0.01685536
*Stomorhina cribrata* + *St. rugosa*	0.02186814
*Stomorhina malobana* + *St. apta*	0.01363106
*Stomorhina lunata* + *St. armatipes*	0.00542285

The ASAP analysis resulted in 62 groups according to the lower ASAP score for each model (JC69 = 1.50, K80 = 2.50, Simple Distance = 1.50), and the threshold distance proposed for the newly 216 COI DNA barcode sequence data set, was between 0.016835–0.017026 (Suppl. material 7, 8). The ABGD analysis gave six partitions according to the K80 model (Suppl. material 7). Partitions five and six proposed 59 species groups (p = 0.00774–0.0129, BG = 0.019%). Partition four proposed 78 species (p = 0.00464, BG = 0.007%) (Suppl. material 7–8), separating morphologically similar species, such as *Cosmina gracilis* Curran, 1927 and *C. margaritae*; *Fainia albitarsis* (Macquart, 1846), *F. elongata* (Bezzi, 1908), and *F. inexpectata* Zumpt, 1973; *Isomyia cuthbertsoni* (Curran, 1938) and *I. distinguenda*, *Rhyncomya minutalis* Villeneuve, 1927, and *Rh. maculata* Macquart, 1846; *Stomorhina malobana* (Lehrer, 2007) and *St. apta* Curran, 1931; *St. lunata* and *St. armatipes* (Malloch, 1926); *St. cribrata* (Bigot, 1874) and *St. rugosa* (Bigot, 1888); and the genus *Rhinia*.

### Species identification using COI DNA barcodes from GenBank and BOLD Systems

We analyzed 60 Rhiniinae DNA barcodes mined from GenBank (Suppl. material 2). The ML analysis grouped sequences previously identified in GenBank only to subfamily (10) or genus (3) within specific species or genera (Fig. 3, Suppl. material 6, 13). Information regarding *Idiella divisa* (Walker, 1861) (KY031805, KY031806), *Isomyia quadrina* Fang & Fan, 1985 (KY031772), and RhiniinaeOM313786 cannot be provided, as they are not clustered with congenerics. Regarding BOLD Systems sequences, the ML analysis assigned the majority of the 957 BINs to genus or species level (Fig. 3, Suppl. material 6, 13). The ASAP analysis suggested 92 species groups according to the lowest ASAP score, with a threshold of 0.027077, while the ABGD analysis produced seven partitions (Suppl. material 7, 10). Partition six proposed 94 groups (p = 0.012915, BG = 0.026%), partition seven 89 groups (p = 0.021544, BG = 0.031%), and partition five 109 groups (p = 0.007743, BG = 0.018%). Partition six separated a large part of the species, but morphologically similar species were not separated (Suppl. material 10); however, with partition five, many of the morphologically similar species were separated into independent groups. Integrating the ML, ASAP, and ABGD results with the examination of the images provided by the author of the sequences in BOLD Systems (Suppl. material 13), a total of 749 unnamed BINs (out of the 957 BINs) were assigned to 11 species names (= *Cosmina aenea* (Fabricius, 1805) (1), *C. gracilis* (3), *Fainia albitarsis* (1), *Isomyia pubera* (Villeneuve, 1917) (1), *I. tristis* (Bigot, 1888) (14), *Rhyncomya cassotis* (Walker, 1849) (23), *Rh. forcipata* Villeneuve, 1927 (425), *Rh. pruinosa* Villeneuve, 1922 (37), *Rh. trispina* (236), *Stomorhina cribrata* (1), *St. rugosa* (6), and *St. cribrata* / rugosa (1), Suppl. material 13) and 193 unnamed BINs were assigned to a genus name, within 24 morphospecies: *Borbororhinia* Townsend, 1917 (3 BINs, 2 morphospecies), *Cosmina* (33 BINs, 6 morphospecies), *Isomyia* (14 BINs, 6 morphospecies), *Metallea* Wulp, 1880 (73 BINs, 2 morphospecies), *Rhinia* (11 BINs), *Rhyncomya* (47 BINs, 5 morphospecies), *Stomorhina* (11 BINs, 2 morphospecies) and *Thoracites* (1 BIN and morphospecies), see Suppl. material 13. The remaining 15 unnamed BINs did not cluster with our morphologically identified species, they remained as Rhiniinae spA (11 BINs), and Rhiniinae-KMPIU596_19 (1 BIN). Three non-Rhiniinae BINs from the Neotropics were detected after reviewing metadata and photographs provided in BOLD Systems. Additionally, three other COI DNA sequences were incorrectly identified in BOLD Systems and were reassigned to other genera (Suppl. material 13).

## Discussion

### DNA barcoding of Afrotropical Rhiniinae, morphology- and molecular-based identifications

Although this study does not aim to revise species boundaries, redefine generic limits, or infer phylogenetic relationships within Rhiniinae, overall, COI DNA barcodes proved effective in supporting the reciprocal clustering of most morphologically defined species represented by at least two specimens and showed strong congruence with morphology-based identifications. Thirty-six (in ML analysis; Fig. 2) and 37 (in NJ analysis; Fig. 1) of the 37 morphospecies that were represented by multiple specimens, formed clear, exclusive barcode clusters and could be reliably identified through DNA barcoding. This pattern aligns with findings from other Oestroidea families, including Calliphoridae, Mesembrinellidae, Oestridae, Polleniidae, and Sarcophagidae, where COI has proven useful for routine species identification despite known limitations (Sonet et al. 2013; Yusseff-Vanegas and Agnarsson 2017; Buenaventura et al. 2018; Li et al. 2019; Whitworth and Yusseff-Vanegas 2019; Szpila et al. 2023). The identification success was observed in monotypic genera such as *Albaredaya* and *Vanemdenia*, as well as in the species-poor genera *Eurhyncomyia* (*Eurhyncomyia diversicolor*), *Trichoberia* (*Trichoberia* sp1) and *Zumba* (*Zumba antennalis* (Villeneuve, 1929)), where molecular and morphological identifications were consistent. Similarly, all the species of *Isomyia*, *Stegosoma*, *Stomorhina*, and *Thoracites* studied were recovered as distinct barcode clusters consistent with their morphological diagnoses.

Despite the overall success of COI barcoding, several morphologically similar species, particularly those whose identification relies heavily on male terminalia, exhibited low interspecific divergence or partial barcode overlap, thereby limiting unambiguous molecular identification (Table 2). This was particularly common in the genera *Fainia*, *Rhinia*, and several species of *Stomorhina*. In *Fainia*, the three described species showed lower interspecific K2P-distances yet remain clearly diagnosable based on male terminalia morphology (Thomas-Cabianca et al. 2021). Similarly, in the species pairs *Stomorhina apta* and *St. malobana*, and in *St. armatipes* and *St. lunata*, COI DNA barcodes showed low divergence despite morphological differentiation. Similar discordance between mitochondrial genetic divergence and morphology has been widely documented in genera comprising morphologically similar species of Calyptratae flies (Meier et al. 2006; Meiklejohn et al. 2011; Sonet et al. 2013; GilArriortua et al. 2014; Buenaventura et al. 2018; Li et al. 2019; Martínez-Sánchez et al. 2025). These cases likely reflect recent divergence, incomplete lineage sorting, mitochondrial introgression, or limited resolution of single-locus mitochondrial markers. They highlight the inherent limits of the COI DNA barcode for species identification.

Overall, NJ and ML analyses produced largely congruent species-level clustering, although support values varied among nodes and were notably lower in species-rich genera with shallow mitochondrial divergence. According to the traditional DNA barcode approach, effective species-level resolution typically occurs when intraspecific divergences are below 3%, and interspecific divergences exceed this threshold (Hebert et al. 2003a, 2003b). However, the Rhiniinae dataset clearly shows that many valid morphospecies exhibit interspecific divergences far below 3% (Table 2, Suppl. material 9), suggesting that a universal genetic distance threshold is unsuitable for assessing identification accuracy in this group, as previously reported for *Lucilia* Robineau-Desvoidy, 1830 and *Calliphora* Robineau-Desvoidy, 1830 (Sonet et al. 2013; GilArriortua et al. 2014; Martínez‐Sánchez et al. 2025; Parmar et al. 2026), among others. Alternative partitions recovered in ABGD analyses frequently produced different clustering solutions for the same dataset, particularly in taxa characterized by low interspecific divergence or high intraspecific variation. However, when combined with NJ and ML clustering analyses and grounded in morphology-based species hypotheses, ABGD and ASAP offer a useful complementary framework for testing identification consistency across datasets, rather than serving as standalone species delimitation tools. A comparative overview of clustering outcomes from NJ, ML, ABGD, and ASAP analyses for the full dataset of 1,388 Rhiniinae sequences is provided in Suppl. material 13. This overview highlights cases of concordance among methods and taxa, as well as taxa for which identification outcomes vary across analytical approaches and species groups that warrant further taxonomic revision (e.g., the genera *Rhinia* and *Rhyncomya*). Together, these results reinforce the need to interpret COI barcoding within a comparative framework that combines distance-based metrics, tree-based clustering, and morphological evidence, rather than relying on any single method or threshold. DNA barcoding performance varied considerably across Rhiniinae genera, correlating with differences in sampling intensity, morphological divergence, and inferred evolutionary history. The most complex patterns were observed in *Rhyncomya*, whose species were recovered in multiple distinct clades. Historically, this genus has served as a “residual group” for species not assignable to better-defined genera and it has repeatedly been subdivided into species groups, with some authors advocating generic splitting (Townsend 1917; Villeneuve 1927; Peris 1952b; Zumpt 1958). Several species groups showed internal coherence, whereas others could not be tested adequately due to limited sampling. Our results support the monophyly of two of the 11 *Rhyncomya* species groups proposed by Zumpt (1958): the *Rhyncomya-forcipata* species group (excluding *Rh. trispina*) and the *Rhyncomya-maculata* species group, while six of the seven Afrotropical species groups proposed by Peris (1952b) had partial recovery (because we lacked some of the species in the analyses): the *Rhyncomya-divisa* species group, the *Rhyncomya-forcipata* species group (excluding *Rh. trispina*), the *Rhyncomya-maculata* species group, the *Rhyncomya*-*pictifacies* species group, the *Rhyncomya*-pruinosa species group, and the *Rhyncomya-tetropsis* species group. In the *Rhyncomya cassotis* complex, geographically structured lineages (ML and NJ analyses = Clade 1: Y35 (M) + Y38 (F), BS: 89–70.2; Clade 2: Y36 (M) + Y41 (F), BS: 83–93.2; Clade 3: Y37 (M) + Y39 (F), BS: 90–96.2; Figs 1, 2, Suppl. material 4, 5) were recovered as distinct clusters, but their taxonomic status cannot be resolved using COI alone. These results are consistent with the long-recognized taxonomic complexity of the genus *Rhyncomya* and point to the need for denser sampling and the analysis of multilocus molecular data. Beyond highlighting existing taxonomic problems, DNA barcoding also revealed potential undescribed diversity in several genera of the Afrotropical region. Five unnamed lineages of *Cosmina* from Kenya and Madagascar were recovered as distinct barcode clusters congruent with morphological differentiation; an additional lineage was detected within *Stegosoma*, two within *Isomyia*, and five within *Rhyncomya*. While COI data alone cannot justify formal taxonomic actions, these findings identify priority targets for future integrative studies.

Another important contribution of this study was the successful association of females and males for 31 morphospecies (= *Cosmina* [2], *Eurhyncomyia* [1], *Fainia* [3], *Isomyia* [5], *Rhinia* [1?], *Rhyncomya* [8], *Stegosoma* [2], *Stomorhina* [7], and *Thoracites* [2]; Figs 1, 2, Suppl. material 4, 5), addressing a long-standing challenge in Rhiniinae taxonomy. Indeed, DNA barcoding is widely known as a powerful tool to link female and male morphotypes (Ekrem et al. 2010; Yusseff-Vanegas and Agnarsson 2017; Li et al. 2019; Grzywacz et al. 2021; Szpila et al. 2025), especially in groups where the morphological identification of species relies heavily on male terminalia, and where female-specific characters are poorly represented in the identification keys (Peris 1952b; Zumpt 1958; Kurahashi and Kirk-Spriggs 2006), as is the case for Rhiniinae. Our findings will substantially contribute to the descriptions and redescriptions of females, and to the improvement of taxonomic identification tools for nose flies in the Afrotropical fauna.

Our results recovered 79 putative species out of ~150 Afrotropical species representing approximately half the fauna currently known from the region. We generated COI DNA barcodes for four of the 19 described species of *Cosmina*, nine of the 46 described species of *Isomyia*, 11 of the 55 described species of *Rhyncomya*, and eight of the 16 described species of *Stomorhina* (Rognes et al. in press). Future research should prioritise these four genera, which are among the most species-rich in the Afrotropics and remain taxonomically challenging, particularly in associating female and male morphotypes, and the continued discovery of new taxa within these groups, as evidenced in our study. While COI barcoding provides a solid initial method for identifying Rhiniinae, our findings emphasize the importance of integrating morphology with additional molecular markers when barcode results are unclear. Employing multilocus strategies that include mitochondrial cytochrome b (CytB) and nuclear markers such as internal transcribed spacer 2 (ITS2) and 28S ribosomal RNA (28S rRNA) has already shown effectiveness in species delimitation and identification within Calliphoridae and other Calyptratae flies (Oliveira et al. 2011; Yusseff-Vanegas and Agnarsson 2017; Madeira-Ott et al. 2019; Yang et al. 2022). The growing accessibility of next-generation sequencing techniques presents promising opportunities to resolve complex species groups, study introgression and hybridization, and produce multilocus data from both fresh and preserved specimens (Mayer et al. 2021; Call et al. 2023), offering a valuable resource for future Rhiniinae research.

### Rhiniinae sequences in BOLD Systems and GenBank, and curated COI DNA barcode library for Afrotropical nose flies

This study offers the most comprehensive COI DNA barcode dataset for Rhiniinae to date, emphasizing the Afrotropical fauna, and introduces the first quality-controlled reference library for the subfamily. The effectiveness of DNA barcoding depends heavily on the quality of its reference database, and precise identification requires DNA barcodes from morphologically confirmed voucher specimens, ideally sampled to reflect each species’ geographic range (Yusseff-Vanegas and Agnarsson 2017). The curated reference library presented here substantially improves the molecular coverage for Rhiniinae. We documented the broad geographic distribution of several species (e.g., *Rhyncomya cassotis*, *Rh. soyauxi*, and *Stomorhina lunata*) across the Afrotropical region, and added ~36 new named species to existing public repositories. Nevertheless, significant gaps remain in taxonomy and geography.

Public reference databases such as BOLD Systems and GenBank play a central role in DNA barcoding studies by providing the comparative framework for sequence identification (Ratnasingham and Hebert 2007; Benson et al. 2013). Recent studies have shown that misidentifications, incomplete metadata, and uneven reference coverage in public databases can substantially reduce identification reliability and distort barcode clustering patterns (Meiklejohn et al. 2019; Pentinsaari et al. 2020; Meier et al. 2022; Phillips et al. 2022). Conversely, large-scale barcode initiatives have shown that, when reference libraries are supported by dense sampling and curated voucher material, DNA barcodes can provide highly effective identification frameworks (Hebert et al. 2016). DNA barcode data of Rhiniinae in public repositories lacked species- and genus-level identifications for the majority of the sequences, likely reflecting both the intrinsic identification difficulty of the group and the scarcity of active taxonomic expertise.

Of the 60 Rhiniinae sequences obtained from GenBank, those previously identified only to subfamily or genus level could be assigned to specific species or genus names when examined in a voucher-based context. Nonetheless, a few sequences did not cluster with verified congeners (e.g., *Idiella divisa* and *Isomyia quadrina*), which prevented confident reclassification and highlights that ambiguous or potentially misidentified entries persist in public databases. The case of BOLD Systems highlights current strengths and weaknesses of the global reference library. Of the analyzed 1,112 COI DNA barcode sequences, 957 BINs initially lacked species- or genus-level names. By integrating ML clustering, ABGD, and ASAP partitions with morphological inspection of voucher images, 749 BINs previously not linked to any species name could be assigned to 11 described species. Additionally, 193 BINs representing 24 morphospecies were assigned to a genus. This finding shows that a large portion of publicly available RhiniinaeCOI DNA barcodes can be taxonomically identified through analysis within a curated, morphology-based framework. A small subset of BINs (15) could not be confidently assigned to any morphologically verified species in our dataset, and a few were found to represent non-Rhiniinae or to have incorrect generic placements. This highlights that, despite their indispensable role, public COI DNA barcode databases contain taxonomic inaccuracies and insufficiently documented records that may compromise automated identification procedures if not critically evaluated.

This study not only enhances species-level resolution but also develops a voucher-based reference framework for assessing records of Rhiniinae in BOLD Systems and GenBank. The long-term reliability of DNA barcode libraries for Afrotropical Rhiniinae depends on expanding sequence data, maintaining taxonomic expertise, thorough voucher documentation, and ongoing re-evaluation of public records. By combining molecular evidence with morphological analysis and a comprehensive database review, this work aims to improve the accuracy and stability of reference systems for this taxonomically complex group.

## Conclusions

This study provides the first comprehensive DNA barcode reference library for Afrotropical Rhiniinae, integrating 216 newly generated COI sequences with more than 1,150 sequences from public databases. By combining molecular and morphological data, we recognized 79 putative species for the Afrotropical region and confirmed female-male associations for 31 morphotypes.

At the species level, most morphologically defined species were also supported by our molecular analyses, showing high congruence between datasets. DNA barcoding proved effective for species identification and for revealing cryptic diversity inside the subfamily. Several lineages, including *Fainia*, *Rhinia*, *Cosmina*, *Stomorhina*, and some *Rhyncomya* groups, showed low COI divergence, suggesting recent diversification, intraspecific polymorphism, or overlooked species complexes. To resolve these cases and test monophyly in groups with shallow COI divergences, multilocus approaches involving ITS2, CytB, and 28S are recommended.

Overall, this study advances the molecular taxonomy of Rhiniinae, clarifies species boundaries, and assigns putative species names to numerous public sequences held in BOLD and GenBank. By providing a quality-assured DNA barcode reference library, it establishes an essential framework for future ecological, evolutionary, and systematic research of this taxonomically challenging group of blow flies.

## Additional information

### Artificial Intelligence (AI) use

Regarding the use of AI in the preparation of this manuscript, the authors declare the following:

Grammarly was used to check the English language grammar.

### Author ORCIDs

A. Thomas-Cabianca https://orcid.org/0000-0003-2126-6222

K. Jordaens https://orcid.org/0000-0003-4321-5944

E. Buenaventura https://orcid.org/0000-0002-5265-815X

A. Rytter-Jensen https://orcid.org/0000-0002-3056-7273

G. Goergen https://orcid.org/0000-0003-4496-0495

S. Rojo https://orcid.org/0000-0003-2160-9643

P. Cerretti https://orcid.org/0000-0002-9204-3352

T. Pape https://orcid.org/0000-0001-6609-0609

### Data availability

All of the data that support the findings of this study are available in the main text or Supplementary Information.

## Supplementary materials

Supplementary material 1 COI amplification primers, PCR reaction mix, and amplification thermal cycling protocol Data type docx Explanation note **G**: group of samples for DNA extraction (**1**: EB, **2**: ATC, **3**: KJ); **Sequences**: **F**: forward; **R**: reverse; **PCR reaction**: **T DNA**: template DNA; **GRG MM**: GoTaq Green Master Mix (Promega); **HFB MM**: HOT FIREPol Blend Master Mix (Solis BioDyne); **TP**: Taq polymerase; **H_2_O**: distilled and autoclaved water; **TV**: total Volumen; **Amplification thermal cycling: ID**: initial denaturation; **C**: cycles; **D**: denaturation; **AN**: annealing; **E**: extension; **FE**: final extension. https://binary.pensoft.net/file/1707714This dataset is made available under the Open Database License (http://opendatacommons.org/licenses/odbl/1.0/). The Open Database License (ODbL) is a license agreement intended to allow users to freely share, modify, and use this Dataset while maintaining this same freedom for others, provided that the original source and author(s) are credited. Arianna Thomas-Cabianca, Kurt Jordaens, Eliana Buenaventura, Arn Rytter-Jensen, Anabel Martínez-Sánchez, Georg Goergen, Santos Rojo, Pierfilippo Cerretti, Thomas Pape

Supplementary material 2 COI DNA barcode sequences downloaded from the GenBank Data type docx Explanation note COI DNA barcode sequences downloaded from the GenBank (https://www.ncbi.nlm.nih.gov/genbank/) on 20 November 2023. Sequences were obtained via BLAST searches (program megablast) using *Stomorhina lunata* (MN868825) as a query, filtered for “Rhiniinae”. Only sequences >500 bp were retained. Scientific Name: scientific name provided for the sequences in the Genbank; Accession: unique, persistent identifier assigned to the sequence; Species code: specimen identifiers (in analyses and trees (Figs 1, 2, 3). https://binary.pensoft.net/file/1707715This dataset is made available under the Open Database License (http://opendatacommons.org/licenses/odbl/1.0/). The Open Database License (ODbL) is a license agreement intended to allow users to freely share, modify, and use this Dataset while maintaining this same freedom for others, provided that the original source and author(s) are credited. Arianna Thomas-Cabianca, Kurt Jordaens, Eliana Buenaventura, Arn Rytter-Jensen, Anabel Martínez-Sánchez, Georg Goergen, Santos Rojo, Pierfilippo Cerretti, Thomas Pape

Supplementary material 3 COI DNA barcode sequences downloaded from the BOLD Systems Data type docx Explanation note COI DNA barcode sequences downloaded from the BOLD Systems (https://portal.boldsystems.org/) on 21 November 2023). Sequences were downloaded using the “Calliphoridae-Rhiniinae” filter. Only sequences >500 bp were retained. Scientific Name: scientific name provided for the sequences in the BOLD Systems; id: unique, persistent identifier assigned to the sequence in the BOLD Systems; Species code: specimen identifiers (in analyses and trees (Figs 1, 2, 3). https://binary.pensoft.net/file/1707716This dataset is made available under the Open Database License (http://opendatacommons.org/licenses/odbl/1.0/). The Open Database License (ODbL) is a license agreement intended to allow users to freely share, modify, and use this Dataset while maintaining this same freedom for others, provided that the original source and author(s) are credited. Arianna Thomas-Cabianca, Kurt Jordaens, Eliana Buenaventura, Arn Rytter-Jensen, Anabel Martínez-Sánchez, Georg Goergen, Santos Rojo, Pierfilippo Cerretti, Thomas Pape

Supplementary material 4 Unedited Neighbor-Joining (NJ) tree inferred using the Kimura 2-parameter (K2P) model and a dataset of 216 COI DNA barcode sequences of Rhiniinae species from the Afrotropical region and four outgroups Data type svg Explanation note The species *Hylemya alcathoe* (Walker, 1849) (Anthomyiinae) was used as outgroup to root the tree. Numbers given above the branches indicate bootstrap (BS) support (500 replicates). https://binary.pensoft.net/file/1707717This dataset is made available under the Open Database License (http://opendatacommons.org/licenses/odbl/1.0/). The Open Database License (ODbL) is a license agreement intended to allow users to freely share, modify, and use this Dataset while maintaining this same freedom for others, provided that the original source and author(s) are credited. Arianna Thomas-Cabianca, Kurt Jordaens, Eliana Buenaventura, Arn Rytter-Jensen, Anabel Martínez-Sánchez, Georg Goergen, Santos Rojo, Pierfilippo Cerretti, Thomas Pape

Supplementary material 5 Unedited Maximum Likelihood (ML) tree inferred using the GTR+I+G codon-position partitioning model and a dataset of 216 COI DNA barcode sequences of Rhiniinae species from the Afrotropical region and four outgroups Data type svg Explanation note The species *Hylemya alcathoe* (Walker, 1849) (Anthomyiinae) was used as outgroup to root the tree. Numbers above the branches indicate bootstrap (BS) support (100 replicates). https://binary.pensoft.net/file/1707718This dataset is made available under the Open Database License (http://opendatacommons.org/licenses/odbl/1.0/). The Open Database License (ODbL) is a license agreement intended to allow users to freely share, modify, and use this Dataset while maintaining this same freedom for others, provided that the original source and author(s) are credited. Arianna Thomas-Cabianca, Kurt Jordaens, Eliana Buenaventura, Arn Rytter-Jensen, Anabel Martínez-Sánchez, Georg Goergen, Santos Rojo, Pierfilippo Cerretti, Thomas Pape

Supplementary material 6 Unedited Maximum Likelihood (ML) tree inferred using the GTR+I+G codon-position partitioning model and a dataset of 1,388 COI DNA barcode sequences of Rhiniinae species and four outgroups. Data type svg Explanation note The species *Hylemya alcathoe* (Walker, 1849) (Anthomyiinae) was used as outgroup to root the tree. Numbers above the branches indicate bootstrap (BS) support (100 replicates). https://binary.pensoft.net/file/1707719This dataset is made available under the Open Database License (http://opendatacommons.org/licenses/odbl/1.0/). The Open Database License (ODbL) is a license agreement intended to allow users to freely share, modify, and use this Dataset while maintaining this same freedom for others, provided that the original source and author(s) are credited. Arianna Thomas-Cabianca, Kurt Jordaens, Eliana Buenaventura, Arn Rytter-Jensen, Anabel Martínez-Sánchez, Georg Goergen, Santos Rojo, Pierfilippo Cerretti, Thomas Pape

Supplementary material 7 Results of the partitions for the ABGD and ASAP analyses of the COI DNA barcode sequences Data type xlsx Explanation note Results of the partitions for the ABGD and ASAP analyses of the COI DNA barcode sequences of the Rhiniinae sequences studied (216 newly generated DNA barcode sequences for Afrotropical fauna and 1,172 DNA barcode sequences downloaded from GenBank and the BOLD Systems platforms accessed in November 2023). https://binary.pensoft.net/file/1707720This dataset is made available under the Open Database License (http://opendatacommons.org/licenses/odbl/1.0/). The Open Database License (ODbL) is a license agreement intended to allow users to freely share, modify, and use this Dataset while maintaining this same freedom for others, provided that the original source and author(s) are credited. Arianna Thomas-Cabianca, Kurt Jordaens, Eliana Buenaventura, Arn Rytter-Jensen, Anabel Martínez-Sánchez, Georg Goergen, Santos Rojo, Pierfilippo Cerretti, Thomas Pape

Supplementary material 8 Results of ASAP with Distance K80_Kimura and ABGD with Distance K80 Kimura Data type docx Explanation note Results of ASAP with Distance K80_Kimura and ABGD with Distance K80 Kimura / MinSlope = 0.900000 for a dataset of 216 COI DNA barcode sequences of Rhiniinae species from the Afrotropical region. For the ABGD analysis (Model = K80), partitions five and six proposed 59 species groups (p = 0.00774–0.0129, BG = 0.019%), and partition four proposed 78 species groups (p = 0.00464, BG = 0.007%); and the ASAP analysis (Model = K80; ASAP score = 2.50) resulted in 62 species groups. https://binary.pensoft.net/file/1707721This dataset is made available under the Open Database License (http://opendatacommons.org/licenses/odbl/1.0/). The Open Database License (ODbL) is a license agreement intended to allow users to freely share, modify, and use this Dataset while maintaining this same freedom for others, provided that the original source and author(s) are credited. Arianna Thomas-Cabianca, Kurt Jordaens, Eliana Buenaventura, Arn Rytter-Jensen, Anabel Martínez-Sánchez, Georg Goergen, Santos Rojo, Pierfilippo Cerretti, Thomas Pape

Supplementary material 9 Interspecific K2P-genetic distances between a dataset of 216 COI DNA barcode sequences of Rhiniinae species from the Afrotropical region Data type xlsx https://binary.pensoft.net/file/1707722This dataset is made available under the Open Database License (http://opendatacommons.org/licenses/odbl/1.0/). The Open Database License (ODbL) is a license agreement intended to allow users to freely share, modify, and use this Dataset while maintaining this same freedom for others, provided that the original source and author(s) are credited. Arianna Thomas-Cabianca, Kurt Jordaens, Eliana Buenaventura, Arn Rytter-Jensen, Anabel Martínez-Sánchez, Georg Goergen, Santos Rojo, Pierfilippo Cerretti, Thomas Pape

Supplementary material 10 Results of ASAP with Distance K80_Kimura and ABGD with Distance K80 Kimura Data type docx Explanation note Results of ASAP with Distance K80_Kimura and ABGD with Distance K80 Kimura / MinSlope = 0.900000 for a dataset of 1,388 COI DNA barcode sequences of Rhiniinae sequences studied, downloaded from GenBank and the BOLD Systems platforms (accessed in November 2023). For the ABGD analysis (Model = K80), partition five proposed 109 species groups (p = 0.007743, BG = 0.018%), partition six proposed 94 species groups (p = 0.012915, BG = 0.026%), and partition seven proposed 89 species groups (p = 0.021544, BG = 0.031%); and the ASAP analysis (Model = K80; ASAP score = 2.50) proposed 92 species groups. https://binary.pensoft.net/file/1707723This dataset is made available under the Open Database License (http://opendatacommons.org/licenses/odbl/1.0/). The Open Database License (ODbL) is a license agreement intended to allow users to freely share, modify, and use this Dataset while maintaining this same freedom for others, provided that the original source and author(s) are credited. Arianna Thomas-Cabianca, Kurt Jordaens, Eliana Buenaventura, Arn Rytter-Jensen, Anabel Martínez-Sánchez, Georg Goergen, Santos Rojo, Pierfilippo Cerretti, Thomas Pape

Supplementary material 11 Alignment #1 for a dataset of 216 newly generated COI DNA barcode sequences of Rhiniinae species and four outgroups Data type COI sequences alignment Explanation note Sequences were edited and assembled using De Novo Assembly with ‘High Sensitivity/Medium’ parameters in Geneious v. 9.0.5 (Biomatters Ltd.) (Kearse et al. 2012), with ends trimmed at an error probability limit of 0.05. Alignments were generated using MUSCLE in MEGA-X v. 10.0.5 (Kumar et al. 2018) and translated to amino acids for error checking. https://binary.pensoft.net/file/1707724This dataset is made available under the Open Database License (http://opendatacommons.org/licenses/odbl/1.0/). The Open Database License (ODbL) is a license agreement intended to allow users to freely share, modify, and use this Dataset while maintaining this same freedom for others, provided that the original source and author(s) are credited. Arianna Thomas-Cabianca, Kurt Jordaens, Eliana Buenaventura, Arn Rytter-Jensen, Anabel Martínez-Sánchez, Georg Goergen, Santos Rojo, Pierfilippo Cerretti, Thomas Pape

Supplementary material 12 Alignment #2 for a dataset of 1,388 COI DNA barcode sequences of Rhiniinae species studied and four outgroups Data type COI sequences alignment Explanation note Alignment #2 for a dataset of 1,388 COI DNA barcode sequences of Rhiniinae species studied and four outgroups (216 newly generated DNA barcode sequences for Afrotropical fauna and 1,172 DNA barcode sequences downloaded from GenBank and the BOLD Systems platforms accessed in November 2023). Sequences were edited and assembled using De Novo Assembly with ‘High Sensitivity/Medium’ parameters in Geneious v. 9.0.5 (Biomatters Ltd.) (Kearse et al. 2012), with ends trimmed at an error probability limit of 0.05. Alignments were generated using MUSCLE in MEGA-X v. 10.0.5 (Kumar et al. 2018) and translated to amino acids for error checking. https://binary.pensoft.net/file/1707725This dataset is made available under the Open Database License (http://opendatacommons.org/licenses/odbl/1.0/). The Open Database License (ODbL) is a license agreement intended to allow users to freely share, modify, and use this Dataset while maintaining this same freedom for others, provided that the original source and author(s) are credited. Arianna Thomas-Cabianca, Kurt Jordaens, Eliana Buenaventura, Arn Rytter-Jensen, Anabel Martínez-Sánchez, Georg Goergen, Santos Rojo, Pierfilippo Cerretti, Thomas Pape

Supplementary material 13 Grouping of the sequences of Rhiniinae studies Data type xlsx Explanation note Grouping of the sequences of Rhiniinae studies (216 newly generated DNA barcode sequences for Afrotropical fauna and 1,172 DNA barcode sequences downloaded from GenBank and the BOLD Systems platforms accessed in November 2023) and final identification of the BINs integrating ML, ABGD, ASAP, and morphological results. When images were available in the BOLD Systems repository, some of the BINs were given at least the genus name. https://binary.pensoft.net/file/1707726This dataset is made available under the Open Database License (http://opendatacommons.org/licenses/odbl/1.0/). The Open Database License (ODbL) is a license agreement intended to allow users to freely share, modify, and use this Dataset while maintaining this same freedom for others, provided that the original source and author(s) are credited. Arianna Thomas-Cabianca, Kurt Jordaens, Eliana Buenaventura, Arn Rytter-Jensen, Anabel Martínez-Sánchez, Georg Goergen, Santos Rojo, Pierfilippo Cerretti, Thomas Pape
