# Supplementary material for: DNA barcoding of Afrotropical nose flies (Diptera, Calliphoridae, Rhiniinae): species identification, female-male morphotype association, and reference library development
Source: Zookeys. 2026 Jul 3;1284:149–83. doi: 10.3897/zookeys.1284.189450 (PMC13354976; doi:10.3897/zookeys.1284.189450)
Supplement: Supplementary material 1 — COI amplification primers, PCR reaction mix, and amplification thermal cycling protocol [file zookeys-1284-149_article-189450__-s001.docx]

**Suppl. Material 1** – COI amplification primers, PCR reaction mix, and amplification thermal cycling protocol. **G:** group of samples for DNA extraction (**1**: EB, **2**: ATC, **3**: KJ); **Sequences**: **F**: forward; **R**: reverse; **PCR reaction**: **T DNA**: template DNA; **GRG MM**: GoTaq Green Master Mix (Promega); **HFB MM**: HOT FIREPol Blend Master Mix (Solis BioDyne); **TP**: Taq polymerase; **H_2_O**: distilled and autoclaved water; **TV**: total Volumen; **Amplification thermal cycling: ID**: initial denaturation; **C**: cycles; **D**: denaturation; **AN**: annealing; **E**: extension; **FE**: final extension.

| **G** | **Primer name** | **Sequences 5'-3'** | **PCR reaction** | **Amplification thermal cycling** | **Reference** |
| --- | --- | --- | --- | --- | --- |
| **1** | TY-J-1460 | F  TACAATYTATCGCCTAAACTTCAGCC | T DNA = 1 μL  GRG MM = 7.5 μL  Each primer = 0.4μL  H_2_O = 5.7 μL  TV = 15 μL | ID = 95 °C 2 min  C = 29  D = 94 °C 30 s  AN = 47.5 ºC 30 s  E =72 °C 120 s  FE = 72 °C 8 min | Adjusted from Simon et al. 1994 and Bernasconi et al. 2000 |
|  | C1-N-2191 | R  CCCGGTAAAATTAAAATATAAACTTC |  |  |  |
| **2** | TY-J-1460 | F  TACAATYTATCGCCTAAACTTCAGCC | T DNA = 4 μL  HFB MM = 4 μL  Each primer = 0.5μL  H_2_O = 16 μL  TV = 25 μL | ID = 95 °C 5 min  C = 40-50  D = 95 °C 60 s  AN = 45 ºC 60 s  E = 72 °C 120 s  FE = 72 °C 10 min | Simon et al. 1994 and Bernasconi et al. 2000 |
|  | C1-N-2191 | R  CCCGGTAAAATTAAAATATAAACTTC |  |  |  |
| **3** | LCO1490 | F GGTCAACAAATCATAAAGATATTGG | T DNA = μL  TP = 0.5 U  MgCl₂ = 1.5 mM  PCR buffer = 1x  Each dNTP = 0.2 mM  Each primer = 0.2 μL  H_2_O = XX μL  TV = 25 μL | ID = 95 °C 5 min  C = 35  D = 95 °C 45 s  AN = 45 ºC 45 s  E = 72 °C 90 s  FE = 72 °C 5 min | Folmer et al. 1994 |
|  | HCO2198 | R TAAACTTCAGGGTGACCAAAAAATCA |  |  |  |
