# Supplementary material for: DNA barcoding of Afrotropical nose flies (Diptera, Calliphoridae, Rhiniinae): species identification, female-male morphotype association, and reference library development
Source: Zookeys. 2026 Jul 3;1284:149–83. doi: 10.3897/zookeys.1284.189450 (PMC13354976; doi:10.3897/zookeys.1284.189450)
Supplement: Supplementary material 2 — COI DNA barcode sequences downloaded from the GenBank [file zookeys-1284-149_article-189450__-s002.docx]

**Suppl. Material 2** – COI DNA barcode sequences downloaded from the GenBank (<https://www.ncbi.nlm.nih.gov/genbank/>) on 20 November 2023. Sequences were obtained via BLAST searches (program megablast) using *Stomorhina lunata* (MN868825) as a query, filtered for “Rhiniinae”. Only sequences >500 bp were retained. **Scientific Name**: scientific name provided for the sequences in the Genbank; **Accession**: unique, persistent identifier assigned to the sequence; **Species code**: specimen identifiers (in analyses and trees (Figures 1–3).

| **Scientific Name** | **Accession** | **Species code** |
| --- | --- | --- |
| *Metallea erinacea* | GQ409337.1 | GQ409337.1_Metallea_erinacea |
| *Rhyncomya nigripes* | GQ409356.1 | GQ409356.1_Rhyncomya_nigripes |
| *Stomorhina discolor* | GQ409375.1 | GQ409375.1_Stomorhina_discolor |
| Rhiniidae sp | GU681898.1 | GU681898.1_Rhiniidae_sp |
| *Rhinia apicalis* | HM375982.1 | HM375982.1_Rhinia_apicalis |
| *Stomorhina pollinosa* | HM399341.1 | HM399341.1_Stomorhina_pollinosa |
| *Metallea incisuralis* | HM399342.1 | HM399342.1_Metallea_incisuralis |
| *Metallea incisuralis* | HM399345.1 | HM399345.1_Metallea_incisuralis |
| *Metallea incisuralis* | HM399346.1 | HM399346.1_Metallea_incisuralis |
| *Isomyia pseudonepalana* | HQ561046.1 | HQ561046.1_Isomyia_pseudonepalana |
| *Stomorhina discolor* | HQ561056.1 | HQ561056.1_Stomorhina_discolor |
| *Isomyia gomezmenor* | JF439553.1 | JF439553.1_Isomyia_gomezmenor |
| *Cosmina fuscipennis* | JQ246691.1 | JQ246691.1_Cosmina_fuscipennis |
| *Rhinia* sp | JQ246692.1 | JQ246692.1_Rhinia_sp |
| *Rhyncomya soyauxi* | JQ246693.1 | JQ246693.1_Rhyncomya_soyauxi |
| *Thoracites* sp | JQ246694.1 | JQ246694.1_Thoracites_sp |
| *Stomorhina lunata* | KP004766.1 | KP004766.1_Stomorhina_lunata |
| Rhiniidae sp | KX054612.1 | KX054612.1_Rhiniidae_sp |
| Rhiniidae sp | KX054613.1 | KX054613.1_Rhiniidae_sp |
| Rhiniidae sp | KX054615.1 | KX054615.1_Rhiniidae_sp |
| *Isomyia electa* | KY031766.1 | KY031766.1_Isomyia_electa |
| *Isomyia electa* | KY031767.1 | KY031767.1_Isomyia_electa |
| *Isomyia electa* | KY031768.1 | KY031768.1_Isomyia_electa |
| *Isomyia pseudolucilia* | KY031769.1 | KY031769.1_Isomyia_pseudolucilia |
| *Isomyia pseudonepalana* | KY031771.1 | KY031771.1_Isomyia_pseudonepalana |
| *Isomyia complantenna* | KY031773.1 | KY031773.1_Isomyia_complantenna |
| *Isomyia verirecta* | KY031774.1 | KY031774.1_Isomyia_verirecta |
| *Stomorhina discolor* | KY031819.1 | KY031819.1_Stomorhina_discolor |
| *Stomorhina discolor* | KY031820.1 | KY031820.1_Stomorhina_discolor |
| *Stomorhina obsoleta* | KY031821.1 | KY031821.1_Stomorhina_obsoleta |
| *Stomorhina lunata* | KY749786.1 | KY749786.1_Stomorhina_lunata |
| Rhiniidae sp | KY835753.1 | KY835753.1_Rhiniidae_sp |
| Rhiniidae sp | KY837821.1 | KY837821.1_Rhiniidae_sp |
| Rhiniidae sp | KY838746.1 | KY838746.1_Rhiniidae_sp |
| Rhiniidae sp | KY838995.1 | KY838995.1_Rhiniidae_sp |
| Rhiniidae sp | KY841564.1 | KY841564.1_Rhiniidae_sp |
| *Stomorhina discolor* | KY842007.1 | KY842007.1_Stomorhina_discolor |
| *Stomorhina discolor* | KY844929.1 | KY844929.1_Stomorhina_discolor |
| *Stomorhina obsoleta* | LC477291.1 | LC477291.1_Stomorhina_obsoleta |
| *Borbororhinia bivittata* | LC549079.1 | LC549079.1_Borbororhinia_bivittata |
| *Borbororhinia bivittata* | LC549094.1 | LC549094.1_Borbororhinia_bivittata |
| *Stomorhina obsoleta* | LC682331.1 | LC682331.1_Stomorhina_obsoleta |
| *Isomyia* sp | MF804688.1 | MF804688.1_Isomyia_sp_ |
| *Alikangiella rufithorax* | MG967831.1 | MG967831.1_Alikangiella_rufithorax |
| *Strongyloneura prolata* | MG967868.1 | MG967868.1_Strongyloneura_prolata |
| *Thoracites abdominalis* | MG968089.1 | MG968089.1_Thoracites_abdominalis |
| *Stegosoma vinculatum* | MG968160.1 | MG968160.1_Stegosoma_vinculatum |
| *Borbororhinia bivittata* | MN411061.1 | MN411061.1_Borbororhinia_bivittata |
| *Fainia albitarsis* | MN411166.1 | MN411166.1_Fainia_albitarsis |
| *Rhyncomya seguyi* | MN411167.1 | MN411167.1_Rhyncomya_seguyi |
| *Isomyia dotata* | MN411223.1 | MN411223.1_Isomyia_dotata |
| *Eurhyncomyia diversicolor* | MN411268.1 | MN411268.1_Eurhyncomyia_diversicolor |
| *Stomorhina lunata* | MN868811.1 | MN868811.1_Stomorhina_lunata |
| *Rhyncomya columbina* | MN868846.1 | MN868846.1_Rhyncomya_columbina |
| *Stomorhina obsoleta* | OL343410.1 | OL343410.1_Stomorhina_o |
| *Stomorhina obsoleta* | OL343411.1 | OL343411.1_Stomorhina_obsoleta |
| *Stomorhina obsoleta* | OL343412.1 | OL343412.1_Stomorhina_obsoleta |
| *Stomorhina discolor* | OP268186.1 | OP268186.1_Stomorhina_discolor |
| *Isomyia nebulosa* | OR497843.1 | OR497843.1_Isomyia_nebulosa |
| *Stomorhina lunata* | OW121746.1 | OW121746.1_Stomorhina_lunata |
