# Supplementary material for: DNA barcoding of Afrotropical nose flies (Diptera, Calliphoridae, Rhiniinae): species identification, female-male morphotype association, and reference library development
Source: Zookeys. 2026 Jul 3;1284:149–83. doi: 10.3897/zookeys.1284.189450 (PMC13354976; doi:10.3897/zookeys.1284.189450)
Supplement: Supplementary material 3 — COI DNA barcode sequences downloaded from the BOLD Systems [file zookeys-1284-149_article-189450__-s003.docx]

**Suppl. Material 3** – COI DNA barcode sequences downloaded from the BOLD Systems (<https://portal.boldsystems.org/>) on 21 November 2023). Sequences were downloaded using the “Calliphoridae-Rhiniinae” filter. Only sequences >500 bp were retained. **Scientific Name**: scientific name provided for the sequences in the BOLD Systems; **id**: unique, persistent identifier assigned to the sequence in the BOLD Systems; **Species code**: specimen identifiers (in analyses and trees (Figures 1–3).

| **Scientific Name** | **Id** | **Species code** |
| --- | --- | --- |
| *Stomorhina* | ASMII1048-22 | ASMII1048-22_ Stomorhina |
| Rhiniinae | ASMII10886-22 | ASMII10886-22_ Rhiniinae |
| Rhiniinae | ASMII10905-22 | ASMII10905-22_ Rhiniinae |
| Rhiniinae | ASMII1109-22 | ASMII1109-22_ Rhiniinae |
| Rhiniinae | ASMII1110-22 | ASMII1110-22_ Rhiniinae |
| Rhiniinae | ASMII1114-22 | ASMII1114-22_ Rhiniinae |
| Rhiniinae | ASMII1136-22 | ASMII1136-22_ Rhiniinae |
| Rhiniinae | ASMII1138-22 | ASMII1138-22_ Rhiniinae |
| Rhiniinae | ASMII1139-22 | ASMII1139-22_ Rhiniinae |
| Rhiniinae | ASMII1152-22 | ASMII1152-22_ Rhiniinae |
| *Stomorhina* | ASMII1170-22 | ASMII1170-22_ Stomorhina |
| *Stomorhina* | ASMII1171-22 | ASMII1171-22_ Stomorhina |
| Rhiniinae | ASMII1192-22 | ASMII1192-22_ Rhiniinae |
| *Stomorhina* | ASMII1195-22 | ASMII1195-22_ Stomorhina |
| Rhiniinae | ASMII12149-22 | ASMII12149-22_ Rhiniinae |
| *Stomorhina discolor* | ASMII12153-22 | ASMII12153-22_ Stomorhina_discolor |
| Rhiniinae | ASMII1352-22 | ASMII1352-22_ Rhiniinae |
| Rhiniinae | ASMII2368-22 | ASMII2368-22_ Rhiniinae |
| Rhiniinae | ASMII2442-22 | ASMII2442-22_ Rhiniinae |
| Rhiniinae | ASMII2448-22 | ASMII2448-22_ Rhiniinae |
| Rhiniinae | ASMII2465-22 | ASMII2465-22_ Rhiniinae |
| Rhiniinae | ASMII3354-22 | ASMII3354-22_ Rhiniinae |
| Rhiniinae | ASMII3356-22 | ASMII3356-22_ Rhiniinae |
| Rhiniinae | ASMII3388-22 | ASMII3388-22_ Rhiniinae |
| Rhiniinae | ASMII3391-22 | ASMII3391-22_ Rhiniinae |
| Rhiniinae | ASMII3436-22 | ASMII3436-22_ Rhiniinae |
| Rhiniinae | ASMII3439-22 | ASMII3439-22_ Rhiniinae |
| Rhiniinae | ASMII3510-22 | ASMII3510-22_ Rhiniinae |
| Rhiniinae | ASMII4719-22 | ASMII4719-22_ Rhiniinae |
| Rhiniinae | ASMII4720-22 | ASMII4720-22_ Rhiniinae |
| Rhiniinae | ASMII4747-22 | ASMII4747-22_ Rhiniinae |
| Rhiniinae | ASMII4749-22 | ASMII4749-22_ Rhiniinae |
| Rhiniinae | ASMII4765-22 | ASMII4765-22_ Rhiniinae |
| *Stomorhina discolor* | ASMII5628-22 | ASMII5628-22_ Stomorhina_discolor |
| Rhiniinae | ASMII6818-22 | ASMII6818-22_ Rhiniinae |
| Rhiniinae | ASMII6865-22 | ASMII6865-22_ Rhiniinae |
| Rhiniinae | ASMII7251-22 | ASMII7251-22_ Rhiniinae |
| Rhiniinae | ASMII7252-22 | ASMII7252-22_ Rhiniinae |
| Rhiniinae | ASMII7268-22 | ASMII7268-22_ Rhiniinae |
| Rhiniinae | ASMII7289-22 | ASMII7289-22_ Rhiniinae |
| Rhiniinae | ASMII7310-22 | ASMII7310-22_ Rhiniinae |
| *Stomorhina* | ASMII7478-22 | ASMII7478-22_ Stomorhina |
| *Stomorhina* | ASMII8543-22 | ASMII8543-22_ Stomorhina |
| *Stomorhina* | ASMII8544-22 | ASMII8544-22_ Stomorhina |
| *Stomorhina discolor* | ASMII9570-22 | ASMII9570-22_ Stomorhina_discolor |
| *Rhinia* | AUSBC1389-12 | AUSBC1389-12_ Rhinia |
| *Rhinia* | AUSBC1393-12 | AUSBC1393-12_ Rhinia |
| Rhiniinae | AUSMG383-20 | AUSMG383-20_ Rhiniinae |
| Rhiniinae | AUSMG385-20 | AUSMG385-20_ Rhiniinae |
| Rhiniinae | AUSMG386-20 | AUSMG386-20_ Rhiniinae |
| *Stomorhina discolor* | DIQTB231-11 | DIQTB231-11_ Stomorhina_discolor |
| *Stomorhina discolor* | DIQTB232-11 | DIQTB232-11_ Stomorhina_discolor |
| *Stomorhina discolor* | DIQTB251-11 | DIQTB251-11_ Stomorhina_discolor |
| Rhiniinae | DIQTB444-12 | DIQTB444-12_ Rhiniinae |
| *Rhinia apicalis* | DIQTB510-12 | DIQTB510-12_ Rhinia_apicalis |
| *Rhinia apicalis* | DIQTB559-12 | DIQTB559-12_ Rhinia_apicalis |
| *Rhinia apicalis* | DIQTB560-12 | DIQTB560-12_ Rhinia_apicalis |
| *Stomorhina discolor* | DIQTB580-12 | DIQTB580-12_ Stomorhina_discolor |
| *Stomorhina* | DPAS1068-11 | DPAS1068-11_ Stomorhina |
| *Rhinia* | DPAST1393-12 | DPAST1393-12_ Rhinia |
| Rhiniinae | DPSAF028-12 | DPSAF028-12_ Rhiniinae |
| Rhiniinae | ETKD1032-13 | ETKD1032-13_ Rhiniinae |
| Rhiniinae | ETKD1044-13 | ETKD1044-13_ Rhiniinae |
| Rhiniinae | ETKD541-12 | ETKD541-12_ Rhiniinae |
| Rhiniinae | ETKD613-12 | ETKD613-12_ Rhiniinae |
| Rhiniinae | ETKD633-12 | ETKD633-12_ Rhiniinae |
| Rhiniinae | ETKD757-12 | ETKD757-12_ Rhiniinae |
| Rhiniinae | ETKD787-13 | ETKD787-13_ Rhiniinae |
| Rhiniinae | ETKD788-13 | ETKD788-13_ Rhiniinae |
| Rhiniinae | ETKD853-13 | ETKD853-13_ Rhiniinae |
| *Stomorhina* | GMAEA5279-22 | GMAEA5279-22_ Stomorhina |
| Rhiniinae | GMAMC010-15 | GMAMC010-15_ Rhiniinae |
| Rhiniinae | GMAMH009-15 | GMAMH009-15_ Rhiniinae |
| Rhiniinae | GMAMI025-15 | GMAMI025-15_ Rhiniinae |
| Rhiniinae | GMAMJ027-15 | GMAMJ027-15_ Rhiniinae |
| Rhiniinae | GMAMJ034-15 | GMAMJ034-15_ Rhiniinae |
| Rhiniinae | GMAMJ186-15 | GMAMJ186-15_ Rhiniinae |
| Rhiniinae | GMAML005-15 | GMAML005-15_ Rhiniinae |
| Rhiniinae | GMAML006-15 | GMAML006-15_ Rhiniinae |
| Rhiniinae | GMAMM349-15 | GMAMM349-15_ Rhiniinae |
| Rhiniinae | GMAMN526-15 | GMAMN526-15_ Rhiniinae |
| Rhiniinae | GMAMS685-15 | GMAMS685-15_ Rhiniinae |
| Rhiniinae | GMAMS688-15 | GMAMS688-15_ Rhiniinae |
| Rhiniinae | GMAMS689-15 | GMAMS689-15_ Rhiniinae |
| Rhiniinae | GMAMS735-15 | GMAMS735-15_ Rhiniinae |
| Rhiniinae | GMAMS750-15 | GMAMS750-15_ Rhiniinae |
| Rhiniinae | GMAMS766-15 | GMAMS766-15_ Rhiniinae |
| Rhiniinae | GMAMT1493-16 | GMAMT1493-16_ Rhiniinae |
| Rhiniinae | GMAMT1494-16 | GMAMT1494-16_ Rhiniinae |
| Rhiniinae | GMAMT1495-16 | GMAMT1495-16_ Rhiniinae |
| Rhiniinae | GMAMT1498-16 | GMAMT1498-16_ Rhiniinae |
| Rhiniinae | GMASF011-17 | GMASF011-17_ Rhiniinae |
| Rhiniinae | GMBCC1933-15 | GMBCC1933-15_ Rhiniinae |
| *Stomorhina discolor* | GMBCC1944-15 | GMBCC1944-15_ Stomorhina_discolor |
| Rhiniinae | GMBCC1947-15 | GMBCC1947-15_ Rhiniinae |
| *Stomorhina discolor* | GMBCC3273-15 | GMBCC3273-15_ Stomorhina_discolor |
| Rhiniinae | GMBCD069-15 | GMBCD069-15_ Rhiniinae |
| Rhiniinae | GMBCD1162-15 | GMBCD1162-15_ Rhiniinae |
| Rhiniinae | GMBCD1482-15 | GMBCD1482-15_ Rhiniinae |
| *Stomorhina discolor* | GMBCD1746-15 | GMBCD1746-15_ Stomorhina_discolor |
| Rhiniinae | GMBCD1761-15 | GMBCD1761-15_ Rhiniinae |
| Rhiniinae | GMBCD1894-15 | GMBCD1894-15_ Rhiniinae |
| Rhiniinae | GMBCD3143-15 | GMBCD3143-15_ Rhiniinae |
| Rhiniinae | GMBCD3155-15 | GMBCD3155-15_ Rhiniinae |
| Rhiniinae | GMBCE1745-15 | GMBCE1745-15_ Rhiniinae |
| Rhiniinae | GMBCE2167-15 | GMBCE2167-15_ Rhiniinae |
| Rhiniinae | GMBCE2722-15 | GMBCE2722-15_ Rhiniinae |
| Rhiniinae | GMBCE2931-15 | GMBCE2931-15_ Rhiniinae |
| Rhiniinae | GMBCE2938-15 | GMBCE2938-15_ Rhiniinae |
| Rhiniinae | GMBCE3416-15 | GMBCE3416-15_ Rhiniinae |
| Rhiniinae | GMBCE3750-15 | GMBCE3750-15_ Rhiniinae |
| Rhiniinae | GMBCF1172-15 | GMBCF1172-15_ Rhiniinae |
| Rhiniinae | GMBCF2805-15 | GMBCF2805-15_ Rhiniinae |
| Rhiniinae | GMBCF3180-15 | GMBCF3180-15_ Rhiniinae |
| Rhiniinae | GMBCF3679-15 | GMBCF3679-15_ Rhiniinae |
| Rhiniinae | GMBCF5409-15 | GMBCF5409-15_ Rhiniinae |
| Rhiniinae | GMBCF5874-15 | GMBCF5874-15_ Rhiniinae |
| Rhiniinae | GMBCH2736-15 | GMBCH2736-15_ Rhiniinae |
| *Stomorhina discolor* | GMBCI160-15 | GMBCI160-15_ Stomorhina_discolor |
| *Stomorhina discolor* | GMBCI1616-15 | GMBCI1616-15_ Stomorhina_discolor |
| *Stomorhina discolor* | GMBCI2361-15 | GMBCI2361-15_ Stomorhina_discolor |
| *Stomorhina discolor* | GMBCI2386-15 | GMBCI2386-15_ Stomorhina_discolor |
| Rhiniinae | GMBCI4695-15 | GMBCI4695-15_ Rhiniinae |
| *Stomorhina discolor* | GMBCM2533-15 | GMBCM2533-15_ Stomorhina_discolor |
| Rhiniinae | GMBCN631-15 | GMBCN631-15_ Rhiniinae |
| Rhiniinae | GMBCN894-15 | GMBCN894-15_ Rhiniinae |
| *Stomorhina discolor* | GMCHB388-14 | GMCHB388-14_ Stomorhina_discolor |
| *Stomorhina discolor* | GMCHE034-14 | GMCHE034-14_ Stomorhina_discolor |
| Rhiniinae | GMCWK043-15 | GMCWK043-15_ Rhiniinae |
| Rhiniinae | GMCWM164-15 | GMCWM164-15_ Rhiniinae |
| Rhiniinae | GMCWN700-15 | GMCWN700-15_ Rhiniinae |
| Rhiniinae | GMCWO139-15 | GMCWO139-15_ Rhiniinae |
| Rhiniinae | GMCWO140-15 | GMCWO140-15_ Rhiniinae |
| Rhiniinae | GMCWP067-15 | GMCWP067-15_ Rhiniinae |
| Rhiniinae | GMEGD012-14 | GMEGD012-14_ Rhiniinae |
| *Stomorhina lunata* | GMGMN1228-14 | GMGMN1228-14_ Stomorhina_lunata |
| Rhiniinae | GMIAE017-17 | GMIAE017-17_ Rhiniinae |
| Rhiniinae | GMIAG050-17 | GMIAG050-17_ Rhiniinae |
| Rhiniinae | GMIAJ036-17 | GMIAJ036-17_ Rhiniinae |
| *Stomorhina discolor* | GMIAK389-17 | GMIAK389-17_ Stomorhina_discolor |
| *Stomorhina discolor* | GMIAK420-17 | GMIAK420-17_ Stomorhina_discolor |
| *Stomorhina discolor* | GMIBA104-17 | GMIBA104-17_ Stomorhina_discolor |
| Rhiniinae | GMIBA251-17 | GMIBA251-17_ Rhiniinae |
| Rhiniinae | GMIBA257-17 | GMIBA257-17_ Rhiniinae |
| Rhiniinae | GMKMA558-15 | GMKMA558-15_ Rhiniinae |
| Rhiniinae | GMKMA582-15 | GMKMA582-15_ Rhiniinae |
| *Rhyncomya soyauxi* | GMKMA584-15 | GMKMA584-15_ Rhyncomya_soyauxi |
| Rhiniinae | GMKMA593-15 | GMKMA593-15_ Rhiniinae |
| Rhiniinae | GMKMB601-15 | GMKMB601-15_ Rhiniinae |
| Rhiniinae | GMKMB609-15 | GMKMB609-15_ Rhiniinae |
| *Rhyncomya soyauxi* | GMKMD027-15 | GMKMD027-15_ Rhyncomya_soyauxi |
| *Rhyncomya soyauxi* | GMKMD029-15 | GMKMD029-15_ Rhyncomya_soyauxi |
| *Rhyncomya soyauxi* | GMKME194-15 | GMKME194-15_ Rhyncomya_soyauxi |
| Rhiniinae | GMKME204-15 | GMKME204-15_ Rhiniinae |
| *Rhyncomya soyauxi* | GMKMF003-15 | GMKMF003-15_ Rhyncomya_soyauxi |
| Rhiniinae | GMKMF022-15 | GMKMF022-15_ Rhiniinae |
| Rhiniinae | GMKMG357-15 | GMKMG357-15_ Rhiniinae |
| Rhiniinae | GMKMH199-15 | GMKMH199-15_ Rhiniinae |
| Rhiniinae | GMKMI691-15 | GMKMI691-15_ Rhiniinae |
| Rhiniinae | GMKMJ917-15 | GMKMJ917-15_ Rhiniinae |
| Rhiniinae | GMKMJ926-15 | GMKMJ926-15_ Rhiniinae |
| *Rhyncomya soyauxi* | GMKMJ950-15 | GMKMJ950-15_ Rhyncomya_soyauxi |
| Rhiniinae | GMKML237-15 | GMKML237-15_ Rhiniinae |
| *Rhyncomya soyauxi* | GMKML243-15 | GMKML243-15_ Rhyncomya_soyauxi |
| Rhiniinae | GMKMN024-15 | GMKMN024-15_ Rhiniinae |
| Rhiniinae | GMKMN026-15 | GMKMN026-15_ Rhiniinae |
| Rhiniinae | GMKMN028-15 | GMKMN028-15_ Rhiniinae |
| *Rhyncomya soyauxi* | GMKMS030-15 | GMKMS030-15_ Rhyncomya_soyauxi |
| Rhiniinae | GMKMT173-15 | GMKMT173-15_ Rhiniinae |
| Rhiniinae | GMKMT188-15 | GMKMT188-15_ Rhiniinae |
| Rhiniinae | GMKMT195-15 | GMKMT195-15_ Rhiniinae |
| Rhiniinae | GMKMT200-15 | GMKMT200-15_ Rhiniinae |
| Rhiniinae | GMKMU108-15 | GMKMU108-15_ Rhiniinae |
| Rhiniinae | GMKMU731-15 | GMKMU731-15_ Rhiniinae |
| Rhiniinae | GMKMV452-15 | GMKMV452-15_ Rhiniinae |
| Rhiniinae | GMKMW2218-15 | GMKMW2218-15_ Rhiniinae |
| Rhiniinae | GMKMW2269-15 | GMKMW2269-15_ Rhiniinae |
| Rhiniinae | GMKMY1974-15 | GMKMY1974-15_ Rhiniinae |
| *Rhyncomya soyauxi* | GMKMY231-15 | GMKMY231-15_ Rhyncomya_soyauxi |
| Rhiniinae | GMMBI001-16 | GMMBI001-16_ Rhiniinae |
| Rhiniinae | GMMBI002-16 | GMMBI002-16_ Rhiniinae |
| Rhiniinae | GMMBR149-16 | GMMBR149-16_ Rhiniinae |
| Rhiniinae | GMMDB131-15 | GMMDB131-15_ Rhiniinae |
| Rhiniinae | GMMDC250-15 | GMMDC250-15_ Rhiniinae |
| Rhiniinae | GMMDD078-15 | GMMDD078-15_ Rhiniinae |
| Rhiniinae | GMMDD083-15 | GMMDD083-15_ Rhiniinae |
| Rhiniinae | GMMDE039-15 | GMMDE039-15_ Rhiniinae |
| Rhiniinae | GMMDE041-15 | GMMDE041-15_ Rhiniinae |
| Rhiniinae | GMMDE044-15 | GMMDE044-15_ Rhiniinae |
| Rhiniinae | GMMDF170-15 | GMMDF170-15_ Rhiniinae |
| Rhiniinae | GMMGA479-14 | GMMGA479-14_ Rhiniinae |
| Rhiniinae | GMPBB006-18 | GMPBB006-18_ Rhiniinae |
| Rhiniinae | GMPBD013-18 | GMPBD013-18_ Rhiniinae |
| Rhiniinae | GMPBH538-18 | GMPBH538-18_ Rhiniinae |
| Rhiniinae | GMPBK065-18 | GMPBK065-18_ Rhiniinae |
| Rhiniinae | GMPBK157-18 | GMPBK157-18_ Rhiniinae |
| Rhiniinae | GMPBK161-18 | GMPBK161-18_ Rhiniinae |
| Rhiniinae | GMPBK162-18 | GMPBK162-18_ Rhiniinae |
| Rhiniinae | GMPBK2143-18 | GMPBK2143-18_ Rhiniinae |
| Rhiniinae | GMPBK2174-18 | GMPBK2174-18_ Rhiniinae |
| Rhiniinae | GMPBK2181-18 | GMPBK2181-18_ Rhiniinae |
| Rhiniinae | GMPBK2183-18 | GMPBK2183-18_ Rhiniinae |
| Rhiniinae | GMPBK2200-18 | GMPBK2200-18_ Rhiniinae |
| Rhiniinae | GMPBK2212-18 | GMPBK2212-18_ Rhiniinae |
| Rhiniinae | GMPBK2780-18 | GMPBK2780-18_ Rhiniinae |
| Rhiniinae | GMPBK2803-18 | GMPBK2803-18_ Rhiniinae |
| Rhiniinae | GMPBK3490-18 | GMPBK3490-18_ Rhiniinae |
| Rhiniinae | GMPBL070-18 | GMPBL070-18_ Rhiniinae |
| Rhiniinae | GMPBL142-18 | GMPBL142-18_ Rhiniinae |
| Rhiniinae | GMPBM134-18 | GMPBM134-18_ Rhiniinae |
| Rhiniinae | GMPBN017-18 | GMPBN017-18_ Rhiniinae |
| Rhiniinae | GMPBP099-18 | GMPBP099-18_ Rhiniinae |
| Rhiniinae | GMPBW002-18 | GMPBW002-18_ Rhiniinae |
| Rhiniinae | GMSAA1283-13 | GMSAA1283-13_ Rhiniinae |
| Rhiniinae | GMSAB2598-13 | GMSAB2598-13_ Rhiniinae |
| Rhiniinae | GMSAC2134-13 | GMSAC2134-13_ Rhiniinae |
| Rhiniinae | GMSAP009-13 | GMSAP009-13_ Rhiniinae |
| Rhiniinae | GMSAP011-13 | GMSAP011-13_ Rhiniinae |
| Rhiniinae | GMSJK001-18 | GMSJK001-18_ Rhiniinae |
| Rhiniinae | GMSJN092-18 | GMSJN092-18_ Rhiniinae |
| Rhiniinae | GMSJR1132-18 | GMSJR1132-18_ Rhiniinae |
| Rhiniinae | GMSJR1197-18 | GMSJR1197-18_ Rhiniinae |
| Rhiniinae | GMSJS033-18 | GMSJS033-18_ Rhiniinae |
| Rhiniinae | GMSJT006-18 | GMSJT006-18_ Rhiniinae |
| Rhiniinae | GMSJV004-18 | GMSJV004-18_ Rhiniinae |
| Rhiniinae | ISER004-05 | ISER004-05_ Rhiniinae |
| Rhiniinae | KMPAB4626-18 | KMPAB4626-18_ Rhiniinae |
| Rhiniinae | KMPAB4671-18 | KMPAB4671-18_ Rhiniinae |
| Rhiniinae | KMPAD2407-19 | KMPAD2407-19_ Rhiniinae |
| *Rhyncomya soyauxi* | KMPAH001-19 | KMPAH001-19_ Rhyncomya_soyauxi |
| *Rhyncomya soyauxi* | KMPAH007-19 | KMPAH007-19_ Rhyncomya_soyauxi |
| *Rhyncomya soyauxi* | KMPAH033-19 | KMPAH033-19_ Rhyncomya_soyauxi |
| *Rhyncomya soyauxi* | KMPAH034-19 | KMPAH034-19_ Rhyncomya_soyauxi |
| *Rhyncomya soyauxi* | KMPAH035-19 | KMPAH035-19_ Rhyncomya_soyauxi |
| *Rhyncomya soyauxi* | KMPAH036-19 | KMPAH036-19_ Rhyncomya_soyauxi |
| Rhiniinae | KMPAH3838-19 | KMPAH3838-19_ Rhiniinae |
| *Rhyncomya soyauxi* | KMPAH3840-19 | KMPAH3840-19_ Rhyncomya_soyauxi |
| Rhiniinae | KMPAH3841-19 | KMPAH3841-19_ Rhiniinae |
| Rhiniinae | KMPAH3842-19 | KMPAH3842-19_ Rhiniinae |
| *Rhyncomya soyauxi* | KMPAH3843-19 | KMPAH3843-19_ Rhyncomya_soyauxi |
| Rhiniinae | KMPAH3845-19 | KMPAH3845-19_ Rhiniinae |
| Rhiniinae | KMPAJ027-19 | KMPAJ027-19_ Rhiniinae |
| Rhiniinae | KMPAJ1372-19 | KMPAJ1372-19_ Rhiniinae |
| Rhiniinae | KMPAJ193-19 | KMPAJ193-19_ Rhiniinae |
| Rhiniinae | KMPAJ212-19 | KMPAJ212-19_ Rhiniinae |
| *Rhyncomya soyauxi* | KMPAL1228-19 | KMPAL1228-19_ Rhyncomya_soyauxi |
| Rhiniinae | KMPAL1251-19 | KMPAL1251-19_ Rhiniinae |
| Rhiniinae | KMPAL1255-19 | KMPAL1255-19_ Rhiniinae |
| Rhiniinae | KMPAL1308-19 | KMPAL1308-19_ Rhiniinae |
| *Rhyncomya soyauxi* | KMPAL168-19 | KMPAL168-19_ Rhyncomya_soyauxi |
| Rhiniinae | KMPAL172-19 | KMPAL172-19_ Rhiniinae |
| *Rhyncomya soyauxi* | KMPAL3472-19 | KMPAL3472-19_ Rhyncomya_soyauxi |
| *Rhyncomya soyauxi* | KMPAL3474-19 | KMPAL3474-19_ Rhyncomya_soyauxi |
| Rhiniinae | KMPAL3481-19 | KMPAL3481-19_ Rhiniinae |
| Rhiniinae | KMPAL3492-19 | KMPAL3492-19_ Rhiniinae |
| *Rhyncomya soyauxi* | KMPAL3498-19 | KMPAL3498-19_ Rhyncomya_soyauxi |
| Rhiniinae | KMPAL3499-19 | KMPAL3499-19_ Rhiniinae |
| Rhiniinae | KMPAL3503-19 | KMPAL3503-19_ Rhiniinae |
| Rhiniinae | KMPAL3513-19 | KMPAL3513-19_ Rhiniinae |
| Rhiniinae | KMPAL3514-19 | KMPAL3514-19_ Rhiniinae |
| *Rhyncomya soyauxi* | KMPAL3515-19 | KMPAL3515-19_ Rhyncomya_soyauxi |
| *Rhyncomya soyauxi* | KMPAL3516-19 | KMPAL3516-19_ Rhyncomya_soyauxi |
| Rhiniinae | KMPAL3523-19 | KMPAL3523-19_ Rhiniinae |
| Rhiniinae | KMPAL3530-19 | KMPAL3530-19_ Rhiniinae |
| *Rhyncomya soyauxi* | KMPAL3533-19 | KMPAL3533-19_ Rhyncomya_soyauxi |
| Rhiniinae | KMPAL3541-19 | KMPAL3541-19_ Rhiniinae |
| Rhiniinae | KMPAL3545-19 | KMPAL3545-19_ Rhiniinae |
| Rhiniinae | KMPAL3552-19 | KMPAL3552-19_ Rhiniinae |
| *Rhyncomya soyauxi* | KMPAL3561-19 | KMPAL3561-19_ Rhyncomya_soyauxi |
| *Rhyncomya soyauxi* | KMPAL3568-19 | KMPAL3568-19_ Rhyncomya_soyauxi |
| Rhiniinae | KMPAL3574-19 | KMPAL3574-19_ Rhiniinae |
| Rhiniinae | KMPAL3593-19 | KMPAL3593-19_ Rhiniinae |
| Rhiniinae | KMPAL3764-19 | KMPAL3764-19_ Rhiniinae |
| Rhiniinae | KMPAL3769-19 | KMPAL3769-19_ Rhiniinae |
| Rhiniinae | KMPAL3773-19 | KMPAL3773-19_ Rhiniinae |
| Rhiniinae | KMPAL3775-19 | KMPAL3775-19_ Rhiniinae |
| Rhiniinae | KMPAL3778-19 | KMPAL3778-19_ Rhiniinae |
| Rhiniinae | KMPAN1048-19 | KMPAN1048-19_ Rhiniinae |
| *Rhyncomya soyauxi* | KMPAN294-19 | KMPAN294-19_ Rhyncomya_soyauxi |
| *Rhyncomya soyauxi* | KMPAN295-19 | KMPAN295-19_ Rhyncomya_soyauxi |
| *Rhyncomya soyauxi* | KMPAN305-19 | KMPAN305-19_ Rhyncomya_soyauxi |
| *Rhyncomya soyauxi* | KMPAN323-19 | KMPAN323-19_ Rhyncomya_soyauxi |
| *Rhyncomya soyauxi* | KMPAN359-19 | KMPAN359-19_ Rhyncomya_soyauxi |
| *Rhyncomya soyauxi* | KMPBB317-18 | KMPBB317-18_ Rhyncomya_soyauxi |
| Rhiniinae | KMPCA008-18 | KMPCA008-18_ Rhiniinae |
| Rhiniinae | KMPCK025-19 | KMPCK025-19_ Rhiniinae |
| Rhiniinae | KMPCL053-19 | KMPCL053-19_ Rhiniinae |
| Rhiniinae | KMPCN128-19 | KMPCN128-19_ Rhiniinae |
| *Rhyncomya soyauxi* | KMPCN343-19 | KMPCN343-19_ Rhyncomya_soyauxi |
| Rhiniinae | KMPCO422-19 | KMPCO422-19_ Rhiniinae |
| Rhiniinae | KMPCO426-19 | KMPCO426-19_ Rhiniinae |
| Rhiniinae | KMPCO427-19 | KMPCO427-19_ Rhiniinae |
| Rhiniinae | KMPCP048-19 | KMPCP048-19_ Rhiniinae |
| Rhiniinae | KMPCP049-19 | KMPCP049-19_ Rhiniinae |
| Rhiniinae | KMPCP586-19 | KMPCP586-19_ Rhiniinae |
| *Rhyncomya soyauxi* | KMPCP592-19 | KMPCP592-19_ Rhyncomya_soyauxi |
| *Rhyncomya soyauxi* | KMPCP597-19 | KMPCP597-19_ Rhyncomya_soyauxi |
| Rhiniinae | KMPCP599-19 | KMPCP599-19_ Rhiniinae |
| *Rhyncomya soyauxi* | KMPCP600-19 | KMPCP600-19_ Rhyncomya_soyauxi |
| *Rhyncomya soyauxi* | KMPCP602-19 | KMPCP602-19_ Rhyncomya_soyauxi |
| *Rhyncomya soyauxi* | KMPDA136-19 | KMPDA136-19_ Rhyncomya_soyauxi |
| Rhiniinae | KMPDA143-19 | KMPDA143-19_ Rhiniinae |
| Rhiniinae | KMPDA277-19 | KMPDA277-19_ Rhiniinae |
| Rhiniinae | KMPDA343-19 | KMPDA343-19_ Rhiniinae |
| *Rhyncomya soyauxi* | KMPDB058-19 | KMPDB058-19_ Rhyncomya_soyauxi |
| *Rhyncomya soyauxi* | KMPDB060-19 | KMPDB060-19_ Rhyncomya_soyauxi |
| Rhiniinae | KMPDB257-19 | KMPDB257-19_ Rhiniinae |
| *Rhyncomya soyauxi* | KMPDC024-19 | KMPDC024-19_ Rhyncomya_soyauxi |
| *Rhyncomya soyauxi* | KMPDC038-19 | KMPDC038-19_ Rhyncomya_soyauxi |
| *Rhyncomya soyauxi* | KMPDD073-19 | KMPDD073-19_ Rhyncomya_soyauxi |
| Rhiniinae | KMPDD074-19 | KMPDD074-19_ Rhiniinae |
| Rhiniinae | KMPDD117-19 | KMPDD117-19_ Rhiniinae |
| Rhiniinae | KMPDD134-19 | KMPDD134-19_ Rhiniinae |
| Rhiniinae | KMPDD145-19 | KMPDD145-19_ Rhiniinae |
| *Rhyncomya soyauxi* | KMPDD207-19 | KMPDD207-19_ Rhyncomya_soyauxi |
| Rhiniinae | KMPDE072-19 | KMPDE072-19_ Rhiniinae |
| *Rhyncomya soyauxi* | KMPDE103-19 | KMPDE103-19_ Rhyncomya_soyauxi |
| *Rhyncomya soyauxi* | KMPDE117-19 | KMPDE117-19_ Rhyncomya_soyauxi |
| Rhiniinae | KMPDE165-19 | KMPDE165-19_ Rhiniinae |
| *Rhyncomya soyauxi* | KMPDE167-19 | KMPDE167-19_ Rhyncomya_soyauxi |
| *Rhyncomya soyauxi* | KMPDE178-19 | KMPDE178-19_ Rhyncomya_soyauxi |
| Rhiniinae | KMPDE961-19 | KMPDE961-19_ Rhiniinae |
| Rhiniinae | KMPDF1858-19 | KMPDF1858-19_ Rhiniinae |
| Rhiniinae | KMPDF1861-19 | KMPDF1861-19_ Rhiniinae |
| Rhiniinae | KMPDF1896-19 | KMPDF1896-19_ Rhiniinae |
| *Rhyncomya soyauxi* | KMPDF1899-19 | KMPDF1899-19_ Rhyncomya_soyauxi |
| Rhiniinae | KMPDG117-19 | KMPDG117-19_ Rhiniinae |
| *Rhyncomya soyauxi* | KMPDG148-19 | KMPDG148-19_ Rhyncomya_soyauxi |
| Rhiniinae | KMPDG151-19 | KMPDG151-19_ Rhiniinae |
| Rhiniinae | KMPDG163-19 | KMPDG163-19_ Rhiniinae |
| Rhiniinae | KMPDG167-19 | KMPDG167-19_ Rhiniinae |
| Rhiniinae | KMPDG703-19 | KMPDG703-19_ Rhiniinae |
| *Rhyncomya soyauxi* | KMPDH061-19 | KMPDH061-19_ Rhyncomya_soyauxi |
| Rhiniinae | KMPDH063-19 | KMPDH063-19_ Rhiniinae |
| Rhiniinae | KMPDH073-19 | KMPDH073-19_ Rhiniinae |
| Rhiniinae | KMPDH075-19 | KMPDH075-19_ Rhiniinae |
| Rhiniinae | KMPDI162-19 | KMPDI162-19_ Rhiniinae |
| Rhiniinae | KMPDI163-19 | KMPDI163-19_ Rhiniinae |
| Rhiniinae | KMPDI168-19 | KMPDI168-19_ Rhiniinae |
| Rhiniinae | KMPDI181-19 | KMPDI181-19_ Rhiniinae |
| Rhiniinae | KMPDI190-19 | KMPDI190-19_ Rhiniinae |
| *Rhyncomya soyauxi* | KMPDI202-19 | KMPDI202-19_ Rhyncomya_soyauxi |
| Rhiniinae | KMPDI213-19 | KMPDI213-19_ Rhiniinae |
| Rhiniinae | KMPDI220-19 | KMPDI220-19_ Rhiniinae |
| Rhiniinae | KMPDI407-19 | KMPDI407-19_ Rhiniinae |
| Rhiniinae | KMPDI414-19 | KMPDI414-19_ Rhiniinae |
| Rhiniinae | KMPDJ1000-19 | KMPDJ1000-19_ Rhiniinae |
| Rhiniinae | KMPDJ1460-19 | KMPDJ1460-19_ Rhiniinae |
| Rhiniinae | KMPDJ182-19 | KMPDJ182-19_ Rhiniinae |
| Rhiniinae | KMPDJ186-19 | KMPDJ186-19_ Rhiniinae |
| Rhiniinae | KMPDJ188-19 | KMPDJ188-19_ Rhiniinae |
| Rhiniinae | KMPDJ189-19 | KMPDJ189-19_ Rhiniinae |
| Rhiniinae | KMPDJ192-19 | KMPDJ192-19_ Rhiniinae |
| Rhiniinae | KMPDJ197-19 | KMPDJ197-19_ Rhiniinae |
| Rhiniinae | KMPDJ208-19 | KMPDJ208-19_ Rhiniinae |
| Rhiniinae | KMPDJ214-19 | KMPDJ214-19_ Rhiniinae |
| Rhiniinae | KMPDJ2238-19 | KMPDJ2238-19_ Rhiniinae |
| Rhiniinae | KMPDJ456-19 | KMPDJ456-19_ Rhiniinae |
| Rhiniinae | KMPDJ929-19 | KMPDJ929-19_ Rhiniinae |
| Rhiniinae | KMPDM072-19 | KMPDM072-19_ Rhiniinae |
| Rhiniinae | KMPEA132-18 | KMPEA132-18_ Rhiniinae |
| Rhiniinae | KMPEC632-18 | KMPEC632-18_ Rhiniinae |
| *Rhyncomya soyauxi* | KMPED003-18 | KMPED003-18_ Rhyncomya_soyauxi |
| *Rhyncomya soyauxi* | KMPED012-18 | KMPED012-18_ Rhyncomya_soyauxi |
| Rhiniinae | KMPED013-18 | KMPED013-18_ Rhiniinae |
| Rhiniinae | KMPED043-18 | KMPED043-18_ Rhiniinae |
| *Rhyncomya soyauxi* | KMPED049-18 | KMPED049-18_ Rhyncomya_soyauxi |
| Rhiniinae | KMPED088-18 | KMPED088-18_ Rhiniinae |
| Rhiniinae | KMPEE056-18 | KMPEE056-18_ Rhiniinae |
| Rhiniinae | KMPEF308-19 | KMPEF308-19_ Rhiniinae |
| *Rhyncomya soyauxi* | KMPEG065-19 | KMPEG065-19_ Rhyncomya_soyauxi |
| *Rhyncomya soyauxi* | KMPEG070-19 | KMPEG070-19_ Rhyncomya_soyauxi |
| Rhiniinae | KMPEH241-19 | KMPEH241-19_ Rhiniinae |
| Rhiniinae | KMPEH283-19 | KMPEH283-19_ Rhiniinae |
| Rhiniinae | KMPEH284-19 | KMPEH284-19_ Rhiniinae |
| Rhiniinae | KMPEH285-19 | KMPEH285-19_ Rhiniinae |
| Rhiniinae | KMPEH286-19 | KMPEH286-19_ Rhiniinae |
| Rhiniinae | KMPEK756-19 | KMPEK756-19_ Rhiniinae |
| Rhiniinae | KMPEL052-19 | KMPEL052-19_ Rhiniinae |
| *Rhyncomya soyauxi* | KMPEM1160-19 | KMPEM1160-19_ Rhyncomya_soyauxi |
| Rhiniinae | KMPEO1242-19 | KMPEO1242-19_ Rhiniinae |
| Rhiniinae | KMPFB141-18 | KMPFB141-18_ Rhiniinae |
| Rhiniinae | KMPFE375-18 | KMPFE375-18_ Rhiniinae |
| Rhiniinae | KMPFH092-19 | KMPFH092-19_ Rhiniinae |
| Rhiniinae | KMPFH216-19 | KMPFH216-19_ Rhiniinae |
| Rhiniinae | KMPFI125-19 | KMPFI125-19_ Rhiniinae |
| Rhiniinae | KMPFI133-19 | KMPFI133-19_ Rhiniinae |
| Rhiniinae | KMPFJ091-19 | KMPFJ091-19_ Rhiniinae |
| Rhiniinae | KMPFN007-19 | KMPFN007-19_ Rhiniinae |
| Rhiniinae | KMPFR135-19 | KMPFR135-19_ Rhiniinae |
| Rhiniinae | KMPFR137-19 | KMPFR137-19_ Rhiniinae |
| Rhiniinae | KMPFR138-19 | KMPFR138-19_ Rhiniinae |
| Rhiniinae | KMPFR141-19 | KMPFR141-19_ Rhiniinae |
| Rhiniinae | KMPFS030-19 | KMPFS030-19_ Rhiniinae |
| Rhiniinae | KMPFS032-19 | KMPFS032-19_ Rhiniinae |
| Rhiniinae | KMPFS033-19 | KMPFS033-19_ Rhiniinae |
| Rhiniinae | KMPFS037-19 | KMPFS037-19_ Rhiniinae |
| Rhiniinae | KMPFT011-19 | KMPFT011-19_ Rhiniinae |
| Rhiniinae | KMPFT012-19 | KMPFT012-19_ Rhiniinae |
| Rhiniinae | KMPGA139-18 | KMPGA139-18_ Rhiniinae |
| Rhiniinae | KMPGB135-18 | KMPGB135-18_ Rhiniinae |
| Rhiniinae | KMPGM300-19 | KMPGM300-19_ Rhiniinae |
| *Rhyncomya soyauxi* | KMPGM302-19 | KMPGM302-19_ Rhyncomya_soyauxi |
| Rhiniinae | KMPGO035-19 | KMPGO035-19_ Rhiniinae |
| *Rhyncomya soyauxi* | KMPGP111-19 | KMPGP111-19_ Rhyncomya_soyauxi |
| Rhiniinae | KMPGQ161-19 | KMPGQ161-19_ Rhiniinae |
| Rhiniinae | KMPGQ432-19 | KMPGQ432-19_ Rhiniinae |
| Rhiniinae | KMPGQ433-19 | KMPGQ433-19_ Rhiniinae |
| Rhiniinae | KMPGS994-19 | KMPGS994-19_ Rhiniinae |
| Rhiniinae | KMPGT039-19 | KMPGT039-19_ Rhiniinae |
| Rhiniinae | KMPGU116-19 | KMPGU116-19_ Rhiniinae |
| Rhiniinae | KMPHA111-18 | KMPHA111-18_ Rhiniinae |
| Rhiniinae | KMPHA131-18 | KMPHA131-18_ Rhiniinae |
| Rhiniinae | KMPHB583-18 | KMPHB583-18_ Rhiniinae |
| Rhiniinae | KMPHC268-18 | KMPHC268-18_ Rhiniinae |
| Rhiniinae | KMPHE323-19 | KMPHE323-19_ Rhiniinae |
| Rhiniinae | KMPHF300-19 | KMPHF300-19_ Rhiniinae |
| Rhiniinae | KMPHG024-19 | KMPHG024-19_ Rhiniinae |
| Rhiniinae | KMPHG026-19 | KMPHG026-19_ Rhiniinae |
| Rhiniinae | KMPHH035-19 | KMPHH035-19_ Rhiniinae |
| *Rhyncomya soyauxi* | KMPHI410-19 | KMPHI410-19_ Rhyncomya_soyauxi |
| Rhiniinae | KMPHI411-19 | KMPHI411-19_ Rhiniinae |
| Rhiniinae | KMPHJ075-19 | KMPHJ075-19_ Rhiniinae |
| Rhiniinae | KMPHJ076-19 | KMPHJ076-19_ Rhiniinae |
| Rhiniinae | KMPHJ078-19 | KMPHJ078-19_ Rhiniinae |
| *Rhyncomya soyauxi* | KMPHJ080-19 | KMPHJ080-19_ Rhyncomya_soyauxi |
| Rhiniinae | KMPHL515-19 | KMPHL515-19_ Rhiniinae |
| Rhiniinae | KMPHL516-19 | KMPHL516-19_ Rhiniinae |
| Rhiniinae | KMPHL517-19 | KMPHL517-19_ Rhiniinae |
| Rhiniinae | KMPHM050-19 | KMPHM050-19_ Rhiniinae |
| Rhiniinae | KMPHM353-19 | KMPHM353-19_ Rhiniinae |
| Rhiniinae | KMPHM360-19 | KMPHM360-19_ Rhiniinae |
| Rhiniinae | KMPHN1942-19 | KMPHN1942-19_ Rhiniinae |
| Rhiniinae | KMPHN1943-19 | KMPHN1943-19_ Rhiniinae |
| Rhiniinae | KMPHN1951-19 | KMPHN1951-19_ Rhiniinae |
| *Rhyncomya soyauxi* | KMPHN1955-19 | KMPHN1955-19_ Rhyncomya_soyauxi |
| Rhiniinae | KMPHN1959-19 | KMPHN1959-19_ Rhiniinae |
| Rhiniinae | KMPHN1960-19 | KMPHN1960-19_ Rhiniinae |
| Rhiniinae | KMPHN1965-19 | KMPHN1965-19_ Rhiniinae |
| Rhiniinae | KMPHN1967-19 | KMPHN1967-19_ Rhiniinae |
| Rhiniinae | KMPHN1971-19 | KMPHN1971-19_ Rhiniinae |
| Rhiniinae | KMPHN1973-19 | KMPHN1973-19_ Rhiniinae |
| Rhiniinae | KMPHN1974-19 | KMPHN1974-19_ Rhiniinae |
| Rhiniinae | KMPHN1978-19 | KMPHN1978-19_ Rhiniinae |
| Rhiniinae | KMPHN1979-19 | KMPHN1979-19_ Rhiniinae |
| Rhiniinae | KMPHO042-19 | KMPHO042-19_ Rhiniinae |
| Rhiniinae | KMPHO043-19 | KMPHO043-19_ Rhiniinae |
| Rhiniinae | KMPHO045-19 | KMPHO045-19_ Rhiniinae |
| Rhiniinae | KMPHO046-19 | KMPHO046-19_ Rhiniinae |
| Rhiniinae | KMPHO047-19 | KMPHO047-19_ Rhiniinae |
| Rhiniinae | KMPHO057-19 | KMPHO057-19_ Rhiniinae |
| Rhiniinae | KMPHP045-19 | KMPHP045-19_ Rhiniinae |
| Rhiniinae | KMPHQ1269-19 | KMPHQ1269-19_ Rhiniinae |
| Rhiniinae | KMPHQ1276-19 | KMPHQ1276-19_ Rhiniinae |
| Rhiniinae | KMPHR034-19 | KMPHR034-19_ Rhiniinae |
| *Rhyncomya soyauxi* | KMPHS132-19 | KMPHS132-19_ Rhyncomya_soyauxi |
| Rhiniinae | KMPHS134-19 | KMPHS134-19_ Rhiniinae |
| Rhiniinae | KMPHS138-19 | KMPHS138-19_ Rhiniinae |
| Rhiniinae | KMPHS140-19 | KMPHS140-19_ Rhiniinae |
| Rhiniinae | KMPHS142-19 | KMPHS142-19_ Rhiniinae |
| Rhiniinae | KMPHS143-19 | KMPHS143-19_ Rhiniinae |
| Rhiniinae | KMPHS149-19 | KMPHS149-19_ Rhiniinae |
| Rhiniinae | KMPHX021-19 | KMPHX021-19_ Rhiniinae |
| Rhiniinae | KMPHY036-19 | KMPHY036-19_ Rhiniinae |
| Rhiniinae | KMPHY042-19 | KMPHY042-19_ Rhiniinae |
| Rhiniinae | KMPIA240-18 | KMPIA240-18_ Rhiniinae |
| Rhiniinae | KMPIA255-18 | KMPIA255-18_ Rhiniinae |
| Rhiniinae | KMPIA262-18 | KMPIA262-18_ Rhiniinae |
| *Rhyncomya soyauxi* | KMPIA267-18 | KMPIA267-18_ Rhyncomya_soyauxi |
| Rhiniinae | KMPIC004-18 | KMPIC004-18_ Rhiniinae |
| Rhiniinae | KMPIC013-18 | KMPIC013-18_ Rhiniinae |
| Rhiniinae | KMPII044-19 | KMPII044-19_ Rhiniinae |
| Rhiniinae | KMPII045-19 | KMPII045-19_ Rhiniinae |
| *Rhyncomya soyauxi* | KMPII089-19 | KMPII089-19_ Rhyncomya_soyauxi |
| Rhiniinae | KMPII095-19 | KMPII095-19_ Rhiniinae |
| Rhiniinae | KMPIJ181-19 | KMPIJ181-19_ Rhiniinae |
| Rhiniinae | KMPIJ182-19 | KMPIJ182-19_ Rhiniinae |
| Rhiniinae | KMPIK064-19 | KMPIK064-19_ Rhiniinae |
| Rhiniinae | KMPIK142-19 | KMPIK142-19_ Rhiniinae |
| Rhiniinae | KMPIL231-19 | KMPIL231-19_ Rhiniinae |
| Rhiniinae | KMPIL234-19 | KMPIL234-19_ Rhiniinae |
| Rhiniinae | KMPIL236-19 | KMPIL236-19_ Rhiniinae |
| Rhiniinae | KMPIL238-19 | KMPIL238-19_ Rhiniinae |
| Rhiniinae | KMPIL239-19 | KMPIL239-19_ Rhiniinae |
| Rhiniinae | KMPIL240-19 | KMPIL240-19_ Rhiniinae |
| Rhiniinae | KMPIL300-19 | KMPIL300-19_ Rhiniinae |
| Rhiniinae | KMPIM021-19 | KMPIM021-19_ Rhiniinae |
| Rhiniinae | KMPIM024-19 | KMPIM024-19_ Rhiniinae |
| Rhiniinae | KMPIM026-19 | KMPIM026-19_ Rhiniinae |
| Rhiniinae | KMPIM028-19 | KMPIM028-19_ Rhiniinae |
| Rhiniinae | KMPIN001-19 | KMPIN001-19_ Rhiniinae |
| Rhiniinae | KMPIN003-19 | KMPIN003-19_ Rhiniinae |
| Rhiniinae | KMPIN004-19 | KMPIN004-19_ Rhiniinae |
| Rhiniinae | KMPIN005-19 | KMPIN005-19_ Rhiniinae |
| Rhiniinae | KMPIN006-19 | KMPIN006-19_ Rhiniinae |
| Rhiniinae | KMPIN007-19 | KMPIN007-19_ Rhiniinae |
| Rhiniinae | KMPIO147-19 | KMPIO147-19_ Rhiniinae |
| Rhiniinae | KMPIO149-19 | KMPIO149-19_ Rhiniinae |
| Rhiniinae | KMPIO150-19 | KMPIO150-19_ Rhiniinae |
| Rhiniinae | KMPIO151-19 | KMPIO151-19_ Rhiniinae |
| Rhiniinae | KMPIO152-19 | KMPIO152-19_ Rhiniinae |
| Rhiniinae | KMPIO153-19 | KMPIO153-19_ Rhiniinae |
| Rhiniinae | KMPIO154-19 | KMPIO154-19_ Rhiniinae |
| Rhiniinae | KMPIO155-19 | KMPIO155-19_ Rhiniinae |
| Rhiniinae | KMPIO156-19 | KMPIO156-19_ Rhiniinae |
| Rhiniinae | KMPIO157-19 | KMPIO157-19_ Rhiniinae |
| Rhiniinae | KMPIO158-19 | KMPIO158-19_ Rhiniinae |
| Rhiniinae | KMPIO159-19 | KMPIO159-19_ Rhiniinae |
| Rhiniinae | KMPIO160-19 | KMPIO160-19_ Rhiniinae |
| *Rhyncomya soyauxi* | KMPIO162-19 | KMPIO162-19_ Rhyncomya_soyauxi |
| Rhiniinae | KMPIO163-19 | KMPIO163-19_ Rhiniinae |
| Rhiniinae | KMPIO164-19 | KMPIO164-19_ Rhiniinae |
| Rhiniinae | KMPIO165-19 | KMPIO165-19_ Rhiniinae |
| Rhiniinae | KMPIO166-19 | KMPIO166-19_ Rhiniinae |
| Rhiniinae | KMPIO167-19 | KMPIO167-19_ Rhiniinae |
| Rhiniinae | KMPIO168-19 | KMPIO168-19_ Rhiniinae |
| Rhiniinae | KMPIO170-19 | KMPIO170-19_ Rhiniinae |
| Rhiniinae | KMPIO171-19 | KMPIO171-19_ Rhiniinae |
| Rhiniinae | KMPIO172-19 | KMPIO172-19_ Rhiniinae |
| Rhiniinae | KMPIO173-19 | KMPIO173-19_ Rhiniinae |
| Rhiniinae | KMPIO174-19 | KMPIO174-19_ Rhiniinae |
| Rhiniinae | KMPIO176-19 | KMPIO176-19_ Rhiniinae |
| Rhiniinae | KMPIO177-19 | KMPIO177-19_ Rhiniinae |
| Rhiniinae | KMPIO178-19 | KMPIO178-19_ Rhiniinae |
| Rhiniinae | KMPIO179-19 | KMPIO179-19_ Rhiniinae |
| Rhiniinae | KMPIO180-19 | KMPIO180-19_ Rhiniinae |
| Rhiniinae | KMPIO181-19 | KMPIO181-19_ Rhiniinae |
| Rhiniinae | KMPIO182-19 | KMPIO182-19_ Rhiniinae |
| Rhiniinae | KMPIO183-19 | KMPIO183-19_ Rhiniinae |
| Rhiniinae | KMPIO186-19 | KMPIO186-19_ Rhiniinae |
| Rhiniinae | KMPIP216-19 | KMPIP216-19_ Rhiniinae |
| Rhiniinae | KMPIP217-19 | KMPIP217-19_ Rhiniinae |
| Rhiniinae | KMPIP222-19 | KMPIP222-19_ Rhiniinae |
| Rhiniinae | KMPIP225-19 | KMPIP225-19_ Rhiniinae |
| Rhiniinae | KMPIP226-19 | KMPIP226-19_ Rhiniinae |
| Rhiniinae | KMPIP228-19 | KMPIP228-19_ Rhiniinae |
| Rhiniinae | KMPIP230-19 | KMPIP230-19_ Rhiniinae |
| Rhiniinae | KMPIP232-19 | KMPIP232-19_ Rhiniinae |
| Rhiniinae | KMPIP235-19 | KMPIP235-19_ Rhiniinae |
| Rhiniinae | KMPIP236-19 | KMPIP236-19_ Rhiniinae |
| Rhiniinae | KMPIP237-19 | KMPIP237-19_ Rhiniinae |
| Rhiniinae | KMPIP240-19 | KMPIP240-19_ Rhiniinae |
| Rhiniinae | KMPIP247-19 | KMPIP247-19_ Rhiniinae |
| Rhiniinae | KMPIP248-19 | KMPIP248-19_ Rhiniinae |
| Rhiniinae | KMPIP249-19 | KMPIP249-19_ Rhiniinae |
| Rhiniinae | KMPIP251-19 | KMPIP251-19_ Rhiniinae |
| Rhiniinae | KMPIP252-19 | KMPIP252-19_ Rhiniinae |
| Rhiniinae | KMPIP254-19 | KMPIP254-19_ Rhiniinae |
| Rhiniinae | KMPIP256-19 | KMPIP256-19_ Rhiniinae |
| *Rhyncomya soyauxi* | KMPIP260-19 | KMPIP260-19_ Rhyncomya_soyauxi |
| Rhiniinae | KMPIP262-19 | KMPIP262-19_ Rhiniinae |
| Rhiniinae | KMPIP273-19 | KMPIP273-19_ Rhiniinae |
| Rhiniinae | KMPIP278-19 | KMPIP278-19_ Rhiniinae |
| Rhiniinae | KMPIP287-19 | KMPIP287-19_ Rhiniinae |
| Rhiniinae | KMPIP288-19 | KMPIP288-19_ Rhiniinae |
| Rhiniinae | KMPIP289-19 | KMPIP289-19_ Rhiniinae |
| Rhiniinae | KMPIP290-19 | KMPIP290-19_ Rhiniinae |
| Rhiniinae | KMPIP291-19 | KMPIP291-19_ Rhiniinae |
| Rhiniinae | KMPIP292-19 | KMPIP292-19_ Rhiniinae |
| Rhiniinae | KMPIP293-19 | KMPIP293-19_ Rhiniinae |
| Rhiniinae | KMPIP296-19 | KMPIP296-19_ Rhiniinae |
| Rhiniinae | KMPIP297-19 | KMPIP297-19_ Rhiniinae |
| Rhiniinae | KMPIP299-19 | KMPIP299-19_ Rhiniinae |
| Rhiniinae | KMPIQ020-19 | KMPIQ020-19_ Rhiniinae |
| Rhiniinae | KMPIQ021-19 | KMPIQ021-19_ Rhiniinae |
| Rhiniinae | KMPIQ022-19 | KMPIQ022-19_ Rhiniinae |
| Rhiniinae | KMPIQ023-19 | KMPIQ023-19_ Rhiniinae |
| Rhiniinae | KMPIQ024-19 | KMPIQ024-19_ Rhiniinae |
| Rhiniinae | KMPIQ025-19 | KMPIQ025-19_ Rhiniinae |
| Rhiniinae | KMPIQ030-19 | KMPIQ030-19_ Rhiniinae |
| Rhiniinae | KMPIQ037-19 | KMPIQ037-19_ Rhiniinae |
| Rhiniinae | KMPIR011-19 | KMPIR011-19_ Rhiniinae |
| Rhiniinae | KMPIS671-19 | KMPIS671-19_ Rhiniinae |
| Rhiniinae | KMPIS673-19 | KMPIS673-19_ Rhiniinae |
| Rhiniinae | KMPIS675-19 | KMPIS675-19_ Rhiniinae |
| Rhiniinae | KMPIS678-19 | KMPIS678-19_ Rhiniinae |
| Rhiniinae | KMPIS679-19 | KMPIS679-19_ Rhiniinae |
| Rhiniinae | KMPIS680-19 | KMPIS680-19_ Rhiniinae |
| Rhiniinae | KMPIS684-19 | KMPIS684-19_ Rhiniinae |
| Rhiniinae | KMPIS686-19 | KMPIS686-19_ Rhiniinae |
| Rhiniinae | KMPIT131-19 | KMPIT131-19_ Rhiniinae |
| Rhiniinae | KMPIT136-19 | KMPIT136-19_ Rhiniinae |
| Rhiniinae | KMPIT138-19 | KMPIT138-19_ Rhiniinae |
| Rhiniinae | KMPIT142-19 | KMPIT142-19_ Rhiniinae |
| Rhiniinae | KMPIT144-19 | KMPIT144-19_ Rhiniinae |
| Rhiniinae | KMPIT148-19 | KMPIT148-19_ Rhiniinae |
| Rhiniinae | KMPIT154-19 | KMPIT154-19_ Rhiniinae |
| Rhiniinae | KMPIT156-19 | KMPIT156-19_ Rhiniinae |
| Rhiniinae | KMPIT161-19 | KMPIT161-19_ Rhiniinae |
| Rhiniinae | KMPIT428-19 | KMPIT428-19_ Rhiniinae |
| Rhiniinae | KMPIU181-19 | KMPIU181-19_ Rhiniinae |
| Rhiniinae | KMPIU594-19 | KMPIU594-19_ Rhiniinae |
| Rhiniinae | KMPIU596-19 | KMPIU596-19_ Rhiniinae |
| Rhiniinae | KMPIU597-19 | KMPIU597-19_ Rhiniinae |
| Rhiniinae | KMPIU603-19 | KMPIU603-19_ Rhiniinae |
| Rhiniinae | KMPIU610-19 | KMPIU610-19_ Rhiniinae |
| Rhiniinae | KMPIU611-19 | KMPIU611-19_ Rhiniinae |
| Rhiniinae | KMPIU614-19 | KMPIU614-19_ Rhiniinae |
| Rhiniinae | KMPIU620-19 | KMPIU620-19_ Rhiniinae |
| Rhiniinae | KMPIU626-19 | KMPIU626-19_ Rhiniinae |
| Rhiniinae | KMPIU629-19 | KMPIU629-19_ Rhiniinae |
| Rhiniinae | KMPIV022-19 | KMPIV022-19_ Rhiniinae |
| Rhiniinae | KMPIV025-19 | KMPIV025-19_ Rhiniinae |
| Rhiniinae | KMPIV029-19 | KMPIV029-19_ Rhiniinae |
| Rhiniinae | KMPIV030-19 | KMPIV030-19_ Rhiniinae |
| Rhiniinae | KMPJB015-18 | KMPJB015-18_ Rhiniinae |
| Rhiniinae | KMPJF263-19 | KMPJF263-19_ Rhiniinae |
| Rhiniinae | KMPJF267-19 | KMPJF267-19_ Rhiniinae |
| Rhiniinae | KMPJI050-19 | KMPJI050-19_ Rhiniinae |
| Rhiniinae | KMPJI051-19 | KMPJI051-19_ Rhiniinae |
| Rhiniinae | KMPJI052-19 | KMPJI052-19_ Rhiniinae |
| Rhiniinae | KMPJI058-19 | KMPJI058-19_ Rhiniinae |
| Rhiniinae | KMPJI063-19 | KMPJI063-19_ Rhiniinae |
| Rhiniinae | KMPJI064-19 | KMPJI064-19_ Rhiniinae |
| Rhiniinae | KMPJI067-19 | KMPJI067-19_ Rhiniinae |
| Rhiniinae | KMPJJ480-19 | KMPJJ480-19_ Rhiniinae |
| Rhiniinae | KMPJJ692-19 | KMPJJ692-19_ Rhiniinae |
| Rhiniinae | KMPJK103-19 | KMPJK103-19_ Rhiniinae |
| Rhiniinae | KMPJK155-19 | KMPJK155-19_ Rhiniinae |
| Rhiniinae | KMPJK158-19 | KMPJK158-19_ Rhiniinae |
| *Rhyncomya soyauxi* | KMPJK160-19 | KMPJK160-19_ Rhyncomya_soyauxi |
| Rhiniinae | KMPJK162-19 | KMPJK162-19_ Rhiniinae |
| Rhiniinae | KMPJK227-19 | KMPJK227-19_ Rhiniinae |
| Rhiniinae | KMPJK229-19 | KMPJK229-19_ Rhiniinae |
| Rhiniinae | KMPJL029-19 | KMPJL029-19_ Rhiniinae |
| Rhiniinae | KMPJL030-19 | KMPJL030-19_ Rhiniinae |
| Rhiniinae | KMPJL040-19 | KMPJL040-19_ Rhiniinae |
| *Rhyncomya soyauxi* | KMPJL045-19 | KMPJL045-19_ Rhyncomya_soyauxi |
| Rhiniinae | KMPJL584-19 | KMPJL584-19_ Rhiniinae |
| Rhiniinae | KMPJM189-19 | KMPJM189-19_ Rhiniinae |
| Rhiniinae | KMPJM191-19 | KMPJM191-19_ Rhiniinae |
| Rhiniinae | KMPJM193-19 | KMPJM193-19_ Rhiniinae |
| Rhiniinae | KMPJM194-19 | KMPJM194-19_ Rhiniinae |
| Rhiniinae | KMPJN461-19 | KMPJN461-19_ Rhiniinae |
| Rhiniinae | KMPJN525-19 | KMPJN525-19_ Rhiniinae |
| Rhiniinae | KMPJO1838-19 | KMPJO1838-19_ Rhiniinae |
| Rhiniinae | KMPJP114-19 | KMPJP114-19_ Rhiniinae |
| Rhiniinae | KMPJP2434-19 | KMPJP2434-19_ Rhiniinae |
| Rhiniinae | KMPJP2435-19 | KMPJP2435-19_ Rhiniinae |
| Rhiniinae | KMPJP2438-19 | KMPJP2438-19_ Rhiniinae |
| Rhiniinae | KMPJQ046-19 | KMPJQ046-19_ Rhiniinae |
| Rhiniinae | KMPJQ074-19 | KMPJQ074-19_ Rhiniinae |
| Rhiniinae | KMPJR294-19 | KMPJR294-19_ Rhiniinae |
| Rhiniinae | KMPJT030-19 | KMPJT030-19_ Rhiniinae |
| Rhiniinae | KMPJT032-19 | KMPJT032-19_ Rhiniinae |
| Rhiniinae | KMPJT034-19 | KMPJT034-19_ Rhiniinae |
| Rhiniinae | KMPJU057-19 | KMPJU057-19_ Rhiniinae |
| Rhiniinae | KMPJV085-19 | KMPJV085-19_ Rhiniinae |
| Rhiniinae | KMPJV093-19 | KMPJV093-19_ Rhiniinae |
| Rhiniinae | KMPJV094-19 | KMPJV094-19_ Rhiniinae |
| Rhiniinae | KMPJV096-19 | KMPJV096-19_ Rhiniinae |
| Rhiniinae | KMPJV137-19 | KMPJV137-19_ Rhiniinae |
| Rhiniinae | KMPJV193-19 | KMPJV193-19_ Rhiniinae |
| Rhiniinae | KMPJV296-19 | KMPJV296-19_ Rhiniinae |
| Rhiniinae | KMPJV556-19 | KMPJV556-19_ Rhiniinae |
| Rhiniinae | KMPJV590-19 | KMPJV590-19_ Rhiniinae |
| Rhiniinae | KMPKB001-18 | KMPKB001-18_ Rhiniinae |
| Rhiniinae | KMPKB064-18 | KMPKB064-18_ Rhiniinae |
| Rhiniinae | KMPKI026-19 | KMPKI026-19_ Rhiniinae |
| Rhiniinae | KMPKJ001-19 | KMPKJ001-19_ Rhiniinae |
| Rhiniinae | KMPKK055-19 | KMPKK055-19_ Rhiniinae |
| Rhiniinae | KMPKK056-19 | KMPKK056-19_ Rhiniinae |
| Rhiniinae | KMPKK057-19 | KMPKK057-19_ Rhiniinae |
| Rhiniinae | KMPKK058-19 | KMPKK058-19_ Rhiniinae |
| Rhiniinae | KMPKK059-19 | KMPKK059-19_ Rhiniinae |
| Rhiniinae | KMPKK061-19 | KMPKK061-19_ Rhiniinae |
| Rhiniinae | KMPKK062-19 | KMPKK062-19_ Rhiniinae |
| Rhiniinae | KMPKK063-19 | KMPKK063-19_ Rhiniinae |
| Rhiniinae | KMPKK064-19 | KMPKK064-19_ Rhiniinae |
| Rhiniinae | KMPKK065-19 | KMPKK065-19_ Rhiniinae |
| Rhiniinae | KMPKK066-19 | KMPKK066-19_ Rhiniinae |
| Rhiniinae | KMPKL079-19 | KMPKL079-19_ Rhiniinae |
| Rhiniinae | KMPKL080-19 | KMPKL080-19_ Rhiniinae |
| Rhiniinae | KMPKL081-19 | KMPKL081-19_ Rhiniinae |
| Rhiniinae | KMPKL082-19 | KMPKL082-19_ Rhiniinae |
| Rhiniinae | KMPKL083-19 | KMPKL083-19_ Rhiniinae |
| Rhiniinae | KMPKL084-19 | KMPKL084-19_ Rhiniinae |
| Rhiniinae | KMPKL085-19 | KMPKL085-19_ Rhiniinae |
| Rhiniinae | KMPKL086-19 | KMPKL086-19_ Rhiniinae |
| Rhiniinae | KMPKL087-19 | KMPKL087-19_ Rhiniinae |
| Rhiniinae | KMPKM029-19 | KMPKM029-19_ Rhiniinae |
| Rhiniinae | KMPKM030-19 | KMPKM030-19_ Rhiniinae |
| Rhiniinae | KMPKM033-19 | KMPKM033-19_ Rhiniinae |
| Rhiniinae | KMPKM035-19 | KMPKM035-19_ Rhiniinae |
| Rhiniinae | KMPKN134-19 | KMPKN134-19_ Rhiniinae |
| Rhiniinae | KMPKN137-19 | KMPKN137-19_ Rhiniinae |
| Rhiniinae | KMPKN142-19 | KMPKN142-19_ Rhiniinae |
| Rhiniinae | KMPKN147-19 | KMPKN147-19_ Rhiniinae |
| Rhiniinae | KMPKN150-19 | KMPKN150-19_ Rhiniinae |
| Rhiniinae | KMPKO189-19 | KMPKO189-19_ Rhiniinae |
| Rhiniinae | KMPKP665-19 | KMPKP665-19_ Rhiniinae |
| Rhiniinae | KMPLC105-18 | KMPLC105-18_ Rhiniinae |
| Rhiniinae | KMPLC317-18 | KMPLC317-18_ Rhiniinae |
| *Rhinia* | KMPLJ134-19 | KMPLJ134-19_ Rhinia |
| Rhiniinae | KMPLJ136-19 | KMPLJ136-19_ Rhiniinae |
| Rhiniinae | KMPLK071-19 | KMPLK071-19_ Rhiniinae |
| Rhiniinae | KMPLQ1635-19 | KMPLQ1635-19_ Rhiniinae |
| Rhiniinae | KMPLX033-19 | KMPLX033-19_ Rhiniinae |
| Rhiniinae | KMPMA102-18 | KMPMA102-18_ Rhiniinae |
| Rhiniinae | KMPMA115-18 | KMPMA115-18_ Rhiniinae |
| Rhiniinae | KMPMA184-18 | KMPMA184-18_ Rhiniinae |
| Rhiniinae | KMPMA186-18 | KMPMA186-18_ Rhiniinae |
| Rhiniinae | KMPMA187-18 | KMPMA187-18_ Rhiniinae |
| Rhiniinae | KMPMA196-18 | KMPMA196-18_ Rhiniinae |
| Rhiniinae | KMPMA198-18 | KMPMA198-18_ Rhiniinae |
| Rhiniinae | KMPMA223-18 | KMPMA223-18_ Rhiniinae |
| Rhiniinae | KMPMB382-18 | KMPMB382-18_ Rhiniinae |
| Rhiniinae | KMPMC036-18 | KMPMC036-18_ Rhiniinae |
| Rhiniinae | KMPMC251-18 | KMPMC251-18_ Rhiniinae |
| Rhiniinae | KMPMC259-18 | KMPMC259-18_ Rhiniinae |
| Rhiniinae | KMPMD012-18 | KMPMD012-18_ Rhiniinae |
| Rhiniinae | KMPMD071-18 | KMPMD071-18_ Rhiniinae |
| Rhiniinae | KMPMD159-18 | KMPMD159-18_ Rhiniinae |
| Rhiniinae | KMPMF080-19 | KMPMF080-19_ Rhiniinae |
| Rhiniinae | KMPMF081-19 | KMPMF081-19_ Rhiniinae |
| Rhiniinae | KMPMH070-19 | KMPMH070-19_ Rhiniinae |
| Rhiniinae | KMPMH072-19 | KMPMH072-19_ Rhiniinae |
| Rhiniinae | KMPML019-19 | KMPML019-19_ Rhiniinae |
| Rhiniinae | KMPML024-19 | KMPML024-19_ Rhiniinae |
| Rhiniinae | KMPMN584-19 | KMPMN584-19_ Rhiniinae |
| Rhiniinae | KMPMO095-19 | KMPMO095-19_ Rhiniinae |
| Rhiniinae | KMPMO098-19 | KMPMO098-19_ Rhiniinae |
| Rhiniinae | KMPMO119-19 | KMPMO119-19_ Rhiniinae |
| Rhiniinae | KMPMO129-19 | KMPMO129-19_ Rhiniinae |
| Rhiniinae | KMPMO134-19 | KMPMO134-19_ Rhiniinae |
| Rhiniinae | KMPMO135-19 | KMPMO135-19_ Rhiniinae |
| Rhiniinae | KMPMO227-19 | KMPMO227-19_ Rhiniinae |
| Rhiniinae | KMPMP121-19 | KMPMP121-19_ Rhiniinae |
| Rhiniinae | KMPMP123-19 | KMPMP123-19_ Rhiniinae |
| Rhiniinae | KMPMP125-19 | KMPMP125-19_ Rhiniinae |
| Rhiniinae | KMPMQ1717-19 | KMPMQ1717-19_ Rhiniinae |
| Rhiniinae | KMPMQ320-19 | KMPMQ320-19_ Rhiniinae |
| Rhiniinae | KMPMQ326-19 | KMPMQ326-19_ Rhiniinae |
| Rhiniinae | KMPMQ332-19 | KMPMQ332-19_ Rhiniinae |
| Rhiniinae | KMPMQ336-19 | KMPMQ336-19_ Rhiniinae |
| Rhiniinae | KMPMQ338-19 | KMPMQ338-19_ Rhiniinae |
| Rhiniinae | KMPMQ341-19 | KMPMQ341-19_ Rhiniinae |
| *Stegosoma vinculatum* | KMPMQ343-19 | KMPMQ343-19_ Stegosoma_vinculatum |
| *Rhyncomya soyauxi* | KMPMQ347-19 | KMPMQ347-19_ Rhyncomya_soyauxi |
| Rhiniinae | KMPMQ348-19 | KMPMQ348-19_ Rhiniinae |
| Rhiniinae | KMPMQ350-19 | KMPMQ350-19_ Rhiniinae |
| Rhiniinae | KMPMQ358-19 | KMPMQ358-19_ Rhiniinae |
| Rhiniinae | KMPMQ360-19 | KMPMQ360-19_ Rhiniinae |
| Rhiniinae | KMPMQ363-19 | KMPMQ363-19_ Rhiniinae |
| Rhiniinae | KMPMQ371-19 | KMPMQ371-19_ Rhiniinae |
| Rhiniinae | KMPMQ372-19 | KMPMQ372-19_ Rhiniinae |
| Rhiniinae | KMPMQ381-19 | KMPMQ381-19_ Rhiniinae |
| Rhiniinae | KMPMQ385-19 | KMPMQ385-19_ Rhiniinae |
| Rhiniinae | KMPMQ388-19 | KMPMQ388-19_ Rhiniinae |
| *Rhyncomya soyauxi* | KMPMQ390-19 | KMPMQ390-19_ Rhyncomya_soyauxi |
| Rhiniinae | KMPMQ394-19 | KMPMQ394-19_ Rhiniinae |
| Rhiniinae | KMPMQ397-19 | KMPMQ397-19_ Rhiniinae |
| Rhiniinae | KMPMQ398-19 | KMPMQ398-19_ Rhiniinae |
| Rhiniinae | KMPMQ406-19 | KMPMQ406-19_ Rhiniinae |
| Rhiniinae | KMPMQ411-19 | KMPMQ411-19_ Rhiniinae |
| Rhiniinae | KMPMQ412-19 | KMPMQ412-19_ Rhiniinae |
| Rhiniinae | KMPMQ418-19 | KMPMQ418-19_ Rhiniinae |
| Rhiniinae | KMPMQ422-19 | KMPMQ422-19_ Rhiniinae |
| Rhiniinae | KMPMQ423-19 | KMPMQ423-19_ Rhiniinae |
| Rhiniinae | KMPMQ427-19 | KMPMQ427-19_ Rhiniinae |
| Rhiniinae | KMPMQ438-19 | KMPMQ438-19_ Rhiniinae |
| Rhiniinae | KMPMQ451-19 | KMPMQ451-19_ Rhiniinae |
| Rhiniinae | KMPMQ461-19 | KMPMQ461-19_ Rhiniinae |
| Rhiniinae | KMPMQ466-19 | KMPMQ466-19_ Rhiniinae |
| Rhiniinae | KMPMQ475-19 | KMPMQ475-19_ Rhiniinae |
| Rhiniinae | KMPMQ481-19 | KMPMQ481-19_ Rhiniinae |
| Rhiniinae | KMPMQ496-19 | KMPMQ496-19_ Rhiniinae |
| Rhiniinae | KMPMS056-19 | KMPMS056-19_ Rhiniinae |
| Rhiniinae | KMPNA1081-18 | KMPNA1081-18_ Rhiniinae |
| Rhiniinae | KMPNA1090-18 | KMPNA1090-18_ Rhiniinae |
| Rhiniinae | KMPNA143-18 | KMPNA143-18_ Rhiniinae |
| Rhiniinae | KMPNA278-18 | KMPNA278-18_ Rhiniinae |
| Rhiniinae | KMPNA288-18 | KMPNA288-18_ Rhiniinae |
| *Rhyncomya soyauxi* | KMPNA345-18 | KMPNA345-18_ Rhyncomya_soyauxi |
| *Rhyncomya soyauxi* | KMPNB1132-18 | KMPNB1132-18_ Rhyncomya_soyauxi |
| Rhiniinae | KMPNB1134-18 | KMPNB1134-18_ Rhiniinae |
| Rhiniinae | KMPNB1200-18 | KMPNB1200-18_ Rhiniinae |
| Rhiniinae | KMPNB1275-18 | KMPNB1275-18_ Rhiniinae |
| Rhiniinae | KMPND457-18 | KMPND457-18_ Rhiniinae |
| Rhiniinae | KMPND461-18 | KMPND461-18_ Rhiniinae |
| Rhiniinae | KMPNG022-19 | KMPNG022-19_ Rhiniinae |
| Rhiniinae | KMPNH1260-19 | KMPNH1260-19_ Rhiniinae |
| Rhiniinae | KMPNH1261-19 | KMPNH1261-19_ Rhiniinae |
| Rhiniinae | KMPNH1262-19 | KMPNH1262-19_ Rhiniinae |
| Rhiniinae | KMPNH1268-19 | KMPNH1268-19_ Rhiniinae |
| Rhiniinae | KMPNH1272-19 | KMPNH1272-19_ Rhiniinae |
| Rhiniinae | KMPNH1275-19 | KMPNH1275-19_ Rhiniinae |
| Rhiniinae | KMPNI1426-19 | KMPNI1426-19_ Rhiniinae |
| Rhiniinae | KMPNI1440-19 | KMPNI1440-19_ Rhiniinae |
| Rhiniinae | KMPNI325-19 | KMPNI325-19_ Rhiniinae |
| Rhiniinae | KMPNI328-19 | KMPNI328-19_ Rhiniinae |
| Rhiniinae | KMPNI331-19 | KMPNI331-19_ Rhiniinae |
| Rhiniinae | KMPNJ009-19 | KMPNJ009-19_ Rhiniinae |
| Rhiniinae | KMPNJ036-19 | KMPNJ036-19_ Rhiniinae |
| Rhiniinae | KMPNJ042-19 | KMPNJ042-19_ Rhiniinae |
| Rhiniinae | KMPNJ046-19 | KMPNJ046-19_ Rhiniinae |
| Rhiniinae | KMPNJ049-19 | KMPNJ049-19_ Rhiniinae |
| Rhiniinae | KMPNJ059-19 | KMPNJ059-19_ Rhiniinae |
| Rhiniinae | KMPNJ1382-19 | KMPNJ1382-19_ Rhiniinae |
| Rhiniinae | KMPNK046-19 | KMPNK046-19_ Rhiniinae |
| Rhiniinae | KMPNK052-19 | KMPNK052-19_ Rhiniinae |
| Rhiniinae | KMPNK053-19 | KMPNK053-19_ Rhiniinae |
| Rhiniinae | KMPNK215-19 | KMPNK215-19_ Rhiniinae |
| Rhiniinae | KMPNK245-19 | KMPNK245-19_ Rhiniinae |
| Rhiniinae | KMPNK344-19 | KMPNK344-19_ Rhiniinae |
| Rhiniinae | KMPNL715-19 | KMPNL715-19_ Rhiniinae |
| Rhiniinae | KMPNL716-19 | KMPNL716-19_ Rhiniinae |
| Rhiniinae | KMPNL724-19 | KMPNL724-19_ Rhiniinae |
| Rhiniinae | KMPNL729-19 | KMPNL729-19_ Rhiniinae |
| Rhiniinae | KMPNL731-19 | KMPNL731-19_ Rhiniinae |
| Rhiniinae | KMPNL732-19 | KMPNL732-19_ Rhiniinae |
| Rhiniinae | KMPNL734-19 | KMPNL734-19_ Rhiniinae |
| Rhiniinae | KMPNL738-19 | KMPNL738-19_ Rhiniinae |
| Rhiniinae | KMPNM007-19 | KMPNM007-19_ Rhiniinae |
| Rhiniinae | KMPNM112-19 | KMPNM112-19_ Rhiniinae |
| Rhiniinae | KMPNM1244-19 | KMPNM1244-19_ Rhiniinae |
| Rhiniinae | KMPNM1249-19 | KMPNM1249-19_ Rhiniinae |
| Rhiniinae | KMPNM1250-19 | KMPNM1250-19_ Rhiniinae |
| Rhiniinae | KMPNM1253-19 | KMPNM1253-19_ Rhiniinae |
| Rhiniinae | KMPNM1254-19 | KMPNM1254-19_ Rhiniinae |
| Rhiniinae | KMPNM1255-19 | KMPNM1255-19_ Rhiniinae |
| Rhiniinae | KMPNM1256-19 | KMPNM1256-19_ Rhiniinae |
| Rhiniinae | KMPNM1257-19 | KMPNM1257-19_ Rhiniinae |
| Rhiniinae | KMPNM1261-19 | KMPNM1261-19_ Rhiniinae |
| Rhiniinae | KMPNM170-19 | KMPNM170-19_ Rhiniinae |
| Rhiniinae | KMPNM177-19 | KMPNM177-19_ Rhiniinae |
| Rhiniinae | KMPNO1136-19 | KMPNO1136-19_ Rhiniinae |
| Rhiniinae | KMPNO1398-19 | KMPNO1398-19_ Rhiniinae |
| Rhiniinae | KMPNO257-19 | KMPNO257-19_ Rhiniinae |
| Rhiniinae | KMPNO831-19 | KMPNO831-19_ Rhiniinae |
| *Rhyncomya soyauxi* | KMPNP1547-19 | KMPNP1547-19_ Rhyncomya_soyauxi |
| Rhiniinae | KMPNP1549-19 | KMPNP1549-19_ Rhiniinae |
| Rhiniinae | KMPNP1562-19 | KMPNP1562-19_ Rhiniinae |
| Rhiniinae | KMPNP1566-19 | KMPNP1566-19_ Rhiniinae |
| Rhiniinae | KMPNP1568-19 | KMPNP1568-19_ Rhiniinae |
| Rhiniinae | KMPNQ2515-19 | KMPNQ2515-19_ Rhiniinae |
| Rhiniinae | KMPNQ2523-19 | KMPNQ2523-19_ Rhiniinae |
| Rhiniinae | KMPNQ2547-19 | KMPNQ2547-19_ Rhiniinae |
| Rhiniinae | KMPNQ2561-19 | KMPNQ2561-19_ Rhiniinae |
| Rhiniinae | KMPNQ2565-19 | KMPNQ2565-19_ Rhiniinae |
| Rhiniinae | KMPNQ2577-19 | KMPNQ2577-19_ Rhiniinae |
| Rhiniinae | KMPNQ455-19 | KMPNQ455-19_ Rhiniinae |
| Rhiniinae | KMPNR091-19 | KMPNR091-19_ Rhiniinae |
| Rhiniinae | KMPNR097-19 | KMPNR097-19_ Rhiniinae |
| Rhiniinae | KMPNR103-19 | KMPNR103-19_ Rhiniinae |
| Rhiniinae | KMPNR659-19 | KMPNR659-19_ Rhiniinae |
| Rhiniinae | KMPNS1504-19 | KMPNS1504-19_ Rhiniinae |
| Rhiniinae | KMPNS1511-19 | KMPNS1511-19_ Rhiniinae |
| *Rhyncomya soyauxi* | KMPOE009-18 | KMPOE009-18_ Rhyncomya_soyauxi |
| Rhiniinae | KMPOG215-19 | KMPOG215-19_ Rhiniinae |
| Rhiniinae | KMPOI052-19 | KMPOI052-19_ Rhiniinae |
| Rhiniinae | KMPOK239-19 | KMPOK239-19_ Rhiniinae |
| Rhiniinae | KMPOK240-19 | KMPOK240-19_ Rhiniinae |
| Rhiniinae | KMPOL085-19 | KMPOL085-19_ Rhiniinae |
| Rhiniinae | KMPOL089-19 | KMPOL089-19_ Rhiniinae |
| Rhiniinae | KMPOM226-19 | KMPOM226-19_ Rhiniinae |
| Rhiniinae | KMPOO174-19 | KMPOO174-19_ Rhiniinae |
| Rhiniinae | KMPOP042-19 | KMPOP042-19_ Rhiniinae |
| Rhiniinae | KMPOP045-19 | KMPOP045-19_ Rhiniinae |
| Rhiniinae | KMPOR020-19 | KMPOR020-19_ Rhiniinae |
| Rhiniinae | KMPOR021-19 | KMPOR021-19_ Rhiniinae |
| Rhiniinae | KMPOS021-19 | KMPOS021-19_ Rhiniinae |
| Rhiniinae | KMPOS024-19 | KMPOS024-19_ Rhiniinae |
| Rhiniinae | KMPOS025-19 | KMPOS025-19_ Rhiniinae |
| Rhiniinae | KMPOS030-19 | KMPOS030-19_ Rhiniinae |
| Rhiniinae | KMPOS078-19 | KMPOS078-19_ Rhiniinae |
| Rhiniinae | KMPOT063-19 | KMPOT063-19_ Rhiniinae |
| Rhiniinae | KMPOT072-19 | KMPOT072-19_ Rhiniinae |
| Rhiniinae | KMPOT218-19 | KMPOT218-19_ Rhiniinae |
| Rhiniinae | KMPOX007-19 | KMPOX007-19_ Rhiniinae |
| *Rhyncomya soyauxi* | KMPOX008-19 | KMPOX008-19_ Rhyncomya_soyauxi |
| Rhiniinae | KMPOX009-19 | KMPOX009-19_ Rhiniinae |
| Rhiniinae | KMPOX015-19 | KMPOX015-19_ Rhiniinae |
| Rhiniinae | KMPPI1145-19 | KMPPI1145-19_ Rhiniinae |
| Rhiniinae | KMPQ1073-19 | KMPQ1073-19_ Rhiniinae |
| Rhiniinae | KMPQJ089-19 | KMPQJ089-19_ Rhiniinae |
| Rhiniinae | KMPQT025-19 | KMPQT025-19_ Rhiniinae |
| Rhiniinae | KMPRF129-19 | KMPRF129-19_ Rhiniinae |
| Rhiniinae | KMPRH082-19 | KMPRH082-19_ Rhiniinae |
| Rhiniinae | KMPRI160-19 | KMPRI160-19_ Rhiniinae |
| *Rhyncomya soyauxi* | KMPRK054-19 | KMPRK054-19_ Rhyncomya_soyauxi |
| Rhiniinae | KMPRL034-19 | KMPRL034-19_ Rhiniinae |
| Rhiniinae | KMPRL036-19 | KMPRL036-19_ Rhiniinae |
| Rhiniinae | KMPRL038-19 | KMPRL038-19_ Rhiniinae |
| Rhiniinae | KMPRM088-19 | KMPRM088-19_ Rhiniinae |
| Rhiniinae | KMPRM325-19 | KMPRM325-19_ Rhiniinae |
| Rhiniinae | KMPRO1095-19 | KMPRO1095-19_ Rhiniinae |
| Rhiniinae | KMPRO1099-19 | KMPRO1099-19_ Rhiniinae |
| Rhiniinae | KMPRO1100-19 | KMPRO1100-19_ Rhiniinae |
| Rhiniinae | KMPRO1101-19 | KMPRO1101-19_ Rhiniinae |
| Rhiniinae | KMPRO1102-19 | KMPRO1102-19_ Rhiniinae |
| Rhiniinae | KMPRO1114-19 | KMPRO1114-19_ Rhiniinae |
| Rhiniinae | KMPRO1126-19 | KMPRO1126-19_ Rhiniinae |
| Rhiniinae | KMPRO1130-19 | KMPRO1130-19_ Rhiniinae |
| Rhiniinae | KMPRO1131-19 | KMPRO1131-19_ Rhiniinae |
| Rhiniinae | KMPRO1140-19 | KMPRO1140-19_ Rhiniinae |
| Rhiniinae | KMPRO1142-19 | KMPRO1142-19_ Rhiniinae |
| Rhiniinae | KMPRO1169-19 | KMPRO1169-19_ Rhiniinae |
| Rhiniinae | KMPRO1178-19 | KMPRO1178-19_ Rhiniinae |
| Rhiniinae | KMPRO1191-19 | KMPRO1191-19_ Rhiniinae |
| Rhiniinae | KMPRO1192-19 | KMPRO1192-19_ Rhiniinae |
| Rhiniinae | KMPRO1200-19 | KMPRO1200-19_ Rhiniinae |
| Rhiniinae | KMPRO1208-19 | KMPRO1208-19_ Rhiniinae |
| Rhiniinae | KMPRO1209-19 | KMPRO1209-19_ Rhiniinae |
| Rhiniinae | KMPRO1210-19 | KMPRO1210-19_ Rhiniinae |
| Rhiniinae | KMPRT093-19 | KMPRT093-19_ Rhiniinae |
| Rhiniinae | KMPRU005-19 | KMPRU005-19_ Rhiniinae |
| Rhiniinae | KMPRV076-19 | KMPRV076-19_ Rhiniinae |
| Rhiniinae | KMPRV083-19 | KMPRV083-19_ Rhiniinae |
| Rhiniinae | KMPRV089-19 | KMPRV089-19_ Rhiniinae |
| Rhiniinae | KMPRV464-19 | KMPRV464-19_ Rhiniinae |
| Rhiniinae | KMPSD032-18 | KMPSD032-18_ Rhiniinae |
| *Rhyncomya soyauxi* | KMPSF185-19 | KMPSF185-19_ Rhyncomya_soyauxi |
| Rhiniinae | KMPSF186-19 | KMPSF186-19_ Rhiniinae |
| Rhiniinae | KMPSH308-19 | KMPSH308-19_ Rhiniinae |
| Rhiniinae | KMPSI076-19 | KMPSI076-19_ Rhiniinae |
| Rhiniinae | KMPSI193-19 | KMPSI193-19_ Rhiniinae |
| Rhiniinae | KMPSO042-19 | KMPSO042-19_ Rhiniinae |
| Rhiniinae | KMPSO515-19 | KMPSO515-19_ Rhiniinae |
| Rhiniinae | KMPSO562-19 | KMPSO562-19_ Rhiniinae |
| Rhiniinae | KMPSP2607-19 | KMPSP2607-19_ Rhiniinae |
| *Rhyncomya soyauxi* | KMPSP2616-19 | KMPSP2616-19_ Rhyncomya_soyauxi |
| Rhiniinae | KMPSP2639-19 | KMPSP2639-19_ Rhiniinae |
| Rhiniinae | KMPSP762-19 | KMPSP762-19_ Rhiniinae |
| Rhiniinae | KMPSP775-19 | KMPSP775-19_ Rhiniinae |
| Rhiniinae | KMPST066-19 | KMPST066-19_ Rhiniinae |
| Rhiniinae | KMPSU033-19 | KMPSU033-19_ Rhiniinae |
| Rhiniinae | KMPSU034-19 | KMPSU034-19_ Rhiniinae |
| Rhiniinae | KMPSV847-19 | KMPSV847-19_ Rhiniinae |
| Rhiniinae | KMPUA165-18 | KMPUA165-18_ Rhiniinae |
| *Rhyncomya soyauxi* | KMPUA168-18 | KMPUA168-18_ Rhyncomya_soyauxi |
| *Rhyncomya soyauxi* | KMPUB2732-18 | KMPUB2732-18_ Rhyncomya_soyauxi |
| Rhiniinae | KMPUB2740-18 | KMPUB2740-18_ Rhiniinae |
| Rhiniinae | KMPUC1609-18 | KMPUC1609-18_ Rhiniinae |
| Rhiniinae | KMPUC1622-18 | KMPUC1622-18_ Rhiniinae |
| Rhiniinae | KMPUC2832-18 | KMPUC2832-18_ Rhiniinae |
| Rhiniinae | KMPUC2839-18 | KMPUC2839-18_ Rhiniinae |
| *Rhyncomya soyauxi* | KMPUD107-19 | KMPUD107-19_ Rhyncomya_soyauxi |
| *Rhyncomya soyauxi* | KMPUD123-19 | KMPUD123-19_ Rhyncomya_soyauxi |
| *Rhyncomya soyauxi* | KMPUD130-19 | KMPUD130-19_ Rhyncomya_soyauxi |
| *Rhyncomya soyauxi* | KMPUD144-19 | KMPUD144-19_ Rhyncomya_soyauxi |
| *Rhyncomya soyauxi* | KMPUD148-19 | KMPUD148-19_ Rhyncomya_soyauxi |
| Rhiniinae | KMPUD1639-19 | KMPUD1639-19_ Rhiniinae |
| *Rhyncomya soyauxi* | KMPUD168-19 | KMPUD168-19_ Rhyncomya_soyauxi |
| *Rhyncomya soyauxi* | KMPUD211-19 | KMPUD211-19_ Rhyncomya_soyauxi |
| Rhiniinae | KMPUD2126-19 | KMPUD2126-19_ Rhiniinae |
| Rhiniinae | KMPUD232-19 | KMPUD232-19_ Rhiniinae |
| Rhiniinae | KMPUD236-19 | KMPUD236-19_ Rhiniinae |
| *Rhyncomya soyauxi* | KMPUD240-19 | KMPUD240-19_ Rhyncomya_soyauxi |
| Rhiniinae | KMPUD260-19 | KMPUD260-19_ Rhiniinae |
| Rhiniinae | KMPUD261-19 | KMPUD261-19_ Rhiniinae |
| *Rhyncomya soyauxi* | KMPUD266-19 | KMPUD266-19_ Rhyncomya_soyauxi |
| Rhiniinae | KMPUD2740-19 | KMPUD2740-19_ Rhiniinae |
| *Rhyncomya soyauxi* | KMPUD281-19 | KMPUD281-19_ Rhyncomya_soyauxi |
| *Rhyncomya soyauxi* | KMPUD309-19 | KMPUD309-19_ Rhyncomya_soyauxi |
| *Rhyncomya soyauxi* | KMPUD329-19 | KMPUD329-19_ Rhyncomya_soyauxi |
| *Rhyncomya soyauxi* | KMPUD339-19 | KMPUD339-19_ Rhyncomya_soyauxi |
| Rhiniinae | KMPUD349-19 | KMPUD349-19_ Rhiniinae |
| *Rhyncomya soyauxi* | KMPUD357-19 | KMPUD357-19_ Rhyncomya_soyauxi |
| Rhiniinae | KMPUD4445-19 | KMPUD4445-19_ Rhiniinae |
| Rhiniinae | KMPUD4446-19 | KMPUD4446-19_ Rhiniinae |
| Rhiniinae | KMPUD4497-19 | KMPUD4497-19_ Rhiniinae |
| Rhiniinae | KMPUD4511-19 | KMPUD4511-19_ Rhiniinae |
| Rhiniinae | KMPUD4516-19 | KMPUD4516-19_ Rhiniinae |
| *Rhyncomya soyauxi* | KMPUE617-19 | KMPUE617-19_ Rhyncomya_soyauxi |
| Rhiniinae | KMPUE627-19 | KMPUE627-19_ Rhiniinae |
| Rhiniinae | KMPUH069-19 | KMPUH069-19_ Rhiniinae |
| Rhiniinae | KMPUH204-19 | KMPUH204-19_ Rhiniinae |
| *Rhyncomya soyauxi* | KMPUH207-19 | KMPUH207-19_ Rhyncomya_soyauxi |
| Rhiniinae | KMPUH226-19 | KMPUH226-19_ Rhiniinae |
| Rhiniinae | KMPUH228-19 | KMPUH228-19_ Rhiniinae |
| Rhiniinae | KMPUH247-19 | KMPUH247-19_ Rhiniinae |
| Rhiniinae | KMPUH274-19 | KMPUH274-19_ Rhiniinae |
| Rhiniinae | KMPUH341-19 | KMPUH341-19_ Rhiniinae |
| Rhiniinae | KMPUI083-19 | KMPUI083-19_ Rhiniinae |
| Rhiniinae | KMPUI085-19 | KMPUI085-19_ Rhiniinae |
| Rhiniinae | KMPUI119-19 | KMPUI119-19_ Rhiniinae |
| Rhiniinae | KMPUI135-19 | KMPUI135-19_ Rhiniinae |
| *Rhyncomya soyauxi* | KMPUI163-19 | KMPUI163-19_ Rhyncomya_soyauxi |
| Rhiniinae | KMPUI166-19 | KMPUI166-19_ Rhiniinae |
| Rhiniinae | KMPUI178-19 | KMPUI178-19_ Rhiniinae |
| Rhiniinae | KMPUI198-19 | KMPUI198-19_ Rhiniinae |
| Rhiniinae | KMPUJ014-19 | KMPUJ014-19_ Rhiniinae |
| Rhiniinae | KMPUJ016-19 | KMPUJ016-19_ Rhiniinae |
| Rhiniinae | KMPUJ046-19 | KMPUJ046-19_ Rhiniinae |
| *Rhyncomya soyauxi* | KMPUJ101-19 | KMPUJ101-19_ Rhyncomya_soyauxi |
| Rhiniinae | KMPUJ129-19 | KMPUJ129-19_ Rhiniinae |
| *Rhyncomya soyauxi* | KMPUJ131-19 | KMPUJ131-19_ Rhyncomya_soyauxi |
| Rhiniinae | KMPUJ1366-19 | KMPUJ1366-19_ Rhiniinae |
| Rhiniinae | KMPUJ1413-19 | KMPUJ1413-19_ Rhiniinae |
| Rhiniinae | KMPUJ3505-19 | KMPUJ3505-19_ Rhiniinae |
| *Rhyncomya soyauxi* | KMPUJ477-19 | KMPUJ477-19_ Rhyncomya_soyauxi |
| Rhiniinae | KMPUJ478-19 | KMPUJ478-19_ Rhiniinae |
| *Rhyncomya soyauxi* | KMPUJ479-19 | KMPUJ479-19_ Rhyncomya_soyauxi |
| Rhiniinae | KMPUJ486-19 | KMPUJ486-19_ Rhiniinae |
| *Rhyncomya soyauxi* | KMPUJ490-19 | KMPUJ490-19_ Rhyncomya_soyauxi |
| Rhiniinae | KMPUJ557-19 | KMPUJ557-19_ Rhiniinae |
| Rhiniinae | KMPUJ578-19 | KMPUJ578-19_ Rhiniinae |
| Rhiniinae | KMPUJ612-19 | KMPUJ612-19_ Rhiniinae |
| Rhiniinae | KMPUJ618-19 | KMPUJ618-19_ Rhiniinae |
| Rhiniinae | KMPUJ6280-19 | KMPUJ6280-19_ Rhiniinae |
| Rhiniinae | KMPUJ831-19 | KMPUJ831-19_ Rhiniinae |
| Rhiniinae | KMPVA007-18 | KMPVA007-18_ Rhiniinae |
| Rhiniinae | KMPVA152-18 | KMPVA152-18_ Rhiniinae |
| Rhiniinae | KMPVH036-19 | KMPVH036-19_ Rhiniinae |
| Rhiniinae | KMPVJ250-19 | KMPVJ250-19_ Rhiniinae |
| Rhiniinae | KMPVV002-19 | KMPVV002-19_ Rhiniinae |
| Rhiniinae | KMPWA512-18 | KMPWA512-18_ Rhiniinae |
| *Rhyncomya soyauxi* | KMPWB072-18 | KMPWB072-18_ Rhyncomya_soyauxi |
| *Rhyncomya soyauxi* | KMPWC021-18 | KMPWC021-18_ Rhyncomya_soyauxi |
| Rhiniinae | KMPWG638-18 | KMPWG638-18_ Rhiniinae |
| Rhiniinae | KMPWL304-18 | KMPWL304-18_ Rhiniinae |
| Rhiniinae | KMPWL309-18 | KMPWL309-18_ Rhiniinae |
| Rhiniinae | KMPWL312-18 | KMPWL312-18_ Rhiniinae |
| Rhiniinae | KMPWM031-18 | KMPWM031-18_ Rhiniinae |
| Rhiniinae | KMPWN152-19 | KMPWN152-19_ Rhiniinae |
| Rhiniinae | KMPWN154-19 | KMPWN154-19_ Rhiniinae |
| Rhiniinae | KMPWO022-19 | KMPWO022-19_ Rhiniinae |
| Rhiniinae | KMPWO024-19 | KMPWO024-19_ Rhiniinae |
| Rhiniinae | KMPWO026-19 | KMPWO026-19_ Rhiniinae |
| Rhiniinae | KMPWO028-19 | KMPWO028-19_ Rhiniinae |
| Rhiniinae | KMPWO030-19 | KMPWO030-19_ Rhiniinae |
| Rhiniinae | KMPWO035-19 | KMPWO035-19_ Rhiniinae |
| Rhiniinae | KMPWO1317-19 | KMPWO1317-19_ Rhiniinae |
| Rhiniinae | KMPWO1556-19 | KMPWO1556-19_ Rhiniinae |
| Rhiniinae | KMPWR110-19 | KMPWR110-19_ Rhiniinae |
| Rhiniinae | KMPWR112-19 | KMPWR112-19_ Rhiniinae |
| Rhiniinae | KMPWR197-19 | KMPWR197-19_ Rhiniinae |
| Rhiniinae | KMPWS172-19 | KMPWS172-19_ Rhiniinae |
| *Rhyncomya soyauxi* | KMPWS186-19 | KMPWS186-19_ Rhyncomya_soyauxi |
| *Rhyncomya soyauxi* | KMPWT2205-19 | KMPWT2205-19_ Rhyncomya_soyauxi |
| Rhiniinae | KMPWT2208-19 | KMPWT2208-19_ Rhiniinae |
| Rhiniinae | KMPWT2210-19 | KMPWT2210-19_ Rhiniinae |
| Rhiniinae | KMPWT411-19 | KMPWT411-19_ Rhiniinae |
| Rhiniinae | KMPWU088-19 | KMPWU088-19_ Rhiniinae |
| Rhiniinae | KMPWU295-19 | KMPWU295-19_ Rhiniinae |
| *Rhyncomya soyauxi* | KMPXH076-19 | KMPXH076-19_ Rhyncomya_soyauxi |
| Rhiniinae | KMPXN1418-19 | KMPXN1418-19_ Rhiniinae |
| Rhiniinae | KMPXN208-19 | KMPXN208-19_ Rhiniinae |
| Rhiniinae | KMPXO359-19 | KMPXO359-19_ Rhiniinae |
| Rhiniinae | KMPXO361-19 | KMPXO361-19_ Rhiniinae |
| Rhiniinae | KMPXO372-19 | KMPXO372-19_ Rhiniinae |
| Rhiniinae | KMPXO373-19 | KMPXO373-19_ Rhiniinae |
| Rhiniinae | KMPXP1890-19 | KMPXP1890-19_ Rhiniinae |
| *Rhyncomya soyauxi* | KMPXP1899-19 | KMPXP1899-19_ Rhyncomya_soyauxi |
| *Rhyncomya soyauxi* | KMPXQ040-19 | KMPXQ040-19_ Rhyncomya_soyauxi |
| Rhiniinae | KMPXQ043-19 | KMPXQ043-19_ Rhiniinae |
| Rhiniinae | KMPXR029-19 | KMPXR029-19_ Rhiniinae |
| Rhiniinae | KMPXR061-19 | KMPXR061-19_ Rhiniinae |
| *Rhyncomya soyauxi* | KMPXS074-19 | KMPXS074-19_ Rhyncomya_soyauxi |
| Rhiniinae | KMPXS075-19 | KMPXS075-19_ Rhiniinae |
| Rhiniinae | KMPYI012-19 | KMPYI012-19_ Rhiniinae |
| Rhiniinae | KMPZB1214-19 | KMPZB1214-19_ Rhiniinae |
| Rhiniinae | KMPZB1217-19 | KMPZB1217-19_ Rhiniinae |
| Rhiniinae | KMPZB1226-19 | KMPZB1226-19_ Rhiniinae |
| Rhiniinae | KMPZB1230-19 | KMPZB1230-19_ Rhiniinae |
| Rhiniinae | KMPZB1232-19 | KMPZB1232-19_ Rhiniinae |
| Rhiniinae | KMPZB1234-19 | KMPZB1234-19_ Rhiniinae |
| Rhiniinae | KMPZB1236-19 | KMPZB1236-19_ Rhiniinae |
| Rhiniinae | KMPZB1261-19 | KMPZB1261-19_ Rhiniinae |
| Rhiniinae | KMPZB1264-19 | KMPZB1264-19_ Rhiniinae |
| Rhiniinae | KMPZB1270-19 | KMPZB1270-19_ Rhiniinae |
| Rhiniinae | KMPZB1283-19 | KMPZB1283-19_ Rhiniinae |
| Rhiniinae | KMPZB214-19 | KMPZB214-19_ Rhiniinae |
| Rhiniinae | KMPZC050-19 | KMPZC050-19_ Rhiniinae |
| Rhiniinae | KMPZC052-19 | KMPZC052-19_ Rhiniinae |
| Rhiniinae | KMPZC060-19 | KMPZC060-19_ Rhiniinae |
| Rhiniinae | KMPZE543-19 | KMPZE543-19_ Rhiniinae |
| Rhiniinae | KMPZE549-19 | KMPZE549-19_ Rhiniinae |
| Rhiniinae | KMPZE551-19 | KMPZE551-19_ Rhiniinae |
| Rhiniinae | KMPZE553-19 | KMPZE553-19_ Rhiniinae |
| Rhiniinae | KMTTB423-18 | KMTTB423-18_ Rhiniinae |
| Rhiniinae | KMTTC030-18 | KMTTC030-18_ Rhiniinae |
| Rhiniinae | KMTTF027-19 | KMTTF027-19_ Rhiniinae |
| Rhiniinae | KMTTG130-19 | KMTTG130-19_ Rhiniinae |
| Rhiniinae | KMTTH809-19 | KMTTH809-19_ Rhiniinae |
| Rhiniinae | KMTTH817-19 | KMTTH817-19_ Rhiniinae |
| Rhiniinae | KMTTH825-19 | KMTTH825-19_ Rhiniinae |
| Rhiniinae | KMTTI015-19 | KMTTI015-19_ Rhiniinae |
| *Rhinia* | KMTTJ070-19 | KMTTJ070-19_ Rhinia |
| Rhiniinae | KMTTL904-19 | KMTTL904-19_ Rhiniinae |
| Rhiniinae | KMTTL906-19 | KMTTL906-19_ Rhiniinae |
| Rhiniinae | KVIS016-21 | KVIS016-21_ Rhiniinae |
| Rhiniinae | KVIS021-21 | KVIS021-21_ Rhiniinae |
| Rhiniinae | MADIP048-10 | MADIP048-10_ Rhiniinae |
| Rhiniinae | MADIP049-10 | MADIP049-10_ Rhiniinae |
| Rhiniinae | MADIP050-10 | MADIP050-10_ Rhiniinae |
| Rhiniinae | MADIP051-10 | MADIP051-10_ Rhiniinae |
| Rhiniinae | MADIP113-10 | MADIP113-10_ Rhiniinae |
| Rhiniinae | MADIP1269-13 | MADIP1269-13_ Rhiniinae |
| Rhiniinae | MADIP567-12 | MADIP567-12_ Rhiniinae |
| Rhiniinae | MADIP655-12 | MADIP655-12_ Rhiniinae |
| Rhiniinae | MBPT074-15 | MBPT074-15_ Rhiniinae |
| *Stomorhina discolor* | NSWHO3428-18 | NSWHO3428-18_ Stomorhina_discolor |
| *Stomorhina discolor* | NSWHO3438-18 | NSWHO3438-18_ Stomorhina_discolor |
| Rhiniinae | PLABN218-19 | PLABN218-19_ Rhiniinae |
| Rhiniinae | PLLBG1418-20 | PLLBG1418-20_ Rhiniinae |
| Rhiniinae | PLRCH080-20 | PLRCH080-20_ Rhiniinae |
| Rhiniinae | SAFRA1138-18 | SAFRA1138-18_ Rhiniinae |
| Rhiniinae | SAFRA2206-18 | SAFRA2206-18_ Rhiniinae |
| Rhiniinae | SAFRA2207-18 | SAFRA2207-18_ Rhiniinae |
| Rhiniinae | SAFRA2211-18 | SAFRA2211-18_ Rhiniinae |
| Rhiniinae | SAFRA2212-18 | SAFRA2212-18_ Rhiniinae |
| Rhiniinae | SAFRA2214-18 | SAFRA2214-18_ Rhiniinae |
| Rhiniinae | SAFRA2215-18 | SAFRA2215-18_ Rhiniinae |
| Rhiniinae | SAFRA2220-18 | SAFRA2220-18_ Rhiniinae |
| Rhiniinae | SAFRA2230-18 | SAFRA2230-18_ Rhiniinae |
| Rhiniinae | SAFRA2243-18 | SAFRA2243-18_ Rhiniinae |
| Rhiniinae | SAFRA2256-18 | SAFRA2256-18_ Rhiniinae |
| Rhiniinae | SAFRA3346-18 | SAFRA3346-18_ Rhiniinae |
| Rhiniinae | SAFRA3350-18 | SAFRA3350-18_ Rhiniinae |
| *Stomorhina discolor* | UKMBB045-13 | UKMBB045-13_ Stomorhina_discolor |
