# Supplementary material for: DNA barcoding of Afrotropical nose flies (Diptera, Calliphoridae, Rhiniinae): species identification, female-male morphotype association, and reference library development
Source: Zookeys. 2026 Jul 3;1284:149–83. doi: 10.3897/zookeys.1284.189450 (PMC13354976; doi:10.3897/zookeys.1284.189450)
Supplement: Supplementary material 8 — Results of ASAP with Distance K80_Kimura and ABGD with Distance K80 Kimura [file zookeys-1284-149_article-189450__-s008.docx]

**Suppl. Material 8** – Results of ASAP with Distance K80_Kimura and ABGD with Distance K80 Kimura / MinSlope = 0.900000 for a dataset of 216 COI DNA barcode sequences of Rhiniinae species from the Afrotropical region. For the ABGD  analysis (Model = K80), partitions five and six proposed 59 species groups (p = 0.00774–0.0129, BG = 0.019 %), and partition four proposed 78 species groups (p = 0.00464, BG = 0.007 %); and the ASAP analysis (Model = K80; ASAP score = 2.50) resulted in 62 species groups.

**- ASAP –**

20250226_Rhiniinae_216_MF_out_groups.fas.res.cvs

| Partition rank | NbSubset | Asap score | p-val | pval-rank | W | W rank | Treshold distance |
| --- | --- | --- | --- | --- | --- | --- | --- |
| 1 | 62 | 2.5 | 2.93E-03 | 1 | 0.00011 | 4 | 0.017023 |
| 2 | 70 | 7 | 1.16E-01 | 7 | 0.000102 | 7 | 0.011976 |
| 3 | 72 | 10 | 2.66E-02 | 3 | 0.00006 | 17 | 0.009248 |
| 4 | 66 | 11 | 5.45E-01 | 21 | 0.00014 | 1 | 0.013724 |
| 5 | 68 | 14.5 | 5.93E-01 | 24 | 0.000108 | 5 | 0.012235 |
| 6 | 64 | 15 | 5.63E-01 | 22 | 0.000095 | 8 | 0.01548 |
| 7 | 61 | 20.5 | 1.38E-01 | 10 | 0.000042 | 31 | 0.017778 |
| 8 | 59 | 21.5 | 4.25E-02 | 4 | 0.00003 | 39 | 0.019412 |
| 9 | 76 | 21.5 | 8.22E-01 | 30 | 0.00008 | 13 | 0.007925 |
| 10 | 67 | 22 | 8.70E-01 | 33 | 0.000085 | 11 | 0.012985 |
| 11 | 60 | 22.5 | 3.13E-01 | 13 | 0.00004 | 32 | 0.018602 |
| 12 | 71 | 23 | 9.44E-01 | 44 | 0.000113 | 2 | 0.010524 |
| 13 | 95 | 24.5 | 5.01E-01 | 19 | 0.000047 | 30 | 0.003036 |
| 14 | 69 | 24.5 | 9.30E-01 | 43 | 0.000102 | 6 | 0.012206 |
| 15 | 65 | 25 | 9.54E-01 | 47 | 0.000112 | 3 | 0.014514 |
| 16 | 58 | 26.5 | 3.99E-01 | 15 | 0.00003 | 38 | 0.019985 |
| 17 | 57 | 27 | 4.47E-01 | 17 | 0.000033 | 37 | 0.020058 |
| 18 | 54 | 27 | 4.67E-01 | 18 | 0.000036 | 36 | 0.024877 |
| 19 | 88 | 27 | 6.09E-01 | 25 | 0.000047 | 29 | 0.003242 |
| 20 | 94 | 27.5 | 6.73E-01 | 27 | 0.000047 | 28 | 0.003047 |
| 21 | 99 | 27.5 | 7.80E-01 | 28 | 0.000047 | 27 | 0.003032 |
| 22 | 51 | 27.5 | 9.22E-01 | 41 | 0.000064 | 14 | 0.028539 |
| 23 | 105 | 28 | 3.79E-01 | 14 | 0.000024 | 42 | 0.002336 |
| 24 | 52 | 28 | 9.00E-01 | 37 | 0.00005 | 19 | 0.026141 |
| 25 | 87 | 28.5 | 8.36E-01 | 31 | 0.000047 | 26 | 0.003266 |
| 26 | 82 | 29.5 | 1.30E-01 | 9 | 0.000018 | 50 | 0.004607 |
| 27 | 112 | 29.5 | 2.75E-01 | 12 | 0.000023 | 47 | 0.001515 |
| 28 | 92 | 29.5 | 8.82E-01 | 34 | 0.000047 | 25 | 0.003142 |
| 29 | 50 | 29.5 | 9.58E-01 | 49 | 0.000089 | 10 | 0.032393 |
| 30 | 46 | 30 | 7.81E-02 | 5 | 0.000017 | 55 | 0.038899 |
| 31 | 73 | 30.5 | 9.50E-01 | 45 | 0.00006 | 16 | 0.009168 |
| 32 | 47 | 31 | 1.28E-01 | 8 | 0.000017 | 54 | 0.038784 |
| 33 | 90 | 31.5 | 9.14E-01 | 39 | 0.000047 | 24 | 0.003223 |
| 34 | 75 | 31.5 | 9.56E-01 | 48 | 0.00006 | 15 | 0.008622 |
| 35 | 63 | 33 | 9.90E-01 | 57 | 0.000091 | 9 | 0.016244 |
| 36 | 86 | 33.5 | 2.38E-01 | 11 | 0.000016 | 56 | 0.003917 |
| 37 | 49 | 34 | 5.05E-01 | 20 | 0.000019 | 48 | 0.035735 |
| 38 | 55 | 34.5 | 7.96E-01 | 29 | 0.00003 | 40 | 0.023042 |
| 39 | 81 | 36 | 5.73E-01 | 23 | 0.000018 | 49 | 0.004717 |
| 40 | 108 | 36 | 6.35E-01 | 26 | 0.000023 | 46 | 0.001537 |
| 41 | 131 | 37 | 1.10E-02 | 2 | 0.000006 | 72 | 0.000757 |
| 42 | 56 | 41.5 | 9.26E-01 | 42 | 0.000026 | 41 | 0.020806 |
| 43 | 109 | 42.5 | 9.18E-01 | 40 | 0.000023 | 45 | 0.001522 |
| 44 | 22 | 43 | 9.76E-02 | 6 | 0.000004 | 80 | 0.057752 |
| 45 | 40 | 43.5 | 8.88E-01 | 35 | 0.000017 | 52 | 0.043051 |
| 46 | 45 | 47.5 | 9.00E-01 | 36 | 0.000014 | 59 | 0.03951 |
| 47 | 83 | 48 | 8.38E-01 | 32 | 0.000011 | 64 | 0.004554 |
| 48 | 44 | 48 | 9.14E-01 | 38 | 0.000014 | 58 | 0.04033 |
| 49 | 20 | 50.5 | 4.45E-01 | 16 | 0.000004 | 85 | 0.058608 |
| 50 | 48 | 51.5 | 9.78E-01 | 50 | 0.000017 | 53 | 0.038121 |
| 51 | 77 | 51.5 | 1.00E+00 | 91 | 0.00008 | 12 | 0.00767 |
| 52 | 43 | 54 | 9.52E-01 | 46 | 0.000012 | 62 | 0.041065 |
| 53 | 53 | 54 | 1.00E+00 | 90 | 0.00005 | 18 | 0.025588 |
| 54 | 38 | 54.5 | 9.80E-01 | 52 | 0.000015 | 57 | 0.04447 |
| 55 | 39 | 58 | 9.96E-01 | 65 | 0.000017 | 51 | 0.044106 |
| 56 | 36 | 58.5 | 9.80E-01 | 51 | 0.000011 | 66 | 0.048245 |
| 57 | 35 | 59 | 9.86E-01 | 53 | 0.000011 | 65 | 0.049124 |
| 58 | 37 | 59 | 9.88E-01 | 55 | 0.000012 | 63 | 0.046288 |
| 59 | 89 | 59 | 1.00E+00 | 95 | 0.000047 | 23 | 0.00323 |
| 60 | 91 | 59 | 1.00E+00 | 96 | 0.000047 | 22 | 0.003216 |
| 61 | 93 | 59 | 1.00E+00 | 97 | 0.000047 | 21 | 0.003066 |
| 62 | 96 | 59 | 1.00E+00 | 98 | 0.000047 | 20 | 0.003034 |
| 63 | 41 | 60.5 | 9.94E-01 | 60 | 0.000012 | 61 | 0.042123 |
| 64 | 78 | 62.5 | 1.00E+00 | 92 | 0.00004 | 33 | 0.006916 |
| 65 | 79 | 64 | 1.00E+00 | 93 | 0.000036 | 35 | 0.006178 |
| 66 | 80 | 64 | 1.00E+00 | 94 | 0.000036 | 34 | 0.005453 |
| 67 | 27 | 67 | 9.94E-01 | 59 | 0.000005 | 75 | 0.055489 |
| 68 | 30 | 67 | 9.96E-01 | 64 | 0.000007 | 70 | 0.052665 |
| 69 | 26 | 70 | 9.96E-01 | 63 | 0.000004 | 77 | 0.056163 |
| 70 | 24 | 70.5 | 9.96E-01 | 62 | 0.000004 | 79 | 0.057053 |
| 71 | 106 | 71.5 | 1.00E+00 | 99 | 0.000023 | 44 | 0.001595 |
| 72 | 107 | 71.5 | 1.00E+00 | 100 | 0.000023 | 43 | 0.001548 |
| 73 | 31 | 73.5 | 9.98E-01 | 76 | 0.000007 | 71 | 0.052088 |
| 74 | 16 | 74 | 9.96E-01 | 61 | 0.000004 | 87 | 0.059275 |
| 75 | 28 | 74.5 | 9.98E-01 | 75 | 0.000005 | 74 | 0.055364 |
| 76 | 42 | 74.5 | 1.00E+00 | 89 | 0.000012 | 60 | 0.041799 |
| 77 | 2 | 75.5 | 9.88E-01 | 54 | 0.000002 | 97 | 0.08674 |
| 78 | 32 | 77 | 1.00E+00 | 86 | 0.000009 | 68 | 0.051411 |
| 79 | 34 | 77.5 | 1.00E+00 | 88 | 0.000011 | 67 | 0.049979 |
| 80 | 33 | 78 | 1.00E+00 | 87 | 0.000009 | 69 | 0.050732 |
| 81 | 5 | 78.5 | 9.90E-01 | 56 | 0.000002 | 101 | 0.076045 |
| 82 | 4 | 79 | 9.92E-01 | 58 | 0.000002 | 100 | 0.081418 |
| 83 | 29 | 79 | 1.00E+00 | 85 | 0.000005 | 73 | 0.054081 |
| 84 | 23 | 79.5 | 1.00E+00 | 83 | 0.000004 | 76 | 0.057226 |
| 85 | 17 | 80 | 9.98E-01 | 74 | 0.000004 | 86 | 0.05896 |
| 86 | 15 | 80.5 | 9.98E-01 | 73 | 0.000003 | 88 | 0.059761 |
| 87 | 25 | 81 | 1.00E+00 | 84 | 0.000004 | 78 | 0.056853 |
| 88 | 12 | 81.5 | 9.98E-01 | 72 | 0.000003 | 91 | 0.061209 |
| 89 | 11 | 81.5 | 9.98E-01 | 71 | 0.000002 | 92 | 0.063351 |
| 90 | 10 | 81.5 | 9.98E-01 | 70 | 0.000002 | 93 | 0.065753 |
| 91 | 9 | 81.5 | 9.98E-01 | 69 | 0.000002 | 94 | 0.067187 |
| 92 | 7 | 82 | 9.98E-01 | 68 | 0.000002 | 96 | 0.070507 |
| 93 | 3 | 82 | 9.98E-01 | 66 | 0.000002 | 98 | 0.085379 |
| 94 | 21 | 82 | 1.00E+00 | 82 | 0.000004 | 82 | 0.058396 |
| 95 | 19 | 82 | 1.00E+00 | 81 | 0.000004 | 83 | 0.058637 |
| 96 | 18 | 82 | 1.00E+00 | 80 | 0.000004 | 84 | 0.058748 |
| 97 | 6 | 83 | 9.98E-01 | 67 | 0.000002 | 99 | 0.07222 |
| 98 | 14 | 84 | 1.00E+00 | 79 | 0.000003 | 89 | 0.060207 |
| 99 | 13 | 84 | 1.00E+00 | 78 | 0.000003 | 90 | 0.060419 |
| 100 | 8 | 86 | 1.00E+00 | 77 | 0.000002 | 95 | 0.069014 |
| 101 | 216 | 91 | 1.00E+00 | 101 | 0.000004 | 81 | 0 |

20250226_Rhiniinae_216_MF_out_groups.fas.scores.png


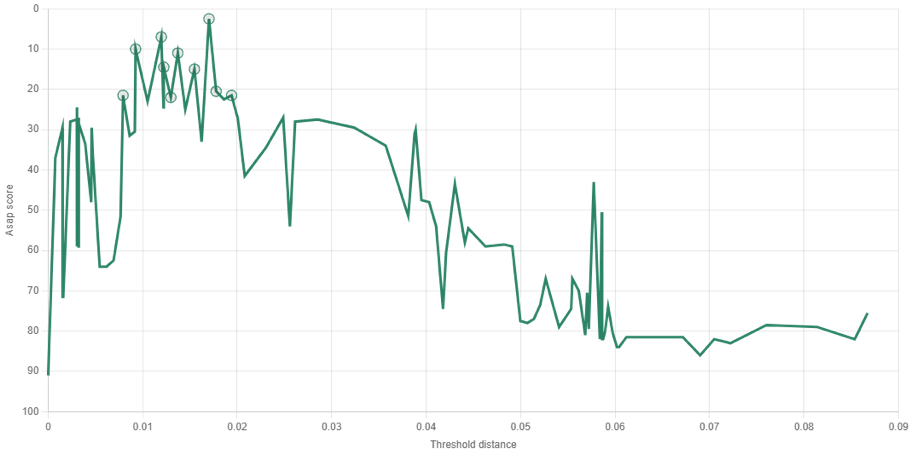


begin spart;

Project_name = 20250226_Rhiniinae_216_MF_out_groups.fas;

Date = 2025-03-13T14:08:23;

N_spartitions = 10 : 20250226_Rhiniinae_216_MF_out_groups.fas_asap_1,21.5 / 20250226_Rhiniinae_216_MF_out_groups.fas_asap_2,10.0 / 20250226_Rhiniinae_216_MF_out_groups.fas_asap_3,7.0 / 20250226_Rhiniinae_216_MF_out_groups.fas_asap_4,14.5 / 20250226_Rhiniinae_216_MF_out_groups.fas_asap_5,22.0 / 20250226_Rhiniinae_216_MF_out_groups.fas_asap_6,11.0 / 20250226_Rhiniinae_216_MF_out_groups.fas_asap_7,15.0 / 20250226_Rhiniinae_216_MF_out_groups.fas_asap_8,2.5 / 20250226_Rhiniinae_216_MF_out_groups.fas_asap_9,20.5 / 20250226_Rhiniinae_216_MF_out_groups.fas_asap_10,21.5;

N_individuals = 216 / 216 / 216 / 216 / 216 / 216 / 216 / 216 / 216 / 216;

N_subsets = 76 : 4.36e-01,8.39e-01,?,?,?,?,9.06e-01,?,?,8.37e-01,?,?,2.39e-01,?,?,?,1.29e-01,?,4.84e-01,8.00e-01,?,?,?,?,?,?,?,?,?,?,?,?,6.73e-01,?,?,?,?,6.35e-01,?,?,?,6.10e-01,?,?,?,?,9.14e-01,?,?,?,?,?,5.97e-01,?,?,?,?,5.72e-01,?,?,7.09e-01,?,5.02e-01,?,?,?,?,?,?,?,?,?,?,?,?,? / 72 : 4.36e-01,?,8.39e-01,?,9.36e-01,?,?,9.06e-01,?,?,8.37e-01,?,?,2.39e-01,?,?,?,1.29e-01,?,4.84e-01,8.00e-01,?,?,?,?,?,?,?,?,?,?,?,6.73e-01,?,?,?,?,6.35e-01,?,?,?,6.10e-01,?,?,?,?,9.14e-01,?,?,?,?,5.97e-01,?,?,?,9.50e-01,5.72e-01,?,?,7.09e-01,5.02e-01,8.23e-01,?,?,?,?,?,?,?,?,?,? / 70 : 2.65e-02,8.39e-01,?,9.36e-01,?,?,9.06e-01,?,?,8.37e-01,?,?,2.39e-01,?,?,?,1.29e-01,?,4.84e-01,8.00e-01,?,?,?,?,?,?,?,?,?,?,?,6.73e-01,?,?,?,?,6.35e-01,?,?,?,6.10e-01,?,?,?,?,9.14e-01,?,?,?,?,5.97e-01,?,?,?,9.44e-01,5.72e-01,?,?,7.09e-01,5.02e-01,8.23e-01,?,?,?,?,?,?,?,?,? / 68 : 2.65e-02,8.39e-01,?,?,9.30e-01,?,?,9.06e-01,?,8.37e-01,?,2.39e-01,?,?,?,1.29e-01,?,4.84e-01,8.00e-01,?,?,?,?,?,?,?,?,?,?,?,6.73e-01,?,?,?,?,6.35e-01,?,?,?,6.10e-01,?,?,?,?,9.14e-01,?,?,?,1.16e-01,?,?,?,9.44e-01,5.72e-01,?,?,7.09e-01,5.02e-01,8.23e-01,?,?,?,?,?,?,?,?,? / 67 : 2.65e-02,5.92e-01,?,9.30e-01,?,?,9.06e-01,?,8.37e-01,?,2.39e-01,?,?,?,1.29e-01,?,4.84e-01,8.00e-01,?,?,?,?,?,?,?,?,?,?,?,6.73e-01,?,?,?,?,6.35e-01,?,?,?,6.10e-01,?,?,?,?,9.14e-01,?,?,?,1.16e-01,?,?,?,9.44e-01,5.72e-01,?,?,7.09e-01,5.02e-01,8.23e-01,?,?,?,?,?,?,?,?,? / 66 : 2.65e-02,5.92e-01,?,9.30e-01,?,?,9.06e-01,?,8.37e-01,?,2.39e-01,?,?,?,1.29e-01,?,4.84e-01,8.00e-01,?,?,?,?,?,?,?,?,?,?,?,6.73e-01,?,?,?,?,?,6.35e-01,?,?,6.10e-01,?,?,?,?,9.14e-01,?,?,?,1.16e-01,?,?,?,9.44e-01,5.72e-01,?,?,7.09e-01,5.02e-01,8.71e-01,?,?,?,?,?,?,?,? / 64 : 2.65e-02,5.92e-01,?,9.54e-01,?,9.06e-01,?,8.37e-01,?,2.39e-01,?,?,?,1.29e-01,?,4.84e-01,8.00e-01,?,?,?,?,?,?,?,?,?,?,?,6.73e-01,?,?,?,5.46e-01,6.35e-01,?,?,6.10e-01,?,?,?,?,9.14e-01,?,?,?,1.16e-01,?,?,?,9.44e-01,5.72e-01,?,?,7.09e-01,5.02e-01,8.71e-01,?,?,?,?,?,?,?,? / 62 : 2.65e-02,5.92e-01,?,9.90e-01,?,9.06e-01,?,8.37e-01,?,2.39e-01,?,1.29e-01,?,?,4.84e-01,8.00e-01,?,?,?,?,?,?,?,?,?,?,?,6.73e-01,?,?,?,5.64e-01,?,?,6.10e-01,?,?,?,?,9.14e-01,?,?,?,1.16e-01,?,?,?,9.44e-01,5.72e-01,?,?,7.09e-01,5.02e-01,8.71e-01,?,?,?,?,?,?,?,? / 61 : 2.65e-02,5.92e-01,?,9.90e-01,?,9.06e-01,?,8.37e-01,?,2.39e-01,2.93e-03,?,?,4.84e-01,8.00e-01,?,?,?,?,?,?,?,?,?,?,?,6.73e-01,?,?,?,5.64e-01,?,?,6.10e-01,?,?,?,?,9.14e-01,?,?,?,1.16e-01,?,?,?,9.44e-01,5.72e-01,?,?,7.09e-01,5.02e-01,8.71e-01,?,?,?,?,?,?,?,? / 59 : 2.65e-02,9.06e-01,3.13e-01,?,?,?,8.37e-01,?,2.39e-01,2.93e-03,?,?,4.84e-01,8.00e-01,?,?,?,?,?,?,?,?,?,?,?,6.73e-01,?,?,?,5.64e-01,?,?,6.10e-01,?,?,?,?,1.37e-01,?,?,1.16e-01,?,?,?,9.44e-01,5.72e-01,?,?,7.09e-01,5.02e-01,8.71e-01,?,?,?,?,?,?,?, ? ;

[Generated by ASAP with Distance K80_Kimura ]

[WARNING: The sample names below may have been changed to fit SPART specification (only alphanumeric characters and _ )]

[Subset scores are p-values; see Puillandre et al. 2021 for details of the algorithm]

| N_spartitions (10) | fas_asap_1 | fas_asap_2 | fas_asap_3 | fas_asap_4 | fas_asap_5 | fas_asap_6 | fas_asap_7 | fas_asap_8 | fas_asap_9 | fas_asap_10 |
| --- | --- | --- | --- | --- | --- | --- | --- | --- | --- | --- |
| Spartition_score_type, Asap-Score | 21.5 | 10 | 7 | 14.5 | 22 | 11 | 15 | 2.5 | 20.5 | 21.5 |
| N_individuals | 216 | 216 | 216 | 216 | 216 | 216 | 216 | 216 | 216 | 216 |
| N_subsets | 76 | 72 | 70 | 68 | 67 | 66 | 64 | 62 | 61 | 59 |
| p-value | 8.22E-01 | 2.66E-02 | 1.16E-01 | 5.93E-01 | 8.70E-01 | 5.45E-01 | 5.63E-01 | 2.93E-03 | 1.38E-01 | 4.25E-02 |
| 1279A01_Stomorhina_cribrata_F_Ghana | 1 | 1 | 1 | 1 | 1 | 1 | 1 | 1 | 1 | 1 |
| 1279C06_Stomorhina_cribrata_M_Togo | 1 | 1 | 1 | 1 | 1 | 1 | 1 | 1 | 1 | 1 |
| 1279D01_Stomorhina_cribrata_F_Togo | 1 | 1 | 1 | 1 | 1 | 1 | 1 | 1 | 1 | 1 |
| 1279D02_Stomorhina_cribrata_M_Togo | 1 | 1 | 1 | 1 | 1 | 1 | 1 | 1 | 1 | 1 |
| 1282C02_Stomorhina_cribrata_M_SouthAfrica | 1 | 1 | 1 | 1 | 1 | 1 | 1 | 1 | 1 | 1 |
| 1306B06_Stomorhina_cribrata_F_SouthAfrica | 1 | 1 | 1 | 1 | 1 | 1 | 1 | 1 | 1 | 1 |
| 1306C03_Stomorhina_cribrata_M_SouthAfrica | 1 | 1 | 1 | 1 | 1 | 1 | 1 | 1 | 1 | 1 |
| 1306E02_Stomorhina_cribrata_F_SouthAfrica | 1 | 1 | 1 | 1 | 1 | 1 | 1 | 1 | 1 | 1 |
| 1306F06_Stomorhina_cribrata_F_SouthAfrica | 1 | 1 | 1 | 1 | 1 | 1 | 1 | 1 | 1 | 1 |
| 1306F07_Stomorhina_cribrata_F_SouthAfrica | 1 | 1 | 1 | 1 | 1 | 1 | 1 | 1 | 1 | 1 |
| 1306F08_Stomorhina_cribrata_F_SouthAfrica | 1 | 1 | 1 | 1 | 1 | 1 | 1 | 1 | 1 | 1 |
| S45_Stomorhina_cribrata_M_Tanzania | 1 | 1 | 1 | 1 | 1 | 1 | 1 | 1 | 1 | 1 |
| 1279E01_Stomorhina_cribrata_F_Togo | 1 | 1 | 1 | 1 | 1 | 1 | 1 | 1 | 1 | 1 |
| 1306F05_Stomorhina_cribrata_F_SouthAfrica | 2 | 2 | 1 | 1 | 1 | 1 | 1 | 1 | 1 | 1 |
| 1279D05_Stomorhina_rugosa_M_Togo | 3 | 3 | 2 | 2 | 2 | 2 | 2 | 2 | 2 | 2 |
| S13_Stomorhina_rugosa_M_Malawi | 3 | 3 | 2 | 2 | 2 | 2 | 2 | 2 | 2 | 2 |
| 1282D01_Stomorhina_rugosa_M_SouthAfrica | 3 | 3 | 2 | 2 | 2 | 2 | 2 | 2 | 2 | 2 |
| 1282D03_Stomorhina_rugosa_F_SouthAfrica | 3 | 3 | 2 | 2 | 2 | 2 | 2 | 2 | 2 | 2 |
| S14_Stomorhina_rugosa_F_Ethiopia | 3 | 3 | 2 | 2 | 2 | 2 | 2 | 2 | 2 | 2 |
| 1282A01_Stomorhina_lunata_M_SouthAfrica | 4 | 4 | 3 | 3 | 3 | 3 | 3 | 3 | 3 | 3 |
| 1282C01_Stomorhina_lunata_M_SouthAfrica | 4 | 4 | 3 | 3 | 3 | 3 | 3 | 3 | 3 | 3 |
| 1282C03_Stomorhina_lunata_M_SouthAfrica | 4 | 4 | 3 | 3 | 3 | 3 | 3 | 3 | 3 | 3 |
| 1282C08_Stomorhina_lunata_F_SouthAfrica | 4 | 4 | 3 | 3 | 3 | 3 | 3 | 3 | 3 | 3 |
| 1282D02_Stomorhina_lunata_M_SouthAfrica | 4 | 4 | 3 | 3 | 3 | 3 | 3 | 3 | 3 | 3 |
| 1299A02_Stomorhina_lunata_F_SouthAfrica | 4 | 4 | 3 | 3 | 3 | 3 | 3 | 3 | 3 | 3 |
| 1299A03_Stomorhina_lunata_F_SouthAfrica | 4 | 4 | 3 | 3 | 3 | 3 | 3 | 3 | 3 | 3 |
| 1306A01_Stomorhina_lunata_F_SouthAfrica | 4 | 4 | 3 | 3 | 3 | 3 | 3 | 3 | 3 | 3 |
| 1306C01_Stomorhina_lunata_M_SouthAfrica | 4 | 4 | 3 | 3 | 3 | 3 | 3 | 3 | 3 | 3 |
| 1306D03_Stormohina_lunata_F_SouthAfrica | 4 | 4 | 3 | 3 | 3 | 3 | 3 | 3 | 3 | 3 |
| 1306D05_Stomorhina_lunata_F_SouthAfrica | 4 | 4 | 3 | 3 | 3 | 3 | 3 | 3 | 3 | 3 |
| S21_Stomorhina_lunata_F_Mauritius | 4 | 4 | 3 | 3 | 3 | 3 | 3 | 3 | 3 | 3 |
| S22_Stomorhina_lunata_M_Mauritius | 4 | 4 | 3 | 3 | 3 | 3 | 3 | 3 | 3 | 3 |
| S23_Stomorhina_lunata_M_Malawi | 4 | 4 | 3 | 3 | 3 | 3 | 3 | 3 | 3 | 3 |
| S24_Stomorhina_lunata_F_Malawi | 4 | 4 | 3 | 3 | 3 | 3 | 3 | 3 | 3 | 3 |
| S26_Stomorhina_lunata_M_SouthAfrica | 4 | 4 | 3 | 3 | 3 | 3 | 3 | 3 | 3 | 3 |
| S7_Stomorhina_lunata_F_SouthAfrica | 4 | 4 | 3 | 3 | 3 | 3 | 3 | 3 | 3 | 3 |
| USA18_Stomorhina_lunata_F_SouthAfrica | 4 | 4 | 3 | 3 | 3 | 3 | 3 | 3 | 3 | 3 |
| USA19_Stomorhina_lunata_M_SouthAfrica | 4 | 4 | 3 | 3 | 3 | 3 | 3 | 3 | 3 | 3 |
| 1282A06_Stomorhina_lunata_F_SouthAfrica | 4 | 4 | 3 | 3 | 3 | 3 | 3 | 3 | 3 | 3 |
| 1299A01_Stomorhina_lunata_F_SouthAfrica | 4 | 4 | 3 | 3 | 3 | 3 | 3 | 3 | 3 | 3 |
| 1306B01_Stomorhina_lunata_F_SouthAfrica | 4 | 4 | 3 | 3 | 3 | 3 | 3 | 3 | 3 | 3 |
| S5_Stomorhina_sp1_cf_armatipes_F_SouthAfrica | 4 | 4 | 3 | 3 | 3 | 3 | 3 | 3 | 3 | 3 |
| S8_Stomorhina_sp1_cf_armatipes_F_SouthAfrica | 4 | 4 | 3 | 3 | 3 | 3 | 3 | 3 | 3 | 3 |
| S9_Stomorhina_armatipes_M_SouthAfrica | 4 | 4 | 3 | 3 | 3 | 3 | 3 | 3 | 3 | 3 |
| 1306B08_Stomorhina_guttata_F_SouthAfrica | 5 | 5 | 4 | 4 | 4 | 4 | 4 | 4 | 4 | 4 |
| S17_Stomorhina_guttata_M_SouthAfrica | 5 | 5 | 4 | 4 | 4 | 4 | 4 | 4 | 4 | 4 |
| S16_Stomorhina_guttata_F_SouthAfrica | 5 | 5 | 4 | 4 | 4 | 4 | 4 | 4 | 4 | 4 |
| 1279A02_Rhinia_apicalis_M_Ghana | 6 | 6 | 5 | 5 | 5 | 5 | 5 | 5 | 5 | 5 |
| 1279F06_Rhinia_apicalis_M_Ghana | 6 | 6 | 5 | 5 | 5 | 5 | 5 | 5 | 5 | 5 |
| 1279C08_Rhinia_cf_apicalis_F_Togo | 6 | 6 | 5 | 5 | 5 | 5 | 5 | 5 | 5 | 5 |
| 1279F02_Rhinia_cf_apicalis_F_Ghana | 6 | 6 | 5 | 5 | 5 | 5 | 5 | 5 | 5 | 5 |
| R24_Rhinia_apicalis_F_Namibia | 6 | 6 | 5 | 5 | 5 | 5 | 5 | 5 | 5 | 5 |
| 1282A08_Rhinia_cf_apicalis_M_SouthAfrica | 6 | 6 | 5 | 5 | 5 | 5 | 5 | 5 | 5 | 5 |
| USA11_Rhiniaa_apicalis_F_Kenya | 6 | 6 | 5 | 5 | 5 | 5 | 5 | 5 | 5 | 5 |
| 1279A03_Rhinia_apicalis_M_Togo | 6 | 6 | 5 | 5 | 5 | 5 | 5 | 5 | 5 | 5 |
| 1279B01_Rhinia_cf_apicalis_F_Togo | 6 | 6 | 5 | 5 | 5 | 5 | 5 | 5 | 5 | 5 |
| 1279B06_Rhinia_cf_apicalis_F_Togo | 6 | 6 | 5 | 5 | 5 | 5 | 5 | 5 | 5 | 5 |
| 1279B08_Rhinia_cf_apicalis_F_Togo | 6 | 6 | 5 | 5 | 5 | 5 | 5 | 5 | 5 | 5 |
| 1279C02_Rhinia_cf_apicalis_F_Togo | 6 | 6 | 5 | 5 | 5 | 5 | 5 | 5 | 5 | 5 |
| 1279C03_Rhinia_cf_apicalis_F_Togo | 6 | 6 | 5 | 5 | 5 | 5 | 5 | 5 | 5 | 5 |
| 1279C04_Rhinia_apicalis_M_Togo | 6 | 6 | 5 | 5 | 5 | 5 | 5 | 5 | 5 | 5 |
| 1279C07_Rhinia_apicalis_M_Togo | 6 | 6 | 5 | 5 | 5 | 5 | 5 | 5 | 5 | 5 |
| 1279E03_Rhinia_apicalis_M_Ghana | 6 | 6 | 5 | 5 | 5 | 5 | 5 | 5 | 5 | 5 |
| 1282B06_Rhinia_apicalis_M_SouthAfrica | 6 | 6 | 5 | 5 | 5 | 5 | 5 | 5 | 5 | 5 |
| R12_Rhinia_apicalis_F_Namibia | 6 | 6 | 5 | 5 | 5 | 5 | 5 | 5 | 5 | 5 |
| R18_Rhinia_apicalis_M_Togo | 6 | 6 | 5 | 5 | 5 | 5 | 5 | 5 | 5 | 5 |
| 1279A04_Rhinia_cf_apicalis_F_Togo | 6 | 6 | 5 | 5 | 5 | 5 | 5 | 5 | 5 | 5 |
| 1279A06_Rhinia_cf_apicalis_F_Togo | 6 | 6 | 5 | 5 | 5 | 5 | 5 | 5 | 5 | 5 |
| 1279E04_Rhinia_cf_apicalis_F_Ghana | 6 | 6 | 5 | 5 | 5 | 5 | 5 | 5 | 5 | 5 |
| 1279E02_Rhinia_cf_apicalis_F_Togo | 6 | 6 | 5 | 5 | 5 | 5 | 5 | 5 | 5 | 5 |
| 1279B02_Rhinia_sp1_M_Togo | 6 | 6 | 5 | 5 | 5 | 5 | 5 | 5 | 5 | 5 |
| 1279A05_Rhinia_cf_apicalis_F_Togo | 6 | 6 | 5 | 5 | 5 | 5 | 5 | 5 | 5 | 5 |
| 1279B03_Rhinia_cf_apicalis_F_Togo | 6 | 6 | 5 | 5 | 5 | 5 | 5 | 5 | 5 | 5 |
| 1279E08_Rhinia_cf_apicalis_F_Ghana | 7 | 7 | 6 | 6 | 5 | 5 | 5 | 5 | 5 | 5 |
| 1279B07_Rhinia_sp3_M_Togo | 8 | 8 | 7 | 7 | 6 | 6 | 6 | 6 | 6 | 5 |
| K4_Rhinia_sp9_F_Tanzania | 8 | 8 | 7 | 7 | 6 | 6 | 6 | 6 | 6 | 5 |
| R3_Rhinia_coxendix_M_DRCongo | 9 | 8 | 7 | 7 | 6 | 6 | 6 | 6 | 6 | 5 |
| 1279F05_Rhinia_sp6_F_Ghana | 10 | 9 | 8 | 7 | 6 | 6 | 6 | 6 | 6 | 5 |
| 1279C05_Rhinia_sp8_M_Togo | 11 | 10 | 9 | 8 | 7 | 7 | 6 | 6 | 6 | 5 |
| 1279F03_Rhinia_sp7_F_Ghana | 11 | 10 | 9 | 8 | 7 | 7 | 6 | 6 | 6 | 5 |
| 1282C07_Rhinia_sp5_M_SouthAfrica | 12 | 11 | 10 | 9 | 8 | 8 | 7 | 6 | 6 | 5 |
| 1279F04_Rhinia_sp4_F_Ghana | 13 | 12 | 11 | 10 | 9 | 9 | 8 | 7 | 7 | 6 |
| R2_Rhinia_sp2_F_Burundi | 14 | 13 | 12 | 11 | 10 | 10 | 9 | 8 | 8 | 7 |
| 1279E07_Stegosoma_vinculatum_M_Ghana | 15 | 14 | 13 | 12 | 11 | 11 | 10 | 9 | 9 | 8 |
| 1279F01_Stegosoma_vinculatum_F_Ghana | 16 | 15 | 14 | 13 | 12 | 12 | 11 | 10 | 10 | 9 |
| G5_Stegosoma_vinculatum_F_SouthAfrica | 16 | 15 | 14 | 13 | 12 | 12 | 11 | 10 | 10 | 9 |
| G7_Stegosoma_vinculatum_M_SouthAfrica | 16 | 15 | 14 | 13 | 12 | 12 | 11 | 10 | 10 | 9 |
| F2_Fainia_elongata_F_DRCongo | 17 | 16 | 15 | 14 | 13 | 13 | 12 | 11 | 11 | 10 |
| F5_Fainia_elongata_M_Malawi | 17 | 16 | 15 | 14 | 13 | 13 | 12 | 11 | 11 | 10 |
| F3_Fainia_inexpectata_F_Malawi | 18 | 17 | 16 | 15 | 14 | 14 | 12 | 11 | 11 | 10 |
| F6_Fainia_inexpectata_M_Kenya | 18 | 17 | 16 | 15 | 14 | 14 | 12 | 11 | 11 | 10 |
| F20_Fainia_albitarsis_M_Tanzania | 19 | 18 | 17 | 16 | 15 | 15 | 13 | 11 | 11 | 10 |
| K3_Fainia_albitarsis_F_Tanzania | 19 | 18 | 17 | 16 | 15 | 15 | 13 | 11 | 11 | 10 |
| K7_Fainia_albitarsis_F_Tanzania | 19 | 18 | 17 | 16 | 15 | 15 | 13 | 11 | 11 | 10 |
| USA03_Fainia_albitarsis_M_Kenya | 19 | 18 | 17 | 16 | 15 | 15 | 13 | 11 | 11 | 10 |
| USA04_Fainia_albitarsis_F_Kenya | 19 | 18 | 17 | 16 | 15 | 15 | 13 | 11 | 11 | 10 |
| A5_Cosmina_margaritae_F_Tanzania | 20 | 19 | 18 | 17 | 16 | 16 | 14 | 12 | 12 | 11 |
| A8_Cosmina_gracilis_M_Namibia | 21 | 20 | 19 | 18 | 17 | 17 | 15 | 13 | 13 | 12 |
| C19_Cosmina_sp6_M_Kenya | 22 | 21 | 20 | 19 | 18 | 18 | 16 | 14 | 14 | 13 |
| C10_Cosmina_sp3_F_Madagascar | 23 | 22 | 21 | 20 | 19 | 19 | 17 | 15 | 15 | 14 |
| C11_Cosmina_sp2_M_Madagascar | 24 | 23 | 22 | 21 | 20 | 20 | 18 | 16 | 16 | 15 |
| C20_Cosmina_sp1_cf_fuscipennis_F_SouthAfrica | 25 | 24 | 23 | 22 | 21 | 21 | 19 | 17 | 17 | 16 |
| USA01_Cosmina_fuscipennis_F_SouthAfrica | 25 | 24 | 23 | 22 | 21 | 21 | 19 | 17 | 17 | 16 |
| USA02_Cosmina_fuscipennis_M_SouthAfrica | 25 | 24 | 23 | 22 | 21 | 21 | 19 | 17 | 17 | 16 |
| A10_Rhyncomya_sp14_F_Tanzania | 26 | 25 | 24 | 23 | 22 | 22 | 20 | 18 | 18 | 17 |
| Y15_Rhyncomya_forcipata_F_SouthAfrica | 27 | 26 | 25 | 24 | 23 | 23 | 21 | 19 | 19 | 18 |
| Y28_Rhyncomya_forcipata_F_SouthAfrica | 27 | 26 | 25 | 24 | 23 | 23 | 21 | 19 | 19 | 18 |
| Y29_Rhyncomya_forcipata_M_Namibia | 27 | 26 | 25 | 24 | 23 | 23 | 21 | 19 | 19 | 18 |
| Y27_Rhyncomya_forcipata_F_SouthAfrica | 27 | 26 | 25 | 24 | 23 | 23 | 21 | 19 | 19 | 18 |
| Y35_Rhyncomya_cassotis_M_Malawi | 28 | 27 | 26 | 25 | 24 | 24 | 22 | 20 | 20 | 19 |
| Y38_Rhyncomya_cassotis_F_Malawi | 28 | 27 | 26 | 25 | 24 | 24 | 22 | 20 | 20 | 19 |
| Y36_Rhyncomya_sp11_cf_cassotis_M_Togo | 29 | 27 | 26 | 25 | 24 | 24 | 22 | 20 | 20 | 19 |
| Y41_Rhyncomya_sp1_F_Cameroon | 29 | 27 | 26 | 25 | 24 | 24 | 22 | 20 | 20 | 19 |
| Y37_Rhyncomya_sp12_cf_cassotis_M_Zambia | 30 | 28 | 27 | 26 | 25 | 24 | 22 | 20 | 20 | 19 |
| Y39_Rhyncomya_cassotis_F_Namibia | 30 | 28 | 27 | 26 | 25 | 24 | 22 | 20 | 20 | 19 |
| Y56_Rhyncomya_sp19_F_Cameroon | 31 | 29 | 28 | 27 | 26 | 25 | 23 | 21 | 21 | 20 |
| Y40_Rhyncomya_sp3_F_SouthAfrica | 32 | 30 | 29 | 28 | 27 | 26 | 24 | 22 | 22 | 21 |
| Y57_Rhyncomya_sp3_F_SouthAfrica | 32 | 30 | 29 | 28 | 27 | 26 | 24 | 22 | 22 | 21 |
| A9_Rhyncomya_interclusa_M_SouthAfrica | 33 | 31 | 30 | 29 | 28 | 27 | 25 | 23 | 23 | 22 |
| Y69_Rhyncomya_interclusa_M_SouthAfrica | 33 | 31 | 30 | 29 | 28 | 27 | 25 | 23 | 23 | 22 |
| Y6_Rhyncomya_sp15_F_SouthAfrica | 34 | 32 | 31 | 30 | 29 | 28 | 26 | 24 | 24 | 23 |
| E1_Eurhyncomyia_diversicolor_M_Mozambique | 35 | 33 | 32 | 31 | 30 | 29 | 27 | 25 | 25 | 24 |
| E2_Eurhyncomyia_diversicolor_F_Mozambique | 35 | 33 | 32 | 31 | 30 | 29 | 27 | 25 | 25 | 24 |
| USA12_Rhyncomya_minutalis_F_SouthAfrica | 36 | 34 | 33 | 32 | 31 | 30 | 28 | 26 | 26 | 25 |
| Y13_Rhyncomya_sp16_cf_minutalis_M_SouthAfrica | 36 | 34 | 33 | 32 | 31 | 30 | 28 | 26 | 26 | 25 |
| Y32_Rhyncomya_minutalis_F_SouthAfrica | 37 | 34 | 33 | 32 | 31 | 30 | 28 | 26 | 26 | 25 |
| Y20_Rhyncomya_maculata_M_SouthAfrica | 38 | 34 | 33 | 32 | 31 | 30 | 28 | 26 | 26 | 25 |
| Y67_Rhyncomya_sp7_M_SouthAfrica | 39 | 35 | 33 | 32 | 31 | 30 | 28 | 26 | 26 | 25 |
| 1279A07_Stomorhina_chapini_F_Togo | 40 | 36 | 34 | 33 | 32 | 31 | 29 | 27 | 27 | 26 |
| S2_Stomorhina_chapini_F_DRCongo | 40 | 36 | 34 | 33 | 32 | 31 | 29 | 27 | 27 | 26 |
| S3_Stomorhina_chapini_F_SouthAfrica | 41 | 37 | 35 | 34 | 33 | 32 | 30 | 28 | 28 | 27 |
| S4_Stomorhina_chapini_M_SouthAfrica | 41 | 37 | 35 | 34 | 33 | 32 | 30 | 28 | 28 | 27 |
| G1_Stegosoma_bowdeni_M_Togo | 42 | 38 | 36 | 35 | 34 | 33 | 31 | 29 | 29 | 28 |
| G3_Stegosoma_bowdeni_F_Togo | 42 | 38 | 36 | 35 | 34 | 33 | 31 | 29 | 29 | 28 |
| 1279C01_Stegosoma_wellmani_M_Togo | 43 | 39 | 37 | 36 | 35 | 34 | 32 | 30 | 30 | 29 |
| G8_Stegosoma_sp1_cf_wellmani_F_DRCongo | 44 | 40 | 38 | 37 | 36 | 35 | 33 | 31 | 31 | 30 |
| Y58_Rhyncomya_trispina_M_Namibia | 45 | 41 | 39 | 38 | 37 | 36 | 34 | 32 | 32 | 31 |
| Y59_Rhyncomya_trispina_F_SouthAfrica | 45 | 41 | 39 | 38 | 37 | 36 | 34 | 32 | 32 | 31 |
| S19_Stomorhina_apta_F_Burundi | 46 | 42 | 40 | 39 | 38 | 37 | 35 | 33 | 33 | 32 |
| S20_Stomorhina_apta_F_Burundi | 46 | 42 | 40 | 39 | 38 | 37 | 35 | 33 | 33 | 32 |
| S28_Stomorhina_cf_malobana_F_Malawi | 47 | 43 | 41 | 39 | 38 | 37 | 35 | 33 | 33 | 32 |
| S30_Stomorhina_sp2_cf_malobana_F_Malawi | 47 | 43 | 41 | 39 | 38 | 37 | 35 | 33 | 33 | 32 |
| S31_Stomorhina_malobana_M_Malawi | 47 | 43 | 41 | 39 | 38 | 37 | 35 | 33 | 33 | 32 |
| S40_Stomorhina_sp2_cf_malobana_F_Tanzania | 47 | 43 | 41 | 39 | 38 | 37 | 35 | 33 | 33 | 32 |
| S42_Stomorhina_sp2_cf_malobana_F_Tanzania | 47 | 43 | 41 | 39 | 38 | 37 | 35 | 33 | 33 | 32 |
| USA13_Rhyncomya_soyauxi_F_Kenya | 48 | 44 | 42 | 40 | 39 | 38 | 36 | 34 | 34 | 33 |
| USA14_Rhyncomya_soyauxi_M_Kenya | 48 | 44 | 42 | 40 | 39 | 38 | 36 | 34 | 34 | 33 |
| USA15_Rhyncomya_soyauxi_F_Kenya | 48 | 44 | 42 | 40 | 39 | 38 | 36 | 34 | 34 | 33 |
| USA16_Rhyncomya_soyauxi_M_Kenya | 49 | 45 | 43 | 41 | 40 | 39 | 37 | 35 | 35 | 34 |
| C18_Cosmina_sp5_F_Kenya | 50 | 46 | 44 | 42 | 41 | 40 | 38 | 36 | 36 | 35 |
| A3_Albaredaya_malgache_F_Madagascar | 51 | 47 | 45 | 43 | 42 | 41 | 39 | 37 | 37 | 36 |
| K5_Albaredaya_malgache_F_Madagascar | 51 | 47 | 45 | 43 | 42 | 41 | 39 | 37 | 37 | 36 |
| 1282A03_Isomyia_tristis_M_SouthAfrica | 52 | 48 | 46 | 44 | 43 | 42 | 40 | 38 | 38 | 37 |
| 1282A04_Isomyia_tristis_F_SouthAfrica | 52 | 48 | 46 | 44 | 43 | 42 | 40 | 38 | 38 | 37 |
| 1282B01_Isomyia_tristis_F_SouthAfrica | 52 | 48 | 46 | 44 | 43 | 42 | 40 | 38 | 38 | 37 |
| 1282B04_Isomyia_tristis_F_SouthAfrica | 52 | 48 | 46 | 44 | 43 | 42 | 40 | 38 | 38 | 37 |
| 1282B05_Isomyia_trisits_F_SouthAfrica | 52 | 48 | 46 | 44 | 43 | 42 | 40 | 38 | 38 | 37 |
| 1306F04_Isomyia_tristis_F_SouthAfrica | 52 | 48 | 46 | 44 | 43 | 42 | 40 | 38 | 38 | 37 |
| USA10_Isomyia_tristis_F_SouthAfrica | 52 | 48 | 46 | 44 | 43 | 42 | 40 | 38 | 38 | 37 |
| 1306A07_Isomyia_tristis_F_SouthAfrica | 53 | 49 | 47 | 45 | 44 | 43 | 41 | 39 | 38 | 37 |
| 1306B03_Isomyia_trisits_F_SouthAfrica | 53 | 49 | 47 | 45 | 44 | 43 | 41 | 39 | 38 | 37 |
| 1306B04_Isomyia_tristis_F_SouthAfrica | 53 | 49 | 47 | 45 | 44 | 43 | 41 | 39 | 38 | 37 |
| 1306C04_Isomyia_tristis_F_SouthAfrica | 53 | 49 | 47 | 45 | 44 | 43 | 41 | 39 | 38 | 37 |
| 1306C06_Isomyia_tristis_M_SouthAfrica | 53 | 49 | 47 | 45 | 44 | 43 | 41 | 39 | 38 | 37 |
| 1306C08_Isomyia_tristis_M_SouthAfrica | 53 | 49 | 47 | 45 | 44 | 43 | 41 | 39 | 38 | 37 |
| 1306F03_Isomyia_tristis_F_SouthAfrica | 53 | 49 | 47 | 45 | 44 | 43 | 41 | 39 | 38 | 37 |
| USA09_Isomyia_tristis_M_SouthAfrica | 53 | 49 | 47 | 45 | 44 | 43 | 41 | 39 | 38 | 37 |
| I20B_Isomyia_tristis_M_SouthAfrica | 53 | 49 | 47 | 45 | 44 | 43 | 41 | 39 | 38 | 37 |
| Y23_Rhyncomya_pruinosa_M_SouthAfrica | 54 | 50 | 48 | 46 | 45 | 44 | 42 | 40 | 39 | 38 |
| Y24_Rhyncomya_pruinosa_M_Malawi | 54 | 50 | 48 | 46 | 45 | 44 | 42 | 40 | 39 | 38 |
| Y26_Rhyncomya_pruinosa_F_SouthAfrica | 54 | 50 | 48 | 46 | 45 | 44 | 42 | 40 | 39 | 38 |
| Y25_Rhyncomya_pruinosa_F_Kenya | 54 | 50 | 48 | 46 | 45 | 44 | 42 | 40 | 39 | 38 |
| T1_Thoracites_sp1_M_SouthAfrica | 55 | 51 | 49 | 47 | 46 | 45 | 43 | 41 | 40 | 39 |
| T3_Thoracites_sp1_F_SouthAfrica | 55 | 51 | 49 | 47 | 46 | 45 | 43 | 41 | 40 | 39 |
| T6_Thoracites_petersiana_F_SouthAfrica | 56 | 52 | 50 | 48 | 47 | 46 | 44 | 42 | 41 | 40 |
| T7_Thoracites_petersiana_M_SouthAfrica | 56 | 52 | 50 | 48 | 47 | 46 | 44 | 42 | 41 | 40 |
| Y44_Rhyncomya_sp8_F_Namibia | 57 | 53 | 51 | 49 | 48 | 47 | 45 | 43 | 42 | 41 |
| 1306A06_Isomyia_pubera_F_SouthAfrica | 58 | 54 | 52 | 50 | 49 | 48 | 46 | 44 | 43 | 42 |
| I5_Isomyia_pubera_M_SouthAfrica | 58 | 54 | 52 | 50 | 49 | 48 | 46 | 44 | 43 | 42 |
| USA07_Isomyia_pubera_F_SouthAfrica | 58 | 54 | 52 | 50 | 49 | 48 | 46 | 44 | 43 | 42 |
| A12_Vanemdenia_africana_F_Tanzania | 59 | 55 | 53 | 51 | 50 | 49 | 47 | 45 | 44 | 43 |
| Y46_Rhyncomya_paratristis_F_SouthAfrica | 60 | 56 | 54 | 52 | 51 | 50 | 48 | 46 | 45 | 44 |
| C8_Cosmina_sp9_cf_testaceipes_M_Madagascar | 61 | 57 | 55 | 53 | 52 | 51 | 49 | 47 | 46 | 45 |
| 1306A08_Rhyncomya_disclusa_M_SouthAfrica | 62 | 58 | 56 | 54 | 53 | 52 | 50 | 48 | 47 | 46 |
| 1306E03_Rhyncomya_disclusa_F_SouthAfrica | 62 | 58 | 56 | 54 | 53 | 52 | 50 | 48 | 47 | 46 |
| 1306E04_Rhyncomya_disclusa_F_SouthAfrica | 62 | 58 | 56 | 54 | 53 | 52 | 50 | 48 | 47 | 46 |
| 1306E05_Rhyncomya_disclusa_F_SouthAfrica | 62 | 58 | 56 | 54 | 53 | 52 | 50 | 48 | 47 | 46 |
| Y64_Rhyncomya_sp6_M_Togo | 63 | 59 | 57 | 55 | 54 | 53 | 51 | 49 | 48 | 47 |
| Y65_Rhyncomya_sp6_F_Togo | 63 | 59 | 57 | 55 | 54 | 53 | 51 | 49 | 48 | 47 |
| C3_Cosmina_aenea_M_Namibia | 64 | 60 | 58 | 56 | 55 | 54 | 52 | 50 | 49 | 48 |
| C4_Cosmina_aenea_F_Namibia | 64 | 60 | 58 | 56 | 55 | 54 | 52 | 50 | 49 | 48 |
| Y47_Rhyncomya_dasyops_F_SouthAfrica | 65 | 61 | 59 | 57 | 56 | 55 | 53 | 51 | 50 | 49 |
| I17_Isomyia_distinguenda_F_Malawi | 66 | 62 | 60 | 58 | 57 | 56 | 54 | 52 | 51 | 50 |
| I19_Isomyia_distinguenda_F_Malawi | 66 | 62 | 60 | 58 | 57 | 56 | 54 | 52 | 51 | 50 |
| I8_Isomyia_distinguenda_M_SouthAfrica | 66 | 62 | 60 | 58 | 57 | 56 | 54 | 52 | 51 | 50 |
| I4_Isomyia_cuthbertsoni_M_SouthAfrica | 67 | 63 | 61 | 59 | 58 | 57 | 55 | 53 | 52 | 50 |
| I6_Isomyia_sp3_cf_cuthbertsoni_F_SouthAfrica | 67 | 63 | 61 | 59 | 58 | 57 | 55 | 53 | 52 | 50 |
| I9_Isomyia_sp3_cf_cuthbertsoni_F_SouthAfrica | 67 | 63 | 61 | 59 | 58 | 57 | 55 | 53 | 52 | 50 |
| I18_Isomyia_sp6_M_Togo | 68 | 64 | 62 | 60 | 59 | 58 | 56 | 54 | 53 | 51 |
| Y10_Zumba_antennalis_F_SouthAfrica | 69 | 65 | 63 | 61 | 60 | 59 | 57 | 55 | 54 | 52 |
| Z2_Zumba_antennalis_F_SouthAfrica | 69 | 65 | 63 | 61 | 60 | 59 | 57 | 55 | 54 | 52 |
| I11_Isomyia_darwini_F_SouthAfrica | 70 | 66 | 64 | 62 | 61 | 60 | 58 | 56 | 55 | 53 |
| 1306B05_Isomyia_natalensis_F_SouthAfrica | 71 | 67 | 65 | 63 | 62 | 61 | 59 | 57 | 56 | 54 |
| 1306C05_Isomyia_natalensis_F_SouthAfrica | 71 | 67 | 65 | 63 | 62 | 61 | 59 | 57 | 56 | 54 |
| 1306D04_Isomyia_natalensis_F_SouthAfrica | 71 | 67 | 65 | 63 | 62 | 61 | 59 | 57 | 56 | 54 |
| USA05_Isomyia_nataliensis_F_SouthAfrica | 71 | 67 | 65 | 63 | 62 | 61 | 59 | 57 | 56 | 54 |
| USA06_Isomyia_nataliensis_M_SouthAfrica | 71 | 67 | 65 | 63 | 62 | 61 | 59 | 57 | 56 | 54 |
| USA08_Isomyia_natalensis_F_SouthAfrica | 71 | 67 | 65 | 63 | 62 | 61 | 59 | 57 | 56 | 54 |
| 1306F01_Isomyia_natalensis_F_SouthAfrica | 71 | 67 | 65 | 63 | 62 | 61 | 59 | 57 | 56 | 54 |
| I10_Isomyia_eos_F_SouthAfrica | 72 | 68 | 66 | 64 | 63 | 62 | 60 | 58 | 57 | 55 |
| I15_Isomyia_sp2_M_Cameroon | 73 | 69 | 67 | 65 | 64 | 63 | 61 | 59 | 58 | 56 |
| B1_Trichoberia_sp1_M_SouthAfrica | 74 | 70 | 68 | 66 | 65 | 64 | 62 | 60 | 59 | 57 |
| I1_Isomyia_dubiosa_F_Togo | 75 | 71 | 69 | 67 | 66 | 65 | 63 | 61 | 60 | 58 |
| I2_Isomyia_dubiosa_F_Togo | 75 | 71 | 69 | 67 | 66 | 65 | 63 | 61 | 60 | 58 |
| I12_Isomyia_dubiosa_F_Togo | 75 | 71 | 69 | 67 | 66 | 65 | 63 | 61 | 60 | 58 |
| I14_Isomyia_sp1_F_Uganda | 76 | 72 | 70 | 68 | 67 | 66 | 64 | 62 | 61 | 59 |

**- ABGD –**

20250226_Rhiniinae_216_MF_out_groups.res.cvs

| prior | nbSubsetInitial | nbSubsetRecursive |
| --- | --- | --- |
| 0.001 | 105 | 119 |
| 0.001668 | 105 | 105 |
| 0.002783 | 105 | 105 |
| 0.004642 | 78 | 79 |
| 0.007743 | 59 | 66 |
| 0.012915 | 59 | 63 |
| 0.021544 | 1 | 1 |

20250226_Rhiniinae_216_MF_out_groups.rank.svg


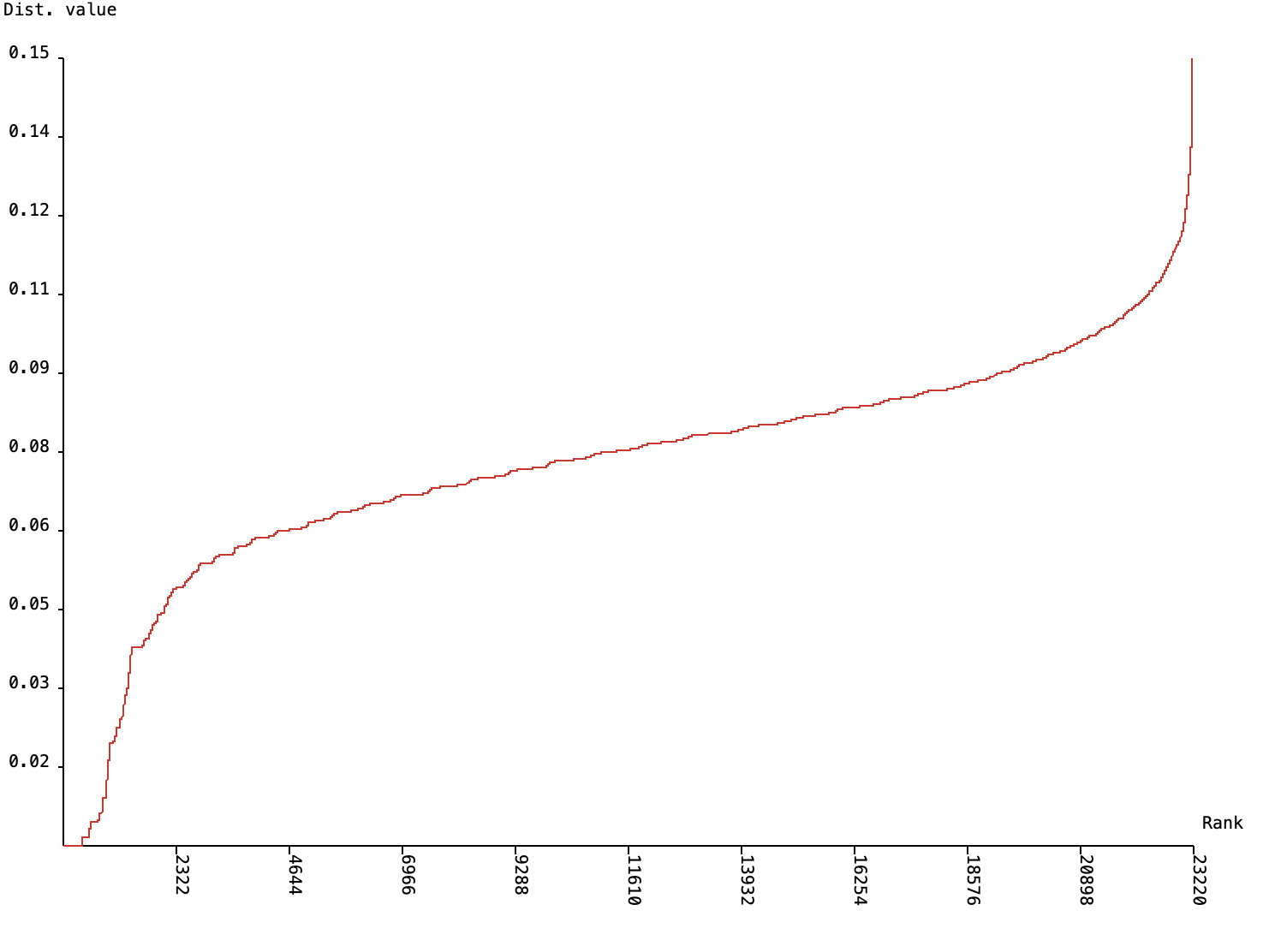


20250226_Rhiniinae_216_MF_out_groups.disthist.svg


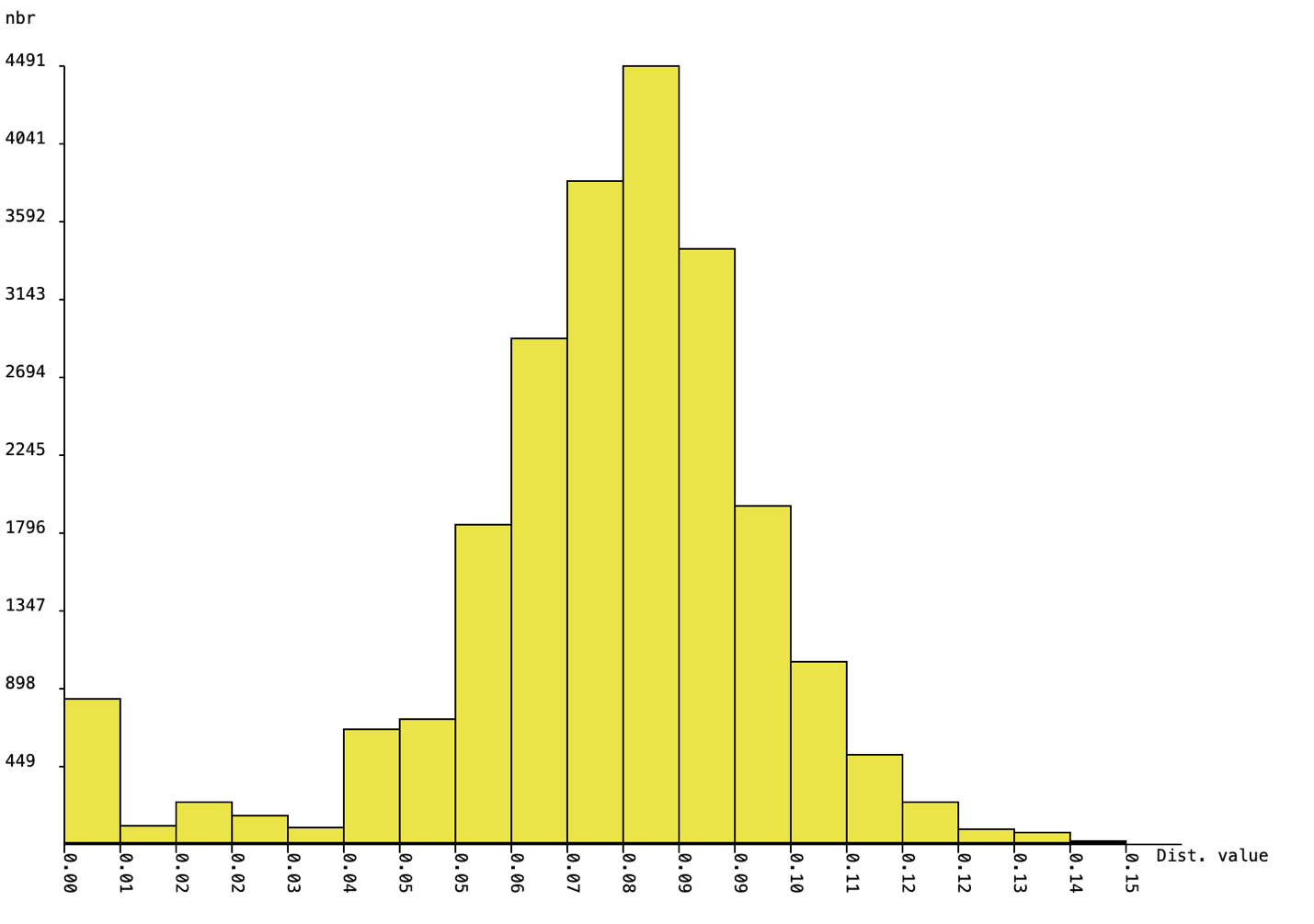


begin spart;

Project_name = 20250226_Rhiniinae_216_MF_out_groups;

Date = 2025-03-13T14:08:23;

[Generated by ABGD with Distance K80 Kimura / MinSlope = 0.900000]

[WARNING: The sample names below may have been changed to fit SPART specification (only alphanumeric characters and _ )]

| **N_spartitions (6)** | **abgd_init_1** | **abgd_init_2** | **abgd_init_3** | **abgd_init_4** | **abgd_init_5** | **abgd_init_6** |
| --- | --- | --- | --- | --- | --- | --- |
| **N_individuals** | **216** | **216** | **216** | **216** | **216** | **216** |
| **N_subsets** | **105** | **105** | **105** | **78** | **59** | **59** |
| **Prior; Barcode gap distance** | **1.0000E-03** | **1.6700E-03** | **2.7800E-03** | **4.6400E-03** | **7.7400E-03** | **1.2916E-02** |
| 1279A01_Stomorhina_cribrata_F_Ghana | 1 | 1 | 1 | 1 | 1 | 1 |
| 1279A02_Rhinia_apicalis_M_Ghana | 2 | 2 | 2 | 2 | 2 | 2 |
| 1279A03_Rhinia_apicalis_M_Togo | 3 | 3 | 3 | 2 | 2 | 2 |
| 1279A04_Rhinia_cf_apicalis_F_Togo | 3 | 3 | 3 | 2 | 2 | 2 |
| 1279A05_Rhinia_cf_apicalis_F_Togo | 4 | 4 | 4 | 2 | 2 | 2 |
| 1279A06_Rhinia_cf_apicalis_F_Togo | 3 | 3 | 3 | 2 | 2 | 2 |
| 1279A07_Stomorhina_chapini_F_Togo | 5 | 5 | 5 | 3 | 3 | 3 |
| 1279B01_Rhinia_cf_apicalis_F_Togo | 3 | 3 | 3 | 2 | 2 | 2 |
| 1279B02_Rhinia_sp1_M_Togo | 6 | 6 | 6 | 2 | 2 | 2 |
| 1279B03_Rhinia_cf_apicalis_F_Togo | 7 | 7 | 7 | 2 | 2 | 2 |
| 1279B06_Rhinia_cf_apicalis_F_Togo | 3 | 3 | 3 | 2 | 2 | 2 |
| 1279B07_Rhinia_sp3_M_Togo | 8 | 8 | 8 | 4 | 2 | 2 |
| 1279B08_Rhinia_cf_apicalis_F_Togo | 3 | 3 | 3 | 2 | 2 | 2 |
| 1279C01_Stegosoma_wellmani_M_Togo | 9 | 9 | 9 | 5 | 4 | 4 |
| 1279C02_Rhinia_cf_apicalis_F_Togo | 3 | 3 | 3 | 2 | 2 | 2 |
| 1279C03_Rhinia_cf_apicalis_F_Togo | 3 | 3 | 3 | 2 | 2 | 2 |
| 1279C04_Rhinia_apicalis_M_Togo | 3 | 3 | 3 | 2 | 2 | 2 |
| 1279C05_Rhinia_sp8_M_Togo | 10 | 10 | 10 | 6 | 2 | 2 |
| 1279C06_Stomorhina_cribrata_M_Togo | 1 | 1 | 1 | 1 | 1 | 1 |
| 1279C07_Rhinia_apicalis_M_Togo | 3 | 3 | 3 | 2 | 2 | 2 |
| 1279C08_Rhinia_cf_apicalis_F_Togo | 2 | 2 | 2 | 2 | 2 | 2 |
| 1279D01_Stomorhina_cribrata_F_Togo | 1 | 1 | 1 | 1 | 1 | 1 |
| 1279D02_Stomorhina_cribrata_M_Togo | 1 | 1 | 1 | 1 | 1 | 1 |
| 1279D05_Stomorhina_rugosa_M_Togo | 11 | 11 | 11 | 7 | 5 | 5 |
| 1279E01_Stomorhina_cribrata_F_Togo | 1 | 1 | 1 | 1 | 1 | 1 |
| 1279E02_Rhinia_cf_apicalis_F_Togo | 12 | 12 | 12 | 2 | 2 | 2 |
| 1279E03_Rhinia_apicalis_M_Ghana | 3 | 3 | 3 | 2 | 2 | 2 |
| 1279E04_Rhinia_cf_apicalis_F_Ghana | 3 | 3 | 3 | 2 | 2 | 2 |
| 1279E07_Stegosoma_vinculatum_M_Ghana | 13 | 13 | 13 | 8 | 6 | 6 |
| 1279E08_Rhinia_cf_apicalis_F_Ghana | 14 | 14 | 14 | 9 | 2 | 2 |
| 1279F01_Stegosoma_vinculatum_F_Ghana | 15 | 15 | 15 | 10 | 7 | 7 |
| 1279F02_Rhinia_cf_apicalis_F_Ghana | 2 | 2 | 2 | 2 | 2 | 2 |
| 1279F03_Rhinia_sp7_F_Ghana | 16 | 16 | 16 | 6 | 2 | 2 |
| 1279F04_Rhinia_sp4_F_Ghana | 17 | 17 | 17 | 11 | 8 | 8 |
| 1279F05_Rhinia_sp6_F_Ghana | 18 | 18 | 18 | 12 | 2 | 2 |
| 1279F06_Rhinia_apicalis_M_Ghana | 2 | 2 | 2 | 2 | 2 | 2 |
| 1282A01_Stomorhina_lunata_M_SouthAfrica | 19 | 19 | 19 | 13 | 9 | 9 |
| 1282A03_Isomyia_tristis_M_SouthAfrica | 20 | 20 | 20 | 14 | 10 | 10 |
| 1282A04_Isomyia_tristis_F_SouthAfrica | 20 | 20 | 20 | 14 | 10 | 10 |
| 1282A06_Stomorhina_lunata_F_SouthAfrica | 19 | 19 | 19 | 13 | 9 | 9 |
| 1282A08_Rhinia_cf_apicalis_M_SouthAfrica | 2 | 2 | 2 | 2 | 2 | 2 |
| 1282B01_Isomyia_tristis_F_SouthAfrica | 20 | 20 | 20 | 14 | 10 | 10 |
| 1282B04_Isomyia_tristis_F_SouthAfrica | 20 | 20 | 20 | 14 | 10 | 10 |
| 1282B05_Isomyia_trisits_F_SouthAfrica | 20 | 20 | 20 | 14 | 10 | 10 |
| 1282B06_Rhinia_apicalis_M_SouthAfrica | 3 | 3 | 3 | 2 | 2 | 2 |
| 1282C01_Stomorhina_lunata_M_SouthAfrica | 19 | 19 | 19 | 13 | 9 | 9 |
| 1282C02_Stomorhina_cribrata_M_SouthAfrica | 1 | 1 | 1 | 1 | 1 | 1 |
| 1282C03_Stomorhina_lunata_M_SouthAfrica | 19 | 19 | 19 | 13 | 9 | 9 |
| 1282C07_Rhinia_sp5_M_SouthAfrica | 21 | 21 | 21 | 15 | 2 | 2 |
| 1282C08_Stomorhina_lunata_F_SouthAfrica | 19 | 19 | 19 | 13 | 9 | 9 |
| 1282D01_Stomorhina_rugosa_M_SouthAfrica | 11 | 11 | 11 | 7 | 5 | 5 |
| 1282D02_Stomorhina_lunata_M_SouthAfrica | 19 | 19 | 19 | 13 | 9 | 9 |
| 1282D03_Stomorhina_rugosa_F_SouthAfrica | 22 | 22 | 22 | 7 | 5 | 5 |
| 1299A01_Stomorhina_lunata_F_SouthAfrica | 19 | 19 | 19 | 13 | 9 | 9 |
| 1299A02_Stomorhina_lunata_F_SouthAfrica | 19 | 19 | 19 | 13 | 9 | 9 |
| 1299A03_Stomorhina_lunata_F_SouthAfrica | 19 | 19 | 19 | 13 | 9 | 9 |
| 1306A01_Stomorhina_lunata_F_SouthAfrica | 19 | 19 | 19 | 13 | 9 | 9 |
| 1306A06_Isomyia_pubera_F_SouthAfrica | 23 | 23 | 23 | 16 | 11 | 11 |
| 1306A07_Isomyia_tristis_F_SouthAfrica | 24 | 24 | 24 | 17 | 10 | 10 |
| 1306A08_Rhyncomya_disclusa_M_SouthAfrica | 25 | 25 | 25 | 18 | 12 | 12 |
| 1306B01_Stomorhina_lunata_F_SouthAfrica | 26 | 26 | 26 | 13 | 9 | 9 |
| 1306B03_Isomyia_trisits_F_SouthAfrica | 24 | 24 | 24 | 17 | 10 | 10 |
| 1306B04_Isomyia_tristis_F_SouthAfrica | 24 | 24 | 24 | 17 | 10 | 10 |
| 1306B05_Isomyia_natalensis_F_SouthAfrica | 27 | 27 | 27 | 19 | 13 | 13 |
| 1306B06_Stomorhina_cribrata_F_SouthAfrica | 1 | 1 | 1 | 1 | 1 | 1 |
| 1306B08_Stomorhina_guttata_F_SouthAfrica | 28 | 28 | 28 | 20 | 14 | 14 |
| 1306C01_Stomorhina_lunata_M_SouthAfrica | 19 | 19 | 19 | 13 | 9 | 9 |
| 1306C03_Stomorhina_cribrata_M_SouthAfrica | 1 | 1 | 1 | 1 | 1 | 1 |
| 1306C04_Isomyia_tristis_F_SouthAfrica | 24 | 24 | 24 | 17 | 10 | 10 |
| 1306C05_Isomyia_natalensis_F_SouthAfrica | 27 | 27 | 27 | 19 | 13 | 13 |
| 1306C06_Isomyia_tristis_M_SouthAfrica | 24 | 24 | 24 | 17 | 10 | 10 |
| 1306C08_Isomyia_tristis_M_SouthAfrica | 24 | 24 | 24 | 17 | 10 | 10 |
| 1306D03_Stormohina_lunata_F_SouthAfrica | 19 | 19 | 19 | 13 | 9 | 9 |
| 1306D04_Isomyia_natalensis_F_SouthAfrica | 27 | 27 | 27 | 19 | 13 | 13 |
| 1306D05_Stomorhina_lunata_F_SouthAfrica | 19 | 19 | 19 | 13 | 9 | 9 |
| 1306E02_Stomorhina_cribrata_F_SouthAfrica | 1 | 1 | 1 | 1 | 1 | 1 |
| 1306E03_Rhyncomya_disclusa_F_SouthAfrica | 25 | 25 | 25 | 18 | 12 | 12 |
| 1306E04_Rhyncomya_disclusa_F_SouthAfrica | 25 | 25 | 25 | 18 | 12 | 12 |
| 1306E05_Rhyncomya_disclusa_F_SouthAfrica | 25 | 25 | 25 | 18 | 12 | 12 |
| 1306F01_Isomyia_natalensis_F_SouthAfrica | 27 | 27 | 27 | 19 | 13 | 13 |
| 1306F03_Isomyia_tristis_F_SouthAfrica | 24 | 24 | 24 | 17 | 10 | 10 |
| 1306F04_Isomyia_tristis_F_SouthAfrica | 20 | 20 | 20 | 14 | 10 | 10 |
| 1306F05_Stomorhina_cribrata_F_SouthAfrica | 29 | 29 | 29 | 21 | 1 | 1 |
| 1306F06_Stomorhina_cribrata_F_SouthAfrica | 1 | 1 | 1 | 1 | 1 | 1 |
| 1306F07_Stomorhina_cribrata_F_SouthAfrica | 1 | 1 | 1 | 1 | 1 | 1 |
| 1306F08_Stomorhina_cribrata_F_SouthAfrica | 1 | 1 | 1 | 1 | 1 | 1 |
| A10_Rhyncomya_sp14_F_Tanzania | 30 | 30 | 30 | 22 | 15 | 15 |
| A12_Vanemdenia_africana_F_Tanzania | 31 | 31 | 31 | 23 | 16 | 16 |
| A3_Albaredaya_malgache_F_Madagascar | 32 | 32 | 32 | 24 | 17 | 17 |
| A5_Cosmina_margaritae_F_Tanzania | 33 | 33 | 33 | 25 | 18 | 18 |
| A8_Cosmina_gracilis_M_Namibia | 34 | 34 | 34 | 26 | 19 | 19 |
| A9_Rhyncomya_interclusa_M_SouthAfrica | 35 | 35 | 35 | 27 | 20 | 20 |
| B1_Trichoberia_sp1_M_SouthAfrica | 36 | 36 | 36 | 28 | 21 | 21 |
| C10_Cosmina_sp3_F_Madagascar | 37 | 37 | 37 | 29 | 22 | 22 |
| C11_Cosmina_sp2_M_Madagascar | 38 | 38 | 38 | 30 | 23 | 23 |
| C18_Cosmina_sp5_F_Kenya | 39 | 39 | 39 | 31 | 24 | 24 |
| C19_Cosmina_sp6_M_Kenya | 40 | 40 | 40 | 32 | 25 | 25 |
| C20_Cosmina_sp1_cf_fuscipennis_F_SouthAfrica | 41 | 41 | 41 | 33 | 26 | 26 |
| C3_Cosmina_aenea_M_Namibia | 42 | 42 | 42 | 34 | 27 | 27 |
| C4_Cosmina_aenea_F_Namibia | 42 | 42 | 42 | 34 | 27 | 27 |
| C8_Cosmina_sp9_cf_testaceipes_M_Madagascar | 43 | 43 | 43 | 35 | 28 | 28 |
| E1_Eurhyncomyia_diversicolor_M_Mozambique | 44 | 44 | 44 | 36 | 29 | 29 |
| E2_Eurhyncomyia_diversicolor_F_Mozambique | 44 | 44 | 44 | 36 | 29 | 29 |
| F2_Fainia_elongata_F_DRCongo | 45 | 45 | 45 | 37 | 30 | 30 |
| F20_Fainia_albitarsis_M_Tanzania | 46 | 46 | 46 | 38 | 30 | 30 |
| F3_Fainia_inexpectata_F_Malawi | 47 | 47 | 47 | 39 | 30 | 30 |
| F5_Fainia_elongata_M_Malawi | 45 | 45 | 45 | 37 | 30 | 30 |
| F6_Fainia_inexpectata_M_Kenya | 48 | 48 | 48 | 39 | 30 | 30 |
| G1_Stegosoma_bowdeni_M_Togo | 49 | 49 | 49 | 40 | 31 | 31 |
| G3_Stegosoma_bowdeni_F_Togo | 49 | 49 | 49 | 40 | 31 | 31 |
| G5_Stegosoma_vinculatum_F_SouthAfrica | 50 | 50 | 50 | 10 | 7 | 7 |
| G7_Stegosoma_vinculatum_M_SouthAfrica | 50 | 50 | 50 | 10 | 7 | 7 |
| G8_Stegosoma_sp1_cf_wellmani_F_DRCongo | 51 | 51 | 51 | 41 | 32 | 32 |
| I1_Isomyia_dubiosa_F_Togo | 52 | 52 | 52 | 42 | 33 | 33 |
| I10_Isomyia_eos_F_SouthAfrica | 53 | 53 | 53 | 43 | 34 | 34 |
| I11_Isomyia_darwini_F_SouthAfrica | 54 | 54 | 54 | 44 | 35 | 35 |
| I12_Isomyia_dubiosa_F_Togo | 55 | 55 | 55 | 42 | 33 | 33 |
| I14_Isomyia_sp1_F_Uganda | 56 | 56 | 56 | 45 | 36 | 36 |
| I15_Isomyia_sp2_M_Cameroon | 57 | 57 | 57 | 46 | 37 | 37 |
| I17_Isomyia_distinguenda_F_Malawi | 58 | 58 | 58 | 47 | 38 | 38 |
| I18_Isomyia_sp6_M_Togo | 59 | 59 | 59 | 48 | 39 | 39 |
| I19_Isomyia_distinguenda_F_Malawi | 60 | 60 | 60 | 47 | 38 | 38 |
| I2_Isomyia_dubiosa_F_Togo | 52 | 52 | 52 | 42 | 33 | 33 |
| I20B_Isomyia_tristis_M_SouthAfrica | 61 | 61 | 61 | 17 | 10 | 10 |
| I4_Isomyia_cuthbertsoni_M_SouthAfrica | 62 | 62 | 62 | 49 | 38 | 38 |
| I5_Isomyia_pubera_M_SouthAfrica | 23 | 23 | 23 | 16 | 11 | 11 |
| I6_Isomyia_sp3_cf_cuthbertsoni_F_SouthAfrica | 62 | 62 | 62 | 49 | 38 | 38 |
| I8_Isomyia_distinguenda_M_SouthAfrica | 63 | 63 | 63 | 47 | 38 | 38 |
| I9_Isomyia_sp3_cf_cuthbertsoni_F_SouthAfrica | 62 | 62 | 62 | 49 | 38 | 38 |
| K3_Fainia_albitarsis_F_Tanzania | 46 | 46 | 46 | 38 | 30 | 30 |
| K4_Rhinia_sp9_F_Tanzania | 64 | 64 | 64 | 50 | 2 | 2 |
| K5_Albaredaya_malgache_F_Madagascar | 32 | 32 | 32 | 24 | 17 | 17 |
| K7_Fainia_albitarsis_F_Tanzania | 46 | 46 | 46 | 38 | 30 | 30 |
| R12_Rhinia_apicalis_F_Namibia | 3 | 3 | 3 | 2 | 2 | 2 |
| R18_Rhinia_apicalis_M_Togo | 3 | 3 | 3 | 2 | 2 | 2 |
| R2_Rhinia_sp2_F_Burundi | 65 | 65 | 65 | 51 | 40 | 40 |
| R24_Rhinia_apicalis_F_Namibia | 2 | 2 | 2 | 2 | 2 | 2 |
| R3_Rhinia_coxendix_M_DRCongo | 66 | 66 | 66 | 52 | 2 | 2 |
| S13_Stomorhina_rugosa_M_Malawi | 11 | 11 | 11 | 7 | 5 | 5 |
| S14_Stomorhina_rugosa_F_Ethiopia | 67 | 67 | 67 | 7 | 5 | 5 |
| S16_Stomorhina_guttata_F_SouthAfrica | 28 | 28 | 28 | 20 | 14 | 14 |
| S17_Stomorhina_guttata_M_SouthAfrica | 28 | 28 | 28 | 20 | 14 | 14 |
| S19_Stomorhina_apta_F_Burundi | 68 | 68 | 68 | 53 | 41 | 41 |
| S2_Stomorhina_chapini_F_DRCongo | 5 | 5 | 5 | 3 | 3 | 3 |
| S20_Stomorhina_apta_F_Burundi | 68 | 68 | 68 | 53 | 41 | 41 |
| S21_Stomorhina_lunata_F_Mauritius | 19 | 19 | 19 | 13 | 9 | 9 |
| S22_Stomorhina_lunata_M_Mauritius | 19 | 19 | 19 | 13 | 9 | 9 |
| S23_Stomorhina_lunata_M_Malawi | 19 | 19 | 19 | 13 | 9 | 9 |
| S24_Stomorhina_lunata_F_Malawi | 19 | 19 | 19 | 13 | 9 | 9 |
| S26_Stomorhina_lunata_M_SouthAfrica | 19 | 19 | 19 | 13 | 9 | 9 |
| S28_Stomorhina_cf_malobana_F_Malawi | 69 | 69 | 69 | 54 | 41 | 41 |
| S3_Stomorhina_chapini_F_SouthAfrica | 70 | 70 | 70 | 55 | 42 | 42 |
| S30_Stomorhina_sp2_cf_malobana_F_Malawi | 69 | 69 | 69 | 54 | 41 | 41 |
| S31_Stomorhina_malobana_M_Malawi | 69 | 69 | 69 | 54 | 41 | 41 |
| S4_Stomorhina_chapini_M_SouthAfrica | 71 | 71 | 71 | 56 | 42 | 42 |
| S40_Stomorhina_sp2_cf_malobana_F_Tanzania | 69 | 69 | 69 | 54 | 41 | 41 |
| S42_Stomorhina_sp2_cf_malobana_F_Tanzania | 69 | 69 | 69 | 54 | 41 | 41 |
| S45_Stomorhina_cribrata_M_Tanzania | 1 | 1 | 1 | 1 | 1 | 1 |
| S5_Stomorhina_sp1_cf_armatipes_F_SouthAfrica | 72 | 72 | 72 | 13 | 9 | 9 |
| S7_Stomorhina_lunata_F_SouthAfrica | 19 | 19 | 19 | 13 | 9 | 9 |
| S8_Stomorhina_sp1_cf_armatipes_F_SouthAfrica | 72 | 72 | 72 | 13 | 9 | 9 |
| S9_Stomorhina_armatipes_M_SouthAfrica | 72 | 72 | 72 | 13 | 9 | 9 |
| T1_Thoracites_sp1_M_SouthAfrica | 73 | 73 | 73 | 57 | 43 | 43 |
| T3_Thoracites_sp1_F_SouthAfrica | 74 | 74 | 74 | 57 | 43 | 43 |
| T6_Thoracites_petersiana_F_SouthAfrica | 75 | 75 | 75 | 58 | 44 | 44 |
| T7_Thoracites_petersiana_M_SouthAfrica | 75 | 75 | 75 | 58 | 44 | 44 |
| USA01_Cosmina_fuscipennis_F_SouthAfrica | 76 | 76 | 76 | 33 | 26 | 26 |
| USA02_Cosmina_fuscipennis_M_SouthAfrica | 76 | 76 | 76 | 33 | 26 | 26 |
| USA03_Fainia_albitarsis_M_Kenya | 46 | 46 | 46 | 38 | 30 | 30 |
| USA04_Fainia_albitarsis_F_Kenya | 46 | 46 | 46 | 38 | 30 | 30 |
| USA05_Isomyia_nataliensis_F_SouthAfrica | 27 | 27 | 27 | 19 | 13 | 13 |
| USA06_Isomyia_nataliensis_M_SouthAfrica | 27 | 27 | 27 | 19 | 13 | 13 |
| USA07_Isomyia_pubera_F_SouthAfrica | 23 | 23 | 23 | 16 | 11 | 11 |
| USA08_Isomyia_natalensis_F_SouthAfrica | 27 | 27 | 27 | 19 | 13 | 13 |
| USA09_Isomyia_tristis_M_SouthAfrica | 24 | 24 | 24 | 17 | 10 | 10 |
| USA10_Isomyia_tristis_F_SouthAfrica | 20 | 20 | 20 | 14 | 10 | 10 |
| USA11_Rhiniaa_apicalis_F_Kenya | 2 | 2 | 2 | 2 | 2 | 2 |
| USA12_Rhyncomya_minutalis_F_SouthAfrica | 77 | 77 | 77 | 59 | 45 | 45 |
| USA13_Rhyncomya_soyauxi_F_Kenya | 78 | 78 | 78 | 60 | 46 | 46 |
| USA14_Rhyncomya_soyauxi_M_Kenya | 79 | 79 | 79 | 60 | 46 | 46 |
| USA15_Rhyncomya_soyauxi_F_Kenya | 79 | 79 | 79 | 60 | 46 | 46 |
| USA16_Rhyncomya_soyauxi_M_Kenya | 80 | 80 | 80 | 61 | 47 | 47 |
| USA18_Stomorhina_lunata_F_SouthAfrica | 19 | 19 | 19 | 13 | 9 | 9 |
| USA19_Stomorhina_lunata_M_SouthAfrica | 19 | 19 | 19 | 13 | 9 | 9 |
| Y10_Zumba_antennalis_F_SouthAfrica | 81 | 81 | 81 | 62 | 48 | 48 |
| Y13_Rhyncomya_sp16_cf_minutalis_M_SouthAfrica | 82 | 82 | 82 | 59 | 45 | 45 |
| Y15_Rhyncomya_forcipata_F_SouthAfrica | 83 | 83 | 83 | 63 | 49 | 49 |
| Y20_Rhyncomya_maculata_M_SouthAfrica | 84 | 84 | 84 | 64 | 45 | 45 |
| Y23_Rhyncomya_pruinosa_M_SouthAfrica | 85 | 85 | 85 | 65 | 50 | 50 |
| Y24_Rhyncomya_pruinosa_M_Malawi | 85 | 85 | 85 | 65 | 50 | 50 |
| Y25_Rhyncomya_pruinosa_F_Kenya | 86 | 86 | 86 | 65 | 50 | 50 |
| Y26_Rhyncomya_pruinosa_F_SouthAfrica | 85 | 85 | 85 | 65 | 50 | 50 |
| Y27_Rhyncomya_forcipata_F_SouthAfrica | 87 | 87 | 87 | 63 | 49 | 49 |
| Y28_Rhyncomya_forcipata_F_SouthAfrica | 83 | 83 | 83 | 63 | 49 | 49 |
| Y29_Rhyncomya_forcipata_M_Namibia | 83 | 83 | 83 | 63 | 49 | 49 |
| Y32_Rhyncomya_minutalis_F_SouthAfrica | 88 | 88 | 88 | 66 | 45 | 45 |
| Y35_Rhyncomya_cassotis_M_Malawi | 89 | 89 | 89 | 67 | 51 | 51 |
| Y36_Rhyncomya_sp11_cf_cassotis_M_Togo | 90 | 90 | 90 | 68 | 51 | 51 |
| Y37_Rhyncomya_sp12_cf_cassotis_M_Zambia | 91 | 91 | 91 | 69 | 51 | 51 |
| Y38_Rhyncomya_cassotis_F_Malawi | 92 | 92 | 92 | 67 | 51 | 51 |
| Y39_Rhyncomya_cassotis_F_Namibia | 93 | 93 | 93 | 69 | 51 | 51 |
| Y40_Rhyncomya_sp3_F_SouthAfrica | 94 | 94 | 94 | 70 | 52 | 52 |
| Y41_Rhyncomya_sp1_F_Cameroon | 95 | 95 | 95 | 68 | 51 | 51 |
| Y44_Rhyncomya_sp8_F_Namibia | 96 | 96 | 96 | 71 | 53 | 53 |
| Y46_Rhyncomya_paratristis_F_SouthAfrica | 97 | 97 | 97 | 72 | 54 | 54 |
| Y47_Rhyncomya_dasyops_F_SouthAfrica | 98 | 98 | 98 | 73 | 55 | 55 |
| Y56_Rhyncomya_sp19_F_Cameroon | 99 | 99 | 99 | 74 | 56 | 56 |
| Y57_Rhyncomya_sp3_F_SouthAfrica | 94 | 94 | 94 | 70 | 52 | 52 |
| Y58_Rhyncomya_trispina_M_Namibia | 100 | 100 | 100 | 75 | 57 | 57 |
| Y59_Rhyncomya_trispina_F_SouthAfrica | 101 | 101 | 101 | 75 | 57 | 57 |
| Y6_Rhyncomya_sp15_F_SouthAfrica | 102 | 102 | 102 | 76 | 58 | 58 |
| Y64_Rhyncomya_sp6_M_Togo | 103 | 103 | 103 | 77 | 59 | 59 |
| Y65_Rhyncomya_sp6_F_Togo | 103 | 103 | 103 | 77 | 59 | 59 |
| Y67_Rhyncomya_sp7_M_SouthAfrica | 104 | 104 | 104 | 78 | 45 | 45 |
| Y69_Rhyncomya_interclusa_M_SouthAfrica | 35 | 35 | 35 | 27 | 20 | 20 |
| Z2_Zumba_antennalis_F_SouthAfrica | 105 | 105 | 105 | 62 | 48 | 48 |
