# Supplementary material for: DNA barcoding of Afrotropical nose flies (Diptera, Calliphoridae, Rhiniinae): species identification, female-male morphotype association, and reference library development
Source: Zookeys. 2026 Jul 3;1284:149–83. doi: 10.3897/zookeys.1284.189450 (PMC13354976; doi:10.3897/zookeys.1284.189450)
Supplement: Supplementary material 10 — Results of ASAP with Distance K80_Kimura and ABGD with Distance K80 Kimura [file zookeys-1284-149_article-189450__-s010.docx]

**Suppl. Material 10** – Results of ASAP with Distance K80_Kimura and ABGD with Distance K80 Kimura / MinSlope = 0.900000 for a dataset of 1,388 COI DNA barcode sequences of Rhiniinae sequences studied, downloaded from GenBank and the BOLD Systems platforms (accessed in November 2023). For the ABGD analysis (Model = K80), partition five proposed 109 species groups (p = 0.007743, BG = 0.018%), partition six proposed 94 species groups (p = 0.012915, BG = 0.026%), and partition seven proposed 89 species groups (p = 0.021544, BG = 0.031%); and the ASAP analysis (Model = K80; ASAP score = 2.50) proposed 92 species groups.

**- ASAP -**

20250228_Rhiniiae_all_data_1384.fas.res.cvs

| Partition rank | NbSubset | Asap score | p-val | pval-rank | W | W rank | Treshold distance |
| --- | --- | --- | --- | --- | --- | --- | --- |
| 1 | 92 | 1.5 | 7.98E-03 | 2 | 0.000006 | 1 | 0.027077 |
| 2 | 94 | 5 | 3.15E-02 | 3 | 0.000004 | 7 | 0.025437 |
| 3 | 88 | 7 | 3.46E-02 | 5 | 0.000004 | 9 | 0.030924 |
| 4 | 84 | 9 | 1.28E-03 | 1 | 0.000002 | 17 | 0.031727 |
| 5 | 96 | 10.5 | 2.14E-01 | 13 | 0.000004 | 8 | 0.024394 |
| 6 | 90 | 11.5 | 3.45E-01 | 21 | 0.000006 | 2 | 0.029501 |
| 7 | 89 | 15.5 | 3.35E-01 | 20 | 0.000003 | 11 | 0.030309 |
| 8 | 105 | 17.5 | 3.49E-02 | 6 | 0.000002 | 29 | 0.019049 |
| 9 | 57 | 20.5 | 3.59E-02 | 7 | 0.000001 | 34 | 0.047232 |
| 10 | 55 | 22 | 5.28E-02 | 8 | 0.000001 | 36 | 0.047888 |
| 11 | 82 | 24 | 2.40E-01 | 15 | 0.000001 | 33 | 0.033633 |
| 12 | 103 | 29.5 | 5.35E-01 | 33 | 0.000002 | 26 | 0.019698 |
| 13 | 91 | 29.5 | 7.33E-01 | 56 | 0.000006 | 3 | 0.028522 |
| 14 | 99 | 30 | 5.79E-01 | 38 | 0.000002 | 22 | 0.021592 |
| 15 | 87 | 30 | 6.95E-01 | 50 | 0.000004 | 10 | 0.031098 |
| 16 | 63 | 33.5 | 1.62E-01 | 11 | 0.000001 | 56 | 0.044319 |
| 17 | 107 | 33.5 | 5.15E-01 | 32 | 0.000001 | 35 | 0.018549 |
| 18 | 222 | 37 | 5.11E-01 | 31 | 0.000001 | 43 | 0.003525 |
| 19 | 109 | 38 | 6.49E-01 | 45 | 0.000001 | 31 | 0.01758 |
| 20 | 104 | 38.5 | 7.19E-01 | 53 | 0.000002 | 24 | 0.019231 |
| 21 | 175 | 38.5 | 7.56E-01 | 59 | 0.000002 | 18 | 0.005346 |
| 22 | 97 | 40 | 7.96E-01 | 67 | 0.000003 | 13 | 0.023746 |
| 23 | 113 | 41 | 3.71E-01 | 22 | 0.000001 | 60 | 0.01548 |
| 24 | 81 | 41 | 6.37E-01 | 44 | 0.000001 | 38 | 0.034731 |
| 25 | 2 | 41 | 8.26E-01 | 77 | 0.000005 | 5 | 0.13271 |
| 26 | 108 | 41.5 | 6.97E-01 | 51 | 0.000001 | 32 | 0.018429 |
| 27 | 79 | 42 | 2.93E-01 | 18 | 0.000001 | 66 | 0.035918 |
| 28 | 83 | 42.5 | 7.64E-01 | 60 | 0.000002 | 25 | 0.032385 |
| 29 | 118 | 44 | 1.31E-01 | 10 | 0.000001 | 78 | 0.013806 |
| 30 | 106 | 44 | 7.50E-01 | 58 | 0.000002 | 30 | 0.018797 |
| 31 | 121 | 47.5 | 3.21E-02 | 4 | 0 | 91 | 0.012357 |
| 32 | 86 | 49.5 | 8.72E-01 | 87 | 0.000003 | 12 | 0.031241 |
| 33 | 93 | 52.5 | 9.08E-01 | 101 | 0.000005 | 4 | 0.02599 |
| 34 | 101 | 58 | 8.72E-01 | 88 | 0.000002 | 28 | 0.020228 |
| 35 | 357 | 58.5 | 6.79E-01 | 47 | 0.000001 | 70 | 0.001804 |
| 36 | 80 | 58.5 | 8.38E-01 | 78 | 0.000001 | 39 | 0.035051 |
| 37 | 77 | 59 | 5.41E-01 | 35 | 0.000001 | 83 | 0.037144 |
| 38 | 124 | 60.5 | 4.63E-01 | 25 | 0 | 96 | 0.011976 |
| 39 | 352 | 63 | 7.82E-01 | 63 | 0.000001 | 63 | 0.001852 |
| 40 | 59 | 63 | 8.62E-01 | 84 | 0.000001 | 42 | 0.046077 |
| 41 | 119 | 63.5 | 6.09E-01 | 41 | 0.000001 | 86 | 0.013749 |
| 42 | 67 | 64.5 | 7.13E-01 | 52 | 0.000001 | 77 | 0.042836 |
| 43 | 85 | 64.5 | 9.28E-01 | 115 | 0.000003 | 14 | 0.031376 |
| 44 | 122 | 65 | 5.47E-01 | 36 | 0 | 94 | 0.012235 |
| 45 | 127 | 67.5 | 5.37E-01 | 34 | 0 | 101 | 0.010806 |
| 46 | 112 | 67.5 | 8.12E-01 | 74 | 0.000001 | 61 | 0.015599 |
| 47 | 95 | 69 | 9.48E-01 | 132 | 0.000004 | 6 | 0.024877 |
| 48 | 114 | 71.5 | 8.14E-01 | 75 | 0.000001 | 68 | 0.015361 |
| 49 | 134 | 74 | 4.89E-01 | 27 | 0 | 121 | 0.009298 |
| 50 | 3 | 75 | 9.08E-01 | 100 | 0.000001 | 50 | 0.101066 |
| 51 | 116 | 76 | 8.46E-01 | 79 | 0.000001 | 73 | 0.014208 |
| 52 | 363 | 77 | 8.81E-02 | 9 | 0 | 145 | 0.00171 |
| 53 | 130 | 78.5 | 7.35E-01 | 57 | 0 | 100 | 0.010391 |
| 54 | 100 | 81 | 9.54E-01 | 139 | 0.000002 | 23 | 0.020931 |
| 55 | 68 | 82.5 | 8.70E-01 | 86 | 0.000001 | 79 | 0.042343 |
| 56 | 139 | 84.5 | 4.95E-01 | 29 | 0 | 140 | 0.008389 |
| 57 | 174 | 84.5 | 7.84E-01 | 64 | 0 | 105 | 0.005567 |
| 58 | 142 | 85.5 | 6.83E-01 | 49 | 0 | 122 | 0.007748 |
| 59 | 102 | 86.5 | 9.60E-01 | 146 | 0.000002 | 27 | 0.020058 |
| 60 | 350 | 86.5 | 9.72E-01 | 157 | 0.000003 | 16 | 0.001875 |
| 61 | 61 | 87 | 9.32E-01 | 119 | 0.000001 | 55 | 0.044817 |
| 62 | 358 | 87.5 | 9.10E-01 | 106 | 0.000001 | 69 | 0.001764 |
| 63 | 219 | 88 | 9.72E-01 | 156 | 0.000002 | 20 | 0.003601 |
| 64 | 132 | 91 | 7.25E-01 | 54 | 0 | 128 | 0.009629 |
| 65 | 376 | 91.5 | 3.27E-01 | 19 | 0 | 164 | 0.001645 |
| 66 | 156 | 92 | 6.27E-01 | 43 | 0 | 141 | 0.006383 |
| 67 | 221 | 92 | 9.58E-01 | 144 | 0.000001 | 40 | 0.003538 |
| 68 | 74 | 92.5 | 8.86E-01 | 95 | 0 | 90 | 0.038399 |
| 69 | 353 | 93 | 9.18E-01 | 111 | 0.000001 | 75 | 0.001837 |
| 70 | 154 | 94.5 | 7.27E-01 | 55 | 0 | 134 | 0.006636 |
| 71 | 148 | 94.5 | 8.04E-01 | 71 | 0 | 118 | 0.00725 |
| 72 | 66 | 94.5 | 9.24E-01 | 113 | 0.000001 | 76 | 0.043246 |
| 73 | 117 | 94.5 | 9.42E-01 | 125 | 0.000001 | 64 | 0.013957 |
| 74 | 65 | 95 | 9.48E-01 | 131 | 0.000001 | 59 | 0.043661 |
| 75 | 172 | 95.5 | 6.21E-01 | 42 | 0 | 149 | 0.005861 |
| 76 | 76 | 96.5 | 9.16E-01 | 109 | 0.000001 | 84 | 0.037511 |
| 77 | 60 | 96.5 | 9.68E-01 | 152 | 0.000001 | 41 | 0.045301 |
| 78 | 379 | 97 | 4.89E-01 | 28 | 0 | 166 | 0.001621 |
| 79 | 372 | 99.5 | 5.87E-01 | 39 | 0 | 160 | 0.001671 |
| 80 | 168 | 99.5 | 6.51E-01 | 46 | 0 | 153 | 0.006147 |
| 81 | 111 | 100 | 9.60E-01 | 147 | 0.000001 | 53 | 0.015858 |
| 82 | 155 | 102.5 | 7.80E-01 | 62 | 0 | 143 | 0.006522 |
| 83 | 675 | 103 | 2.34E-01 | 14 | 0 | 192 | 0.001515 |
| 84 | 138 | 103.5 | 8.74E-01 | 90 | 0 | 117 | 0.008693 |
| 85 | 227 | 104.5 | 8.50E-01 | 80 | 0 | 129 | 0.003449 |
| 86 | 73 | 104.5 | 9.32E-01 | 120 | 0.000001 | 89 | 0.039289 |
| 87 | 123 | 105.5 | 9.28E-01 | 116 | 0 | 95 | 0.012206 |
| 88 | 75 | 106.5 | 9.46E-01 | 128 | 0.000001 | 85 | 0.03777 |
| 89 | 140 | 107 | 8.72E-01 | 89 | 0 | 125 | 0.00802 |
| 90 | 115 | 107 | 9.58E-01 | 142 | 0.000001 | 72 | 0.014822 |
| 91 | 672 | 108.5 | 4.77E-01 | 26 | 0 | 191 | 0.001519 |
| 92 | 128 | 109.5 | 9.28E-01 | 117 | 0 | 102 | 0.010768 |
| 93 | 58 | 111.5 | 9.84E-01 | 174 | 0.000001 | 49 | 0.046725 |
| 94 | 51 | 112 | 9.86E-01 | 178 | 0.000001 | 46 | 0.049037 |
| 95 | 141 | 112.5 | 9.04E-01 | 99 | 0 | 126 | 0.007797 |
| 96 | 193 | 114 | 2.85E-01 | 17 | 0 | 211 | 0.004652 |
| 97 | 160 | 114 | 7.98E-01 | 69 | 0 | 159 | 0.006266 |
| 98 | 62 | 114.5 | 9.84E-01 | 175 | 0.000001 | 54 | 0.044536 |
| 99 | 373 | 115.5 | 7.98E-01 | 70 | 0 | 161 | 0.001667 |

20250228_Rhiniiae_all_data_1384.fas.scores.png
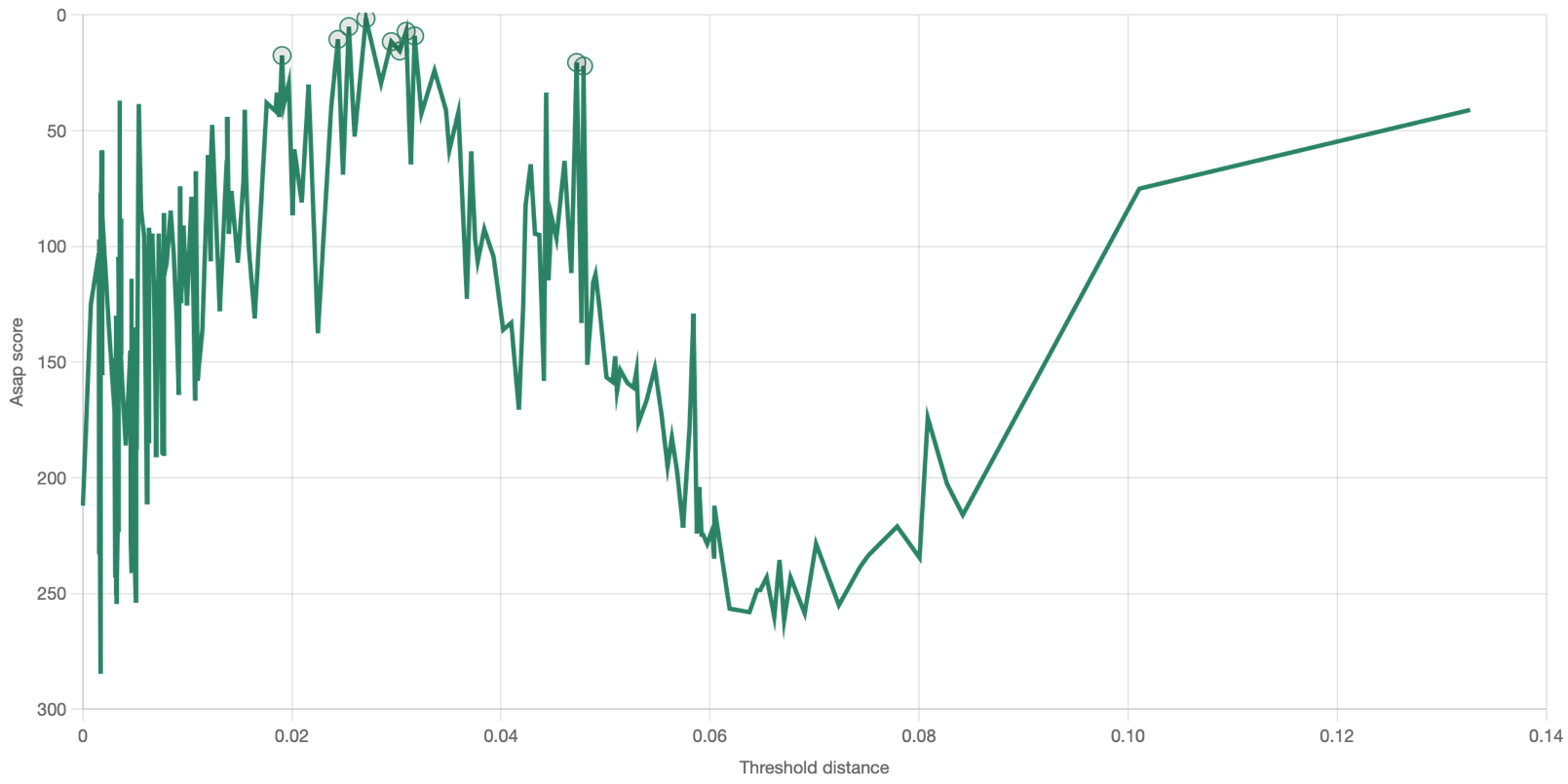


begin spart;

Project_name = 20250228_Rhiniiae_all_data_1384.fas;

Date = 2025-03-13T21:43:10;

N_spartitions = 10 : 20250228_Rhiniiae_all_data_1384.fas_asap_1,17.5 / 20250228_Rhiniiae_all_data_1384.fas_asap_2,10.5 / 20250228_Rhiniiae_all_data_1384.fas_asap_3,5.0 / 20250228_Rhiniiae_all_data_1384.fas_asap_4,1.5 / 20250228_Rhiniiae_all_data_1384.fas_asap_5,11.5 / 20250228_Rhiniiae_all_data_1384.fas_asap_6,15.5 / 20250228_Rhiniiae_all_data_1384.fas_asap_7,7.0 / 20250228_Rhiniiae_all_data_1384.fas_asap_8,9.0 / 20250228_Rhiniiae_all_data_1384.fas_asap_9,20.5 / 20250228_Rhiniiae_all_data_1384.fas_asap_10,22.0;

N_individuals = 1384 / 1384 / 1384 / 1384 / 1384 / 1384 / 1384 / 1384 / 1384 / 1384;

N_subsets = 105 : 8.16e-02,6.91e-01,5.08e-01,?,?,?,7.37e-01,?,2.01e-01,9.58e-01,?,?,4.92e-01,8.15e-01,5.36e-01,?,?,?,3.88e-01,?,1.06e-02,7.21e-01,9.96e-01,3.90e-01,?,?,?,?,?,4.78e-01,3.92e-01,?,?,?,?,6.85e-01,?,?,?,?,2.59e-01,4.74e-01,?,5.20e-01,?,4.40e-01,8.17e-01,5.38e-01,8.45e-01,?,?,9.34e-01,9.46e-01,9.80e-01,5.54e-01,7.03e-01,9.88e-01,?,7.43e-01,?,?,6.08e-01,?,?,?,?,1.29e-01,?,?,?,9.38e-01,7.13e-01,9.44e-01,2.31e-01,?,?,?,?,?,?,?,?,?,?,?,?,?,?,?,?,?,6.45e-01,?,1.20e-01,?,?,9.30e-01,?,?,?,?,?,?,?,? / 96 : 2.74e-02,9.54e-01,?,?,2.87e-01,2.01e-01,9.58e-01,?,?,4.92e-01,8.15e-01,5.36e-01,?,?,5.66e-01,?,1.06e-02,5.80e-01,9.96e-01,?,?,?,?,?,4.78e-01,3.92e-01,?,?,?,?,6.85e-01,?,?,5.32e-01,2.59e-01,4.74e-01,?,5.20e-01,?,4.40e-01,8.17e-01,5.38e-01,8.45e-01,?,?,9.34e-01,9.46e-01,5.54e-01,7.03e-01,9.88e-01,?,7.43e-01,?,?,6.08e-01,?,?,?,?,1.29e-01,?,?,4.36e-01,9.38e-01,7.13e-01,?,9.44e-01,2.31e-01,?,?,?,?,?,?,?,?,?,?,?,?,?,?,?,6.45e-01,?,1.20e-01,?,?,9.30e-01,?,?,?,?,?,?,? / 94 : 2.74e-02,9.54e-01,5.00e-01,?,2.87e-01,2.01e-01,9.58e-01,?,?,4.92e-01,8.15e-01,5.36e-01,?,?,5.66e-01,?,1.06e-02,5.80e-01,9.96e-01,?,?,?,?,?,4.78e-01,3.92e-01,?,?,?,?,6.85e-01,?,?,5.32e-01,2.59e-01,4.74e-01,?,?,5.20e-01,?,4.40e-01,8.17e-01,5.38e-01,8.45e-01,?,?,9.34e-01,9.46e-01,5.54e-01,7.03e-01,9.88e-01,?,7.43e-01,?,?,6.08e-01,?,?,?,?,1.29e-01,?,?,4.36e-01,9.38e-01,2.07e-01,9.44e-01,2.31e-01,?,?,?,?,?,?,?,?,?,?,?,?,?,?,6.45e-01,?,1.20e-01,?,9.30e-01,?,?,?,?,?,?,? / 92 : 2.74e-02,9.08e-01,5.00e-01,?,2.87e-01,2.01e-01,9.58e-01,?,?,4.92e-01,8.15e-01,5.36e-01,?,?,5.66e-01,?,1.06e-02,5.80e-01,9.96e-01,?,?,?,?,?,4.78e-01,3.92e-01,?,?,?,?,6.85e-01,?,?,5.32e-01,2.59e-01,9.84e-03,?,5.20e-01,?,4.40e-01,8.17e-01,2.07e-01,5.38e-01,8.45e-01,?,?,9.34e-01,9.46e-01,5.54e-01,7.03e-01,9.88e-01,?,7.43e-01,?,?,6.08e-01,?,?,?,?,1.29e-01,?,?,4.36e-01,9.38e-01,9.44e-01,2.31e-01,?,?,?,?,?,?,?,?,?,?,?,?,?,?,6.45e-01,1.20e-01,?,9.30e-01,?,?,?,?,?,?,? / 90 : 2.74e-02,9.08e-01,5.00e-01,?,2.87e-01,2.01e-01,9.58e-01,?,?,4.92e-01,8.15e-01,5.36e-01,7.98e-03,?,?,5.66e-01,?,1.06e-02,5.80e-01,9.96e-01,?,?,?,?,?,4.78e-01,3.92e-01,?,?,1.95e-01,?,6.85e-01,?,?,5.32e-01,2.59e-01,9.84e-03,?,5.20e-01,?,4.40e-01,5.38e-01,8.45e-01,?,?,9.34e-01,9.46e-01,5.54e-01,7.03e-01,9.88e-01,?,7.43e-01,?,?,?,?,?,?,1.29e-01,?,?,4.36e-01,9.38e-01,9.44e-01,2.31e-01,?,?,?,?,?,?,?,?,?,?,?,?,?,?,6.45e-01,1.20e-01,?,9.30e-01,?,?,?,?,?,?,? / 89 : 2.74e-02,9.08e-01,5.00e-01,?,2.87e-01,2.01e-01,9.58e-01,?,?,4.92e-01,8.15e-01,3.45e-01,?,?,?,5.66e-01,?,1.06e-02,5.80e-01,9.96e-01,?,?,?,?,?,4.78e-01,3.92e-01,?,?,1.95e-01,?,6.85e-01,?,?,5.32e-01,2.59e-01,9.84e-03,?,5.20e-01,?,4.40e-01,5.38e-01,8.45e-01,?,?,9.34e-01,9.46e-01,5.54e-01,7.03e-01,9.88e-01,?,7.43e-01,?,?,?,?,?,?,1.29e-01,?,?,4.36e-01,9.38e-01,9.44e-01,2.31e-01,?,?,?,?,?,?,?,?,?,?,?,?,?,?,6.45e-01,1.20e-01,?,9.30e-01,?,?,?,?,?,? / 88 : 2.74e-02,9.08e-01,5.00e-01,?,2.87e-01,2.01e-01,9.58e-01,?,?,4.92e-01,8.15e-01,3.37e-01,?,?,5.66e-01,?,1.06e-02,5.80e-01,9.96e-01,?,?,?,?,?,4.78e-01,3.92e-01,?,?,1.95e-01,?,6.85e-01,?,?,5.32e-01,2.59e-01,5.54e-01,9.84e-03,?,5.20e-01,?,4.40e-01,5.38e-01,8.45e-01,?,?,9.34e-01,9.46e-01,7.03e-01,9.88e-01,?,7.43e-01,?,?,?,?,?,?,1.29e-01,?,?,4.36e-01,9.38e-01,9.44e-01,2.31e-01,?,?,?,?,?,?,?,?,?,?,?,?,?,?,6.45e-01,1.20e-01,?,9.30e-01,?,?,?,?,?,? / 84 : 2.74e-02,9.08e-01,5.00e-01,?,2.87e-01,2.01e-01,9.34e-01,8.71e-01,?,?,4.92e-01,8.15e-01,3.37e-01,?,?,9.28e-01,?,1.06e-02,6.95e-01,9.96e-01,?,?,?,?,?,4.78e-01,3.92e-01,?,?,1.95e-01,?,6.85e-01,?,?,9.84e-03,?,5.20e-01,?,4.40e-01,5.38e-01,8.45e-01,?,?,9.46e-01,7.03e-01,9.88e-01,?,7.43e-01,?,?,?,?,?,?,1.29e-01,?,?,4.36e-01,9.38e-01,9.44e-01,2.31e-01,?,?,?,?,?,?,?,?,?,?,?,?,?,6.45e-01,1.20e-01,?,9.30e-01,?,?,?,?,?,? / 57 : 9.84e-01,5.00e-01,?,8.71e-01,?,?,4.92e-01,?,?,9.76e-01,?,4.78e-01,3.86e-01,?,?,1.95e-01,?,?,8.45e-01,5.20e-01,8.27e-01,4.40e-01,5.38e-01,7.43e-01,8.45e-01,?,?,9.88e-01,?,?,?,?,?,?,1.29e-01,?,1.06e-01,9.38e-01,9.44e-01,2.31e-01,?,?,?,?,?,?,?,?,?,?,6.45e-01,1.20e-01,?,?,?,?,? / 55 : 9.98e-01,5.00e-01,?,8.71e-01,?,?,4.92e-01,?,?,9.76e-01,9.44e-01,?,4.78e-01,3.86e-01,?,?,1.95e-01,?,?,8.45e-01,5.20e-01,8.27e-01,4.40e-01,3.59e-02,?,?,9.88e-01,?,?,?,?,?,?,1.29e-01,?,1.06e-01,9.38e-01,2.31e-01,?,?,?,?,?,?,?,?,?,?,6.45e-01,1.20e-01,?,?,?,?, ? ;

[Generated by ASAP with Distance K80_Kimura ]

[WARNING: The sample names below may have been changed to fit SPART specification (only alphanumeric characters and _ )]

[Subset scores are p-values; see Puillandre et al. 2021 for details of the algorithm]

| **N_spartitions (10)** | **fas_asap_1** | **fas_asap_2** | **fas_asap_3** | **fas_asap_4** | **fas_asap_5** | **fas_asap_6** | **fas_asap_7** | **fas_asap_8** | **fas_asap_9** | **fas_asap_10** |
| --- | --- | --- | --- | --- | --- | --- | --- | --- | --- | --- |
| **Spartition_score_type, Asap-Score** | **17.5** | **10.5** | **5** | **1.5** | **11.5** | **15.5** | **7** | **9** | **20.5** | **22** |
| **N_individuals** | **1384** | **1384** | **1384** | **1384** | **1384** | **1384** | **1384** | **1384** | **1384** | **1384** |
| **N_subsets** | **105** | **96** | **94** | **92** | **90** | **89** | **88** | **84** | **57** | **55** |
| **p-value** | **3.49E-02** | **2.14E-01** | **3.15E-02** | **7.98E-03** | **3.45E-01** | **3.35E-01** | **3.46E-02** | **1.28E-03** | **3.59E-02** | **5.28E-02** |
| 1279A01_Stomorhina_cribrata_F_Ghana | 1 | 1 | 1 | 1 | 1 | 1 | 1 | 1 | 1 | 1 |
| 1279C06_Stomorhina_cribrata_M_Togo | 1 | 1 | 1 | 1 | 1 | 1 | 1 | 1 | 1 | 1 |
| 1279D01_Stomorhina_cribrata_F_Togo | 1 | 1 | 1 | 1 | 1 | 1 | 1 | 1 | 1 | 1 |
| 1279D02_Stomorhina_cribrata_M_Togo | 1 | 1 | 1 | 1 | 1 | 1 | 1 | 1 | 1 | 1 |
| 1282C02_Stomorhina_cribrata_M_SouthAfrica | 1 | 1 | 1 | 1 | 1 | 1 | 1 | 1 | 1 | 1 |
| 1306B06_Stomorhina_cribrata_F_SouthAfrica | 1 | 1 | 1 | 1 | 1 | 1 | 1 | 1 | 1 | 1 |
| 1306C03_Stomorhina_cribrata_M_SouthAfrica | 1 | 1 | 1 | 1 | 1 | 1 | 1 | 1 | 1 | 1 |
| 1306E02_Stomorhina_cribrata_F_SouthAfrica | 1 | 1 | 1 | 1 | 1 | 1 | 1 | 1 | 1 | 1 |
| 1306F06_Stomorhina_cribrata_F_SouthAfrica | 1 | 1 | 1 | 1 | 1 | 1 | 1 | 1 | 1 | 1 |
| 1306F07_Stomorhina_cribrata_F_SouthAfrica | 1 | 1 | 1 | 1 | 1 | 1 | 1 | 1 | 1 | 1 |
| 1306F08_Stomorhina_cribrata_F_SouthAfrica | 1 | 1 | 1 | 1 | 1 | 1 | 1 | 1 | 1 | 1 |
| KMPDE165_19_Rhiniinae | 1 | 1 | 1 | 1 | 1 | 1 | 1 | 1 | 1 | 1 |
| S45_Stomorhina_cribrata_M_Tanzania | 1 | 1 | 1 | 1 | 1 | 1 | 1 | 1 | 1 | 1 |
| 1279E01_Stomorhina_cribrata_F_Togo | 1 | 1 | 1 | 1 | 1 | 1 | 1 | 1 | 1 | 1 |
| GMEGD012_14_Rhiniinae | 1 | 1 | 1 | 1 | 1 | 1 | 1 | 1 | 1 | 1 |
| 1306F05_Stomorhina_cribrata_F_SouthAfrica | 1 | 1 | 1 | 1 | 1 | 1 | 1 | 1 | 1 | 1 |
| 1279D05_Stomorhina_rugosa_M_Togo | 2 | 1 | 1 | 1 | 1 | 1 | 1 | 1 | 1 | 1 |
| ETKD757_12_Rhiniinae | 2 | 1 | 1 | 1 | 1 | 1 | 1 | 1 | 1 | 1 |
| ETKD853_13_Rhiniinae | 2 | 1 | 1 | 1 | 1 | 1 | 1 | 1 | 1 | 1 |
| KMPFJ091_19_Rhiniinae | 2 | 1 | 1 | 1 | 1 | 1 | 1 | 1 | 1 | 1 |
| KMPNA1090_18_Rhiniinae | 2 | 1 | 1 | 1 | 1 | 1 | 1 | 1 | 1 | 1 |
| S13_Stomorhina_rugosa_M_Malawi | 2 | 1 | 1 | 1 | 1 | 1 | 1 | 1 | 1 | 1 |
| 1282D01_Stomorhina_rugosa_M_SouthAfrica | 2 | 1 | 1 | 1 | 1 | 1 | 1 | 1 | 1 | 1 |
| 1282D03_Stomorhina_rugosa_F_SouthAfrica | 2 | 1 | 1 | 1 | 1 | 1 | 1 | 1 | 1 | 1 |
| KMPKB001_18_Rhiniinae | 2 | 1 | 1 | 1 | 1 | 1 | 1 | 1 | 1 | 1 |
| KMPKB064_18_Rhiniinae | 2 | 1 | 1 | 1 | 1 | 1 | 1 | 1 | 1 | 1 |
| S14_Stomorhina_rugosa_F_Ethiopia | 2 | 1 | 1 | 1 | 1 | 1 | 1 | 1 | 1 | 1 |
| 1282A01_Stomorhina_lunata_M_SouthAfrica | 3 | 2 | 2 | 2 | 2 | 2 | 2 | 2 | 1 | 1 |
| 1282C01_Stomorhina_lunata_M_SouthAfrica | 3 | 2 | 2 | 2 | 2 | 2 | 2 | 2 | 1 | 1 |
| 1282C03_Stomorhina_lunata_M_SouthAfrica | 3 | 2 | 2 | 2 | 2 | 2 | 2 | 2 | 1 | 1 |
| 1282C08_Stomorhina_lunata_F_SouthAfrica | 3 | 2 | 2 | 2 | 2 | 2 | 2 | 2 | 1 | 1 |
| 1282D02_Stomorhina_lunata_M_SouthAfrica | 3 | 2 | 2 | 2 | 2 | 2 | 2 | 2 | 1 | 1 |
| 1299A02_Stomorhina_lunata_F_SouthAfrica | 3 | 2 | 2 | 2 | 2 | 2 | 2 | 2 | 1 | 1 |
| 1299A03_Stomorhina_lunata_F_SouthAfrica | 3 | 2 | 2 | 2 | 2 | 2 | 2 | 2 | 1 | 1 |
| 1306A01_Stomorhina_lunata_F_SouthAfrica | 3 | 2 | 2 | 2 | 2 | 2 | 2 | 2 | 1 | 1 |
| 1306C01_Stomorhina_lunata_M_SouthAfrica | 3 | 2 | 2 | 2 | 2 | 2 | 2 | 2 | 1 | 1 |
| 1306D03_Stormohina_lunata_F_SouthAfrica | 3 | 2 | 2 | 2 | 2 | 2 | 2 | 2 | 1 | 1 |
| 1306D05_Stomorhina_lunata_F_SouthAfrica | 3 | 2 | 2 | 2 | 2 | 2 | 2 | 2 | 1 | 1 |
| GMGMN1228_14_Stomorhina_lunata | 3 | 2 | 2 | 2 | 2 | 2 | 2 | 2 | 1 | 1 |
| KP004766_1_9_658_Stomorhina_lunata | 3 | 2 | 2 | 2 | 2 | 2 | 2 | 2 | 1 | 1 |
| KY749786_1_42_635_Stomorhina_lunata | 3 | 2 | 2 | 2 | 2 | 2 | 2 | 2 | 1 | 1 |
| MN868811_1_1_650_Stomorhina_lunata | 3 | 2 | 2 | 2 | 2 | 2 | 2 | 2 | 1 | 1 |
| S21_Stomorhina_lunata_F_Mauritius | 3 | 2 | 2 | 2 | 2 | 2 | 2 | 2 | 1 | 1 |
| S22_Stomorhina_lunata_M_Mauritius | 3 | 2 | 2 | 2 | 2 | 2 | 2 | 2 | 1 | 1 |
| S23_Stomorhina_lunata_M_Malawi | 3 | 2 | 2 | 2 | 2 | 2 | 2 | 2 | 1 | 1 |
| S24_Stomorhina_lunata_F_Malawi | 3 | 2 | 2 | 2 | 2 | 2 | 2 | 2 | 1 | 1 |
| S26_Stomorhina_lunata_M_SouthAfrica | 3 | 2 | 2 | 2 | 2 | 2 | 2 | 2 | 1 | 1 |
| S7_Stomorhina_lunata_F_SouthAfrica | 3 | 2 | 2 | 2 | 2 | 2 | 2 | 2 | 1 | 1 |
| USA18_Stomorhina_lunata_F_SouthAfrica | 3 | 2 | 2 | 2 | 2 | 2 | 2 | 2 | 1 | 1 |
| USA19_Stomorhina_lunata_M_SouthAfrica | 3 | 2 | 2 | 2 | 2 | 2 | 2 | 2 | 1 | 1 |
| 1282A06_Stomorhina_lunata_F_SouthAfrica | 3 | 2 | 2 | 2 | 2 | 2 | 2 | 2 | 1 | 1 |
| 1299A01_Stomorhina_lunata_F_SouthAfrica | 3 | 2 | 2 | 2 | 2 | 2 | 2 | 2 | 1 | 1 |
| 1306B01_Stomorhina_lunata_F_SouthAfrica | 3 | 2 | 2 | 2 | 2 | 2 | 2 | 2 | 1 | 1 |
| S5_Stomorhina_sp1_cf_armatipes_F_SouthAfrica | 3 | 2 | 2 | 2 | 2 | 2 | 2 | 2 | 1 | 1 |
| S8_Stomorhina_sp1_cf_armatipes_F_SouthAfrica | 3 | 2 | 2 | 2 | 2 | 2 | 2 | 2 | 1 | 1 |
| S9_Stomorhina_armatipes_M_SouthAfrica | 3 | 2 | 2 | 2 | 2 | 2 | 2 | 2 | 1 | 1 |
| GMMDB131_15_Rhiniinae | 4 | 3 | 3 | 3 | 3 | 3 | 3 | 3 | 1 | 1 |
| GMMDC250_15_Rhiniinae | 4 | 3 | 3 | 3 | 3 | 3 | 3 | 3 | 1 | 1 |
| GMMDD078_15_Rhiniinae | 4 | 3 | 3 | 3 | 3 | 3 | 3 | 3 | 1 | 1 |
| GMMDE039_15_Rhiniinae | 4 | 3 | 3 | 3 | 3 | 3 | 3 | 3 | 1 | 1 |
| GMMDE044_15_Rhiniinae | 4 | 3 | 3 | 3 | 3 | 3 | 3 | 3 | 1 | 1 |
| GMMDE041_15_Rhiniinae | 4 | 3 | 3 | 3 | 3 | 3 | 3 | 3 | 1 | 1 |
| GMMDF170_15_Rhiniinae | 4 | 3 | 3 | 3 | 3 | 3 | 3 | 3 | 1 | 1 |
| GMMDD083_15_Rhiniinae | 4 | 3 | 3 | 3 | 3 | 3 | 3 | 3 | 1 | 1 |
| 1306B08_Stomorhina_guttata_F_SouthAfrica | 5 | 4 | 4 | 4 | 4 | 4 | 4 | 4 | 1 | 1 |
| S17_Stomorhina_guttata_M_SouthAfrica | 5 | 4 | 4 | 4 | 4 | 4 | 4 | 4 | 1 | 1 |
| S16_Stomorhina_guttata_F_SouthAfrica | 5 | 4 | 4 | 4 | 4 | 4 | 4 | 4 | 1 | 1 |
| 1279A02_Rhinia_apicalis_M_Ghana | 6 | 5 | 5 | 5 | 5 | 5 | 5 | 5 | 1 | 1 |
| 1279F06_Rhinia_apicalis_M_Ghana | 6 | 5 | 5 | 5 | 5 | 5 | 5 | 5 | 1 | 1 |
| 1279C08_Rhinia_cf_apicalis_F_Togo | 6 | 5 | 5 | 5 | 5 | 5 | 5 | 5 | 1 | 1 |
| 1279F02_Rhinia_cf_apicalis_F_Ghana | 6 | 5 | 5 | 5 | 5 | 5 | 5 | 5 | 1 | 1 |
| KX054613_1_1_650_Rhiniidae_sp | 6 | 5 | 5 | 5 | 5 | 5 | 5 | 5 | 1 | 1 |
| R24_Rhinia_apicalis_F_Namibia | 6 | 5 | 5 | 5 | 5 | 5 | 5 | 5 | 1 | 1 |
| 1282A08_Rhinia_cf_apicalis_M_SouthAfrica | 6 | 5 | 5 | 5 | 5 | 5 | 5 | 5 | 1 | 1 |
| USA11_Rhiniaa_apicalis_F_Kenya | 6 | 5 | 5 | 5 | 5 | 5 | 5 | 5 | 1 | 1 |
| 1279A03_Rhinia_apicalis_M_Togo | 6 | 5 | 5 | 5 | 5 | 5 | 5 | 5 | 1 | 1 |
| 1279B01_Rhinia_cf_apicalis_F_Togo | 6 | 5 | 5 | 5 | 5 | 5 | 5 | 5 | 1 | 1 |
| 1279B06_Rhinia_cf_apicalis_F_Togo | 6 | 5 | 5 | 5 | 5 | 5 | 5 | 5 | 1 | 1 |
| 1279B08_Rhinia_cf_apicalis_F_Togo | 6 | 5 | 5 | 5 | 5 | 5 | 5 | 5 | 1 | 1 |
| 1279C02_Rhinia_cf_apicalis_F_Togo | 6 | 5 | 5 | 5 | 5 | 5 | 5 | 5 | 1 | 1 |
| 1279C03_Rhinia_cf_apicalis_F_Togo | 6 | 5 | 5 | 5 | 5 | 5 | 5 | 5 | 1 | 1 |
| 1279C04_Rhinia_apicalis_M_Togo | 6 | 5 | 5 | 5 | 5 | 5 | 5 | 5 | 1 | 1 |
| 1279C07_Rhinia_apicalis_M_Togo | 6 | 5 | 5 | 5 | 5 | 5 | 5 | 5 | 1 | 1 |
| 1279E03_Rhinia_apicalis_M_Ghana | 6 | 5 | 5 | 5 | 5 | 5 | 5 | 5 | 1 | 1 |
| 1282B06_Rhinia_apicalis_M_SouthAfrica | 6 | 5 | 5 | 5 | 5 | 5 | 5 | 5 | 1 | 1 |
| KMPJQ074_19_Rhiniinae | 6 | 5 | 5 | 5 | 5 | 5 | 5 | 5 | 1 | 1 |
| R12_Rhinia_apicalis_F_Namibia | 6 | 5 | 5 | 5 | 5 | 5 | 5 | 5 | 1 | 1 |
| R18_Rhinia_apicalis_M_Togo | 6 | 5 | 5 | 5 | 5 | 5 | 5 | 5 | 1 | 1 |
| 1279A04_Rhinia_cf_apicalis_F_Togo | 6 | 5 | 5 | 5 | 5 | 5 | 5 | 5 | 1 | 1 |
| 1279A06_Rhinia_cf_apicalis_F_Togo | 6 | 5 | 5 | 5 | 5 | 5 | 5 | 5 | 1 | 1 |
| 1279E04_Rhinia_cf_apicalis_F_Ghana | 6 | 5 | 5 | 5 | 5 | 5 | 5 | 5 | 1 | 1 |
| ETKD788_13_Rhiniinae | 6 | 5 | 5 | 5 | 5 | 5 | 5 | 5 | 1 | 1 |
| KMPGS994_19_Rhiniinae | 6 | 5 | 5 | 5 | 5 | 5 | 5 | 5 | 1 | 1 |
| KMPHO045_19_Rhiniinae | 6 | 5 | 5 | 5 | 5 | 5 | 5 | 5 | 1 | 1 |
| KMPIQ037_19_Rhiniinae | 6 | 5 | 5 | 5 | 5 | 5 | 5 | 5 | 1 | 1 |
| KMPJV085_19_Rhiniinae | 6 | 5 | 5 | 5 | 5 | 5 | 5 | 5 | 1 | 1 |
| KMPNJ009_19_Rhiniinae | 6 | 5 | 5 | 5 | 5 | 5 | 5 | 5 | 1 | 1 |
| 1279E02_Rhinia_cf_apicalis_F_Togo | 6 | 5 | 5 | 5 | 5 | 5 | 5 | 5 | 1 | 1 |
| 1279B02_Rhinia_sp1_M_Togo | 6 | 5 | 5 | 5 | 5 | 5 | 5 | 5 | 1 | 1 |
| 1279A05_Rhinia_cf_apicalis_F_Togo | 6 | 5 | 5 | 5 | 5 | 5 | 5 | 5 | 1 | 1 |
| 1279B03_Rhinia_cf_apicalis_F_Togo | 6 | 5 | 5 | 5 | 5 | 5 | 5 | 5 | 1 | 1 |
| 1279E08_Rhinia_cf_apicalis_F_Ghana | 6 | 5 | 5 | 5 | 5 | 5 | 5 | 5 | 1 | 1 |
| KMPWO024_19_Rhiniinae | 6 | 5 | 5 | 5 | 5 | 5 | 5 | 5 | 1 | 1 |
| 1279B07_Rhinia_sp3_M_Togo | 6 | 5 | 5 | 5 | 5 | 5 | 5 | 5 | 1 | 1 |
| KMPLJ134_19_Rhinia | 6 | 5 | 5 | 5 | 5 | 5 | 5 | 5 | 1 | 1 |
| KMTTJ070_19_Rhinia | 6 | 5 | 5 | 5 | 5 | 5 | 5 | 5 | 1 | 1 |
| ETKD613_12_Rhiniinae | 6 | 5 | 5 | 5 | 5 | 5 | 5 | 5 | 1 | 1 |
| K4_Rhinia_sp9_F_Tanzania | 6 | 5 | 5 | 5 | 5 | 5 | 5 | 5 | 1 | 1 |
| ETKD1032_13_Rhiniinae | 6 | 5 | 5 | 5 | 5 | 5 | 5 | 5 | 1 | 1 |
| R3_Rhinia_coxendix_M_DRCongo | 6 | 5 | 5 | 5 | 5 | 5 | 5 | 5 | 1 | 1 |
| 1279F05_Rhinia_sp6_F_Ghana | 6 | 5 | 5 | 5 | 5 | 5 | 5 | 5 | 1 | 1 |
| JQ246692_1_42_691_Rhinia_sp | 6 | 5 | 5 | 5 | 5 | 5 | 5 | 5 | 1 | 1 |
| 1279C05_Rhinia_sp8_M_Togo | 6 | 5 | 5 | 5 | 5 | 5 | 5 | 5 | 1 | 1 |
| 1279F03_Rhinia_sp7_F_Ghana | 6 | 5 | 5 | 5 | 5 | 5 | 5 | 5 | 1 | 1 |
| 1282C07_Rhinia_sp5_M_SouthAfrica | 6 | 5 | 5 | 5 | 5 | 5 | 5 | 5 | 1 | 1 |
| ETKD633_12_Rhiniinae | 7 | 5 | 5 | 5 | 5 | 5 | 5 | 5 | 1 | 1 |
| 1279F04_Rhinia_sp4_F_Ghana | 8 | 5 | 5 | 5 | 5 | 5 | 5 | 5 | 1 | 1 |
| R2_Rhinia_sp2_F_Burundi | 9 | 6 | 6 | 5 | 5 | 5 | 5 | 5 | 1 | 1 |
| 1279E07_Stegosoma_vinculatum_M_Ghana | 10 | 7 | 7 | 6 | 6 | 6 | 6 | 6 | 1 | 1 |
| 1279F01_Stegosoma_vinculatum_F_Ghana | 11 | 7 | 7 | 6 | 6 | 6 | 6 | 6 | 1 | 1 |
| G5_Stegosoma_vinculatum_F_SouthAfrica | 11 | 7 | 7 | 6 | 6 | 6 | 6 | 6 | 1 | 1 |
| G7_Stegosoma_vinculatum_M_SouthAfrica | 11 | 7 | 7 | 6 | 6 | 6 | 6 | 6 | 1 | 1 |
| KMPMQ343_19_Stegosoma_vinculatum | 11 | 7 | 7 | 6 | 6 | 6 | 6 | 6 | 1 | 1 |
| MG968160_1_1_650_Stegosoma_vinculatum | 11 | 7 | 7 | 6 | 6 | 6 | 6 | 6 | 1 | 1 |
| ETKD1044_13_Rhiniinae | 12 | 8 | 8 | 7 | 7 | 7 | 7 | 7 | 1 | 1 |
| F20_Fainia_albitarsis_M_Tanzania | 12 | 8 | 8 | 7 | 7 | 7 | 7 | 7 | 1 | 1 |
| K3_Fainia_albitarsis_F_Tanzania | 12 | 8 | 8 | 7 | 7 | 7 | 7 | 7 | 1 | 1 |
| K7_Fainia_albitarsis_F_Tanzania | 12 | 8 | 8 | 7 | 7 | 7 | 7 | 7 | 1 | 1 |
| USA03_Fainia_albitarsis_M_Kenya | 12 | 8 | 8 | 7 | 7 | 7 | 7 | 7 | 1 | 1 |
| USA04_Fainia_albitarsis_F_Kenya | 12 | 8 | 8 | 7 | 7 | 7 | 7 | 7 | 1 | 1 |
| F2_Fainia_elongata_F_DRCongo | 12 | 8 | 8 | 7 | 7 | 7 | 7 | 7 | 1 | 1 |
| F5_Fainia_elongata_M_Malawi | 12 | 8 | 8 | 7 | 7 | 7 | 7 | 7 | 1 | 1 |
| F3_Fainia_inexpectata_F_Malawi | 12 | 8 | 8 | 7 | 7 | 7 | 7 | 7 | 1 | 1 |
| F6_Fainia_inexpectata_M_Kenya | 12 | 8 | 8 | 7 | 7 | 7 | 7 | 7 | 1 | 1 |
| A10_Rhyncomya_sp14_F_Tanzania | 13 | 9 | 9 | 8 | 8 | 8 | 8 | 8 | 1 | 1 |
| GMKMB601_15_Rhiniinae | 13 | 9 | 9 | 8 | 8 | 8 | 8 | 8 | 1 | 1 |
| GMKMY1974_15_Rhiniinae | 13 | 9 | 9 | 8 | 8 | 8 | 8 | 8 | 1 | 1 |
| GMKMB609_15_Rhiniinae | 13 | 9 | 9 | 8 | 8 | 8 | 8 | 8 | 1 | 1 |
| GMKMN026_15_Rhiniinae | 13 | 9 | 9 | 8 | 8 | 8 | 8 | 8 | 1 | 1 |
| GMKMT195_15_Rhiniinae | 13 | 9 | 9 | 8 | 8 | 8 | 8 | 8 | 1 | 1 |
| GMKMH199_15_Rhiniinae | 13 | 9 | 9 | 8 | 8 | 8 | 8 | 8 | 1 | 1 |
| GMKMA582_15_Rhiniinae | 14 | 10 | 10 | 9 | 9 | 8 | 8 | 8 | 1 | 1 |
| GMKMG357_15_Rhiniinae | 14 | 10 | 10 | 9 | 9 | 8 | 8 | 8 | 1 | 1 |
| GMKMW2269_15_Rhiniinae | 14 | 10 | 10 | 9 | 9 | 8 | 8 | 8 | 1 | 1 |
| GMKMT200_15_Rhiniinae | 14 | 10 | 10 | 9 | 9 | 8 | 8 | 8 | 1 | 1 |
| KMPIO155_19_Rhiniinae | 14 | 10 | 10 | 9 | 9 | 8 | 8 | 8 | 1 | 1 |
| KMPAB4626_18_Rhiniinae | 14 | 10 | 10 | 9 | 9 | 8 | 8 | 8 | 1 | 1 |
| KMPAL3593_19_Rhiniinae | 14 | 10 | 10 | 9 | 9 | 8 | 8 | 8 | 1 | 1 |
| KMPCO422_19_Rhiniinae | 14 | 10 | 10 | 9 | 9 | 8 | 8 | 8 | 1 | 1 |
| KMPDJ188_19_Rhiniinae | 14 | 10 | 10 | 9 | 9 | 8 | 8 | 8 | 1 | 1 |
| KMPFR135_19_Rhiniinae | 14 | 10 | 10 | 9 | 9 | 8 | 8 | 8 | 1 | 1 |
| KMPFS033_19_Rhiniinae | 14 | 10 | 10 | 9 | 9 | 8 | 8 | 8 | 1 | 1 |
| KMPGT039_19_Rhiniinae | 14 | 10 | 10 | 9 | 9 | 8 | 8 | 8 | 1 | 1 |
| KMPHG024_19_Rhiniinae | 14 | 10 | 10 | 9 | 9 | 8 | 8 | 8 | 1 | 1 |
| KMPHI411_19_Rhiniinae | 14 | 10 | 10 | 9 | 9 | 8 | 8 | 8 | 1 | 1 |
| KMPHN1967_19_Rhiniinae | 14 | 10 | 10 | 9 | 9 | 8 | 8 | 8 | 1 | 1 |
| KMPHQ1276_19_Rhiniinae | 14 | 10 | 10 | 9 | 9 | 8 | 8 | 8 | 1 | 1 |
| KMPIM021_19_Rhiniinae | 14 | 10 | 10 | 9 | 9 | 8 | 8 | 8 | 1 | 1 |
| KMPIM026_19_Rhiniinae | 14 | 10 | 10 | 9 | 9 | 8 | 8 | 8 | 1 | 1 |
| KMPIO158_19_Rhiniinae | 14 | 10 | 10 | 9 | 9 | 8 | 8 | 8 | 1 | 1 |
| KMPIO174_19_Rhiniinae | 14 | 10 | 10 | 9 | 9 | 8 | 8 | 8 | 1 | 1 |
| KMPIO176_19_Rhiniinae | 14 | 10 | 10 | 9 | 9 | 8 | 8 | 8 | 1 | 1 |
| KMPIO180_19_Rhiniinae | 14 | 10 | 10 | 9 | 9 | 8 | 8 | 8 | 1 | 1 |
| KMPIP254_19_Rhiniinae | 14 | 10 | 10 | 9 | 9 | 8 | 8 | 8 | 1 | 1 |
| KMPIP278_19_Rhiniinae | 14 | 10 | 10 | 9 | 9 | 8 | 8 | 8 | 1 | 1 |
| KMPIQ021_19_Rhiniinae | 14 | 10 | 10 | 9 | 9 | 8 | 8 | 8 | 1 | 1 |
| KMPIS680_19_Rhiniinae | 14 | 10 | 10 | 9 | 9 | 8 | 8 | 8 | 1 | 1 |
| KMPIU620_19_Rhiniinae | 14 | 10 | 10 | 9 | 9 | 8 | 8 | 8 | 1 | 1 |
| KMPJI050_19_Rhiniinae | 14 | 10 | 10 | 9 | 9 | 8 | 8 | 8 | 1 | 1 |
| KMPJI058_19_Rhiniinae | 14 | 10 | 10 | 9 | 9 | 8 | 8 | 8 | 1 | 1 |
| KMPMB382_18_Rhiniinae | 14 | 10 | 10 | 9 | 9 | 8 | 8 | 8 | 1 | 1 |
| KMPMQ358_19_Rhiniinae | 14 | 10 | 10 | 9 | 9 | 8 | 8 | 8 | 1 | 1 |
| KMPMQ360_19_Rhiniinae | 14 | 10 | 10 | 9 | 9 | 8 | 8 | 8 | 1 | 1 |
| KMPNK052_19_Rhiniinae | 14 | 10 | 10 | 9 | 9 | 8 | 8 | 8 | 1 | 1 |
| KMPNL729_19_Rhiniinae | 14 | 10 | 10 | 9 | 9 | 8 | 8 | 8 | 1 | 1 |
| KMPNL732_19_Rhiniinae | 14 | 10 | 10 | 9 | 9 | 8 | 8 | 8 | 1 | 1 |
| KMPNM1244_19_Rhiniinae | 14 | 10 | 10 | 9 | 9 | 8 | 8 | 8 | 1 | 1 |
| KMPNM1255_19_Rhiniinae | 14 | 10 | 10 | 9 | 9 | 8 | 8 | 8 | 1 | 1 |
| KMPOK240_19_Rhiniinae | 14 | 10 | 10 | 9 | 9 | 8 | 8 | 8 | 1 | 1 |
| KMPOL089_19_Rhiniinae | 14 | 10 | 10 | 9 | 9 | 8 | 8 | 8 | 1 | 1 |
| KMPOP045_19_Rhiniinae | 14 | 10 | 10 | 9 | 9 | 8 | 8 | 8 | 1 | 1 |
| KMPRO1178_19_Rhiniinae | 14 | 10 | 10 | 9 | 9 | 8 | 8 | 8 | 1 | 1 |
| KMPUC2832_18_Rhiniinae | 14 | 10 | 10 | 9 | 9 | 8 | 8 | 8 | 1 | 1 |
| KMPXO361_19_Rhiniinae | 14 | 10 | 10 | 9 | 9 | 8 | 8 | 8 | 1 | 1 |
| KMPZC052_19_Rhiniinae | 14 | 10 | 10 | 9 | 9 | 8 | 8 | 8 | 1 | 1 |
| KMTTF027_19_Rhiniinae | 14 | 10 | 10 | 9 | 9 | 8 | 8 | 8 | 1 | 1 |
| KMTTI015_19_Rhiniinae | 14 | 10 | 10 | 9 | 9 | 8 | 8 | 8 | 1 | 1 |
| Y15_Rhyncomya_forcipata_F_SouthAfrica | 14 | 10 | 10 | 9 | 9 | 8 | 8 | 8 | 1 | 1 |
| KMPMQ320_19_Rhiniinae | 14 | 10 | 10 | 9 | 9 | 8 | 8 | 8 | 1 | 1 |
| KMPNM1249_19_Rhiniinae | 14 | 10 | 10 | 9 | 9 | 8 | 8 | 8 | 1 | 1 |
| KMPAH3838_19_Rhiniinae | 14 | 10 | 10 | 9 | 9 | 8 | 8 | 8 | 1 | 1 |
| KMPAH3841_19_Rhiniinae | 14 | 10 | 10 | 9 | 9 | 8 | 8 | 8 | 1 | 1 |
| KMPAH3842_19_Rhiniinae | 14 | 10 | 10 | 9 | 9 | 8 | 8 | 8 | 1 | 1 |
| KMPAJ027_19_Rhiniinae | 14 | 10 | 10 | 9 | 9 | 8 | 8 | 8 | 1 | 1 |
| KMPAL3481_19_Rhiniinae | 14 | 10 | 10 | 9 | 9 | 8 | 8 | 8 | 1 | 1 |
| KMPAL3492_19_Rhiniinae | 14 | 10 | 10 | 9 | 9 | 8 | 8 | 8 | 1 | 1 |
| KMPAL3514_19_Rhiniinae | 14 | 10 | 10 | 9 | 9 | 8 | 8 | 8 | 1 | 1 |
| KMPAL3530_19_Rhiniinae | 14 | 10 | 10 | 9 | 9 | 8 | 8 | 8 | 1 | 1 |
| KMPAL3574_19_Rhiniinae | 14 | 10 | 10 | 9 | 9 | 8 | 8 | 8 | 1 | 1 |
| KMPAL3764_19_Rhiniinae | 14 | 10 | 10 | 9 | 9 | 8 | 8 | 8 | 1 | 1 |
| KMPCP586_19_Rhiniinae | 14 | 10 | 10 | 9 | 9 | 8 | 8 | 8 | 1 | 1 |
| KMPDD074_19_Rhiniinae | 14 | 10 | 10 | 9 | 9 | 8 | 8 | 8 | 1 | 1 |
| KMPDH073_19_Rhiniinae | 14 | 10 | 10 | 9 | 9 | 8 | 8 | 8 | 1 | 1 |
| KMPDJ182_19_Rhiniinae | 14 | 10 | 10 | 9 | 9 | 8 | 8 | 8 | 1 | 1 |
| KMPDJ197_19_Rhiniinae | 14 | 10 | 10 | 9 | 9 | 8 | 8 | 8 | 1 | 1 |
| KMPDJ208_19_Rhiniinae | 14 | 10 | 10 | 9 | 9 | 8 | 8 | 8 | 1 | 1 |
| KMPDJ214_19_Rhiniinae | 14 | 10 | 10 | 9 | 9 | 8 | 8 | 8 | 1 | 1 |
| KMPDJ2238_19_Rhiniinae | 14 | 10 | 10 | 9 | 9 | 8 | 8 | 8 | 1 | 1 |
| KMPEH283_19_Rhiniinae | 14 | 10 | 10 | 9 | 9 | 8 | 8 | 8 | 1 | 1 |
| KMPFR138_19_Rhiniinae | 14 | 10 | 10 | 9 | 9 | 8 | 8 | 8 | 1 | 1 |
| KMPFR141_19_Rhiniinae | 14 | 10 | 10 | 9 | 9 | 8 | 8 | 8 | 1 | 1 |
| KMPFT011_19_Rhiniinae | 14 | 10 | 10 | 9 | 9 | 8 | 8 | 8 | 1 | 1 |
| KMPGM300_19_Rhiniinae | 14 | 10 | 10 | 9 | 9 | 8 | 8 | 8 | 1 | 1 |
| KMPHA131_18_Rhiniinae | 14 | 10 | 10 | 9 | 9 | 8 | 8 | 8 | 1 | 1 |
| KMPHH035_19_Rhiniinae | 14 | 10 | 10 | 9 | 9 | 8 | 8 | 8 | 1 | 1 |
| KMPHL516_19_Rhiniinae | 14 | 10 | 10 | 9 | 9 | 8 | 8 | 8 | 1 | 1 |
| KMPHN1965_19_Rhiniinae | 14 | 10 | 10 | 9 | 9 | 8 | 8 | 8 | 1 | 1 |
| KMPHS142_19_Rhiniinae | 14 | 10 | 10 | 9 | 9 | 8 | 8 | 8 | 1 | 1 |
| KMPHY036_19_Rhiniinae | 14 | 10 | 10 | 9 | 9 | 8 | 8 | 8 | 1 | 1 |
| KMPIA240_18_Rhiniinae | 14 | 10 | 10 | 9 | 9 | 8 | 8 | 8 | 1 | 1 |
| KMPII044_19_Rhiniinae | 14 | 10 | 10 | 9 | 9 | 8 | 8 | 8 | 1 | 1 |
| KMPIL236_19_Rhiniinae | 14 | 10 | 10 | 9 | 9 | 8 | 8 | 8 | 1 | 1 |
| KMPIN006_19_Rhiniinae | 14 | 10 | 10 | 9 | 9 | 8 | 8 | 8 | 1 | 1 |
| KMPIO147_19_Rhiniinae | 14 | 10 | 10 | 9 | 9 | 8 | 8 | 8 | 1 | 1 |
| KMPIO149_19_Rhiniinae | 14 | 10 | 10 | 9 | 9 | 8 | 8 | 8 | 1 | 1 |
| KMPIO150_19_Rhiniinae | 14 | 10 | 10 | 9 | 9 | 8 | 8 | 8 | 1 | 1 |
| KMPIO156_19_Rhiniinae | 14 | 10 | 10 | 9 | 9 | 8 | 8 | 8 | 1 | 1 |
| KMPIO163_19_Rhiniinae | 14 | 10 | 10 | 9 | 9 | 8 | 8 | 8 | 1 | 1 |
| KMPIO164_19_Rhiniinae | 14 | 10 | 10 | 9 | 9 | 8 | 8 | 8 | 1 | 1 |
| KMPIO165_19_Rhiniinae | 14 | 10 | 10 | 9 | 9 | 8 | 8 | 8 | 1 | 1 |
| KMPIO171_19_Rhiniinae | 14 | 10 | 10 | 9 | 9 | 8 | 8 | 8 | 1 | 1 |
| KMPIO182_19_Rhiniinae | 14 | 10 | 10 | 9 | 9 | 8 | 8 | 8 | 1 | 1 |
| KMPIP226_19_Rhiniinae | 14 | 10 | 10 | 9 | 9 | 8 | 8 | 8 | 1 | 1 |
| KMPIP228_19_Rhiniinae | 14 | 10 | 10 | 9 | 9 | 8 | 8 | 8 | 1 | 1 |
| KMPIP232_19_Rhiniinae | 14 | 10 | 10 | 9 | 9 | 8 | 8 | 8 | 1 | 1 |
| KMPIP235_19_Rhiniinae | 14 | 10 | 10 | 9 | 9 | 8 | 8 | 8 | 1 | 1 |
| KMPIP262_19_Rhiniinae | 14 | 10 | 10 | 9 | 9 | 8 | 8 | 8 | 1 | 1 |
| KMPIP290_19_Rhiniinae | 14 | 10 | 10 | 9 | 9 | 8 | 8 | 8 | 1 | 1 |
| KMPIP292_19_Rhiniinae | 14 | 10 | 10 | 9 | 9 | 8 | 8 | 8 | 1 | 1 |
| KMPIQ023_19_Rhiniinae | 14 | 10 | 10 | 9 | 9 | 8 | 8 | 8 | 1 | 1 |
| KMPIQ025_19_Rhiniinae | 14 | 10 | 10 | 9 | 9 | 8 | 8 | 8 | 1 | 1 |
| KMPIS671_19_Rhiniinae | 14 | 10 | 10 | 9 | 9 | 8 | 8 | 8 | 1 | 1 |
| KMPIT136_19_Rhiniinae | 14 | 10 | 10 | 9 | 9 | 8 | 8 | 8 | 1 | 1 |
| KMPIU597_19_Rhiniinae | 14 | 10 | 10 | 9 | 9 | 8 | 8 | 8 | 1 | 1 |
| KMPIV022_19_Rhiniinae | 14 | 10 | 10 | 9 | 9 | 8 | 8 | 8 | 1 | 1 |
| KMPJI067_19_Rhiniinae | 14 | 10 | 10 | 9 | 9 | 8 | 8 | 8 | 1 | 1 |
| KMPJL040_19_Rhiniinae | 14 | 10 | 10 | 9 | 9 | 8 | 8 | 8 | 1 | 1 |
| KMPJM191_19_Rhiniinae | 14 | 10 | 10 | 9 | 9 | 8 | 8 | 8 | 1 | 1 |
| KMPJM194_19_Rhiniinae | 14 | 10 | 10 | 9 | 9 | 8 | 8 | 8 | 1 | 1 |
| KMPJN461_19_Rhiniinae | 14 | 10 | 10 | 9 | 9 | 8 | 8 | 8 | 1 | 1 |
| KMPJT032_19_Rhiniinae | 14 | 10 | 10 | 9 | 9 | 8 | 8 | 8 | 1 | 1 |
| KMPJV094_19_Rhiniinae | 14 | 10 | 10 | 9 | 9 | 8 | 8 | 8 | 1 | 1 |
| KMPKK055_19_Rhiniinae | 14 | 10 | 10 | 9 | 9 | 8 | 8 | 8 | 1 | 1 |
| KMPKK057_19_Rhiniinae | 14 | 10 | 10 | 9 | 9 | 8 | 8 | 8 | 1 | 1 |
| KMPKL080_19_Rhiniinae | 14 | 10 | 10 | 9 | 9 | 8 | 8 | 8 | 1 | 1 |
| KMPKL081_19_Rhiniinae | 14 | 10 | 10 | 9 | 9 | 8 | 8 | 8 | 1 | 1 |
| KMPKM029_19_Rhiniinae | 14 | 10 | 10 | 9 | 9 | 8 | 8 | 8 | 1 | 1 |
| KMPKO189_19_Rhiniinae | 14 | 10 | 10 | 9 | 9 | 8 | 8 | 8 | 1 | 1 |
| KMPMA187_18_Rhiniinae | 14 | 10 | 10 | 9 | 9 | 8 | 8 | 8 | 1 | 1 |
| KMPMA196_18_Rhiniinae | 14 | 10 | 10 | 9 | 9 | 8 | 8 | 8 | 1 | 1 |
| KMPMH070_19_Rhiniinae | 14 | 10 | 10 | 9 | 9 | 8 | 8 | 8 | 1 | 1 |
| KMPMP121_19_Rhiniinae | 14 | 10 | 10 | 9 | 9 | 8 | 8 | 8 | 1 | 1 |
| KMPMQ336_19_Rhiniinae | 14 | 10 | 10 | 9 | 9 | 8 | 8 | 8 | 1 | 1 |
| KMPMQ338_19_Rhiniinae | 14 | 10 | 10 | 9 | 9 | 8 | 8 | 8 | 1 | 1 |
| KMPMQ348_19_Rhiniinae | 14 | 10 | 10 | 9 | 9 | 8 | 8 | 8 | 1 | 1 |
| KMPMQ350_19_Rhiniinae | 14 | 10 | 10 | 9 | 9 | 8 | 8 | 8 | 1 | 1 |
| KMPMQ381_19_Rhiniinae | 14 | 10 | 10 | 9 | 9 | 8 | 8 | 8 | 1 | 1 |
| KMPMQ385_19_Rhiniinae | 14 | 10 | 10 | 9 | 9 | 8 | 8 | 8 | 1 | 1 |
| KMPMQ394_19_Rhiniinae | 14 | 10 | 10 | 9 | 9 | 8 | 8 | 8 | 1 | 1 |
| KMPMQ398_19_Rhiniinae | 14 | 10 | 10 | 9 | 9 | 8 | 8 | 8 | 1 | 1 |
| KMPMQ412_19_Rhiniinae | 14 | 10 | 10 | 9 | 9 | 8 | 8 | 8 | 1 | 1 |
| KMPMQ423_19_Rhiniinae | 14 | 10 | 10 | 9 | 9 | 8 | 8 | 8 | 1 | 1 |
| KMPMQ461_19_Rhiniinae | 14 | 10 | 10 | 9 | 9 | 8 | 8 | 8 | 1 | 1 |
| KMPNA143_18_Rhiniinae | 14 | 10 | 10 | 9 | 9 | 8 | 8 | 8 | 1 | 1 |
| KMPNH1260_19_Rhiniinae | 14 | 10 | 10 | 9 | 9 | 8 | 8 | 8 | 1 | 1 |
| KMPNH1262_19_Rhiniinae | 14 | 10 | 10 | 9 | 9 | 8 | 8 | 8 | 1 | 1 |
| KMPNH1268_19_Rhiniinae | 14 | 10 | 10 | 9 | 9 | 8 | 8 | 8 | 1 | 1 |
| KMPNI1426_19_Rhiniinae | 14 | 10 | 10 | 9 | 9 | 8 | 8 | 8 | 1 | 1 |
| KMPNJ046_19_Rhiniinae | 14 | 10 | 10 | 9 | 9 | 8 | 8 | 8 | 1 | 1 |
| KMPNJ1382_19_Rhiniinae | 14 | 10 | 10 | 9 | 9 | 8 | 8 | 8 | 1 | 1 |
| KMPNK046_19_Rhiniinae | 14 | 10 | 10 | 9 | 9 | 8 | 8 | 8 | 1 | 1 |
| KMPNL715_19_Rhiniinae | 14 | 10 | 10 | 9 | 9 | 8 | 8 | 8 | 1 | 1 |
| KMPNL734_19_Rhiniinae | 14 | 10 | 10 | 9 | 9 | 8 | 8 | 8 | 1 | 1 |
| KMPNM1250_19_Rhiniinae | 14 | 10 | 10 | 9 | 9 | 8 | 8 | 8 | 1 | 1 |
| KMPNM1254_19_Rhiniinae | 14 | 10 | 10 | 9 | 9 | 8 | 8 | 8 | 1 | 1 |
| KMPNP1562_19_Rhiniinae | 14 | 10 | 10 | 9 | 9 | 8 | 8 | 8 | 1 | 1 |
| KMPNP1566_19_Rhiniinae | 14 | 10 | 10 | 9 | 9 | 8 | 8 | 8 | 1 | 1 |
| KMPNQ2515_19_Rhiniinae | 14 | 10 | 10 | 9 | 9 | 8 | 8 | 8 | 1 | 1 |
| KMPNQ2523_19_Rhiniinae | 14 | 10 | 10 | 9 | 9 | 8 | 8 | 8 | 1 | 1 |
| KMPNQ2561_19_Rhiniinae | 14 | 10 | 10 | 9 | 9 | 8 | 8 | 8 | 1 | 1 |
| KMPNR091_19_Rhiniinae | 14 | 10 | 10 | 9 | 9 | 8 | 8 | 8 | 1 | 1 |
| KMPNR097_19_Rhiniinae | 14 | 10 | 10 | 9 | 9 | 8 | 8 | 8 | 1 | 1 |
| KMPOI052_19_Rhiniinae | 14 | 10 | 10 | 9 | 9 | 8 | 8 | 8 | 1 | 1 |
| KMPOR020_19_Rhiniinae | 14 | 10 | 10 | 9 | 9 | 8 | 8 | 8 | 1 | 1 |
| KMPOS024_19_Rhiniinae | 14 | 10 | 10 | 9 | 9 | 8 | 8 | 8 | 1 | 1 |
| KMPOT063_19_Rhiniinae | 14 | 10 | 10 | 9 | 9 | 8 | 8 | 8 | 1 | 1 |
| KMPRI160_19_Rhiniinae | 14 | 10 | 10 | 9 | 9 | 8 | 8 | 8 | 1 | 1 |
| KMPRO1095_19_Rhiniinae | 14 | 10 | 10 | 9 | 9 | 8 | 8 | 8 | 1 | 1 |
| KMPRO1099_19_Rhiniinae | 14 | 10 | 10 | 9 | 9 | 8 | 8 | 8 | 1 | 1 |
| KMPRO1101_19_Rhiniinae | 14 | 10 | 10 | 9 | 9 | 8 | 8 | 8 | 1 | 1 |
| KMPRO1102_19_Rhiniinae | 14 | 10 | 10 | 9 | 9 | 8 | 8 | 8 | 1 | 1 |
| KMPRO1114_19_Rhiniinae | 14 | 10 | 10 | 9 | 9 | 8 | 8 | 8 | 1 | 1 |
| KMPRO1126_19_Rhiniinae | 14 | 10 | 10 | 9 | 9 | 8 | 8 | 8 | 1 | 1 |
| KMPRO1140_19_Rhiniinae | 14 | 10 | 10 | 9 | 9 | 8 | 8 | 8 | 1 | 1 |
| KMPRO1209_19_Rhiniinae | 14 | 10 | 10 | 9 | 9 | 8 | 8 | 8 | 1 | 1 |
| KMPRU005_19_Rhiniinae | 14 | 10 | 10 | 9 | 9 | 8 | 8 | 8 | 1 | 1 |
| KMPRV089_19_Rhiniinae | 14 | 10 | 10 | 9 | 9 | 8 | 8 | 8 | 1 | 1 |
| KMPUD232_19_Rhiniinae | 14 | 10 | 10 | 9 | 9 | 8 | 8 | 8 | 1 | 1 |
| KMPUE627_19_Rhiniinae | 14 | 10 | 10 | 9 | 9 | 8 | 8 | 8 | 1 | 1 |
| KMPUJ016_19_Rhiniinae | 14 | 10 | 10 | 9 | 9 | 8 | 8 | 8 | 1 | 1 |
| KMPUJ486_19_Rhiniinae | 14 | 10 | 10 | 9 | 9 | 8 | 8 | 8 | 1 | 1 |
| KMPZC050_19_Rhiniinae | 14 | 10 | 10 | 9 | 9 | 8 | 8 | 8 | 1 | 1 |
| KMTTB423_18_Rhiniinae | 14 | 10 | 10 | 9 | 9 | 8 | 8 | 8 | 1 | 1 |
| KMTTG130_19_Rhiniinae | 14 | 10 | 10 | 9 | 9 | 8 | 8 | 8 | 1 | 1 |
| KMTTH809_19_Rhiniinae | 14 | 10 | 10 | 9 | 9 | 8 | 8 | 8 | 1 | 1 |
| KMTTH817_19_Rhiniinae | 14 | 10 | 10 | 9 | 9 | 8 | 8 | 8 | 1 | 1 |
| SAFRA3350_18_Rhiniinae | 14 | 10 | 10 | 9 | 9 | 8 | 8 | 8 | 1 | 1 |
| Y28_Rhyncomya_forcipata_F_SouthAfrica | 14 | 10 | 10 | 9 | 9 | 8 | 8 | 8 | 1 | 1 |
| Y29_Rhyncomya_forcipata_M_Namibia | 14 | 10 | 10 | 9 | 9 | 8 | 8 | 8 | 1 | 1 |
| KMPJK162_19_Rhiniinae | 14 | 10 | 10 | 9 | 9 | 8 | 8 | 8 | 1 | 1 |
| KMPMA186_18_Rhiniinae | 14 | 10 | 10 | 9 | 9 | 8 | 8 | 8 | 1 | 1 |
| KMPMF080_19_Rhiniinae | 14 | 10 | 10 | 9 | 9 | 8 | 8 | 8 | 1 | 1 |
| KMPAL3503_19_Rhiniinae | 14 | 10 | 10 | 9 | 9 | 8 | 8 | 8 | 1 | 1 |
| KMPEL052_19_Rhiniinae | 14 | 10 | 10 | 9 | 9 | 8 | 8 | 8 | 1 | 1 |
| KMPFR137_19_Rhiniinae | 14 | 10 | 10 | 9 | 9 | 8 | 8 | 8 | 1 | 1 |
| KMPHJ076_19_Rhiniinae | 14 | 10 | 10 | 9 | 9 | 8 | 8 | 8 | 1 | 1 |
| KMPIO154_19_Rhiniinae | 14 | 10 | 10 | 9 | 9 | 8 | 8 | 8 | 1 | 1 |
| KMPIO167_19_Rhiniinae | 14 | 10 | 10 | 9 | 9 | 8 | 8 | 8 | 1 | 1 |
| KMPIO172_19_Rhiniinae | 14 | 10 | 10 | 9 | 9 | 8 | 8 | 8 | 1 | 1 |
| KMPJL029_19_Rhiniinae | 14 | 10 | 10 | 9 | 9 | 8 | 8 | 8 | 1 | 1 |
| KMPKK062_19_Rhiniinae | 14 | 10 | 10 | 9 | 9 | 8 | 8 | 8 | 1 | 1 |
| KMPKK064_19_Rhiniinae | 14 | 10 | 10 | 9 | 9 | 8 | 8 | 8 | 1 | 1 |
| KMPKN150_19_Rhiniinae | 14 | 10 | 10 | 9 | 9 | 8 | 8 | 8 | 1 | 1 |
| KMPMH072_19_Rhiniinae | 14 | 10 | 10 | 9 | 9 | 8 | 8 | 8 | 1 | 1 |
| KMPMO134_19_Rhiniinae | 14 | 10 | 10 | 9 | 9 | 8 | 8 | 8 | 1 | 1 |
| KMPMQ341_19_Rhiniinae | 14 | 10 | 10 | 9 | 9 | 8 | 8 | 8 | 1 | 1 |
| KMPNH1272_19_Rhiniinae | 14 | 10 | 10 | 9 | 9 | 8 | 8 | 8 | 1 | 1 |
| KMPOX009_19_Rhiniinae | 14 | 10 | 10 | 9 | 9 | 8 | 8 | 8 | 1 | 1 |
| KMPRV076_19_Rhiniinae | 14 | 10 | 10 | 9 | 9 | 8 | 8 | 8 | 1 | 1 |
| KMPVA152_18_Rhiniinae | 14 | 10 | 10 | 9 | 9 | 8 | 8 | 8 | 1 | 1 |
| KMPXO359_19_Rhiniinae | 14 | 10 | 10 | 9 | 9 | 8 | 8 | 8 | 1 | 1 |
| KMPIL300_19_Rhiniinae | 14 | 10 | 10 | 9 | 9 | 8 | 8 | 8 | 1 | 1 |
| KMPIO166_19_Rhiniinae | 14 | 10 | 10 | 9 | 9 | 8 | 8 | 8 | 1 | 1 |
| KMPGQ161_19_Rhiniinae | 14 | 10 | 10 | 9 | 9 | 8 | 8 | 8 | 1 | 1 |
| KMPIP240_19_Rhiniinae | 14 | 10 | 10 | 9 | 9 | 8 | 8 | 8 | 1 | 1 |
| KMPLX033_19_Rhiniinae | 14 | 10 | 10 | 9 | 9 | 8 | 8 | 8 | 1 | 1 |
| KMPMQ332_19_Rhiniinae | 14 | 10 | 10 | 9 | 9 | 8 | 8 | 8 | 1 | 1 |
| KMPRO1130_19_Rhiniinae | 14 | 10 | 10 | 9 | 9 | 8 | 8 | 8 | 1 | 1 |
| KMPRO1131_19_Rhiniinae | 14 | 10 | 10 | 9 | 9 | 8 | 8 | 8 | 1 | 1 |
| Y27_Rhyncomya_forcipata_F_SouthAfrica | 14 | 10 | 10 | 9 | 9 | 8 | 8 | 8 | 1 | 1 |
| KMPFS030_19_Rhiniinae | 14 | 10 | 10 | 9 | 9 | 8 | 8 | 8 | 1 | 1 |
| KMPHP045_19_Rhiniinae | 14 | 10 | 10 | 9 | 9 | 8 | 8 | 8 | 1 | 1 |
| KMPKM035_19_Rhiniinae | 14 | 10 | 10 | 9 | 9 | 8 | 8 | 8 | 1 | 1 |
| KMPMD012_18_Rhiniinae | 14 | 10 | 10 | 9 | 9 | 8 | 8 | 8 | 1 | 1 |
| KMPMO098_19_Rhiniinae | 14 | 10 | 10 | 9 | 9 | 8 | 8 | 8 | 1 | 1 |
| KMPNI331_19_Rhiniinae | 14 | 10 | 10 | 9 | 9 | 8 | 8 | 8 | 1 | 1 |
| KMPUJ129_19_Rhiniinae | 14 | 10 | 10 | 9 | 9 | 8 | 8 | 8 | 1 | 1 |
| KMPWO022_19_Rhiniinae | 14 | 10 | 10 | 9 | 9 | 8 | 8 | 8 | 1 | 1 |
| KMPAH3845_19_Rhiniinae | 14 | 10 | 10 | 9 | 9 | 8 | 8 | 8 | 1 | 1 |
| KMPAL3769_19_Rhiniinae | 14 | 10 | 10 | 9 | 9 | 8 | 8 | 8 | 1 | 1 |
| KMPAL3773_19_Rhiniinae | 14 | 10 | 10 | 9 | 9 | 8 | 8 | 8 | 1 | 1 |
| KMPDG117_19_Rhiniinae | 14 | 10 | 10 | 9 | 9 | 8 | 8 | 8 | 1 | 1 |
| KMPDI213_19_Rhiniinae | 14 | 10 | 10 | 9 | 9 | 8 | 8 | 8 | 1 | 1 |
| KMPDJ186_19_Rhiniinae | 14 | 10 | 10 | 9 | 9 | 8 | 8 | 8 | 1 | 1 |
| KMPHE323_19_Rhiniinae | 14 | 10 | 10 | 9 | 9 | 8 | 8 | 8 | 1 | 1 |
| KMPHM353_19_Rhiniinae | 14 | 10 | 10 | 9 | 9 | 8 | 8 | 8 | 1 | 1 |
| KMPHN1942_19_Rhiniinae | 14 | 10 | 10 | 9 | 9 | 8 | 8 | 8 | 1 | 1 |
| KMPIJ181_19_Rhiniinae | 14 | 10 | 10 | 9 | 9 | 8 | 8 | 8 | 1 | 1 |
| KMPIM024_19_Rhiniinae | 14 | 10 | 10 | 9 | 9 | 8 | 8 | 8 | 1 | 1 |
| KMPIO151_19_Rhiniinae | 14 | 10 | 10 | 9 | 9 | 8 | 8 | 8 | 1 | 1 |
| KMPIO152_19_Rhiniinae | 14 | 10 | 10 | 9 | 9 | 8 | 8 | 8 | 1 | 1 |
| KMPIO157_19_Rhiniinae | 14 | 10 | 10 | 9 | 9 | 8 | 8 | 8 | 1 | 1 |
| KMPIQ020_19_Rhiniinae | 14 | 10 | 10 | 9 | 9 | 8 | 8 | 8 | 1 | 1 |
| KMPIR011_19_Rhiniinae | 14 | 10 | 10 | 9 | 9 | 8 | 8 | 8 | 1 | 1 |
| KMPIT131_19_Rhiniinae | 14 | 10 | 10 | 9 | 9 | 8 | 8 | 8 | 1 | 1 |
| KMPJK155_19_Rhiniinae | 14 | 10 | 10 | 9 | 9 | 8 | 8 | 8 | 1 | 1 |
| KMPJO1838_19_Rhiniinae | 14 | 10 | 10 | 9 | 9 | 8 | 8 | 8 | 1 | 1 |
| KMPJP2434_19_Rhiniinae | 14 | 10 | 10 | 9 | 9 | 8 | 8 | 8 | 1 | 1 |
| KMPJQ046_19_Rhiniinae | 14 | 10 | 10 | 9 | 9 | 8 | 8 | 8 | 1 | 1 |
| KMPKK058_19_Rhiniinae | 14 | 10 | 10 | 9 | 9 | 8 | 8 | 8 | 1 | 1 |
| KMPKL083_19_Rhiniinae | 14 | 10 | 10 | 9 | 9 | 8 | 8 | 8 | 1 | 1 |
| KMPKN137_19_Rhiniinae | 14 | 10 | 10 | 9 | 9 | 8 | 8 | 8 | 1 | 1 |
| KMPMA184_18_Rhiniinae | 14 | 10 | 10 | 9 | 9 | 8 | 8 | 8 | 1 | 1 |
| KMPMC259_18_Rhiniinae | 14 | 10 | 10 | 9 | 9 | 8 | 8 | 8 | 1 | 1 |
| KMPMF081_19_Rhiniinae | 14 | 10 | 10 | 9 | 9 | 8 | 8 | 8 | 1 | 1 |
| KMPML019_19_Rhiniinae | 14 | 10 | 10 | 9 | 9 | 8 | 8 | 8 | 1 | 1 |
| KMPMO095_19_Rhiniinae | 14 | 10 | 10 | 9 | 9 | 8 | 8 | 8 | 1 | 1 |
| KMPMP125_19_Rhiniinae | 14 | 10 | 10 | 9 | 9 | 8 | 8 | 8 | 1 | 1 |
| KMPNH1261_19_Rhiniinae | 14 | 10 | 10 | 9 | 9 | 8 | 8 | 8 | 1 | 1 |
| KMPNM1256_19_Rhiniinae | 14 | 10 | 10 | 9 | 9 | 8 | 8 | 8 | 1 | 1 |
| KMPNR103_19_Rhiniinae | 14 | 10 | 10 | 9 | 9 | 8 | 8 | 8 | 1 | 1 |
| KMPOM226_19_Rhiniinae | 14 | 10 | 10 | 9 | 9 | 8 | 8 | 8 | 1 | 1 |
| KMPRO1169_19_Rhiniinae | 14 | 10 | 10 | 9 | 9 | 8 | 8 | 8 | 1 | 1 |
| KMPUJ014_19_Rhiniinae | 14 | 10 | 10 | 9 | 9 | 8 | 8 | 8 | 1 | 1 |
| KMPLQ1635_19_Rhiniinae | 14 | 10 | 10 | 9 | 9 | 8 | 8 | 8 | 1 | 1 |
| KMPCO426_19_Rhiniinae | 14 | 10 | 10 | 9 | 9 | 8 | 8 | 8 | 1 | 1 |
| KMPWO026_19_Rhiniinae | 14 | 10 | 10 | 9 | 9 | 8 | 8 | 8 | 1 | 1 |
| KMPDJ189_19_Rhiniinae | 14 | 10 | 10 | 9 | 9 | 8 | 8 | 8 | 1 | 1 |
| KMPIO183_19_Rhiniinae | 14 | 10 | 10 | 9 | 9 | 8 | 8 | 8 | 1 | 1 |
| KMPJF263_19_Rhiniinae | 14 | 10 | 10 | 9 | 9 | 8 | 8 | 8 | 1 | 1 |
| KMPMQ422_19_Rhiniinae | 14 | 10 | 10 | 9 | 9 | 8 | 8 | 8 | 1 | 1 |
| KMPNO1398_19_Rhiniinae | 14 | 10 | 10 | 9 | 9 | 8 | 8 | 8 | 1 | 1 |
| KMPNS1504_19_Rhiniinae | 14 | 10 | 10 | 9 | 9 | 8 | 8 | 8 | 1 | 1 |
| KMPWR112_19_Rhiniinae | 14 | 10 | 10 | 9 | 9 | 8 | 8 | 8 | 1 | 1 |
| KMPDI163_19_Rhiniinae | 14 | 10 | 10 | 9 | 9 | 8 | 8 | 8 | 1 | 1 |
| KMPDJ192_19_Rhiniinae | 14 | 10 | 10 | 9 | 9 | 8 | 8 | 8 | 1 | 1 |
| KMPIO160_19_Rhiniinae | 14 | 10 | 10 | 9 | 9 | 8 | 8 | 8 | 1 | 1 |
| KMPHN1943_19_Rhiniinae | 14 | 10 | 10 | 9 | 9 | 8 | 8 | 8 | 1 | 1 |
| KMPIJ182_19_Rhiniinae | 14 | 10 | 10 | 9 | 9 | 8 | 8 | 8 | 1 | 1 |
| KMPNL716_19_Rhiniinae | 14 | 10 | 10 | 9 | 9 | 8 | 8 | 8 | 1 | 1 |
| KMPAL3513_19_Rhiniinae | 14 | 10 | 10 | 9 | 9 | 8 | 8 | 8 | 1 | 1 |
| KMPIO179_19_Rhiniinae | 14 | 10 | 10 | 9 | 9 | 8 | 8 | 8 | 1 | 1 |
| KMPIP296_19_Rhiniinae | 14 | 10 | 10 | 9 | 9 | 8 | 8 | 8 | 1 | 1 |
| KMPJI051_19_Rhiniinae | 14 | 10 | 10 | 9 | 9 | 8 | 8 | 8 | 1 | 1 |
| KMPHS134_19_Rhiniinae | 14 | 10 | 10 | 9 | 9 | 8 | 8 | 8 | 1 | 1 |
| KMPIO181_19_Rhiniinae | 14 | 10 | 10 | 9 | 9 | 8 | 8 | 8 | 1 | 1 |
| KMPIS673_19_Rhiniinae | 14 | 10 | 10 | 9 | 9 | 8 | 8 | 8 | 1 | 1 |
| KMPNB1200_18_Rhiniinae | 14 | 10 | 10 | 9 | 9 | 8 | 8 | 8 | 1 | 1 |
| KMPIO153_19_Rhiniinae | 14 | 10 | 10 | 9 | 9 | 8 | 8 | 8 | 1 | 1 |
| KMPIU594_19_Rhiniinae | 14 | 10 | 10 | 9 | 9 | 8 | 8 | 8 | 1 | 1 |
| KMPSO042_19_Rhiniinae | 14 | 10 | 10 | 9 | 9 | 8 | 8 | 8 | 1 | 1 |
| KMPNI328_19_Rhiniinae | 14 | 10 | 10 | 9 | 9 | 8 | 8 | 8 | 1 | 1 |
| KMPOS025_19_Rhiniinae | 14 | 10 | 10 | 9 | 9 | 8 | 8 | 8 | 1 | 1 |
| KMPPI1145_19_Rhiniinae | 14 | 10 | 10 | 9 | 9 | 8 | 8 | 8 | 1 | 1 |
| KMPRL036_19_Rhiniinae | 14 | 10 | 10 | 9 | 9 | 8 | 8 | 8 | 1 | 1 |
| KMPRO1210_19_Rhiniinae | 14 | 10 | 10 | 9 | 9 | 8 | 8 | 8 | 1 | 1 |
| KMPDE072_19_Rhiniinae | 14 | 10 | 10 | 9 | 9 | 8 | 8 | 8 | 1 | 1 |
| KMPDM072_19_Rhiniinae | 14 | 10 | 10 | 9 | 9 | 8 | 8 | 8 | 1 | 1 |
| KMPFS037_19_Rhiniinae | 14 | 10 | 10 | 9 | 9 | 8 | 8 | 8 | 1 | 1 |
| KMPHR034_19_Rhiniinae | 14 | 10 | 10 | 9 | 9 | 8 | 8 | 8 | 1 | 1 |
| KMPIL239_19_Rhiniinae | 14 | 10 | 10 | 9 | 9 | 8 | 8 | 8 | 1 | 1 |
| KMPIT144_19_Rhiniinae | 14 | 10 | 10 | 9 | 9 | 8 | 8 | 8 | 1 | 1 |
| KMPDI190_19_Rhiniinae | 14 | 10 | 10 | 9 | 9 | 8 | 8 | 8 | 1 | 1 |
| KMPIO159_19_Rhiniinae | 14 | 10 | 10 | 9 | 9 | 8 | 8 | 8 | 1 | 1 |
| KMPIU610_19_Rhiniinae | 14 | 10 | 10 | 9 | 9 | 8 | 8 | 8 | 1 | 1 |
| KMPIU611_19_Rhiniinae | 14 | 10 | 10 | 9 | 9 | 8 | 8 | 8 | 1 | 1 |
| KMPJI064_19_Rhiniinae | 14 | 10 | 10 | 9 | 9 | 8 | 8 | 8 | 1 | 1 |
| KMPKL085_19_Rhiniinae | 14 | 10 | 10 | 9 | 9 | 8 | 8 | 8 | 1 | 1 |
| KMPMQ397_19_Rhiniinae | 14 | 10 | 10 | 9 | 9 | 8 | 8 | 8 | 1 | 1 |
| KMPOX007_19_Rhiniinae | 14 | 10 | 10 | 9 | 9 | 8 | 8 | 8 | 1 | 1 |
| KMPWO028_19_Rhiniinae | 14 | 10 | 10 | 9 | 9 | 8 | 8 | 8 | 1 | 1 |
| KMPSP2607_19_Rhiniinae | 14 | 10 | 10 | 9 | 9 | 8 | 8 | 8 | 1 | 1 |
| KMPIL238_19_Rhiniinae | 14 | 10 | 10 | 9 | 9 | 8 | 8 | 8 | 1 | 1 |
| KMPNB1134_18_Rhiniinae | 14 | 10 | 10 | 9 | 9 | 8 | 8 | 8 | 1 | 1 |
| KMPWR110_19_Rhiniinae | 14 | 10 | 10 | 9 | 9 | 8 | 8 | 8 | 1 | 1 |
| KMPRO1208_19_Rhiniinae | 14 | 10 | 10 | 9 | 9 | 8 | 8 | 8 | 1 | 1 |
| KMPDI220_19_Rhiniinae | 14 | 10 | 10 | 9 | 9 | 8 | 8 | 8 | 1 | 1 |
| KMPHS138_19_Rhiniinae | 14 | 10 | 10 | 9 | 9 | 8 | 8 | 8 | 1 | 1 |
| KMPHS143_19_Rhiniinae | 14 | 10 | 10 | 9 | 9 | 8 | 8 | 8 | 1 | 1 |
| KMPNM1261_19_Rhiniinae | 14 | 10 | 10 | 9 | 9 | 8 | 8 | 8 | 1 | 1 |
| KMPNI325_19_Rhiniinae | 14 | 10 | 10 | 9 | 9 | 8 | 8 | 8 | 1 | 1 |
| KMPRO1192_19_Rhiniinae | 14 | 10 | 10 | 9 | 9 | 8 | 8 | 8 | 1 | 1 |
| KMPDD134_19_Rhiniinae | 14 | 10 | 10 | 9 | 9 | 8 | 8 | 8 | 1 | 1 |
| KMPIO178_19_Rhiniinae | 14 | 10 | 10 | 9 | 9 | 8 | 8 | 8 | 1 | 1 |
| KMPHA111_18_Rhiniinae | 14 | 10 | 10 | 9 | 9 | 8 | 8 | 8 | 1 | 1 |
| KMPJB015_18_Rhiniinae | 14 | 10 | 10 | 9 | 9 | 8 | 8 | 8 | 1 | 1 |
| KMPMA223_18_Rhiniinae | 14 | 10 | 10 | 9 | 9 | 8 | 8 | 8 | 1 | 1 |
| KMPND461_18_Rhiniinae | 14 | 10 | 10 | 9 | 9 | 8 | 8 | 8 | 1 | 1 |
| KMPUH204_19_Rhiniinae | 14 | 10 | 10 | 9 | 9 | 8 | 8 | 8 | 1 | 1 |
| KMPWL304_18_Rhiniinae | 14 | 10 | 10 | 9 | 9 | 8 | 8 | 8 | 1 | 1 |
| KMPDF1896_19_Rhiniinae | 14 | 10 | 10 | 9 | 9 | 8 | 8 | 8 | 1 | 1 |
| KMPJK158_19_Rhiniinae | 14 | 10 | 10 | 9 | 9 | 8 | 8 | 8 | 1 | 1 |
| KMPHO046_19_Rhiniinae | 14 | 10 | 10 | 9 | 9 | 8 | 8 | 8 | 1 | 1 |
| KMPNL738_19_Rhiniinae | 14 | 10 | 10 | 9 | 9 | 8 | 8 | 8 | 1 | 1 |
| KMPRO1142_19_Rhiniinae | 14 | 10 | 10 | 9 | 9 | 8 | 8 | 8 | 1 | 1 |
| KMPIN001_19_Rhiniinae | 14 | 10 | 10 | 9 | 9 | 8 | 8 | 8 | 1 | 1 |
| KMPMQ427_19_Rhiniinae | 14 | 10 | 10 | 9 | 9 | 8 | 8 | 8 | 1 | 1 |
| KMPUJ557_19_Rhiniinae | 14 | 10 | 10 | 9 | 9 | 8 | 8 | 8 | 1 | 1 |
| KMPUI119_19_Rhiniinae | 14 | 10 | 10 | 9 | 9 | 8 | 8 | 8 | 1 | 1 |
| KMPRO1191_19_Rhiniinae | 14 | 10 | 10 | 9 | 9 | 8 | 8 | 8 | 1 | 1 |
| KMPHO043_19_Rhiniinae | 14 | 10 | 10 | 9 | 9 | 8 | 8 | 8 | 1 | 1 |
| KMPOT072_19_Rhiniinae | 14 | 10 | 10 | 9 | 9 | 8 | 8 | 8 | 1 | 1 |
| KMPNK053_19_Rhiniinae | 14 | 10 | 10 | 9 | 9 | 8 | 8 | 8 | 1 | 1 |
| KMPHN1978_19_Rhiniinae | 14 | 10 | 10 | 9 | 9 | 8 | 8 | 8 | 1 | 1 |
| KMPVV002_19_Rhiniinae | 14 | 10 | 10 | 9 | 9 | 8 | 8 | 8 | 1 | 1 |
| KMPWN154_19_Rhiniinae | 14 | 10 | 10 | 9 | 9 | 8 | 8 | 8 | 1 | 1 |
| KMPIP293_19_Rhiniinae | 14 | 10 | 10 | 9 | 9 | 8 | 8 | 8 | 1 | 1 |
| KMPRV083_19_Rhiniinae | 14 | 10 | 10 | 9 | 9 | 8 | 8 | 8 | 1 | 1 |
| KMPHO042_19_Rhiniinae | 14 | 10 | 10 | 9 | 9 | 8 | 8 | 8 | 1 | 1 |
| KMPIP252_19_Rhiniinae | 14 | 10 | 10 | 9 | 9 | 8 | 8 | 8 | 1 | 1 |
| KMPIT142_19_Rhiniinae | 14 | 10 | 10 | 9 | 9 | 8 | 8 | 8 | 1 | 1 |
| KMPEO1242_19_Rhiniinae | 14 | 10 | 10 | 9 | 9 | 8 | 8 | 8 | 1 | 1 |
| KMPIP291_19_Rhiniinae | 14 | 10 | 10 | 9 | 9 | 8 | 8 | 8 | 1 | 1 |
| KMPKN142_19_Rhiniinae | 14 | 10 | 10 | 9 | 9 | 8 | 8 | 8 | 1 | 1 |
| KMPMQ411_19_Rhiniinae | 14 | 10 | 10 | 9 | 9 | 8 | 8 | 8 | 1 | 1 |
| KMPAL3541_19_Rhiniinae | 14 | 10 | 10 | 9 | 9 | 8 | 8 | 8 | 1 | 1 |
| KMPDH075_19_Rhiniinae | 14 | 10 | 10 | 9 | 9 | 8 | 8 | 8 | 1 | 1 |
| KMPIP299_19_Rhiniinae | 14 | 10 | 10 | 9 | 9 | 8 | 8 | 8 | 1 | 1 |
| KMPJP114_19_Rhiniinae | 14 | 10 | 10 | 9 | 9 | 8 | 8 | 8 | 1 | 1 |
| KMPMQ363_19_Rhiniinae | 14 | 10 | 10 | 9 | 9 | 8 | 8 | 8 | 1 | 1 |
| KMPMQ451_19_Rhiniinae | 14 | 10 | 10 | 9 | 9 | 8 | 8 | 8 | 1 | 1 |
| KMPMQ438_19_Rhiniinae | 14 | 10 | 10 | 9 | 9 | 8 | 8 | 8 | 1 | 1 |
| KMPIO177_19_Rhiniinae | 14 | 10 | 10 | 9 | 9 | 8 | 8 | 8 | 1 | 1 |
| KMPKL082_19_Rhiniinae | 14 | 10 | 10 | 9 | 9 | 8 | 8 | 8 | 1 | 1 |
| KMPIP249_19_Rhiniinae | 14 | 10 | 10 | 9 | 9 | 8 | 8 | 8 | 1 | 1 |
| KMPII045_19_Rhiniinae | 14 | 10 | 10 | 9 | 9 | 8 | 8 | 8 | 1 | 1 |
| KMPRO1200_19_Rhiniinae | 14 | 10 | 10 | 9 | 9 | 8 | 8 | 8 | 1 | 1 |
| KMPHN1971_19_Rhiniinae | 14 | 10 | 10 | 9 | 9 | 8 | 8 | 8 | 1 | 1 |
| KMPQ1073_19_Rhiniinae | 14 | 10 | 10 | 9 | 9 | 8 | 8 | 8 | 1 | 1 |
| KMPAL3775_19_Rhiniinae | 14 | 10 | 10 | 9 | 9 | 8 | 8 | 8 | 1 | 1 |
| KMPAL3778_19_Rhiniinae | 14 | 10 | 10 | 9 | 9 | 8 | 8 | 8 | 1 | 1 |
| KMPUC1622_18_Rhiniinae | 14 | 10 | 10 | 9 | 9 | 8 | 8 | 8 | 1 | 1 |
| KMPIO170_19_Rhiniinae | 14 | 10 | 10 | 9 | 9 | 8 | 8 | 8 | 1 | 1 |
| KMPJP2435_19_Rhiniinae | 14 | 10 | 10 | 9 | 9 | 8 | 8 | 8 | 1 | 1 |
| KMPIP287_19_Rhiniinae | 14 | 10 | 10 | 9 | 9 | 8 | 8 | 8 | 1 | 1 |
| KMPNA278_18_Rhiniinae | 14 | 10 | 10 | 9 | 9 | 8 | 8 | 8 | 1 | 1 |
| KMPIP225_19_Rhiniinae | 14 | 10 | 10 | 9 | 9 | 8 | 8 | 8 | 1 | 1 |
| KMPDI414_19_Rhiniinae | 14 | 10 | 10 | 9 | 9 | 8 | 8 | 8 | 1 | 1 |
| KMPHB583_18_Rhiniinae | 14 | 10 | 10 | 9 | 9 | 8 | 8 | 8 | 1 | 1 |
| KMPSO515_19_Rhiniinae | 14 | 10 | 10 | 9 | 9 | 8 | 8 | 8 | 1 | 1 |
| KMPGQ432_19_Rhiniinae | 14 | 10 | 10 | 9 | 9 | 8 | 8 | 8 | 1 | 1 |
| KMPEA132_18_Rhiniinae | 14 | 10 | 10 | 9 | 9 | 8 | 8 | 8 | 1 | 1 |
| KMPOS021_19_Rhiniinae | 14 | 10 | 10 | 9 | 9 | 8 | 8 | 8 | 1 | 1 |
| KMPKN134_19_Rhiniinae | 14 | 10 | 10 | 9 | 9 | 8 | 8 | 8 | 1 | 1 |
| KMPCK025_19_Rhiniinae | 14 | 10 | 10 | 9 | 9 | 8 | 8 | 8 | 1 | 1 |
| KMPJJ692_19_Rhiniinae | 14 | 10 | 10 | 9 | 9 | 8 | 8 | 8 | 1 | 1 |
| KMPUJ478_19_Rhiniinae | 14 | 10 | 10 | 9 | 9 | 8 | 8 | 8 | 1 | 1 |
| KMTTL904_19_Rhiniinae | 14 | 10 | 10 | 9 | 9 | 8 | 8 | 8 | 1 | 1 |
| KMPIQ030_19_Rhiniinae | 14 | 10 | 10 | 9 | 9 | 8 | 8 | 8 | 1 | 1 |
| KMPUD260_19_Rhiniinae | 14 | 10 | 10 | 9 | 9 | 8 | 8 | 8 | 1 | 1 |
| KMPFT012_19_Rhiniinae | 14 | 10 | 10 | 9 | 9 | 8 | 8 | 8 | 1 | 1 |
| KMPIP289_19_Rhiniinae | 14 | 10 | 10 | 9 | 9 | 8 | 8 | 8 | 1 | 1 |
| KMPIP273_19_Rhiniinae | 14 | 10 | 10 | 9 | 9 | 8 | 8 | 8 | 1 | 1 |
| KMPAL3545_19_Rhiniinae | 14 | 10 | 10 | 9 | 9 | 8 | 8 | 8 | 1 | 1 |
| KMPHN1960_19_Rhiniinae | 14 | 10 | 10 | 9 | 9 | 8 | 8 | 8 | 1 | 1 |
| KMPHN1973_19_Rhiniinae | 14 | 10 | 10 | 9 | 9 | 8 | 8 | 8 | 1 | 1 |
| KMPHQ1269_19_Rhiniinae | 14 | 10 | 10 | 9 | 9 | 8 | 8 | 8 | 1 | 1 |
| KMPHN1959_19_Rhiniinae | 14 | 10 | 10 | 9 | 9 | 8 | 8 | 8 | 1 | 1 |
| KMPIN007_19_Rhiniinae | 14 | 10 | 10 | 9 | 9 | 8 | 8 | 8 | 1 | 1 |
| KMPIP251_19_Rhiniinae | 14 | 10 | 10 | 9 | 9 | 8 | 8 | 8 | 1 | 1 |
| KMPJP2438_19_Rhiniinae | 14 | 10 | 10 | 9 | 9 | 8 | 8 | 8 | 1 | 1 |
| KMPNP1568_19_Rhiniinae | 14 | 10 | 10 | 9 | 9 | 8 | 8 | 8 | 1 | 1 |
| KMPIN004_19_Rhiniinae | 14 | 10 | 10 | 9 | 9 | 8 | 8 | 8 | 1 | 1 |
| KMPIP217_19_Rhiniinae | 14 | 10 | 10 | 9 | 9 | 8 | 8 | 8 | 1 | 1 |
| KMPMQ371_19_Rhiniinae | 14 | 10 | 10 | 9 | 9 | 8 | 8 | 8 | 1 | 1 |
| KMPIT138_19_Rhiniinae | 14 | 10 | 10 | 9 | 9 | 8 | 8 | 8 | 1 | 1 |
| KMPMQ418_19_Rhiniinae | 14 | 10 | 10 | 9 | 9 | 8 | 8 | 8 | 1 | 1 |
| KMPHN1974_19_Rhiniinae | 14 | 10 | 10 | 9 | 9 | 8 | 8 | 8 | 1 | 1 |
| KMPMP123_19_Rhiniinae | 14 | 10 | 10 | 9 | 9 | 8 | 8 | 8 | 1 | 1 |
| KMPMQ406_19_Rhiniinae | 14 | 10 | 10 | 9 | 9 | 8 | 8 | 8 | 1 | 1 |
| KMPIK064_19_Rhiniinae | 14 | 10 | 10 | 9 | 9 | 8 | 8 | 8 | 1 | 1 |
| KMPNA288_18_Rhiniinae | 14 | 10 | 10 | 9 | 9 | 8 | 8 | 8 | 1 | 1 |
| KMPIP230_19_Rhiniinae | 14 | 10 | 10 | 9 | 9 | 8 | 8 | 8 | 1 | 1 |
| KMPIP216_19_Rhiniinae | 14 | 10 | 10 | 9 | 9 | 8 | 8 | 8 | 1 | 1 |
| KMPIP236_19_Rhiniinae | 14 | 10 | 10 | 9 | 9 | 8 | 8 | 8 | 1 | 1 |
| KMPIP247_19_Rhiniinae | 14 | 10 | 10 | 9 | 9 | 8 | 8 | 8 | 1 | 1 |
| GMKMW2218_15_Rhiniinae | 14 | 10 | 10 | 9 | 9 | 8 | 8 | 8 | 1 | 1 |
| GMKMA593_15_Rhiniinae | 14 | 10 | 10 | 9 | 9 | 8 | 8 | 8 | 1 | 1 |
| GMKMF022_15_Rhiniinae | 14 | 10 | 10 | 9 | 9 | 8 | 8 | 8 | 1 | 1 |
| GMKMJ917_15_Rhiniinae | 14 | 10 | 10 | 9 | 9 | 8 | 8 | 8 | 1 | 1 |
| GMKMJ926_15_Rhiniinae | 14 | 10 | 10 | 9 | 9 | 8 | 8 | 8 | 1 | 1 |
| GMKMT173_15_Rhiniinae | 14 | 10 | 10 | 9 | 9 | 8 | 8 | 8 | 1 | 1 |
| GMKMU731_15_Rhiniinae | 14 | 10 | 10 | 9 | 9 | 8 | 8 | 8 | 1 | 1 |
| KMPFS032_19_Rhiniinae | 14 | 10 | 10 | 9 | 9 | 8 | 8 | 8 | 1 | 1 |
| KMPHS140_19_Rhiniinae | 14 | 10 | 10 | 9 | 9 | 8 | 8 | 8 | 1 | 1 |
| KMPHX021_19_Rhiniinae | 14 | 10 | 10 | 9 | 9 | 8 | 8 | 8 | 1 | 1 |
| KMPIL240_19_Rhiniinae | 14 | 10 | 10 | 9 | 9 | 8 | 8 | 8 | 1 | 1 |
| KMPIP237_19_Rhiniinae | 14 | 10 | 10 | 9 | 9 | 8 | 8 | 8 | 1 | 1 |
| KMPNS1511_19_Rhiniinae | 14 | 10 | 10 | 9 | 9 | 8 | 8 | 8 | 1 | 1 |
| KMPRO1100_19_Rhiniinae | 14 | 10 | 10 | 9 | 9 | 8 | 8 | 8 | 1 | 1 |
| GMKMN024_15_Rhiniinae | 14 | 10 | 10 | 9 | 9 | 8 | 8 | 8 | 1 | 1 |
| KMPNG022_19_Rhiniinae | 14 | 10 | 10 | 9 | 9 | 8 | 8 | 8 | 1 | 1 |
| KMPOK239_19_Rhiniinae | 14 | 10 | 10 | 9 | 9 | 8 | 8 | 8 | 1 | 1 |
| GMKML237_15_Rhiniinae | 14 | 10 | 10 | 9 | 9 | 8 | 8 | 8 | 1 | 1 |
| GMKMV452_15_Rhiniinae | 14 | 10 | 10 | 9 | 9 | 8 | 8 | 8 | 1 | 1 |
| KMPIQ022_19_Rhiniinae | 14 | 10 | 10 | 9 | 9 | 8 | 8 | 8 | 1 | 1 |
| KMPIP222_19_Rhiniinae | 14 | 10 | 10 | 9 | 9 | 8 | 8 | 8 | 1 | 1 |
| KMPDG163_19_Rhiniinae | 14 | 10 | 10 | 9 | 9 | 8 | 8 | 8 | 1 | 1 |
| KMPIP248_19_Rhiniinae | 14 | 10 | 10 | 9 | 9 | 8 | 8 | 8 | 1 | 1 |
| KMPIP288_19_Rhiniinae | 14 | 10 | 10 | 9 | 9 | 8 | 8 | 8 | 1 | 1 |
| KMPAL1308_19_Rhiniinae | 15 | 11 | 11 | 10 | 9 | 8 | 8 | 8 | 1 | 1 |
| KMPCO427_19_Rhiniinae | 15 | 11 | 11 | 10 | 9 | 8 | 8 | 8 | 1 | 1 |
| KMPCP599_19_Rhiniinae | 15 | 11 | 11 | 10 | 9 | 8 | 8 | 8 | 1 | 1 |
| KMPDA277_19_Rhiniinae | 15 | 11 | 11 | 10 | 9 | 8 | 8 | 8 | 1 | 1 |
| KMPDB257_19_Rhiniinae | 15 | 11 | 11 | 10 | 9 | 8 | 8 | 8 | 1 | 1 |
| KMPDD145_19_Rhiniinae | 15 | 11 | 11 | 10 | 9 | 8 | 8 | 8 | 1 | 1 |
| KMPDI407_19_Rhiniinae | 15 | 11 | 11 | 10 | 9 | 8 | 8 | 8 | 1 | 1 |
| KMPUC2839_18_Rhiniinae | 15 | 11 | 11 | 10 | 9 | 8 | 8 | 8 | 1 | 1 |
| KMPUD4445_19_Rhiniinae | 15 | 11 | 11 | 10 | 9 | 8 | 8 | 8 | 1 | 1 |
| KMPUD4511_19_Rhiniinae | 15 | 11 | 11 | 10 | 9 | 8 | 8 | 8 | 1 | 1 |
| KMPUH247_19_Rhiniinae | 15 | 11 | 11 | 10 | 9 | 8 | 8 | 8 | 1 | 1 |
| KMPUI135_19_Rhiniinae | 15 | 11 | 11 | 10 | 9 | 8 | 8 | 8 | 1 | 1 |
| KMPUI166_19_Rhiniinae | 15 | 11 | 11 | 10 | 9 | 8 | 8 | 8 | 1 | 1 |
| KMPDD117_19_Rhiniinae | 15 | 11 | 11 | 10 | 9 | 8 | 8 | 8 | 1 | 1 |
| KMPUH228_19_Rhiniinae | 15 | 11 | 11 | 10 | 9 | 8 | 8 | 8 | 1 | 1 |
| KMPUA165_18_Rhiniinae | 15 | 11 | 11 | 10 | 9 | 8 | 8 | 8 | 1 | 1 |
| KMPVJ250_19_Rhiniinae | 15 | 11 | 11 | 10 | 9 | 8 | 8 | 8 | 1 | 1 |
| KMPDI168_19_Rhiniinae | 15 | 11 | 11 | 10 | 9 | 8 | 8 | 8 | 1 | 1 |
| KMPUC1609_18_Rhiniinae | 15 | 11 | 11 | 10 | 9 | 8 | 8 | 8 | 1 | 1 |
| KMPUI083_19_Rhiniinae | 15 | 11 | 11 | 10 | 9 | 8 | 8 | 8 | 1 | 1 |
| KMPUI085_19_Rhiniinae | 15 | 11 | 11 | 10 | 9 | 8 | 8 | 8 | 1 | 1 |
| KMPUI178_19_Rhiniinae | 15 | 11 | 11 | 10 | 9 | 8 | 8 | 8 | 1 | 1 |
| Y36_Rhyncomya_sp11_cf_cassotis_M_Togo | 15 | 11 | 11 | 10 | 9 | 8 | 8 | 8 | 1 | 1 |
| Y41_Rhyncomya_sp1_F_Cameroon | 15 | 11 | 11 | 10 | 9 | 8 | 8 | 8 | 1 | 1 |
| Y35_Rhyncomya_cassotis_M_Malawi | 15 | 11 | 11 | 10 | 9 | 8 | 8 | 8 | 1 | 1 |
| Y38_Rhyncomya_cassotis_F_Malawi | 15 | 11 | 11 | 10 | 9 | 8 | 8 | 8 | 1 | 1 |
| SAFRA1138_18_Rhiniinae | 15 | 11 | 11 | 10 | 9 | 8 | 8 | 8 | 1 | 1 |
| Y37_Rhyncomya_sp12_cf_cassotis_M_Zambia | 15 | 11 | 11 | 10 | 9 | 8 | 8 | 8 | 1 | 1 |
| Y39_Rhyncomya_cassotis_F_Namibia | 15 | 11 | 11 | 10 | 9 | 8 | 8 | 8 | 1 | 1 |
| Y56_Rhyncomya_sp19_F_Cameroon | 16 | 12 | 11 | 10 | 9 | 8 | 8 | 8 | 1 | 1 |
| Y40_Rhyncomya_sp3_F_SouthAfrica | 17 | 13 | 12 | 11 | 10 | 9 | 8 | 8 | 1 | 1 |
| Y57_Rhyncomya_sp3_F_SouthAfrica | 17 | 13 | 12 | 11 | 10 | 9 | 8 | 8 | 1 | 1 |
| ASMII1048_22_Stomorhina | 18 | 14 | 13 | 12 | 11 | 10 | 9 | 9 | 1 | 1 |
| ASMII1170_22_Stomorhina | 18 | 14 | 13 | 12 | 11 | 10 | 9 | 9 | 1 | 1 |
| ASMII1171_22_Stomorhina | 18 | 14 | 13 | 12 | 11 | 10 | 9 | 9 | 1 | 1 |
| ASMII1195_22_Stomorhina | 18 | 14 | 13 | 12 | 11 | 10 | 9 | 9 | 1 | 1 |
| ASMII7478_22_Stomorhina | 18 | 14 | 13 | 12 | 11 | 10 | 9 | 9 | 1 | 1 |
| ASMII8543_22_Stomorhina | 18 | 14 | 13 | 12 | 11 | 10 | 9 | 9 | 1 | 1 |
| ASMII8544_22_Stomorhina | 18 | 14 | 13 | 12 | 11 | 10 | 9 | 9 | 1 | 1 |
| DPAS1068_11_Stomorhina | 18 | 14 | 13 | 12 | 11 | 10 | 9 | 9 | 1 | 1 |
| GMAEA5279_22_Stomorhina | 18 | 14 | 13 | 12 | 11 | 10 | 9 | 9 | 1 | 1 |
| HM399341_1_2_650_Stomorhina_pollinosa | 18 | 14 | 13 | 12 | 11 | 10 | 9 | 9 | 1 | 1 |
| ASMII12153_22_Stomorhina_discolor | 19 | 15 | 14 | 13 | 12 | 11 | 10 | 10 | 1 | 1 |
| DIQTB232_11_Stomorhina_discolor | 19 | 15 | 14 | 13 | 12 | 11 | 10 | 10 | 1 | 1 |
| DIQTB580_12_Stomorhina_discolor | 19 | 15 | 14 | 13 | 12 | 11 | 10 | 10 | 1 | 1 |
| HQ561046_1_1_567_Stomorhina_discolor | 19 | 15 | 14 | 13 | 12 | 11 | 10 | 10 | 1 | 1 |
| HQ561056_1_1_650_Stomorhina_discolor | 19 | 15 | 14 | 13 | 12 | 11 | 10 | 10 | 1 | 1 |
| NSWHO3438_18_Stomorhina_discolor | 19 | 15 | 14 | 13 | 12 | 11 | 10 | 10 | 1 | 1 |
| DIQTB231_11_Stomorhina_discolor | 19 | 15 | 14 | 13 | 12 | 11 | 10 | 10 | 1 | 1 |
| KX054615_1_1_615_Rhiniidae_sp | 19 | 15 | 14 | 13 | 12 | 11 | 10 | 10 | 1 | 1 |
| GMBCC1944_15_Stomorhina_discolor | 19 | 15 | 14 | 13 | 12 | 11 | 10 | 10 | 1 | 1 |
| GMBCC3273_15_Stomorhina_discolor | 19 | 15 | 14 | 13 | 12 | 11 | 10 | 10 | 1 | 1 |
| GMBCI160_15_Stomorhina_discolor | 19 | 15 | 14 | 13 | 12 | 11 | 10 | 10 | 1 | 1 |
| GMBCI1616_15_Stomorhina_discolor | 19 | 15 | 14 | 13 | 12 | 11 | 10 | 10 | 1 | 1 |
| GMBCM2533_15_Stomorhina_discolor | 19 | 15 | 14 | 13 | 12 | 11 | 10 | 10 | 1 | 1 |
| GMIBA104_17_Stomorhina_discolor | 19 | 15 | 14 | 13 | 12 | 11 | 10 | 10 | 1 | 1 |
| KY031820_1_1_650_Stomorhina_discolor | 19 | 15 | 14 | 13 | 12 | 11 | 10 | 10 | 1 | 1 |
| KY842007_1_1_588_Stomorhina_discolor | 19 | 15 | 14 | 13 | 12 | 11 | 10 | 10 | 1 | 1 |
| KY844929_1_1_546_Stomorhina_discolor | 19 | 15 | 14 | 13 | 12 | 11 | 10 | 10 | 1 | 1 |
| UKMBB045_13_Stomorhina_discolor | 19 | 15 | 14 | 13 | 12 | 11 | 10 | 10 | 1 | 1 |
| GMBCD1746_15_Stomorhina_discolor | 19 | 15 | 14 | 13 | 12 | 11 | 10 | 10 | 1 | 1 |
| ASMII9570_22_Stomorhina_discolor | 19 | 15 | 14 | 13 | 12 | 11 | 10 | 10 | 1 | 1 |
| GMIAK420_17_Stomorhina_discolor | 19 | 15 | 14 | 13 | 12 | 11 | 10 | 10 | 1 | 1 |
| ASMII5628_22_Stomorhina_discolor | 19 | 15 | 14 | 13 | 12 | 11 | 10 | 10 | 1 | 1 |
| OP268186_1_1_646_Stomorhina_discolor | 19 | 15 | 14 | 13 | 12 | 11 | 10 | 10 | 1 | 1 |
| DIQTB251_11_Stomorhina_discolor | 19 | 15 | 14 | 13 | 12 | 11 | 10 | 10 | 1 | 1 |
| GMBCI2361_15_Stomorhina_discolor | 19 | 15 | 14 | 13 | 12 | 11 | 10 | 10 | 1 | 1 |
| NSWHO3428_18_Stomorhina_discolor | 19 | 15 | 14 | 13 | 12 | 11 | 10 | 10 | 1 | 1 |
| GMBCI2386_15_Stomorhina_discolor | 19 | 15 | 14 | 13 | 12 | 11 | 10 | 10 | 1 | 1 |
| KY031819_1_1_650_Stomorhina_discolor | 19 | 15 | 14 | 13 | 12 | 11 | 10 | 10 | 1 | 1 |
| GMIAK389_17_Stomorhina_discolor | 19 | 15 | 14 | 13 | 12 | 11 | 10 | 10 | 1 | 1 |
| KX054612_1_1_650_Rhiniidae_sp | 19 | 15 | 14 | 13 | 12 | 11 | 10 | 10 | 1 | 1 |
| GMCHB388_14_Stomorhina_discolor | 19 | 15 | 14 | 13 | 12 | 11 | 10 | 10 | 1 | 1 |
| GMCHE034_14_Stomorhina_discolor | 19 | 15 | 14 | 13 | 12 | 11 | 10 | 10 | 1 | 1 |
| KY031821_1_1_650_Stomorhina_obsoleta | 19 | 15 | 14 | 13 | 12 | 11 | 10 | 10 | 1 | 1 |
| LC477291_1_1_650_Stomorhina_obsoleta | 19 | 15 | 14 | 13 | 12 | 11 | 10 | 10 | 1 | 1 |
| LC682331_1_1_650_Stomorhina_obsoleta | 19 | 15 | 14 | 13 | 12 | 11 | 10 | 10 | 1 | 1 |
| OL343412_1_1_650_Stomorhina_obsoleta | 19 | 15 | 14 | 13 | 12 | 11 | 10 | 10 | 1 | 1 |
| OL343410_1_1_650_Stomorhina_obsoleta | 19 | 15 | 14 | 13 | 12 | 11 | 10 | 10 | 1 | 1 |
| OL343411_1_1_650_Stomorhina_obsoleta | 19 | 15 | 14 | 13 | 12 | 11 | 10 | 10 | 1 | 1 |
| GQ409375_1_13_606_Stomorhina_discolor | 19 | 15 | 14 | 13 | 12 | 11 | 10 | 10 | 1 | 1 |
| A5_Cosmina_margaritae_F_Tanzania | 20 | 16 | 15 | 14 | 13 | 12 | 11 | 11 | 1 | 1 |
| A8_Cosmina_gracilis_M_Namibia | 21 | 16 | 15 | 14 | 13 | 12 | 11 | 11 | 1 | 1 |
| KMPDJ929_19_Rhiniinae | 21 | 16 | 15 | 14 | 13 | 12 | 11 | 11 | 1 | 1 |
| KMPUJ618_19_Rhiniinae | 21 | 16 | 15 | 14 | 13 | 12 | 11 | 11 | 1 | 1 |
| KMPUH226_19_Rhiniinae | 21 | 16 | 15 | 14 | 13 | 12 | 11 | 11 | 1 | 1 |
| GMBCC1947_15_Rhiniinae | 22 | 17 | 16 | 15 | 14 | 13 | 12 | 11 | 1 | 1 |
| GMBCD1482_15_Rhiniinae | 22 | 17 | 16 | 15 | 14 | 13 | 12 | 11 | 1 | 1 |
| GMBCD1894_15_Rhiniinae | 22 | 17 | 16 | 15 | 14 | 13 | 12 | 11 | 1 | 1 |
| GMBCE2722_15_Rhiniinae | 22 | 17 | 16 | 15 | 14 | 13 | 12 | 11 | 1 | 1 |
| GMBCE2931_15_Rhiniinae | 22 | 17 | 16 | 15 | 14 | 13 | 12 | 11 | 1 | 1 |
| GMBCF1172_15_Rhiniinae | 22 | 17 | 16 | 15 | 14 | 13 | 12 | 11 | 1 | 1 |
| GMBCF3180_15_Rhiniinae | 22 | 17 | 16 | 15 | 14 | 13 | 12 | 11 | 1 | 1 |
| GMBCN631_15_Rhiniinae | 22 | 17 | 16 | 15 | 14 | 13 | 12 | 11 | 1 | 1 |
| KY835753_1_4_588_Rhiniidae_sp | 22 | 17 | 16 | 15 | 14 | 13 | 12 | 11 | 1 | 1 |
| KY837821_1_15_579_Rhiniidae_sp | 22 | 17 | 16 | 15 | 14 | 13 | 12 | 11 | 1 | 1 |
| KY838746_1_1_573_Rhiniidae_sp | 22 | 17 | 16 | 15 | 14 | 13 | 12 | 11 | 1 | 1 |
| KY838995_1_4_593_Rhiniidae_sp | 22 | 17 | 16 | 15 | 14 | 13 | 12 | 11 | 1 | 1 |
| KY841564_1_1_645_Rhiniidae_sp | 22 | 17 | 16 | 15 | 14 | 13 | 12 | 11 | 1 | 1 |
| GMBCD069_15_Rhiniinae | 22 | 17 | 16 | 15 | 14 | 13 | 12 | 11 | 1 | 1 |
| GMBCD1761_15_Rhiniinae | 22 | 17 | 16 | 15 | 14 | 13 | 12 | 11 | 1 | 1 |
| GMBCE1745_15_Rhiniinae | 22 | 17 | 16 | 15 | 14 | 13 | 12 | 11 | 1 | 1 |
| GMBCE2938_15_Rhiniinae | 22 | 17 | 16 | 15 | 14 | 13 | 12 | 11 | 1 | 1 |
| GMBCE3750_15_Rhiniinae | 22 | 17 | 16 | 15 | 14 | 13 | 12 | 11 | 1 | 1 |
| GMBCF2805_15_Rhiniinae | 22 | 17 | 16 | 15 | 14 | 13 | 12 | 11 | 1 | 1 |
| GMBCN894_15_Rhiniinae | 22 | 17 | 16 | 15 | 14 | 13 | 12 | 11 | 1 | 1 |
| GMPBD013_18_Rhiniinae | 23 | 18 | 17 | 16 | 15 | 14 | 13 | 11 | 1 | 1 |
| GU681898_1_1_650_Rhiniidae_sp | 23 | 18 | 17 | 16 | 15 | 14 | 13 | 11 | 1 | 1 |
| MADIP567_12_Rhiniinae | 23 | 18 | 17 | 16 | 15 | 14 | 13 | 11 | 1 | 1 |
| GMPBK2212_18_Rhiniinae | 23 | 18 | 17 | 16 | 15 | 14 | 13 | 11 | 1 | 1 |
| MADIP1269_13_Rhiniinae | 23 | 18 | 17 | 16 | 15 | 14 | 13 | 11 | 1 | 1 |
| MADIP655_12_Rhiniinae | 23 | 18 | 17 | 16 | 15 | 14 | 13 | 11 | 1 | 1 |
| MADIP048_10_Rhiniinae | 23 | 18 | 17 | 16 | 15 | 14 | 13 | 11 | 1 | 1 |
| MADIP049_10_Rhiniinae | 23 | 18 | 17 | 16 | 15 | 14 | 13 | 11 | 1 | 1 |
| MADIP050_10_Rhiniinae | 23 | 18 | 17 | 16 | 15 | 14 | 13 | 11 | 1 | 1 |
| MADIP051_10_Rhiniinae | 23 | 18 | 17 | 16 | 15 | 14 | 13 | 11 | 1 | 1 |
| MADIP113_10_Rhiniinae | 23 | 18 | 17 | 16 | 15 | 14 | 13 | 11 | 1 | 1 |
| C18_Cosmina_sp5_F_Kenya | 24 | 19 | 18 | 17 | 16 | 15 | 14 | 12 | 1 | 1 |
| GMPBK065_18_Rhiniinae | 25 | 20 | 19 | 18 | 17 | 16 | 15 | 13 | 1 | 1 |
| GMPBK2174_18_Rhiniinae | 25 | 20 | 19 | 18 | 17 | 16 | 15 | 13 | 1 | 1 |
| GMPBK2181_18_Rhiniinae | 25 | 20 | 19 | 18 | 17 | 16 | 15 | 13 | 1 | 1 |
| GMPBK2143_18_Rhiniinae | 25 | 20 | 19 | 18 | 17 | 16 | 15 | 13 | 1 | 1 |
| C19_Cosmina_sp6_M_Kenya | 26 | 21 | 20 | 19 | 18 | 17 | 16 | 14 | 1 | 1 |
| GMBCE3416_15_Rhiniinae | 27 | 22 | 21 | 20 | 19 | 18 | 17 | 15 | 1 | 1 |
| GMBCF5409_15_Rhiniinae | 27 | 22 | 21 | 20 | 19 | 18 | 17 | 15 | 1 | 1 |
| C10_Cosmina_sp3_F_Madagascar | 28 | 23 | 22 | 21 | 20 | 19 | 18 | 16 | 1 | 1 |
| C11_Cosmina_sp2_M_Madagascar | 29 | 24 | 23 | 22 | 21 | 20 | 19 | 17 | 1 | 1 |
| ASMII10886_22_Rhiniinae | 30 | 25 | 24 | 23 | 22 | 21 | 20 | 18 | 1 | 1 |
| ASMII10905_22_Rhiniinae | 30 | 25 | 24 | 23 | 22 | 21 | 20 | 18 | 1 | 1 |
| ASMII2368_22_Rhiniinae | 30 | 25 | 24 | 23 | 22 | 21 | 20 | 18 | 1 | 1 |
| ASMII2465_22_Rhiniinae | 30 | 25 | 24 | 23 | 22 | 21 | 20 | 18 | 1 | 1 |
| ASMII3388_22_Rhiniinae | 30 | 25 | 24 | 23 | 22 | 21 | 20 | 18 | 1 | 1 |
| ASMII7268_22_Rhiniinae | 30 | 25 | 24 | 23 | 22 | 21 | 20 | 18 | 1 | 1 |
| ASMII7289_22_Rhiniinae | 30 | 25 | 24 | 23 | 22 | 21 | 20 | 18 | 1 | 1 |
| AUSMG386_20_Rhiniinae | 30 | 25 | 24 | 23 | 22 | 21 | 20 | 18 | 1 | 1 |
| GMAMH009_15_Rhiniinae | 30 | 25 | 24 | 23 | 22 | 21 | 20 | 18 | 1 | 1 |
| GMAMS685_15_Rhiniinae | 30 | 25 | 24 | 23 | 22 | 21 | 20 | 18 | 1 | 1 |
| GMAMS688_15_Rhiniinae | 30 | 25 | 24 | 23 | 22 | 21 | 20 | 18 | 1 | 1 |
| ASMII2448_22_Rhiniinae | 30 | 25 | 24 | 23 | 22 | 21 | 20 | 18 | 1 | 1 |
| ASMII1114_22_Rhiniinae | 30 | 25 | 24 | 23 | 22 | 21 | 20 | 18 | 1 | 1 |
| ASMII1138_22_Rhiniinae | 30 | 25 | 24 | 23 | 22 | 21 | 20 | 18 | 1 | 1 |
| ASMII7310_22_Rhiniinae | 30 | 25 | 24 | 23 | 22 | 21 | 20 | 18 | 1 | 1 |
| DPAST1393_12_Rhinia | 30 | 25 | 24 | 23 | 22 | 21 | 20 | 18 | 1 | 1 |
| ASMII3436_22_Rhiniinae | 30 | 25 | 24 | 23 | 22 | 21 | 20 | 18 | 1 | 1 |
| ASMII6865_22_Rhiniinae | 30 | 25 | 24 | 23 | 22 | 21 | 20 | 18 | 1 | 1 |
| AUSMG385_20_Rhiniinae | 30 | 25 | 24 | 23 | 22 | 21 | 20 | 18 | 1 | 1 |
| GMAMS750_15_Rhiniinae | 30 | 25 | 24 | 23 | 22 | 21 | 20 | 18 | 1 | 1 |
| GMAMJ027_15_Rhiniinae | 30 | 25 | 24 | 23 | 22 | 21 | 20 | 18 | 1 | 1 |
| GMAML006_15_Rhiniinae | 30 | 25 | 24 | 23 | 22 | 21 | 20 | 18 | 1 | 1 |
| GMAMN526_15_Rhiniinae | 30 | 25 | 24 | 23 | 22 | 21 | 20 | 18 | 1 | 1 |
| GMAMS766_15_Rhiniinae | 30 | 25 | 24 | 23 | 22 | 21 | 20 | 18 | 1 | 1 |
| ASMII2442_22_Rhiniinae | 30 | 25 | 24 | 23 | 22 | 21 | 20 | 18 | 1 | 1 |
| ASMII6818_22_Rhiniinae | 30 | 25 | 24 | 23 | 22 | 21 | 20 | 18 | 1 | 1 |
| GMAMJ186_15_Rhiniinae | 30 | 25 | 24 | 23 | 22 | 21 | 20 | 18 | 1 | 1 |
| ASMII3391_22_Rhiniinae | 30 | 25 | 24 | 23 | 22 | 21 | 20 | 18 | 1 | 1 |
| ASMII3439_22_Rhiniinae | 30 | 25 | 24 | 23 | 22 | 21 | 20 | 18 | 1 | 1 |
| AUSMG383_20_Rhiniinae | 30 | 25 | 24 | 23 | 22 | 21 | 20 | 18 | 1 | 1 |
| ASMII7252_22_Rhiniinae | 30 | 25 | 24 | 23 | 22 | 21 | 20 | 18 | 1 | 1 |
| ASMII7251_22_Rhiniinae | 30 | 25 | 24 | 23 | 22 | 21 | 20 | 18 | 1 | 1 |
| AUSBC1393_12_Rhinia | 30 | 25 | 24 | 23 | 22 | 21 | 20 | 18 | 1 | 1 |
| HM399342_1_1_632_Metallea_incisuralis | 30 | 25 | 24 | 23 | 22 | 21 | 20 | 18 | 1 | 1 |
| HM399346_1_1_650_Metallea_incisuralis | 30 | 25 | 24 | 23 | 22 | 21 | 20 | 18 | 1 | 1 |
| GMAMM349_15_Rhiniinae | 30 | 25 | 24 | 23 | 22 | 21 | 20 | 18 | 1 | 1 |
| ASMII1110_22_Rhiniinae | 30 | 25 | 24 | 23 | 22 | 21 | 20 | 18 | 1 | 1 |
| ASMII1192_22_Rhiniinae | 30 | 25 | 24 | 23 | 22 | 21 | 20 | 18 | 1 | 1 |
| GMCWM164_15_Rhiniinae | 30 | 25 | 24 | 23 | 22 | 21 | 20 | 18 | 1 | 1 |
| GMCWO139_15_Rhiniinae | 30 | 25 | 24 | 23 | 22 | 21 | 20 | 18 | 1 | 1 |
| ASMII1139_22_Rhiniinae | 30 | 25 | 24 | 23 | 22 | 21 | 20 | 18 | 1 | 1 |
| GMCWN700_15_Rhiniinae | 30 | 25 | 24 | 23 | 22 | 21 | 20 | 18 | 1 | 1 |
| GMAMS735_15_Rhiniinae | 30 | 25 | 24 | 23 | 22 | 21 | 20 | 18 | 1 | 1 |
| ASMII1109_22_Rhiniinae | 30 | 25 | 24 | 23 | 22 | 21 | 20 | 18 | 1 | 1 |
| DIQTB444_12_Rhiniinae | 30 | 25 | 24 | 23 | 22 | 21 | 20 | 18 | 1 | 1 |
| HM399345_1_1_650_Metallea_incisuralis | 30 | 25 | 24 | 23 | 22 | 21 | 20 | 18 | 1 | 1 |
| AUSBC1389_12_Rhinia | 30 | 25 | 24 | 23 | 22 | 21 | 20 | 18 | 1 | 1 |
| GMCWK043_15_Rhiniinae | 30 | 25 | 24 | 23 | 22 | 21 | 20 | 18 | 1 | 1 |
| GMCWP067_15_Rhiniinae | 30 | 25 | 24 | 23 | 22 | 21 | 20 | 18 | 1 | 1 |
| ASMII1136_22_Rhiniinae | 30 | 25 | 24 | 23 | 22 | 21 | 20 | 18 | 1 | 1 |
| ASMII1152_22_Rhiniinae | 30 | 25 | 24 | 23 | 22 | 21 | 20 | 18 | 1 | 1 |
| ASMII12149_22_Rhiniinae | 30 | 25 | 24 | 23 | 22 | 21 | 20 | 18 | 1 | 1 |
| GMCWO140_15_Rhiniinae | 30 | 25 | 24 | 23 | 22 | 21 | 20 | 18 | 1 | 1 |
| ASMII4719_22_Rhiniinae | 30 | 25 | 24 | 23 | 22 | 21 | 20 | 18 | 1 | 1 |
| ASMII4749_22_Rhiniinae | 30 | 25 | 24 | 23 | 22 | 21 | 20 | 18 | 1 | 1 |
| GMASF011_17_Rhiniinae | 30 | 25 | 24 | 23 | 22 | 21 | 20 | 18 | 1 | 1 |
| ASMII1352_22_Rhiniinae | 30 | 25 | 24 | 23 | 22 | 21 | 20 | 18 | 1 | 1 |
| ASMII3354_22_Rhiniinae | 30 | 25 | 24 | 23 | 22 | 21 | 20 | 18 | 1 | 1 |
| GMAMI025_15_Rhiniinae | 30 | 25 | 24 | 23 | 22 | 21 | 20 | 18 | 1 | 1 |
| GMAMS689_15_Rhiniinae | 30 | 25 | 24 | 23 | 22 | 21 | 20 | 18 | 1 | 1 |
| GMAMT1498_16_Rhiniinae | 30 | 25 | 24 | 23 | 22 | 21 | 20 | 18 | 1 | 1 |
| ASMII3356_22_Rhiniinae | 30 | 25 | 24 | 23 | 22 | 21 | 20 | 18 | 1 | 1 |
| GMAMJ034_15_Rhiniinae | 30 | 25 | 24 | 23 | 22 | 21 | 20 | 18 | 1 | 1 |
| ASMII3510_22_Rhiniinae | 31 | 25 | 24 | 23 | 22 | 21 | 20 | 18 | 1 | 1 |
| GMAMC010_15_Rhiniinae | 31 | 25 | 24 | 23 | 22 | 21 | 20 | 18 | 1 | 1 |
| GMAML005_15_Rhiniinae | 31 | 25 | 24 | 23 | 22 | 21 | 20 | 18 | 1 | 1 |
| GMAMT1493_16_Rhiniinae | 31 | 25 | 24 | 23 | 22 | 21 | 20 | 18 | 1 | 1 |
| GMAMT1494_16_Rhiniinae | 31 | 25 | 24 | 23 | 22 | 21 | 20 | 18 | 1 | 1 |
| GMAMT1495_16_Rhiniinae | 31 | 25 | 24 | 23 | 22 | 21 | 20 | 18 | 1 | 1 |
| ASMII4720_22_Rhiniinae | 31 | 25 | 24 | 23 | 22 | 21 | 20 | 18 | 1 | 1 |
| ASMII4765_22_Rhiniinae | 31 | 25 | 24 | 23 | 22 | 21 | 20 | 18 | 1 | 1 |
| ASMII4747_22_Rhiniinae | 31 | 25 | 24 | 23 | 22 | 21 | 20 | 18 | 1 | 1 |
| GMBCC1933_15_Rhiniinae | 32 | 26 | 25 | 24 | 23 | 22 | 21 | 18 | 1 | 1 |
| GMBCE2167_15_Rhiniinae | 32 | 26 | 25 | 24 | 23 | 22 | 21 | 18 | 1 | 1 |
| GMPBB006_18_Rhiniinae | 33 | 26 | 25 | 24 | 23 | 22 | 21 | 18 | 1 | 1 |
| GMPBH538_18_Rhiniinae | 33 | 26 | 25 | 24 | 23 | 22 | 21 | 18 | 1 | 1 |
| GMPBK157_18_Rhiniinae | 33 | 26 | 25 | 24 | 23 | 22 | 21 | 18 | 1 | 1 |
| GMPBK2803_18_Rhiniinae | 33 | 26 | 25 | 24 | 23 | 22 | 21 | 18 | 1 | 1 |
| GMPBK161_18_Rhiniinae | 33 | 26 | 25 | 24 | 23 | 22 | 21 | 18 | 1 | 1 |
| GMKME204_15_Rhiniinae | 34 | 27 | 26 | 25 | 24 | 23 | 22 | 19 | 2 | 1 |
| GMKMI691_15_Rhiniinae | 34 | 27 | 26 | 25 | 24 | 23 | 22 | 19 | 2 | 1 |
| GMKMN028_15_Rhiniinae | 34 | 27 | 26 | 25 | 24 | 23 | 22 | 19 | 2 | 1 |
| GMKMU108_15_Rhiniinae | 34 | 27 | 26 | 25 | 24 | 23 | 22 | 19 | 2 | 1 |
| KMPAB4671_18_Rhiniinae | 34 | 27 | 26 | 25 | 24 | 23 | 22 | 19 | 2 | 1 |
| KMPAJ1372_19_Rhiniinae | 34 | 27 | 26 | 25 | 24 | 23 | 22 | 19 | 2 | 1 |
| KMPJR294_19_Rhiniinae | 34 | 27 | 26 | 25 | 24 | 23 | 22 | 19 | 2 | 1 |
| KMPUD349_19_Rhiniinae | 34 | 27 | 26 | 25 | 24 | 23 | 22 | 19 | 2 | 1 |
| KMPHF300_19_Rhiniinae | 34 | 27 | 26 | 25 | 24 | 23 | 22 | 19 | 2 | 1 |
| KMPIM028_19_Rhiniinae | 34 | 27 | 26 | 25 | 24 | 23 | 22 | 19 | 2 | 1 |
| KMPHM360_19_Rhiniinae | 34 | 27 | 26 | 25 | 24 | 23 | 22 | 19 | 2 | 1 |
| KMPAL3523_19_Rhiniinae | 34 | 27 | 26 | 25 | 24 | 23 | 22 | 19 | 2 | 1 |
| KMPJV137_19_Rhiniinae | 34 | 27 | 26 | 25 | 24 | 23 | 22 | 19 | 2 | 1 |
| KMPMQ1717_19_Rhiniinae | 34 | 27 | 26 | 25 | 24 | 23 | 22 | 19 | 2 | 1 |
| KMPMQ388_19_Rhiniinae | 34 | 27 | 26 | 25 | 24 | 23 | 22 | 19 | 2 | 1 |
| KMPNO1136_19_Rhiniinae | 34 | 27 | 26 | 25 | 24 | 23 | 22 | 19 | 2 | 1 |
| KMPWL309_18_Rhiniinae | 34 | 27 | 26 | 25 | 24 | 23 | 22 | 19 | 2 | 1 |
| KMPXS075_19_Rhiniinae | 34 | 27 | 26 | 25 | 24 | 23 | 22 | 19 | 2 | 1 |
| KMPDE961_19_Rhiniinae | 34 | 27 | 26 | 25 | 24 | 23 | 22 | 19 | 2 | 1 |
| KMPIA262_18_Rhiniinae | 34 | 27 | 26 | 25 | 24 | 23 | 22 | 19 | 2 | 1 |
| KMPKL079_19_Rhiniinae | 34 | 27 | 26 | 25 | 24 | 23 | 22 | 19 | 2 | 1 |
| KMPKN147_19_Rhiniinae | 34 | 27 | 26 | 25 | 24 | 23 | 22 | 19 | 2 | 1 |
| KMPMN584_19_Rhiniinae | 34 | 27 | 26 | 25 | 24 | 23 | 22 | 19 | 2 | 1 |
| KMPMO227_19_Rhiniinae | 34 | 27 | 26 | 25 | 24 | 23 | 22 | 19 | 2 | 1 |
| KMPMQ475_19_Rhiniinae | 34 | 27 | 26 | 25 | 24 | 23 | 22 | 19 | 2 | 1 |
| KMPNL724_19_Rhiniinae | 34 | 27 | 26 | 25 | 24 | 23 | 22 | 19 | 2 | 1 |
| KMPUJ831_19_Rhiniinae | 34 | 27 | 26 | 25 | 24 | 23 | 22 | 19 | 2 | 1 |
| KMPWG638_18_Rhiniinae | 34 | 27 | 26 | 25 | 24 | 23 | 22 | 19 | 2 | 1 |
| KMPEC632_18_Rhiniinae | 34 | 27 | 26 | 25 | 24 | 23 | 22 | 19 | 2 | 1 |
| KMPJJ480_19_Rhiniinae | 34 | 27 | 26 | 25 | 24 | 23 | 22 | 19 | 2 | 1 |
| KMPKM033_19_Rhiniinae | 34 | 27 | 26 | 25 | 24 | 23 | 22 | 19 | 2 | 1 |
| KMPCP049_19_Rhiniinae | 34 | 27 | 26 | 25 | 24 | 23 | 22 | 19 | 2 | 1 |
| KMPFI133_19_Rhiniinae | 34 | 27 | 26 | 25 | 24 | 23 | 22 | 19 | 2 | 1 |
| KMPJI052_19_Rhiniinae | 34 | 27 | 26 | 25 | 24 | 23 | 22 | 19 | 2 | 1 |
| KMPUJ046_19_Rhiniinae | 34 | 27 | 26 | 25 | 24 | 23 | 22 | 19 | 2 | 1 |
| KMPXN208_19_Rhiniinae | 34 | 27 | 26 | 25 | 24 | 23 | 22 | 19 | 2 | 1 |
| KMPNQ2565_19_Rhiniinae | 34 | 27 | 26 | 25 | 24 | 23 | 22 | 19 | 2 | 1 |
| KMPWR197_19_Rhiniinae | 34 | 27 | 26 | 25 | 24 | 23 | 22 | 19 | 2 | 1 |
| KMPGA139_18_Rhiniinae | 34 | 27 | 26 | 25 | 24 | 23 | 22 | 19 | 2 | 1 |
| KMPHL515_19_Rhiniinae | 34 | 27 | 26 | 25 | 24 | 23 | 22 | 19 | 2 | 1 |
| KMPMC036_18_Rhiniinae | 34 | 27 | 26 | 25 | 24 | 23 | 22 | 19 | 2 | 1 |
| KMPNM177_19_Rhiniinae | 34 | 27 | 26 | 25 | 24 | 23 | 22 | 19 | 2 | 1 |
| KMPRF129_19_Rhiniinae | 34 | 27 | 26 | 25 | 24 | 23 | 22 | 19 | 2 | 1 |
| KMPHY042_19_Rhiniinae | 34 | 27 | 26 | 25 | 24 | 23 | 22 | 19 | 2 | 1 |
| KMPNJ036_19_Rhiniinae | 34 | 27 | 26 | 25 | 24 | 23 | 22 | 19 | 2 | 1 |
| KMPWT411_19_Rhiniinae | 34 | 27 | 26 | 25 | 24 | 23 | 22 | 19 | 2 | 1 |
| KMPHS149_19_Rhiniinae | 34 | 27 | 26 | 25 | 24 | 23 | 22 | 19 | 2 | 1 |
| KMPKK063_19_Rhiniinae | 34 | 27 | 26 | 25 | 24 | 23 | 22 | 19 | 2 | 1 |
| KMPMQ496_19_Rhiniinae | 34 | 27 | 26 | 25 | 24 | 23 | 22 | 19 | 2 | 1 |
| KMPNM1253_19_Rhiniinae | 34 | 27 | 26 | 25 | 24 | 23 | 22 | 19 | 2 | 1 |
| KMPDG703_19_Rhiniinae | 34 | 27 | 26 | 25 | 24 | 23 | 22 | 19 | 2 | 1 |
| KMPDJ456_19_Rhiniinae | 34 | 27 | 26 | 25 | 24 | 23 | 22 | 19 | 2 | 1 |
| KMPHJ075_19_Rhiniinae | 34 | 27 | 26 | 25 | 24 | 23 | 22 | 19 | 2 | 1 |
| KMPMQ466_19_Rhiniinae | 34 | 27 | 26 | 25 | 24 | 23 | 22 | 19 | 2 | 1 |
| KMPNJ059_19_Rhiniinae | 34 | 27 | 26 | 25 | 24 | 23 | 22 | 19 | 2 | 1 |
| KMPSI076_19_Rhiniinae | 34 | 27 | 26 | 25 | 24 | 23 | 22 | 19 | 2 | 1 |
| KMPUD4446_19_Rhiniinae | 34 | 27 | 26 | 25 | 24 | 23 | 22 | 19 | 2 | 1 |
| KMPDJ1460_19_Rhiniinae | 34 | 27 | 26 | 25 | 24 | 23 | 22 | 19 | 2 | 1 |
| KMPUJ1413_19_Rhiniinae | 34 | 27 | 26 | 25 | 24 | 23 | 22 | 19 | 2 | 1 |
| KMPXQ043_19_Rhiniinae | 34 | 27 | 26 | 25 | 24 | 23 | 22 | 19 | 2 | 1 |
| KMPKL087_19_Rhiniinae | 34 | 27 | 26 | 25 | 24 | 23 | 22 | 19 | 2 | 1 |
| KMPWO1317_19_Rhiniinae | 34 | 27 | 26 | 25 | 24 | 23 | 22 | 19 | 2 | 1 |
| KMPDI162_19_Rhiniinae | 34 | 27 | 26 | 25 | 24 | 23 | 22 | 19 | 2 | 1 |
| KMPXO373_19_Rhiniinae | 34 | 27 | 26 | 25 | 24 | 23 | 22 | 19 | 2 | 1 |
| KMPAL1251_19_Rhiniinae | 34 | 27 | 26 | 25 | 24 | 23 | 22 | 19 | 2 | 1 |
| KMPDJ1000_19_Rhiniinae | 34 | 27 | 26 | 25 | 24 | 23 | 22 | 19 | 2 | 1 |
| KMPFH216_19_Rhiniinae | 34 | 27 | 26 | 25 | 24 | 23 | 22 | 19 | 2 | 1 |
| KMPMO129_19_Rhiniinae | 34 | 27 | 26 | 25 | 24 | 23 | 22 | 19 | 2 | 1 |
| KMPGB135_18_Rhiniinae | 34 | 27 | 26 | 25 | 24 | 23 | 22 | 19 | 2 | 1 |
| KMPLC105_18_Rhiniinae | 34 | 27 | 26 | 25 | 24 | 23 | 22 | 19 | 2 | 1 |
| KMPMQ372_19_Rhiniinae | 34 | 27 | 26 | 25 | 24 | 23 | 22 | 19 | 2 | 1 |
| KMPXO372_19_Rhiniinae | 34 | 27 | 26 | 25 | 24 | 23 | 22 | 19 | 2 | 1 |
| KMPCL053_19_Rhiniinae | 34 | 27 | 26 | 25 | 24 | 23 | 22 | 19 | 2 | 1 |
| KMPHO057_19_Rhiniinae | 34 | 27 | 26 | 25 | 24 | 23 | 22 | 19 | 2 | 1 |
| KMPAL1255_19_Rhiniinae | 34 | 27 | 26 | 25 | 24 | 23 | 22 | 19 | 2 | 1 |
| KMPKI026_19_Rhiniinae | 34 | 27 | 26 | 25 | 24 | 23 | 22 | 19 | 2 | 1 |
| KMPKJ001_19_Rhiniinae | 34 | 27 | 26 | 25 | 24 | 23 | 22 | 19 | 2 | 1 |
| KMPML024_19_Rhiniinae | 34 | 27 | 26 | 25 | 24 | 23 | 22 | 19 | 2 | 1 |
| KMPOG215_19_Rhiniinae | 34 | 27 | 26 | 25 | 24 | 23 | 22 | 19 | 2 | 1 |
| KMPOS078_19_Rhiniinae | 34 | 27 | 26 | 25 | 24 | 23 | 22 | 19 | 2 | 1 |
| KMPSP2639_19_Rhiniinae | 34 | 27 | 26 | 25 | 24 | 23 | 22 | 19 | 2 | 1 |
| KMPXR061_19_Rhiniinae | 34 | 27 | 26 | 25 | 24 | 23 | 22 | 19 | 2 | 1 |
| KMPZB1226_19_Rhiniinae | 34 | 27 | 26 | 25 | 24 | 23 | 22 | 19 | 2 | 1 |
| KMPZE551_19_Rhiniinae | 34 | 27 | 26 | 25 | 24 | 23 | 22 | 19 | 2 | 1 |
| KMPRV464_19_Rhiniinae | 34 | 27 | 26 | 25 | 24 | 23 | 22 | 19 | 2 | 1 |
| KMPST066_19_Rhiniinae | 34 | 27 | 26 | 25 | 24 | 23 | 22 | 19 | 2 | 1 |
| KMPAN1048_19_Rhiniinae | 34 | 27 | 26 | 25 | 24 | 23 | 22 | 19 | 2 | 1 |
| KMPHM050_19_Rhiniinae | 34 | 27 | 26 | 25 | 24 | 23 | 22 | 19 | 2 | 1 |
| KMPIS686_19_Rhiniinae | 34 | 27 | 26 | 25 | 24 | 23 | 22 | 19 | 2 | 1 |
| KMPXP1890_19_Rhiniinae | 34 | 27 | 26 | 25 | 24 | 23 | 22 | 19 | 2 | 1 |
| KMPHG026_19_Rhiniinae | 34 | 27 | 26 | 25 | 24 | 23 | 22 | 19 | 2 | 1 |
| KMPUD2126_19_Rhiniinae | 34 | 27 | 26 | 25 | 24 | 23 | 22 | 19 | 2 | 1 |
| KMPEK756_19_Rhiniinae | 34 | 27 | 26 | 25 | 24 | 23 | 22 | 19 | 2 | 1 |
| KMPWM031_18_Rhiniinae | 34 | 27 | 26 | 25 | 24 | 23 | 22 | 19 | 2 | 1 |
| KMPCP048_19_Rhiniinae | 34 | 27 | 26 | 25 | 24 | 23 | 22 | 19 | 2 | 1 |
| KMPEF308_19_Rhiniinae | 34 | 27 | 26 | 25 | 24 | 23 | 22 | 19 | 2 | 1 |
| KMPNJ049_19_Rhiniinae | 34 | 27 | 26 | 25 | 24 | 23 | 22 | 19 | 2 | 1 |
| KMPUD236_19_Rhiniinae | 34 | 27 | 26 | 25 | 24 | 23 | 22 | 19 | 2 | 1 |
| KMPMO119_19_Rhiniinae | 34 | 27 | 26 | 25 | 24 | 23 | 22 | 19 | 2 | 1 |
| KMPFH092_19_Rhiniinae | 34 | 27 | 26 | 25 | 24 | 23 | 22 | 19 | 2 | 1 |
| KMPNM007_19_Rhiniinae | 34 | 27 | 26 | 25 | 24 | 23 | 22 | 19 | 2 | 1 |
| KMTTC030_18_Rhiniinae | 34 | 27 | 26 | 25 | 24 | 23 | 22 | 19 | 2 | 1 |
| KMPZE553_19_Rhiniinae | 34 | 27 | 26 | 25 | 24 | 23 | 22 | 19 | 2 | 1 |
| KMPWU295_19_Rhiniinae | 34 | 27 | 26 | 25 | 24 | 23 | 22 | 19 | 2 | 1 |
| KMPMQ326_19_Rhiniinae | 34 | 27 | 26 | 25 | 24 | 23 | 22 | 19 | 2 | 1 |
| KMPOL085_19_Rhiniinae | 34 | 27 | 26 | 25 | 24 | 23 | 22 | 19 | 2 | 1 |
| KMPNH1275_19_Rhiniinae | 34 | 27 | 26 | 25 | 24 | 23 | 22 | 19 | 2 | 1 |
| KMPSD032_18_Rhiniinae | 34 | 27 | 26 | 25 | 24 | 23 | 22 | 19 | 2 | 1 |
| KMPSU034_19_Rhiniinae | 34 | 27 | 26 | 25 | 24 | 23 | 22 | 19 | 2 | 1 |
| KMPZE543_19_Rhiniinae | 34 | 27 | 26 | 25 | 24 | 23 | 22 | 19 | 2 | 1 |
| KMPFI125_19_Rhiniinae | 34 | 27 | 26 | 25 | 24 | 23 | 22 | 19 | 2 | 1 |
| KMPJM193_19_Rhiniinae | 34 | 27 | 26 | 25 | 24 | 23 | 22 | 19 | 2 | 1 |
| KMPNR659_19_Rhiniinae | 34 | 27 | 26 | 25 | 24 | 23 | 22 | 19 | 2 | 1 |
| KMPHN1979_19_Rhiniinae | 34 | 27 | 26 | 25 | 24 | 23 | 22 | 19 | 2 | 1 |
| KMPHO047_19_Rhiniinae | 34 | 27 | 26 | 25 | 24 | 23 | 22 | 19 | 2 | 1 |
| KMPIC004_18_Rhiniinae | 34 | 27 | 26 | 25 | 24 | 23 | 22 | 19 | 2 | 1 |
| KMPIC013_18_Rhiniinae | 34 | 27 | 26 | 25 | 24 | 23 | 22 | 19 | 2 | 1 |
| KMPED088_18_Rhiniinae | 34 | 27 | 26 | 25 | 24 | 23 | 22 | 19 | 2 | 1 |
| KMPMC251_18_Rhiniinae | 34 | 27 | 26 | 25 | 24 | 23 | 22 | 19 | 2 | 1 |
| KMPOS030_19_Rhiniinae | 34 | 27 | 26 | 25 | 24 | 23 | 22 | 19 | 2 | 1 |
| KMPNQ2547_19_Rhiniinae | 34 | 27 | 26 | 25 | 24 | 23 | 22 | 19 | 2 | 1 |
| KMPNB1275_18_Rhiniinae | 34 | 27 | 26 | 25 | 24 | 23 | 22 | 19 | 2 | 1 |
| KMPSI193_19_Rhiniinae | 34 | 27 | 26 | 25 | 24 | 23 | 22 | 19 | 2 | 1 |
| KMPIS675_19_Rhiniinae | 34 | 27 | 26 | 25 | 24 | 23 | 22 | 19 | 2 | 1 |
| KMPIS678_19_Rhiniinae | 34 | 27 | 26 | 25 | 24 | 23 | 22 | 19 | 2 | 1 |
| KMPJN525_19_Rhiniinae | 34 | 27 | 26 | 25 | 24 | 23 | 22 | 19 | 2 | 1 |
| KMPWO035_19_Rhiniinae | 34 | 27 | 26 | 25 | 24 | 23 | 22 | 19 | 2 | 1 |
| KMPNJ042_19_Rhiniinae | 34 | 27 | 26 | 25 | 24 | 23 | 22 | 19 | 2 | 1 |
| KMPLC317_18_Rhiniinae | 34 | 27 | 26 | 25 | 24 | 23 | 22 | 19 | 2 | 1 |
| KMPUD1639_19_Rhiniinae | 34 | 27 | 26 | 25 | 24 | 23 | 22 | 19 | 2 | 1 |
| KMPUD261_19_Rhiniinae | 34 | 27 | 26 | 25 | 24 | 23 | 22 | 19 | 2 | 1 |
| KMPXR029_19_Rhiniinae | 34 | 27 | 26 | 25 | 24 | 23 | 22 | 19 | 2 | 1 |
| KMPNO831_19_Rhiniinae | 34 | 27 | 26 | 25 | 24 | 23 | 22 | 19 | 2 | 1 |
| KMPSP775_19_Rhiniinae | 34 | 27 | 26 | 25 | 24 | 23 | 22 | 19 | 2 | 1 |
| KMPUD4516_19_Rhiniinae | 34 | 27 | 26 | 25 | 24 | 23 | 22 | 19 | 2 | 1 |
| KMPHN1951_19_Rhiniinae | 34 | 27 | 26 | 25 | 24 | 23 | 22 | 19 | 2 | 1 |
| KMPIO168_19_Rhiniinae | 34 | 27 | 26 | 25 | 24 | 23 | 22 | 19 | 2 | 1 |
| KMPIV030_19_Rhiniinae | 34 | 27 | 26 | 25 | 24 | 23 | 22 | 19 | 2 | 1 |
| KMPII095_19_Rhiniinae | 34 | 27 | 26 | 25 | 24 | 23 | 22 | 19 | 2 | 1 |
| KMPMD071_18_Rhiniinae | 34 | 27 | 26 | 25 | 24 | 23 | 22 | 19 | 2 | 1 |
| KMPRL034_19_Rhiniinae | 34 | 27 | 26 | 25 | 24 | 23 | 22 | 19 | 2 | 1 |
| KMPCN128_19_Rhiniinae | 34 | 27 | 26 | 25 | 24 | 23 | 22 | 19 | 2 | 1 |
| KMPIP297_19_Rhiniinae | 34 | 27 | 26 | 25 | 24 | 23 | 22 | 19 | 2 | 1 |
| KMPGO035_19_Rhiniinae | 34 | 27 | 26 | 25 | 24 | 23 | 22 | 19 | 2 | 1 |
| KMPAL172_19_Rhiniinae | 34 | 27 | 26 | 25 | 24 | 23 | 22 | 19 | 2 | 1 |
| KMPIK142_19_Rhiniinae | 34 | 27 | 26 | 25 | 24 | 23 | 22 | 19 | 2 | 1 |
| KMPOT218_19_Rhiniinae | 34 | 27 | 26 | 25 | 24 | 23 | 22 | 19 | 2 | 1 |
| KMPUJ3505_19_Rhiniinae | 34 | 27 | 26 | 25 | 24 | 23 | 22 | 19 | 2 | 1 |
| Y59_Rhyncomya_trispina_F_SouthAfrica | 34 | 27 | 26 | 25 | 24 | 23 | 22 | 19 | 2 | 1 |
| KMPDA143_19_Rhiniinae | 34 | 27 | 26 | 25 | 24 | 23 | 22 | 19 | 2 | 1 |
| KMPMQ481_19_Rhiniinae | 34 | 27 | 26 | 25 | 24 | 23 | 22 | 19 | 2 | 1 |
| KMPNO257_19_Rhiniinae | 34 | 27 | 26 | 25 | 24 | 23 | 22 | 19 | 2 | 1 |
| KMPUJ612_19_Rhiniinae | 34 | 27 | 26 | 25 | 24 | 23 | 22 | 19 | 2 | 1 |
| KMPWT2210_19_Rhiniinae | 34 | 27 | 26 | 25 | 24 | 23 | 22 | 19 | 2 | 1 |
| KMPIQ024_19_Rhiniinae | 34 | 27 | 26 | 25 | 24 | 23 | 22 | 19 | 2 | 1 |
| KMPSU033_19_Rhiniinae | 34 | 27 | 26 | 25 | 24 | 23 | 22 | 19 | 2 | 1 |
| KMPIN003_19_Rhiniinae | 34 | 27 | 26 | 25 | 24 | 23 | 22 | 19 | 2 | 1 |
| KMPMA102_18_Rhiniinae | 34 | 27 | 26 | 25 | 24 | 23 | 22 | 19 | 2 | 1 |
| KMPVH036_19_Rhiniinae | 34 | 27 | 26 | 25 | 24 | 23 | 22 | 19 | 2 | 1 |
| KMPNK215_19_Rhiniinae | 34 | 27 | 26 | 25 | 24 | 23 | 22 | 19 | 2 | 1 |
| KMPWO030_19_Rhiniinae | 34 | 27 | 26 | 25 | 24 | 23 | 22 | 19 | 2 | 1 |
| KMPUH069_19_Rhiniinae | 34 | 27 | 26 | 25 | 24 | 23 | 22 | 19 | 2 | 1 |
| KMPDA343_19_Rhiniinae | 34 | 27 | 26 | 25 | 24 | 23 | 22 | 19 | 2 | 1 |
| KMPIO173_19_Rhiniinae | 34 | 27 | 26 | 25 | 24 | 23 | 22 | 19 | 2 | 1 |
| KMPAD2407_19_Rhiniinae | 34 | 27 | 26 | 25 | 24 | 23 | 22 | 19 | 2 | 1 |
| KMPIS684_19_Rhiniinae | 34 | 27 | 26 | 25 | 24 | 23 | 22 | 19 | 2 | 1 |
| KMPOP042_19_Rhiniinae | 34 | 27 | 26 | 25 | 24 | 23 | 22 | 19 | 2 | 1 |
| KMPSO562_19_Rhiniinae | 34 | 27 | 26 | 25 | 24 | 23 | 22 | 19 | 2 | 1 |
| KMPJV096_19_Rhiniinae | 34 | 27 | 26 | 25 | 24 | 23 | 22 | 19 | 2 | 1 |
| KMPKK061_19_Rhiniinae | 34 | 27 | 26 | 25 | 24 | 23 | 22 | 19 | 2 | 1 |
| KMPNQ455_19_Rhiniinae | 34 | 27 | 26 | 25 | 24 | 23 | 22 | 19 | 2 | 1 |
| KMPFB141_18_Rhiniinae | 34 | 27 | 26 | 25 | 24 | 23 | 22 | 19 | 2 | 1 |
| KMPWN152_19_Rhiniinae | 34 | 27 | 26 | 25 | 24 | 23 | 22 | 19 | 2 | 1 |
| KMPOO174_19_Rhiniinae | 34 | 27 | 26 | 25 | 24 | 23 | 22 | 19 | 2 | 1 |
| KMPUI198_19_Rhiniinae | 34 | 27 | 26 | 25 | 24 | 23 | 22 | 19 | 2 | 1 |
| KMPYI012_19_Rhiniinae | 34 | 27 | 26 | 25 | 24 | 23 | 22 | 19 | 2 | 1 |
| KMPAJ193_19_Rhiniinae | 34 | 27 | 26 | 25 | 24 | 23 | 22 | 19 | 2 | 1 |
| KMPMD159_18_Rhiniinae | 34 | 27 | 26 | 25 | 24 | 23 | 22 | 19 | 2 | 1 |
| KMPJL584_19_Rhiniinae | 34 | 27 | 26 | 25 | 24 | 23 | 22 | 19 | 2 | 1 |
| KMPLJ136_19_Rhiniinae | 34 | 27 | 26 | 25 | 24 | 23 | 22 | 19 | 2 | 1 |
| KMPUJ578_19_Rhiniinae | 34 | 27 | 26 | 25 | 24 | 23 | 22 | 19 | 2 | 1 |
| KMPZE549_19_Rhiniinae | 34 | 27 | 26 | 25 | 24 | 23 | 22 | 19 | 2 | 1 |
| KMPNM170_19_Rhiniinae | 34 | 27 | 26 | 25 | 24 | 23 | 22 | 19 | 2 | 1 |
| KMPMA115_18_Rhiniinae | 34 | 27 | 26 | 25 | 24 | 23 | 22 | 19 | 2 | 1 |
| KMPKK066_19_Rhiniinae | 34 | 27 | 26 | 25 | 24 | 23 | 22 | 19 | 2 | 1 |
| KMPWU088_19_Rhiniinae | 34 | 27 | 26 | 25 | 24 | 23 | 22 | 19 | 2 | 1 |
| KMPIT161_19_Rhiniinae | 34 | 27 | 26 | 25 | 24 | 23 | 22 | 19 | 2 | 1 |
| KMPNM112_19_Rhiniinae | 34 | 27 | 26 | 25 | 24 | 23 | 22 | 19 | 2 | 1 |
| KMPEE056_18_Rhiniinae | 34 | 27 | 26 | 25 | 24 | 23 | 22 | 19 | 2 | 1 |
| KMPJK227_19_Rhiniinae | 34 | 27 | 26 | 25 | 24 | 23 | 22 | 19 | 2 | 1 |
| KMPSH308_19_Rhiniinae | 34 | 27 | 26 | 25 | 24 | 23 | 22 | 19 | 2 | 1 |
| KMPRH082_19_Rhiniinae | 34 | 27 | 26 | 25 | 24 | 23 | 22 | 19 | 2 | 1 |
| KMPHC268_18_Rhiniinae | 34 | 27 | 26 | 25 | 24 | 23 | 22 | 19 | 2 | 1 |
| KMPIN005_19_Rhiniinae | 34 | 27 | 26 | 25 | 24 | 23 | 22 | 19 | 2 | 1 |
| KMPDG167_19_Rhiniinae | 34 | 27 | 26 | 25 | 24 | 23 | 22 | 19 | 2 | 1 |
| KMPIS679_19_Rhiniinae | 34 | 27 | 26 | 25 | 24 | 23 | 22 | 19 | 2 | 1 |
| KMPRM325_19_Rhiniinae | 34 | 27 | 26 | 25 | 24 | 23 | 22 | 19 | 2 | 1 |
| KMPFE375_18_Rhiniinae | 34 | 27 | 26 | 25 | 24 | 23 | 22 | 19 | 2 | 1 |
| KMPWL312_18_Rhiniinae | 34 | 27 | 26 | 25 | 24 | 23 | 22 | 19 | 2 | 1 |
| KMPMO135_19_Rhiniinae | 34 | 27 | 26 | 25 | 24 | 23 | 22 | 19 | 2 | 1 |
| KMPIP256_19_Rhiniinae | 34 | 27 | 26 | 25 | 24 | 23 | 22 | 19 | 2 | 1 |
| KMPKP665_19_Rhiniinae | 34 | 27 | 26 | 25 | 24 | 23 | 22 | 19 | 2 | 1 |
| KMPWO1556_19_Rhiniinae | 34 | 27 | 26 | 25 | 24 | 23 | 22 | 19 | 2 | 1 |
| KMPUD2740_19_Rhiniinae | 34 | 27 | 26 | 25 | 24 | 23 | 22 | 19 | 2 | 1 |
| Y58_Rhyncomya_trispina_M_Namibia | 34 | 27 | 26 | 25 | 24 | 23 | 22 | 19 | 2 | 1 |
| KMPWT2208_19_Rhiniinae | 34 | 27 | 26 | 25 | 24 | 23 | 22 | 19 | 2 | 1 |
| KMPMS056_19_Rhiniinae | 34 | 27 | 26 | 25 | 24 | 23 | 22 | 19 | 2 | 1 |
| KMPNK245_19_Rhiniinae | 34 | 27 | 26 | 25 | 24 | 23 | 22 | 19 | 2 | 1 |
| KMPZC060_19_Rhiniinae | 34 | 27 | 26 | 25 | 24 | 23 | 22 | 19 | 2 | 1 |
| KMPKL084_19_Rhiniinae | 34 | 27 | 26 | 25 | 24 | 23 | 22 | 19 | 2 | 1 |
| KMPRM088_19_Rhiniinae | 34 | 27 | 26 | 25 | 24 | 23 | 22 | 19 | 2 | 1 |
| KMPUH274_19_Rhiniinae | 34 | 27 | 26 | 25 | 24 | 23 | 22 | 19 | 2 | 1 |
| KMPDF1861_19_Rhiniinae | 34 | 27 | 26 | 25 | 24 | 23 | 22 | 19 | 2 | 1 |
| KMPHJ078_19_Rhiniinae | 34 | 27 | 26 | 25 | 24 | 23 | 22 | 19 | 2 | 1 |
| KMPUH341_19_Rhiniinae | 34 | 27 | 26 | 25 | 24 | 23 | 22 | 19 | 2 | 1 |
| KMPNQ2577_19_Rhiniinae | 34 | 27 | 26 | 25 | 24 | 23 | 22 | 19 | 2 | 1 |
| KMPJI063_19_Rhiniinae | 34 | 27 | 26 | 25 | 24 | 23 | 22 | 19 | 2 | 1 |
| KMPJK229_19_Rhiniinae | 34 | 27 | 26 | 25 | 24 | 23 | 22 | 19 | 2 | 1 |
| KMPLK071_19_Rhiniinae | 34 | 27 | 26 | 25 | 24 | 23 | 22 | 19 | 2 | 1 |
| KMPWA512_18_Rhiniinae | 34 | 27 | 26 | 25 | 24 | 23 | 22 | 19 | 2 | 1 |
| KMPXN1418_19_Rhiniinae | 34 | 27 | 26 | 25 | 24 | 23 | 22 | 19 | 2 | 1 |
| KMPOX015_19_Rhiniinae | 34 | 27 | 26 | 25 | 24 | 23 | 22 | 19 | 2 | 1 |
| KMPUJ6280_19_Rhiniinae | 34 | 27 | 26 | 25 | 24 | 23 | 22 | 19 | 2 | 1 |
| KMPJV590_19_Rhiniinae | 34 | 27 | 26 | 25 | 24 | 23 | 22 | 19 | 2 | 1 |
| KMPWS172_19_Rhiniinae | 34 | 27 | 26 | 25 | 24 | 23 | 22 | 19 | 2 | 1 |
| KMPOR021_19_Rhiniinae | 34 | 27 | 26 | 25 | 24 | 23 | 22 | 19 | 2 | 1 |
| KMPNA1081_18_Rhiniinae | 34 | 27 | 26 | 25 | 24 | 23 | 22 | 19 | 2 | 1 |
| KMPAJ212_19_Rhiniinae | 34 | 27 | 26 | 25 | 24 | 23 | 22 | 19 | 2 | 1 |
| KMPAL3552_19_Rhiniinae | 34 | 27 | 26 | 25 | 24 | 23 | 22 | 19 | 2 | 1 |
| KMPSP762_19_Rhiniinae | 34 | 27 | 26 | 25 | 24 | 23 | 22 | 19 | 2 | 1 |
| KMPRT093_19_Rhiniinae | 34 | 27 | 26 | 25 | 24 | 23 | 22 | 19 | 2 | 1 |
| KMPCA008_18_Rhiniinae | 34 | 27 | 26 | 25 | 24 | 23 | 22 | 19 | 2 | 1 |
| KMPNK344_19_Rhiniinae | 34 | 27 | 26 | 25 | 24 | 23 | 22 | 19 | 2 | 1 |
| KMPUD4497_19_Rhiniinae | 34 | 27 | 26 | 25 | 24 | 23 | 22 | 19 | 2 | 1 |
| KMPJK103_19_Rhiniinae | 34 | 27 | 26 | 25 | 24 | 23 | 22 | 19 | 2 | 1 |
| KMPUJ1366_19_Rhiniinae | 34 | 27 | 26 | 25 | 24 | 23 | 22 | 19 | 2 | 1 |
| KMPIO186_19_Rhiniinae | 34 | 27 | 26 | 25 | 24 | 23 | 22 | 19 | 2 | 1 |
| KMPVA007_18_Rhiniinae | 34 | 27 | 26 | 25 | 24 | 23 | 22 | 19 | 2 | 1 |
| C20_Cosmina_sp1_cf_fuscipennis_F_SouthAfrica | 35 | 28 | 27 | 26 | 25 | 24 | 23 | 20 | 3 | 2 |
| JQ246691_1_45_694_Cosmina_fuscipennis | 35 | 28 | 27 | 26 | 25 | 24 | 23 | 20 | 3 | 2 |
| USA01_Cosmina_fuscipennis_F_SouthAfrica | 35 | 28 | 27 | 26 | 25 | 24 | 23 | 20 | 3 | 2 |
| USA02_Cosmina_fuscipennis_M_SouthAfrica | 35 | 28 | 27 | 26 | 25 | 24 | 23 | 20 | 3 | 2 |
| A9_Rhyncomya_interclusa_M_SouthAfrica | 36 | 29 | 28 | 27 | 26 | 25 | 24 | 21 | 4 | 3 |
| Y69_Rhyncomya_interclusa_M_SouthAfrica | 36 | 29 | 28 | 27 | 26 | 25 | 24 | 21 | 4 | 3 |
| Y6_Rhyncomya_sp15_F_SouthAfrica | 37 | 30 | 29 | 28 | 27 | 26 | 25 | 22 | 4 | 3 |
| E1_Eurhyncomyia_diversicolor_M_Mozambique | 38 | 31 | 30 | 29 | 28 | 27 | 26 | 23 | 4 | 3 |
| E2_Eurhyncomyia_diversicolor_F_Mozambique | 38 | 31 | 30 | 29 | 28 | 27 | 26 | 23 | 4 | 3 |
| USA12_Rhyncomya_minutalis_F_SouthAfrica | 39 | 32 | 31 | 30 | 29 | 28 | 27 | 24 | 4 | 3 |
| Y13_Rhyncomya_sp16_cf_minutalis_M_SouthAfrica | 39 | 32 | 31 | 30 | 29 | 28 | 27 | 24 | 4 | 3 |
| Y32_Rhyncomya_minutalis_F_SouthAfrica | 39 | 32 | 31 | 30 | 29 | 28 | 27 | 24 | 4 | 3 |
| Y20_Rhyncomya_maculata_M_SouthAfrica | 39 | 32 | 31 | 30 | 29 | 28 | 27 | 24 | 4 | 3 |
| Y67_Rhyncomya_sp7_M_SouthAfrica | 39 | 32 | 31 | 30 | 29 | 28 | 27 | 24 | 4 | 3 |
| KMPED043_18_Rhiniinae | 40 | 33 | 32 | 31 | 30 | 29 | 28 | 25 | 5 | 4 |
| KMPEH241_19_Rhiniinae | 40 | 33 | 32 | 31 | 30 | 29 | 28 | 25 | 5 | 4 |
| KMPIT148_19_Rhiniinae | 40 | 33 | 32 | 31 | 30 | 29 | 28 | 25 | 5 | 4 |
| KMPJT034_19_Rhiniinae | 40 | 33 | 32 | 31 | 30 | 29 | 28 | 25 | 5 | 4 |
| KMPJV556_19_Rhiniinae | 40 | 33 | 32 | 31 | 30 | 29 | 28 | 25 | 5 | 4 |
| KMPNI1440_19_Rhiniinae | 40 | 33 | 32 | 31 | 30 | 29 | 28 | 25 | 5 | 4 |
| KMPGU116_19_Rhiniinae | 40 | 33 | 32 | 31 | 30 | 29 | 28 | 25 | 5 | 4 |
| KMPIT154_19_Rhiniinae | 40 | 33 | 32 | 31 | 30 | 29 | 28 | 25 | 5 | 4 |
| KMPIT156_19_Rhiniinae | 40 | 33 | 32 | 31 | 30 | 29 | 28 | 25 | 5 | 4 |
| KMPIU614_19_Rhiniinae | 40 | 33 | 32 | 31 | 30 | 29 | 28 | 25 | 5 | 4 |
| KMPIV025_19_Rhiniinae | 40 | 33 | 32 | 31 | 30 | 29 | 28 | 25 | 5 | 4 |
| KMPJT030_19_Rhiniinae | 40 | 33 | 32 | 31 | 30 | 29 | 28 | 25 | 5 | 4 |
| KMPJV093_19_Rhiniinae | 40 | 33 | 32 | 31 | 30 | 29 | 28 | 25 | 5 | 4 |
| KMPRL038_19_Rhiniinae | 40 | 33 | 32 | 31 | 30 | 29 | 28 | 25 | 5 | 4 |
| KMPSF186_19_Rhiniinae | 40 | 33 | 32 | 31 | 30 | 29 | 28 | 25 | 5 | 4 |
| KMPSV847_19_Rhiniinae | 40 | 33 | 32 | 31 | 30 | 29 | 28 | 25 | 5 | 4 |
| KMPJU057_19_Rhiniinae | 40 | 33 | 32 | 31 | 30 | 29 | 28 | 25 | 5 | 4 |
| KMPIU626_19_Rhiniinae | 40 | 33 | 32 | 31 | 30 | 29 | 28 | 25 | 5 | 4 |
| KMPIV029_19_Rhiniinae | 40 | 33 | 32 | 31 | 30 | 29 | 28 | 25 | 5 | 4 |
| KMPJV193_19_Rhiniinae | 40 | 33 | 32 | 31 | 30 | 29 | 28 | 25 | 5 | 4 |
| KMPJV296_19_Rhiniinae | 40 | 33 | 32 | 31 | 30 | 29 | 28 | 25 | 5 | 4 |
| KMPIT428_19_Rhiniinae | 40 | 33 | 32 | 31 | 30 | 29 | 28 | 25 | 5 | 4 |
| KMPIU603_19_Rhiniinae | 40 | 33 | 32 | 31 | 30 | 29 | 28 | 25 | 5 | 4 |
| KMPJF267_19_Rhiniinae | 40 | 33 | 32 | 31 | 30 | 29 | 28 | 25 | 5 | 4 |
| KMTTH825_19_Rhiniinae | 40 | 33 | 32 | 31 | 30 | 29 | 28 | 25 | 5 | 4 |
| KMPIU629_19_Rhiniinae | 40 | 33 | 32 | 31 | 30 | 29 | 28 | 25 | 5 | 4 |
| KMPIA255_18_Rhiniinae | 40 | 33 | 32 | 31 | 30 | 29 | 28 | 25 | 5 | 4 |
| KMPIU181_19_Rhiniinae | 40 | 33 | 32 | 31 | 30 | 29 | 28 | 25 | 5 | 4 |
| KMPQJ089_19_Rhiniinae | 40 | 33 | 32 | 31 | 30 | 29 | 28 | 25 | 5 | 4 |
| DIQTB510_12_Rhinia_apicalis | 41 | 34 | 33 | 32 | 31 | 30 | 29 | 26 | 6 | 5 |
| DIQTB559_12_Rhinia_apicalis | 41 | 34 | 33 | 32 | 31 | 30 | 29 | 26 | 6 | 5 |
| DIQTB560_12_Rhinia_apicalis | 41 | 34 | 33 | 32 | 31 | 30 | 29 | 26 | 6 | 5 |
| HM375982_1_1_650_Rhinia_apicalis | 41 | 34 | 33 | 32 | 31 | 30 | 29 | 26 | 6 | 5 |
| MN868846_1_1_650_Rhyncomya_columbina | 42 | 35 | 34 | 33 | 32 | 31 | 30 | 27 | 7 | 6 |
| GMKMA558_15_Rhiniinae | 43 | 36 | 35 | 34 | 33 | 32 | 31 | 28 | 8 | 7 |
| GMKMT188_15_Rhiniinae | 43 | 36 | 35 | 34 | 33 | 32 | 31 | 28 | 8 | 7 |
| Y25_Rhyncomya_pruinosa_F_Kenya | 43 | 36 | 35 | 34 | 33 | 32 | 31 | 28 | 8 | 7 |
| KMPAL3499_19_Rhiniinae | 43 | 36 | 35 | 34 | 33 | 32 | 31 | 28 | 8 | 7 |
| KMPDG151_19_Rhiniinae | 43 | 36 | 35 | 34 | 33 | 32 | 31 | 28 | 8 | 7 |
| KMPDI181_19_Rhiniinae | 43 | 36 | 35 | 34 | 33 | 32 | 31 | 28 | 8 | 7 |
| KMPED013_18_Rhiniinae | 43 | 36 | 35 | 34 | 33 | 32 | 31 | 28 | 8 | 7 |
| KMPEH284_19_Rhiniinae | 43 | 36 | 35 | 34 | 33 | 32 | 31 | 28 | 8 | 7 |
| KMPEH286_19_Rhiniinae | 43 | 36 | 35 | 34 | 33 | 32 | 31 | 28 | 8 | 7 |
| KMPFN007_19_Rhiniinae | 43 | 36 | 35 | 34 | 33 | 32 | 31 | 28 | 8 | 7 |
| KMPHL517_19_Rhiniinae | 43 | 36 | 35 | 34 | 33 | 32 | 31 | 28 | 8 | 7 |
| KMPIL231_19_Rhiniinae | 43 | 36 | 35 | 34 | 33 | 32 | 31 | 28 | 8 | 7 |
| KMPJM189_19_Rhiniinae | 43 | 36 | 35 | 34 | 33 | 32 | 31 | 28 | 8 | 7 |
| KMPKK059_19_Rhiniinae | 43 | 36 | 35 | 34 | 33 | 32 | 31 | 28 | 8 | 7 |
| KMPKK065_19_Rhiniinae | 43 | 36 | 35 | 34 | 33 | 32 | 31 | 28 | 8 | 7 |
| KMPKL086_19_Rhiniinae | 43 | 36 | 35 | 34 | 33 | 32 | 31 | 28 | 8 | 7 |
| KMPKM030_19_Rhiniinae | 43 | 36 | 35 | 34 | 33 | 32 | 31 | 28 | 8 | 7 |
| KMPND457_18_Rhiniinae | 43 | 36 | 35 | 34 | 33 | 32 | 31 | 28 | 8 | 7 |
| KMPNL731_19_Rhiniinae | 43 | 36 | 35 | 34 | 33 | 32 | 31 | 28 | 8 | 7 |
| KMPNM1257_19_Rhiniinae | 43 | 36 | 35 | 34 | 33 | 32 | 31 | 28 | 8 | 7 |
| KMPNP1549_19_Rhiniinae | 43 | 36 | 35 | 34 | 33 | 32 | 31 | 28 | 8 | 7 |
| KMPUB2740_18_Rhiniinae | 43 | 36 | 35 | 34 | 33 | 32 | 31 | 28 | 8 | 7 |
| KMPZB1232_19_Rhiniinae | 43 | 36 | 35 | 34 | 33 | 32 | 31 | 28 | 8 | 7 |
| KMPZB1234_19_Rhiniinae | 43 | 36 | 35 | 34 | 33 | 32 | 31 | 28 | 8 | 7 |
| KMPZB1236_19_Rhiniinae | 43 | 36 | 35 | 34 | 33 | 32 | 31 | 28 | 8 | 7 |
| KMPZB1261_19_Rhiniinae | 43 | 36 | 35 | 34 | 33 | 32 | 31 | 28 | 8 | 7 |
| KMPZB1270_19_Rhiniinae | 43 | 36 | 35 | 34 | 33 | 32 | 31 | 28 | 8 | 7 |
| KMPZB1283_19_Rhiniinae | 43 | 36 | 35 | 34 | 33 | 32 | 31 | 28 | 8 | 7 |
| Y23_Rhyncomya_pruinosa_M_SouthAfrica | 43 | 36 | 35 | 34 | 33 | 32 | 31 | 28 | 8 | 7 |
| Y24_Rhyncomya_pruinosa_M_Malawi | 43 | 36 | 35 | 34 | 33 | 32 | 31 | 28 | 8 | 7 |
| Y26_Rhyncomya_pruinosa_F_SouthAfrica | 43 | 36 | 35 | 34 | 33 | 32 | 31 | 28 | 8 | 7 |
| KMPDF1858_19_Rhiniinae | 43 | 36 | 35 | 34 | 33 | 32 | 31 | 28 | 8 | 7 |
| KMPDH063_19_Rhiniinae | 43 | 36 | 35 | 34 | 33 | 32 | 31 | 28 | 8 | 7 |
| KMPKK056_19_Rhiniinae | 43 | 36 | 35 | 34 | 33 | 32 | 31 | 28 | 8 | 7 |
| KMPMA198_18_Rhiniinae | 43 | 36 | 35 | 34 | 33 | 32 | 31 | 28 | 8 | 7 |
| KMPZB1214_19_Rhiniinae | 43 | 36 | 35 | 34 | 33 | 32 | 31 | 28 | 8 | 7 |
| KMTTL906_19_Rhiniinae | 43 | 36 | 35 | 34 | 33 | 32 | 31 | 28 | 8 | 7 |
| KMPIL234_19_Rhiniinae | 43 | 36 | 35 | 34 | 33 | 32 | 31 | 28 | 8 | 7 |
| KMPZB1264_19_Rhiniinae | 43 | 36 | 35 | 34 | 33 | 32 | 31 | 28 | 8 | 7 |
| KMPEH285_19_Rhiniinae | 43 | 36 | 35 | 34 | 33 | 32 | 31 | 28 | 8 | 7 |
| KMPZB1217_19_Rhiniinae | 43 | 36 | 35 | 34 | 33 | 32 | 31 | 28 | 8 | 7 |
| Y46_Rhyncomya_paratristis_F_SouthAfrica | 44 | 37 | 36 | 35 | 34 | 33 | 32 | 29 | 9 | 8 |
| G8_Stegosoma_sp1_cf_wellmani_F_DRCongo | 45 | 38 | 37 | 36 | 35 | 34 | 33 | 30 | 10 | 9 |
| 1279A07_Stomorhina_chapini_F_Togo | 46 | 39 | 38 | 37 | 36 | 35 | 34 | 31 | 11 | 10 |
| S2_Stomorhina_chapini_F_DRCongo | 46 | 39 | 38 | 37 | 36 | 35 | 34 | 31 | 11 | 10 |
| S3_Stomorhina_chapini_F_SouthAfrica | 47 | 40 | 38 | 37 | 36 | 35 | 34 | 31 | 11 | 10 |
| S4_Stomorhina_chapini_M_SouthAfrica | 47 | 40 | 38 | 37 | 36 | 35 | 34 | 31 | 11 | 10 |
| 1279C01_Stegosoma_wellmani_M_Togo | 48 | 41 | 39 | 38 | 37 | 36 | 35 | 32 | 12 | 11 |
| G1_Stegosoma_bowdeni_M_Togo | 49 | 42 | 40 | 39 | 38 | 37 | 36 | 33 | 13 | 12 |
| G3_Stegosoma_bowdeni_F_Togo | 49 | 42 | 40 | 39 | 38 | 37 | 36 | 33 | 13 | 12 |
| GMKMA584_15_Rhyncomya_soyauxi | 50 | 43 | 41 | 40 | 39 | 38 | 37 | 34 | 14 | 13 |
| GMKMD029_15_Rhyncomya_soyauxi | 50 | 43 | 41 | 40 | 39 | 38 | 37 | 34 | 14 | 13 |
| GMKME194_15_Rhyncomya_soyauxi | 50 | 43 | 41 | 40 | 39 | 38 | 37 | 34 | 14 | 13 |
| GMKMF003_15_Rhyncomya_soyauxi | 50 | 43 | 41 | 40 | 39 | 38 | 37 | 34 | 14 | 13 |
| GMKMJ950_15_Rhyncomya_soyauxi | 50 | 43 | 41 | 40 | 39 | 38 | 37 | 34 | 14 | 13 |
| GMKMS030_15_Rhyncomya_soyauxi | 50 | 43 | 41 | 40 | 39 | 38 | 37 | 34 | 14 | 13 |
| GMKMY231_15_Rhyncomya_soyauxi | 50 | 43 | 41 | 40 | 39 | 38 | 37 | 34 | 14 | 13 |
| USA14_Rhyncomya_soyauxi_M_Kenya | 50 | 43 | 41 | 40 | 39 | 38 | 37 | 34 | 14 | 13 |
| USA15_Rhyncomya_soyauxi_F_Kenya | 50 | 43 | 41 | 40 | 39 | 38 | 37 | 34 | 14 | 13 |
| KMPUD357_19_Rhyncomya_soyauxi | 50 | 43 | 41 | 40 | 39 | 38 | 37 | 34 | 14 | 13 |
| GMKML243_15_Rhyncomya_soyauxi | 50 | 43 | 41 | 40 | 39 | 38 | 37 | 34 | 14 | 13 |
| GMKMD027_15_Rhyncomya_soyauxi | 50 | 43 | 41 | 40 | 39 | 38 | 37 | 34 | 14 | 13 |
| USA13_Rhyncomya_soyauxi_F_Kenya | 50 | 43 | 41 | 40 | 39 | 38 | 37 | 34 | 14 | 13 |
| JQ246693_1_42_691_Rhyncomya_soyauxi | 50 | 43 | 41 | 40 | 39 | 38 | 37 | 34 | 14 | 13 |
| KMPAH001_19_Rhyncomya_soyauxi | 50 | 43 | 41 | 40 | 39 | 38 | 37 | 34 | 14 | 13 |
| KMPAL1228_19_Rhyncomya_soyauxi | 50 | 43 | 41 | 40 | 39 | 38 | 37 | 34 | 14 | 13 |
| KMPAH007_19_Rhyncomya_soyauxi | 50 | 43 | 41 | 40 | 39 | 38 | 37 | 34 | 14 | 13 |
| KMPAH3843_19_Rhyncomya_soyauxi | 50 | 43 | 41 | 40 | 39 | 38 | 37 | 34 | 14 | 13 |
| KMPCP597_19_Rhyncomya_soyauxi | 50 | 43 | 41 | 40 | 39 | 38 | 37 | 34 | 14 | 13 |
| KMPCP600_19_Rhyncomya_soyauxi | 50 | 43 | 41 | 40 | 39 | 38 | 37 | 34 | 14 | 13 |
| KMPDI202_19_Rhyncomya_soyauxi | 50 | 43 | 41 | 40 | 39 | 38 | 37 | 34 | 14 | 13 |
| KMPED012_18_Rhyncomya_soyauxi | 50 | 43 | 41 | 40 | 39 | 38 | 37 | 34 | 14 | 13 |
| KMPEG065_19_Rhyncomya_soyauxi | 50 | 43 | 41 | 40 | 39 | 38 | 37 | 34 | 14 | 13 |
| KMPGM302_19_Rhyncomya_soyauxi | 50 | 43 | 41 | 40 | 39 | 38 | 37 | 34 | 14 | 13 |
| KMPMQ390_19_Rhyncomya_soyauxi | 50 | 43 | 41 | 40 | 39 | 38 | 37 | 34 | 14 | 13 |
| KMPUD266_19_Rhyncomya_soyauxi | 50 | 43 | 41 | 40 | 39 | 38 | 37 | 34 | 14 | 13 |
| KMPUH207_19_Rhyncomya_soyauxi | 50 | 43 | 41 | 40 | 39 | 38 | 37 | 34 | 14 | 13 |
| KMPXH076_19_Rhyncomya_soyauxi | 50 | 43 | 41 | 40 | 39 | 38 | 37 | 34 | 14 | 13 |
| KMPDD073_19_Rhyncomya_soyauxi | 50 | 43 | 41 | 40 | 39 | 38 | 37 | 34 | 14 | 13 |
| KMPAN359_19_Rhyncomya_soyauxi | 50 | 43 | 41 | 40 | 39 | 38 | 37 | 34 | 14 | 13 |
| KMPBB317_18_Rhyncomya_soyauxi | 50 | 43 | 41 | 40 | 39 | 38 | 37 | 34 | 14 | 13 |
| KMPHI410_19_Rhyncomya_soyauxi | 50 | 43 | 41 | 40 | 39 | 38 | 37 | 34 | 14 | 13 |
| KMPJK160_19_Rhyncomya_soyauxi | 50 | 43 | 41 | 40 | 39 | 38 | 37 | 34 | 14 | 13 |
| KMPAH3840_19_Rhyncomya_soyauxi | 50 | 43 | 41 | 40 | 39 | 38 | 37 | 34 | 14 | 13 |
| KMPCP592_19_Rhyncomya_soyauxi | 50 | 43 | 41 | 40 | 39 | 38 | 37 | 34 | 14 | 13 |
| KMPDH061_19_Rhyncomya_soyauxi | 50 | 43 | 41 | 40 | 39 | 38 | 37 | 34 | 14 | 13 |
| KMPJL045_19_Rhyncomya_soyauxi | 50 | 43 | 41 | 40 | 39 | 38 | 37 | 34 | 14 | 13 |
| KMPUJ479_19_Rhyncomya_soyauxi | 50 | 43 | 41 | 40 | 39 | 38 | 37 | 34 | 14 | 13 |
| KMPIO162_19_Rhyncomya_soyauxi | 50 | 43 | 41 | 40 | 39 | 38 | 37 | 34 | 14 | 13 |
| KMPUI163_19_Rhyncomya_soyauxi | 50 | 43 | 41 | 40 | 39 | 38 | 37 | 34 | 14 | 13 |
| KMPUA168_18_Rhyncomya_soyauxi | 50 | 43 | 41 | 40 | 39 | 38 | 37 | 34 | 14 | 13 |
| KMPUE617_19_Rhyncomya_soyauxi | 50 | 43 | 41 | 40 | 39 | 38 | 37 | 34 | 14 | 13 |
| KMPAH035_19_Rhyncomya_soyauxi | 50 | 43 | 41 | 40 | 39 | 38 | 37 | 34 | 14 | 13 |
| KMPAL3472_19_Rhyncomya_soyauxi | 50 | 43 | 41 | 40 | 39 | 38 | 37 | 34 | 14 | 13 |
| KMPAL3474_19_Rhyncomya_soyauxi | 50 | 43 | 41 | 40 | 39 | 38 | 37 | 34 | 14 | 13 |
| KMPAL3515_19_Rhyncomya_soyauxi | 50 | 43 | 41 | 40 | 39 | 38 | 37 | 34 | 14 | 13 |
| KMPAL3561_19_Rhyncomya_soyauxi | 50 | 43 | 41 | 40 | 39 | 38 | 37 | 34 | 14 | 13 |
| KMPAL3568_19_Rhyncomya_soyauxi | 50 | 43 | 41 | 40 | 39 | 38 | 37 | 34 | 14 | 13 |
| KMPAN294_19_Rhyncomya_soyauxi | 50 | 43 | 41 | 40 | 39 | 38 | 37 | 34 | 14 | 13 |
| KMPDE117_19_Rhyncomya_soyauxi | 50 | 43 | 41 | 40 | 39 | 38 | 37 | 34 | 14 | 13 |
| KMPDE167_19_Rhyncomya_soyauxi | 50 | 43 | 41 | 40 | 39 | 38 | 37 | 34 | 14 | 13 |
| KMPDF1899_19_Rhyncomya_soyauxi | 50 | 43 | 41 | 40 | 39 | 38 | 37 | 34 | 14 | 13 |
| KMPDG148_19_Rhyncomya_soyauxi | 50 | 43 | 41 | 40 | 39 | 38 | 37 | 34 | 14 | 13 |
| KMPEG070_19_Rhyncomya_soyauxi | 50 | 43 | 41 | 40 | 39 | 38 | 37 | 34 | 14 | 13 |
| KMPHN1955_19_Rhyncomya_soyauxi | 50 | 43 | 41 | 40 | 39 | 38 | 37 | 34 | 14 | 13 |
| KMPII089_19_Rhyncomya_soyauxi | 50 | 43 | 41 | 40 | 39 | 38 | 37 | 34 | 14 | 13 |
| KMPNP1547_19_Rhyncomya_soyauxi | 50 | 43 | 41 | 40 | 39 | 38 | 37 | 34 | 14 | 13 |
| KMPOE009_18_Rhyncomya_soyauxi | 50 | 43 | 41 | 40 | 39 | 38 | 37 | 34 | 14 | 13 |
| KMPOX008_19_Rhyncomya_soyauxi | 50 | 43 | 41 | 40 | 39 | 38 | 37 | 34 | 14 | 13 |
| KMPUD309_19_Rhyncomya_soyauxi | 50 | 43 | 41 | 40 | 39 | 38 | 37 | 34 | 14 | 13 |
| KMPUD329_19_Rhyncomya_soyauxi | 50 | 43 | 41 | 40 | 39 | 38 | 37 | 34 | 14 | 13 |
| KMPWC021_18_Rhyncomya_soyauxi | 50 | 43 | 41 | 40 | 39 | 38 | 37 | 34 | 14 | 13 |
| KMPWS186_19_Rhyncomya_soyauxi | 50 | 43 | 41 | 40 | 39 | 38 | 37 | 34 | 14 | 13 |
| KMPCP602_19_Rhyncomya_soyauxi | 50 | 43 | 41 | 40 | 39 | 38 | 37 | 34 | 14 | 13 |
| KMPUD123_19_Rhyncomya_soyauxi | 50 | 43 | 41 | 40 | 39 | 38 | 37 | 34 | 14 | 13 |
| KMPCN343_19_Rhyncomya_soyauxi | 50 | 43 | 41 | 40 | 39 | 38 | 37 | 34 | 14 | 13 |
| KMPHJ080_19_Rhyncomya_soyauxi | 50 | 43 | 41 | 40 | 39 | 38 | 37 | 34 | 14 | 13 |
| KMPUB2732_18_Rhyncomya_soyauxi | 50 | 43 | 41 | 40 | 39 | 38 | 37 | 34 | 14 | 13 |
| KMPXQ040_19_Rhyncomya_soyauxi | 50 | 43 | 41 | 40 | 39 | 38 | 37 | 34 | 14 | 13 |
| KMPDC024_19_Rhyncomya_soyauxi | 50 | 43 | 41 | 40 | 39 | 38 | 37 | 34 | 14 | 13 |
| KMPHS132_19_Rhyncomya_soyauxi | 50 | 43 | 41 | 40 | 39 | 38 | 37 | 34 | 14 | 13 |
| KMPNA345_18_Rhyncomya_soyauxi | 50 | 43 | 41 | 40 | 39 | 38 | 37 | 34 | 14 | 13 |
| KMPRK054_19_Rhyncomya_soyauxi | 50 | 43 | 41 | 40 | 39 | 38 | 37 | 34 | 14 | 13 |
| KMPAN305_19_Rhyncomya_soyauxi | 50 | 43 | 41 | 40 | 39 | 38 | 37 | 34 | 14 | 13 |
| KMPAL168_19_Rhyncomya_soyauxi | 50 | 43 | 41 | 40 | 39 | 38 | 37 | 34 | 14 | 13 |
| KMPAL3516_19_Rhyncomya_soyauxi | 50 | 43 | 41 | 40 | 39 | 38 | 37 | 34 | 14 | 13 |
| KMPDD207_19_Rhyncomya_soyauxi | 50 | 43 | 41 | 40 | 39 | 38 | 37 | 34 | 14 | 13 |
| KMPAH036_19_Rhyncomya_soyauxi | 50 | 43 | 41 | 40 | 39 | 38 | 37 | 34 | 14 | 13 |
| KMPAN323_19_Rhyncomya_soyauxi | 50 | 43 | 41 | 40 | 39 | 38 | 37 | 34 | 14 | 13 |
| KMPUD168_19_Rhyncomya_soyauxi | 50 | 43 | 41 | 40 | 39 | 38 | 37 | 34 | 14 | 13 |
| KMPDE103_19_Rhyncomya_soyauxi | 50 | 43 | 41 | 40 | 39 | 38 | 37 | 34 | 14 | 13 |
| KMPUJ131_19_Rhyncomya_soyauxi | 50 | 43 | 41 | 40 | 39 | 38 | 37 | 34 | 14 | 13 |
| KMPUJ477_19_Rhyncomya_soyauxi | 50 | 43 | 41 | 40 | 39 | 38 | 37 | 34 | 14 | 13 |
| KMPGP111_19_Rhyncomya_soyauxi | 50 | 43 | 41 | 40 | 39 | 38 | 37 | 34 | 14 | 13 |
| KMPDB060_19_Rhyncomya_soyauxi | 50 | 43 | 41 | 40 | 39 | 38 | 37 | 34 | 14 | 13 |
| KMPXS074_19_Rhyncomya_soyauxi | 50 | 43 | 41 | 40 | 39 | 38 | 37 | 34 | 14 | 13 |
| KMPAH034_19_Rhyncomya_soyauxi | 50 | 43 | 41 | 40 | 39 | 38 | 37 | 34 | 14 | 13 |
| KMPDC038_19_Rhyncomya_soyauxi | 50 | 43 | 41 | 40 | 39 | 38 | 37 | 34 | 14 | 13 |
| KMPED003_18_Rhyncomya_soyauxi | 50 | 43 | 41 | 40 | 39 | 38 | 37 | 34 | 14 | 13 |
| KMPMQ347_19_Rhyncomya_soyauxi | 50 | 43 | 41 | 40 | 39 | 38 | 37 | 34 | 14 | 13 |
| KMPNB1132_18_Rhyncomya_soyauxi | 50 | 43 | 41 | 40 | 39 | 38 | 37 | 34 | 14 | 13 |
| KMPUJ101_19_Rhyncomya_soyauxi | 50 | 43 | 41 | 40 | 39 | 38 | 37 | 34 | 14 | 13 |
| KMPWB072_18_Rhyncomya_soyauxi | 50 | 43 | 41 | 40 | 39 | 38 | 37 | 34 | 14 | 13 |
| KMPUD240_19_Rhyncomya_soyauxi | 50 | 43 | 41 | 40 | 39 | 38 | 37 | 34 | 14 | 13 |
| KMPUJ490_19_Rhyncomya_soyauxi | 50 | 43 | 41 | 40 | 39 | 38 | 37 | 34 | 14 | 13 |
| KMPWT2205_19_Rhyncomya_soyauxi | 50 | 43 | 41 | 40 | 39 | 38 | 37 | 34 | 14 | 13 |
| KMPAN295_19_Rhyncomya_soyauxi | 50 | 43 | 41 | 40 | 39 | 38 | 37 | 34 | 14 | 13 |
| KMPUD211_19_Rhyncomya_soyauxi | 50 | 43 | 41 | 40 | 39 | 38 | 37 | 34 | 14 | 13 |
| KMPXP1899_19_Rhyncomya_soyauxi | 50 | 43 | 41 | 40 | 39 | 38 | 37 | 34 | 14 | 13 |
| KMPAL3498_19_Rhyncomya_soyauxi | 50 | 43 | 41 | 40 | 39 | 38 | 37 | 34 | 14 | 13 |
| KMPUD281_19_Rhyncomya_soyauxi | 50 | 43 | 41 | 40 | 39 | 38 | 37 | 34 | 14 | 13 |
| KMPUD130_19_Rhyncomya_soyauxi | 50 | 43 | 41 | 40 | 39 | 38 | 37 | 34 | 14 | 13 |
| KMPUD148_19_Rhyncomya_soyauxi | 50 | 43 | 41 | 40 | 39 | 38 | 37 | 34 | 14 | 13 |
| KMPAL3533_19_Rhyncomya_soyauxi | 50 | 43 | 41 | 40 | 39 | 38 | 37 | 34 | 14 | 13 |
| KMPUD107_19_Rhyncomya_soyauxi | 50 | 43 | 41 | 40 | 39 | 38 | 37 | 34 | 14 | 13 |
| KMPAH033_19_Rhyncomya_soyauxi | 50 | 43 | 41 | 40 | 39 | 38 | 37 | 34 | 14 | 13 |
| KMPDE178_19_Rhyncomya_soyauxi | 50 | 43 | 41 | 40 | 39 | 38 | 37 | 34 | 14 | 13 |
| KMPDB058_19_Rhyncomya_soyauxi | 50 | 43 | 41 | 40 | 39 | 38 | 37 | 34 | 14 | 13 |
| KMPDA136_19_Rhyncomya_soyauxi | 50 | 43 | 41 | 40 | 39 | 38 | 37 | 34 | 14 | 13 |
| KMPSF185_19_Rhyncomya_soyauxi | 50 | 43 | 41 | 40 | 39 | 38 | 37 | 34 | 14 | 13 |
| KMPIA267_18_Rhyncomya_soyauxi | 50 | 43 | 41 | 40 | 39 | 38 | 37 | 34 | 14 | 13 |
| KMPED049_18_Rhyncomya_soyauxi | 50 | 43 | 41 | 40 | 39 | 38 | 37 | 34 | 14 | 13 |
| KMPEM1160_19_Rhyncomya_soyauxi | 50 | 43 | 41 | 40 | 39 | 38 | 37 | 34 | 14 | 13 |
| KMPUD339_19_Rhyncomya_soyauxi | 50 | 43 | 41 | 40 | 39 | 38 | 37 | 34 | 14 | 13 |
| KMPIP260_19_Rhyncomya_soyauxi | 50 | 43 | 41 | 40 | 39 | 38 | 37 | 34 | 14 | 13 |
| KMPSP2616_19_Rhyncomya_soyauxi | 50 | 43 | 41 | 40 | 39 | 38 | 37 | 34 | 14 | 13 |
| KMPUD144_19_Rhyncomya_soyauxi | 50 | 43 | 41 | 40 | 39 | 38 | 37 | 34 | 14 | 13 |
| USA16_Rhyncomya_soyauxi_M_Kenya | 50 | 43 | 41 | 40 | 39 | 38 | 37 | 34 | 14 | 13 |
| GMSJK001_18_Rhiniinae | 51 | 44 | 42 | 41 | 40 | 39 | 38 | 35 | 15 | 13 |
| GMSJR1132_18_Rhiniinae | 51 | 44 | 42 | 41 | 40 | 39 | 38 | 35 | 15 | 13 |
| GMSJT006_18_Rhiniinae | 51 | 44 | 42 | 41 | 40 | 39 | 38 | 35 | 15 | 13 |
| GMSJV004_18_Rhiniinae | 51 | 44 | 42 | 41 | 40 | 39 | 38 | 35 | 15 | 13 |
| GMSJN092_18_Rhiniinae | 51 | 44 | 42 | 41 | 40 | 39 | 38 | 35 | 15 | 13 |
| GMSJS033_18_Rhiniinae | 51 | 44 | 42 | 41 | 40 | 39 | 38 | 35 | 15 | 13 |
| GMSJR1197_18_Rhiniinae | 51 | 44 | 42 | 41 | 40 | 39 | 38 | 35 | 15 | 13 |
| S19_Stomorhina_apta_F_Burundi | 52 | 45 | 43 | 42 | 41 | 40 | 39 | 36 | 16 | 14 |
| S20_Stomorhina_apta_F_Burundi | 52 | 45 | 43 | 42 | 41 | 40 | 39 | 36 | 16 | 14 |
| S28_Stomorhina_cf_malobana_F_Malawi | 52 | 45 | 43 | 42 | 41 | 40 | 39 | 36 | 16 | 14 |
| S30_Stomorhina_sp2_cf_malobana_F_Malawi | 52 | 45 | 43 | 42 | 41 | 40 | 39 | 36 | 16 | 14 |
| S31_Stomorhina_malobana_M_Malawi | 52 | 45 | 43 | 42 | 41 | 40 | 39 | 36 | 16 | 14 |
| S40_Stomorhina_sp2_cf_malobana_F_Tanzania | 52 | 45 | 43 | 42 | 41 | 40 | 39 | 36 | 16 | 14 |
| S42_Stomorhina_sp2_cf_malobana_F_Tanzania | 52 | 45 | 43 | 42 | 41 | 40 | 39 | 36 | 16 | 14 |
| MG967868_1_1_650_Strongyloneura_prolata | 53 | 46 | 44 | 43 | 42 | 41 | 40 | 37 | 17 | 15 |
| 1282A03_Isomyia_tristis_M_SouthAfrica | 54 | 47 | 45 | 44 | 43 | 42 | 41 | 38 | 18 | 16 |
| 1282A04_Isomyia_tristis_F_SouthAfrica | 54 | 47 | 45 | 44 | 43 | 42 | 41 | 38 | 18 | 16 |
| 1282B01_Isomyia_tristis_F_SouthAfrica | 54 | 47 | 45 | 44 | 43 | 42 | 41 | 38 | 18 | 16 |
| 1282B04_Isomyia_tristis_F_SouthAfrica | 54 | 47 | 45 | 44 | 43 | 42 | 41 | 38 | 18 | 16 |
| 1282B05_Isomyia_trisits_F_SouthAfrica | 54 | 47 | 45 | 44 | 43 | 42 | 41 | 38 | 18 | 16 |
| 1306F04_Isomyia_tristis_F_SouthAfrica | 54 | 47 | 45 | 44 | 43 | 42 | 41 | 38 | 18 | 16 |
| USA10_Isomyia_tristis_F_SouthAfrica | 54 | 47 | 45 | 44 | 43 | 42 | 41 | 38 | 18 | 16 |
| 1306A07_Isomyia_tristis_F_SouthAfrica | 54 | 47 | 45 | 44 | 43 | 42 | 41 | 38 | 18 | 16 |
| 1306B03_Isomyia_trisits_F_SouthAfrica | 54 | 47 | 45 | 44 | 43 | 42 | 41 | 38 | 18 | 16 |
| 1306B04_Isomyia_tristis_F_SouthAfrica | 54 | 47 | 45 | 44 | 43 | 42 | 41 | 38 | 18 | 16 |
| 1306C04_Isomyia_tristis_F_SouthAfrica | 54 | 47 | 45 | 44 | 43 | 42 | 41 | 38 | 18 | 16 |
| 1306C06_Isomyia_tristis_M_SouthAfrica | 54 | 47 | 45 | 44 | 43 | 42 | 41 | 38 | 18 | 16 |
| 1306C08_Isomyia_tristis_M_SouthAfrica | 54 | 47 | 45 | 44 | 43 | 42 | 41 | 38 | 18 | 16 |
| 1306F03_Isomyia_tristis_F_SouthAfrica | 54 | 47 | 45 | 44 | 43 | 42 | 41 | 38 | 18 | 16 |
| USA09_Isomyia_tristis_M_SouthAfrica | 54 | 47 | 45 | 44 | 43 | 42 | 41 | 38 | 18 | 16 |
| I20B_Isomyia_tristis_M_SouthAfrica | 54 | 47 | 45 | 44 | 43 | 42 | 41 | 38 | 18 | 16 |
| GMSAA1283_13_Rhiniinae | 54 | 47 | 45 | 44 | 43 | 42 | 41 | 38 | 18 | 16 |
| GMSAB2598_13_Rhiniinae | 54 | 47 | 45 | 44 | 43 | 42 | 41 | 38 | 18 | 16 |
| GMSAC2134_13_Rhiniinae | 54 | 47 | 45 | 44 | 43 | 42 | 41 | 38 | 18 | 16 |
| SAFRA2206_18_Rhiniinae | 54 | 47 | 45 | 44 | 43 | 42 | 41 | 38 | 18 | 16 |
| SAFRA2207_18_Rhiniinae | 54 | 47 | 45 | 44 | 43 | 42 | 41 | 38 | 18 | 16 |
| SAFRA2211_18_Rhiniinae | 54 | 47 | 45 | 44 | 43 | 42 | 41 | 38 | 18 | 16 |
| SAFRA2212_18_Rhiniinae | 54 | 47 | 45 | 44 | 43 | 42 | 41 | 38 | 18 | 16 |
| SAFRA2214_18_Rhiniinae | 54 | 47 | 45 | 44 | 43 | 42 | 41 | 38 | 18 | 16 |
| SAFRA2215_18_Rhiniinae | 54 | 47 | 45 | 44 | 43 | 42 | 41 | 38 | 18 | 16 |
| SAFRA2220_18_Rhiniinae | 54 | 47 | 45 | 44 | 43 | 42 | 41 | 38 | 18 | 16 |
| SAFRA2230_18_Rhiniinae | 54 | 47 | 45 | 44 | 43 | 42 | 41 | 38 | 18 | 16 |
| SAFRA2243_18_Rhiniinae | 54 | 47 | 45 | 44 | 43 | 42 | 41 | 38 | 18 | 16 |
| SAFRA2256_18_Rhiniinae | 54 | 47 | 45 | 44 | 43 | 42 | 41 | 38 | 18 | 16 |
| SAFRA3346_18_Rhiniinae | 54 | 47 | 45 | 44 | 43 | 42 | 41 | 38 | 18 | 16 |
| KVIS021_21_Rhiniinae | 55 | 48 | 46 | 45 | 44 | 43 | 42 | 38 | 18 | 16 |
| GMIAG050_17_Rhiniinae | 56 | 49 | 47 | 46 | 45 | 44 | 43 | 39 | 19 | 17 |
| GMIBA257_17_Rhiniinae | 56 | 49 | 47 | 46 | 45 | 44 | 43 | 39 | 19 | 17 |
| MN411061_1_1_650_Borbororhinia_bivittata | 57 | 50 | 48 | 47 | 46 | 45 | 44 | 40 | 19 | 17 |
| GMMGA479_14_Rhiniinae | 58 | 51 | 49 | 48 | 47 | 46 | 45 | 41 | 19 | 17 |
| LC549094_1_1_650_Borbororhinia_bivittata | 58 | 51 | 49 | 48 | 47 | 46 | 45 | 41 | 19 | 17 |
| LC549079_1_1_650_Borbororhinia_bivittata | 58 | 51 | 49 | 48 | 47 | 46 | 45 | 41 | 19 | 17 |
| KMPGQ433_19_Rhiniinae | 59 | 52 | 50 | 49 | 48 | 47 | 46 | 42 | 20 | 18 |
| KMPQT025_19_Rhiniinae | 59 | 52 | 50 | 49 | 48 | 47 | 46 | 42 | 20 | 18 |
| KMPZB214_19_Rhiniinae | 59 | 52 | 50 | 49 | 48 | 47 | 46 | 42 | 20 | 18 |
| KMPZB1230_19_Rhiniinae | 59 | 52 | 50 | 49 | 48 | 47 | 46 | 42 | 20 | 18 |
| Y44_Rhyncomya_sp8_F_Namibia | 60 | 53 | 51 | 50 | 49 | 48 | 47 | 43 | 21 | 19 |
| ISER004_05_Rhiniinae | 61 | 54 | 52 | 51 | 50 | 49 | 48 | 44 | 22 | 20 |
| T6_Thoracites_petersiana_F_SouthAfrica | 62 | 55 | 53 | 52 | 51 | 50 | 49 | 45 | 22 | 20 |
| T7_Thoracites_petersiana_M_SouthAfrica | 62 | 55 | 53 | 52 | 51 | 50 | 49 | 45 | 22 | 20 |
| JQ246694_1_42_691_Thoracites_sp | 63 | 56 | 54 | 53 | 52 | 51 | 50 | 46 | 23 | 21 |
| T3_Thoracites_sp1_F_SouthAfrica | 63 | 56 | 54 | 53 | 52 | 51 | 50 | 46 | 23 | 21 |
| T1_Thoracites_sp1_M_SouthAfrica | 63 | 56 | 54 | 53 | 52 | 51 | 50 | 46 | 23 | 21 |
| GMIAE017_17_Rhiniinae | 64 | 57 | 55 | 54 | 53 | 52 | 51 | 47 | 24 | 22 |
| GMIAJ036_17_Rhiniinae | 64 | 57 | 55 | 54 | 53 | 52 | 51 | 47 | 24 | 22 |
| GMIBA251_17_Rhiniinae | 64 | 57 | 55 | 54 | 53 | 52 | 51 | 47 | 24 | 22 |
| A3_Albaredaya_malgache_F_Madagascar | 65 | 58 | 56 | 55 | 54 | 53 | 52 | 48 | 25 | 23 |
| K5_Albaredaya_malgache_F_Madagascar | 65 | 58 | 56 | 55 | 54 | 53 | 52 | 48 | 25 | 23 |
| Y10_Zumba_antennalis_F_SouthAfrica | 66 | 59 | 57 | 56 | 55 | 54 | 53 | 49 | 26 | 24 |
| Z2_Zumba_antennalis_F_SouthAfrica | 66 | 59 | 57 | 56 | 55 | 54 | 53 | 49 | 26 | 24 |
| 1306A06_Isomyia_pubera_F_SouthAfrica | 67 | 60 | 58 | 57 | 56 | 55 | 54 | 50 | 27 | 25 |
| ETKD787_13_Rhiniinae | 67 | 60 | 58 | 57 | 56 | 55 | 54 | 50 | 27 | 25 |
| I5_Isomyia_pubera_M_SouthAfrica | 67 | 60 | 58 | 57 | 56 | 55 | 54 | 50 | 27 | 25 |
| USA07_Isomyia_pubera_F_SouthAfrica | 67 | 60 | 58 | 57 | 56 | 55 | 54 | 50 | 27 | 25 |
| A12_Vanemdenia_africana_F_Tanzania | 68 | 61 | 59 | 58 | 57 | 56 | 55 | 51 | 28 | 26 |
| C3_Cosmina_aenea_M_Namibia | 69 | 62 | 60 | 59 | 58 | 57 | 56 | 52 | 29 | 27 |
| C4_Cosmina_aenea_F_Namibia | 69 | 62 | 60 | 59 | 58 | 57 | 56 | 52 | 29 | 27 |
| ETKD541_12_Rhiniinae | 69 | 62 | 60 | 59 | 58 | 57 | 56 | 52 | 29 | 27 |
| GMSAP009_13_Rhiniinae | 70 | 63 | 61 | 60 | 59 | 58 | 57 | 53 | 29 | 27 |
| GMSAP011_13_Rhiniinae | 70 | 63 | 61 | 60 | 59 | 58 | 57 | 53 | 29 | 27 |
| Y64_Rhyncomya_sp6_M_Togo | 71 | 64 | 62 | 61 | 60 | 59 | 58 | 54 | 30 | 28 |
| Y65_Rhyncomya_sp6_F_Togo | 71 | 64 | 62 | 61 | 60 | 59 | 58 | 54 | 30 | 28 |
| C8_Cosmina_sp9_cf_testaceipes_M_Madagascar | 72 | 65 | 63 | 62 | 61 | 60 | 59 | 55 | 31 | 29 |
| 1306A08_Rhyncomya_disclusa_M_SouthAfrica | 73 | 66 | 64 | 63 | 62 | 61 | 60 | 56 | 32 | 30 |
| 1306E03_Rhyncomya_disclusa_F_SouthAfrica | 73 | 66 | 64 | 63 | 62 | 61 | 60 | 56 | 32 | 30 |
| 1306E04_Rhyncomya_disclusa_F_SouthAfrica | 73 | 66 | 64 | 63 | 62 | 61 | 60 | 56 | 32 | 30 |
| 1306E05_Rhyncomya_disclusa_F_SouthAfrica | 73 | 66 | 64 | 63 | 62 | 61 | 60 | 56 | 32 | 30 |
| GMBCD1162_15_Rhiniinae | 74 | 67 | 65 | 64 | 63 | 62 | 61 | 57 | 33 | 31 |
| GMBCD3155_15_Rhiniinae | 74 | 67 | 65 | 64 | 63 | 62 | 61 | 57 | 33 | 31 |
| GMBCF3679_15_Rhiniinae | 74 | 67 | 65 | 64 | 63 | 62 | 61 | 57 | 33 | 31 |
| GMBCD3143_15_Rhiniinae | 74 | 67 | 65 | 64 | 63 | 62 | 61 | 57 | 33 | 31 |
| GMBCF5874_15_Rhiniinae | 74 | 67 | 65 | 64 | 63 | 62 | 61 | 57 | 33 | 31 |
| GMBCH2736_15_Rhiniinae | 74 | 67 | 65 | 64 | 63 | 62 | 61 | 57 | 33 | 31 |
| GMBCI4695_15_Rhiniinae | 74 | 67 | 65 | 64 | 63 | 62 | 61 | 57 | 33 | 31 |
| MF804688_1_6_650_Isomyia_sp | 75 | 68 | 66 | 64 | 63 | 62 | 61 | 57 | 33 | 31 |
| KY031769_1_1_650_Isomyia_pseudolucilia | 76 | 69 | 67 | 65 | 64 | 63 | 62 | 58 | 33 | 31 |
| OR497843_1_1560_2209_Isomyia_nebulosa | 77 | 70 | 68 | 66 | 65 | 64 | 63 | 59 | 33 | 31 |
| JF439553_1_27_676_Isomyia_gomezmenori | 78 | 71 | 69 | 67 | 66 | 65 | 64 | 60 | 33 | 31 |
| KY031766_1_1_650_Isomyia_electa | 79 | 71 | 69 | 67 | 66 | 65 | 64 | 60 | 33 | 31 |
| KY031767_1_1_650_Isomyia_electa | 79 | 71 | 69 | 67 | 66 | 65 | 64 | 60 | 33 | 31 |
| KY031768_1_1_650_Isomyia_electa | 79 | 71 | 69 | 67 | 66 | 65 | 64 | 60 | 33 | 31 |
| GMMBI002_16_Rhiniinae | 80 | 72 | 70 | 68 | 67 | 66 | 65 | 61 | 34 | 32 |
| MBPT074_15_Rhiniinae | 80 | 72 | 70 | 68 | 67 | 66 | 65 | 61 | 34 | 32 |
| KY031773_1_1_650_Isomyia_complantenna | 81 | 73 | 71 | 69 | 68 | 67 | 66 | 62 | 35 | 33 |
| GMPBK162_18_Rhiniinae | 82 | 74 | 72 | 70 | 69 | 68 | 67 | 63 | 36 | 34 |
| GMPBK2200_18_Rhiniinae | 82 | 74 | 72 | 70 | 69 | 68 | 67 | 63 | 36 | 34 |
| GMPBK2780_18_Rhiniinae | 82 | 74 | 72 | 70 | 69 | 68 | 67 | 63 | 36 | 34 |
| GMPBL142_18_Rhiniinae | 82 | 74 | 72 | 70 | 69 | 68 | 67 | 63 | 36 | 34 |
| GMPBM134_18_Rhiniinae | 82 | 74 | 72 | 70 | 69 | 68 | 67 | 63 | 36 | 34 |
| GMPBN017_18_Rhiniinae | 82 | 74 | 72 | 70 | 69 | 68 | 67 | 63 | 36 | 34 |
| GMPBP099_18_Rhiniinae | 82 | 74 | 72 | 70 | 69 | 68 | 67 | 63 | 36 | 34 |
| GMPBK2183_18_Rhiniinae | 82 | 74 | 72 | 70 | 69 | 68 | 67 | 63 | 36 | 34 |
| GMPBK3490_18_Rhiniinae | 82 | 74 | 72 | 70 | 69 | 68 | 67 | 63 | 36 | 34 |
| GMPBL070_18_Rhiniinae | 82 | 74 | 72 | 70 | 69 | 68 | 67 | 63 | 36 | 34 |
| GMPBW002_18_Rhiniinae | 82 | 74 | 72 | 70 | 69 | 68 | 67 | 63 | 36 | 34 |
| MG968089_1_5_650_Thoracites_abdominalis | 83 | 75 | 73 | 71 | 70 | 69 | 68 | 64 | 37 | 35 |
| Y47_Rhyncomya_dasyops_F_SouthAfrica | 84 | 76 | 74 | 72 | 71 | 70 | 69 | 65 | 38 | 36 |
| 1306B05_Isomyia_natalensis_F_SouthAfrica | 85 | 77 | 75 | 73 | 72 | 71 | 70 | 66 | 39 | 37 |
| 1306C05_Isomyia_natalensis_F_SouthAfrica | 85 | 77 | 75 | 73 | 72 | 71 | 70 | 66 | 39 | 37 |
| 1306D04_Isomyia_natalensis_F_SouthAfrica | 85 | 77 | 75 | 73 | 72 | 71 | 70 | 66 | 39 | 37 |
| USA05_Isomyia_nataliensis_F_SouthAfrica | 85 | 77 | 75 | 73 | 72 | 71 | 70 | 66 | 39 | 37 |
| USA06_Isomyia_nataliensis_M_SouthAfrica | 85 | 77 | 75 | 73 | 72 | 71 | 70 | 66 | 39 | 37 |
| USA08_Isomyia_natalensis_F_SouthAfrica | 85 | 77 | 75 | 73 | 72 | 71 | 70 | 66 | 39 | 37 |
| 1306F01_Isomyia_natalensis_F_SouthAfrica | 85 | 77 | 75 | 73 | 72 | 71 | 70 | 66 | 39 | 37 |
| I17_Isomyia_distinguenda_F_Malawi | 86 | 78 | 76 | 74 | 73 | 72 | 71 | 67 | 40 | 38 |
| I19_Isomyia_distinguenda_F_Malawi | 86 | 78 | 76 | 74 | 73 | 72 | 71 | 67 | 40 | 38 |
| I8_Isomyia_distinguenda_M_SouthAfrica | 86 | 78 | 76 | 74 | 73 | 72 | 71 | 67 | 40 | 38 |
| I4_Isomyia_cuthbertsoni_M_SouthAfrica | 86 | 78 | 76 | 74 | 73 | 72 | 71 | 67 | 40 | 38 |
| I6_Isomyia_sp3_cf_cuthbertsoni_F_SouthAfrica | 86 | 78 | 76 | 74 | 73 | 72 | 71 | 67 | 40 | 38 |
| I9_Isomyia_sp3_cf_cuthbertsoni_F_SouthAfrica | 86 | 78 | 76 | 74 | 73 | 72 | 71 | 67 | 40 | 38 |
| I18_Isomyia_sp6_M_Togo | 87 | 79 | 77 | 75 | 74 | 73 | 72 | 68 | 41 | 39 |
| GQ409356_1_27_620_Rhyncomya_nigripes | 88 | 80 | 78 | 76 | 75 | 74 | 73 | 69 | 42 | 40 |
| I10_Isomyia_eos_F_SouthAfrica | 89 | 81 | 79 | 77 | 76 | 75 | 74 | 70 | 43 | 41 |
| KMPJL030_19_Rhiniinae | 90 | 82 | 80 | 78 | 77 | 76 | 75 | 71 | 44 | 42 |
| GQ409337_1_13_606_Metallea_erinacea | 91 | 83 | 81 | 79 | 78 | 77 | 76 | 72 | 45 | 43 |
| MN868726_1_1_650_Rhyncomya_felina | 92 | 84 | 82 | 80 | 79 | 78 | 77 | 73 | 46 | 44 |
| I11_Isomyia_darwini_F_SouthAfrica | 93 | 85 | 83 | 81 | 80 | 79 | 78 | 74 | 47 | 45 |
| KY031771_1_1_650_Isomyia_pseudonepalana | 94 | 86 | 84 | 82 | 81 | 80 | 79 | 75 | 48 | 46 |
| I15_Isomyia_sp2_M_Cameroon | 95 | 87 | 85 | 83 | 82 | 81 | 80 | 76 | 49 | 47 |
| MG967831_1_1_650_Alikangiella_rufithorax | 96 | 88 | 86 | 84 | 83 | 82 | 81 | 77 | 50 | 48 |
| GMMBI001_16_Rhiniinae | 97 | 89 | 87 | 85 | 84 | 83 | 82 | 78 | 51 | 49 |
| GMMBR149_16_Rhiniinae | 97 | 89 | 87 | 85 | 84 | 83 | 82 | 78 | 51 | 49 |
| KY031774_1_1_650_Isomyia_verirecta | 98 | 90 | 88 | 86 | 85 | 84 | 83 | 79 | 52 | 50 |
| DPSAF028_12_Rhiniinae | 99 | 91 | 89 | 87 | 86 | 85 | 84 | 80 | 53 | 51 |
| I1_Isomyia_dubiosa_F_Togo | 100 | 92 | 90 | 88 | 86 | 85 | 84 | 80 | 53 | 51 |
| I2_Isomyia_dubiosa_F_Togo | 100 | 92 | 90 | 88 | 86 | 85 | 84 | 80 | 53 | 51 |
| I12_Isomyia_dubiosa_F_Togo | 100 | 92 | 90 | 88 | 86 | 85 | 84 | 80 | 53 | 51 |
| I14_Isomyia_sp1_F_Uganda | 101 | 93 | 91 | 89 | 87 | 86 | 85 | 81 | 54 | 52 |
| KVIS016_21_Rhiniinae | 102 | 93 | 91 | 89 | 87 | 86 | 85 | 81 | 54 | 52 |
| B1_Trichoberia_sp1_M_SouthAfrica | 103 | 94 | 92 | 90 | 88 | 87 | 86 | 82 | 55 | 53 |
| PLABN218_19_Rhiniinae | 104 | 95 | 93 | 91 | 89 | 88 | 87 | 83 | 56 | 54 |
| PLLBG1418_20_Rhiniinae | 104 | 95 | 93 | 91 | 89 | 88 | 87 | 83 | 56 | 54 |
| PLRCH080_20_Rhiniinae | 104 | 95 | 93 | 91 | 89 | 88 | 87 | 83 | 56 | 54 |
| KMPIU596_19_Rhiniinae | 105 | 96 | 94 | 92 | 90 | 89 | 88 | 84 | 57 | 55 |

**- ABGD –**

20250228_Rhiniiae_all_data_1384.res.cvs

| prior | nbSubsetInitial | nbSubsetRecursive |
| --- | --- | --- |
| 0.001 | 694 | 694 |
| 0.001668 | 172 | 190 |
| 0.002783 | 172 | 181 |
| 0.004642 | 110 | 130 |
| 0.007743 | 109 | 120 |
| 0.012915 | 94 | 100 |
| 0.021544 | 89 | 94 |
| 0.035938 | 1 | 1 |

20250228_Rhiniiae_all_data_1384.disthist.svg


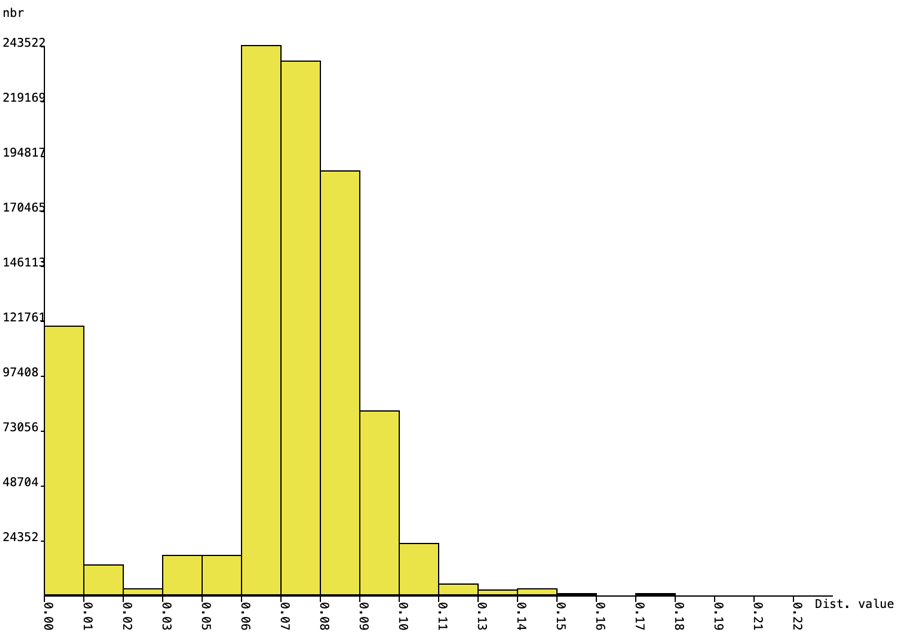


20250228_Rhiniiae_all_data_1384.rank.svg


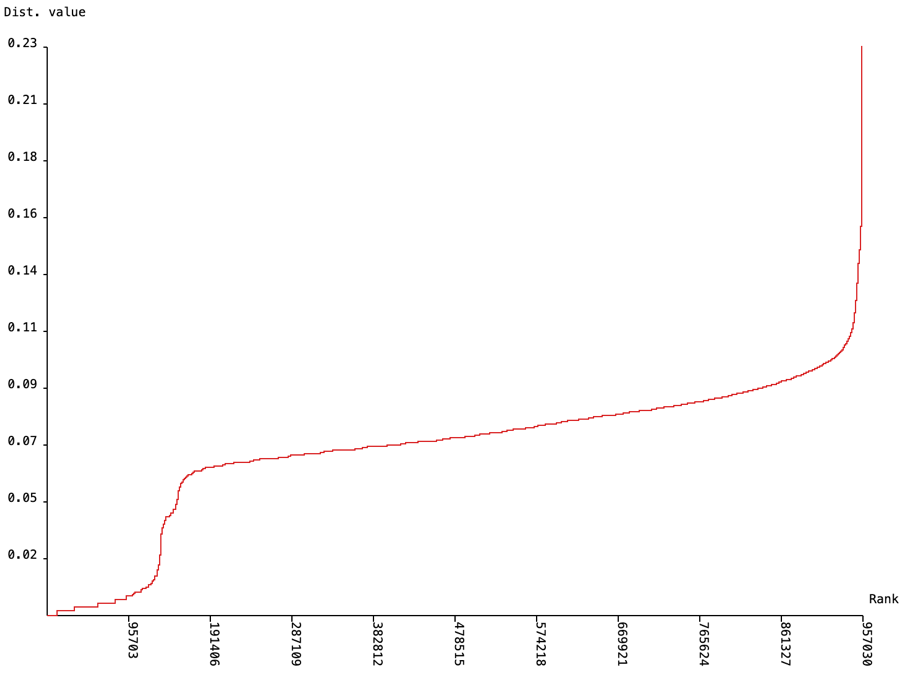


begin spart;

Project_name = 20250228_Rhiniiae_all_data_1384;

Date = 2025-03-13T21:42:55;

[Generated by ABGD with Distance K80 Kimura / MinSlope = 0.900000]

[WARNING: The sample names below may have been changed to fit SPART specification (only alphanumeric characters and _ )]

| **N_spartitions (6)** | **abgd_init_1** | **abgd_init_2** | **abgd_init_3** | **abgd_init_4** | **abgd_init_5** | **abgd_init_6** | **abgd_init_7** |
| --- | --- | --- | --- | --- | --- | --- | --- |
| **N_individuals** | 1384 | 1384 | 1384 | 1384 | 1384 | 1384 | 1384 |
| **N_subsets** | 694 | 172 | 172 | 110 | 109 | 94 | 89 |
| **Prior; Barcode gap distance** | 1.00E-03 | 1.67E-03 | 2.78E-03 | 4.64E-03 | 7.74E-03 | 1.29E-02 | 2.15E-02 |
| 1279A01_Stomorhina_cribrata_F_Ghana | 1 | 1 | 1 | 1 | 1 | 1 | 1 |
| 1279A02_Rhinia_apicalis_M_Ghana | 2 | 2 | 2 | 2 | 2 | 2 | 2 |
| 1279A03_Rhinia_apicalis_M_Togo | 3 | 2 | 2 | 2 | 2 | 2 | 2 |
| 1279A04_Rhinia_cf_apicalis_F_Togo | 4 | 2 | 2 | 2 | 2 | 2 | 2 |
| 1279A05_Rhinia_cf_apicalis_F_Togo | 5 | 2 | 2 | 2 | 2 | 2 | 2 |
| 1279A06_Rhinia_cf_apicalis_F_Togo | 6 | 2 | 2 | 2 | 2 | 2 | 2 |
| 1279A07_Stomorhina_chapini_F_Togo | 7 | 3 | 3 | 3 | 3 | 3 | 3 |
| 1279B01_Rhinia_cf_apicalis_F_Togo | 3 | 2 | 2 | 2 | 2 | 2 | 2 |
| 1279B02_Rhinia_sp1_M_Togo | 8 | 2 | 2 | 2 | 2 | 2 | 2 |
| 1279B03_Rhinia_cf_apicalis_F_Togo | 9 | 2 | 2 | 2 | 2 | 2 | 2 |
| 1279B06_Rhinia_cf_apicalis_F_Togo | 3 | 2 | 2 | 2 | 2 | 2 | 2 |
| 1279B07_Rhinia_sp3_M_Togo | 10 | 4 | 4 | 4 | 4 | 2 | 2 |
| 1279B08_Rhinia_cf_apicalis_F_Togo | 3 | 2 | 2 | 2 | 2 | 2 | 2 |
| 1279C01_Stegosoma_wellmani_M_Togo | 11 | 5 | 5 | 5 | 5 | 4 | 4 |
| 1279C02_Rhinia_cf_apicalis_F_Togo | 3 | 2 | 2 | 2 | 2 | 2 | 2 |
| 1279C03_Rhinia_cf_apicalis_F_Togo | 3 | 2 | 2 | 2 | 2 | 2 | 2 |
| 1279C04_Rhinia_apicalis_M_Togo | 3 | 2 | 2 | 2 | 2 | 2 | 2 |
| 1279C05_Rhinia_sp8_M_Togo | 12 | 6 | 6 | 4 | 4 | 2 | 2 |
| 1279C06_Stomorhina_cribrata_M_Togo | 1 | 1 | 1 | 1 | 1 | 1 | 1 |
| 1279C07_Rhinia_apicalis_M_Togo | 3 | 2 | 2 | 2 | 2 | 2 | 2 |
| 1279C08_Rhinia_cf_apicalis_F_Togo | 13 | 2 | 2 | 2 | 2 | 2 | 2 |
| 1279D01_Stomorhina_cribrata_F_Togo | 1 | 1 | 1 | 1 | 1 | 1 | 1 |
| 1279D02_Stomorhina_cribrata_M_Togo | 1 | 1 | 1 | 1 | 1 | 1 | 1 |
| 1279D05_Stomorhina_rugosa_M_Togo | 14 | 7 | 7 | 6 | 6 | 1 | 1 |
| 1279E01_Stomorhina_cribrata_F_Togo | 15 | 1 | 1 | 1 | 1 | 1 | 1 |
| 1279E02_Rhinia_cf_apicalis_F_Togo | 16 | 2 | 2 | 2 | 2 | 2 | 2 |
| 1279E03_Rhinia_apicalis_M_Ghana | 3 | 2 | 2 | 2 | 2 | 2 | 2 |
| 1279E04_Rhinia_cf_apicalis_F_Ghana | 17 | 2 | 2 | 2 | 2 | 2 | 2 |
| 1279E07_Stegosoma_vinculatum_M_Ghana | 18 | 8 | 8 | 7 | 7 | 5 | 5 |
| 1279E08_Rhinia_cf_apicalis_F_Ghana | 19 | 9 | 9 | 2 | 2 | 2 | 2 |
| 1279F01_Stegosoma_vinculatum_F_Ghana | 20 | 10 | 10 | 8 | 8 | 5 | 5 |
| 1279F02_Rhinia_cf_apicalis_F_Ghana | 13 | 2 | 2 | 2 | 2 | 2 | 2 |
| 1279F03_Rhinia_sp7_F_Ghana | 21 | 11 | 11 | 4 | 4 | 2 | 2 |
| 1279F04_Rhinia_sp4_F_Ghana | 22 | 12 | 12 | 9 | 9 | 2 | 2 |
| 1279F05_Rhinia_sp6_F_Ghana | 23 | 13 | 13 | 4 | 4 | 2 | 2 |
| 1279F06_Rhinia_apicalis_M_Ghana | 24 | 2 | 2 | 2 | 2 | 2 | 2 |
| 1282A01_Stomorhina_lunata_M_SouthAfrica | 25 | 14 | 14 | 10 | 10 | 6 | 6 |
| 1282A03_Isomyia_tristis_M_SouthAfrica | 26 | 15 | 15 | 11 | 11 | 7 | 7 |
| 1282A04_Isomyia_tristis_F_SouthAfrica | 26 | 15 | 15 | 11 | 11 | 7 | 7 |
| 1282A06_Stomorhina_lunata_F_SouthAfrica | 27 | 14 | 14 | 10 | 10 | 6 | 6 |
| 1282A08_Rhinia_cf_apicalis_M_SouthAfrica | 28 | 2 | 2 | 2 | 2 | 2 | 2 |
| 1282B01_Isomyia_tristis_F_SouthAfrica | 26 | 15 | 15 | 11 | 11 | 7 | 7 |
| 1282B04_Isomyia_tristis_F_SouthAfrica | 26 | 15 | 15 | 11 | 11 | 7 | 7 |
| 1282B05_Isomyia_trisits_F_SouthAfrica | 26 | 15 | 15 | 11 | 11 | 7 | 7 |
| 1282B06_Rhinia_apicalis_M_SouthAfrica | 3 | 2 | 2 | 2 | 2 | 2 | 2 |
| 1282C01_Stomorhina_lunata_M_SouthAfrica | 25 | 14 | 14 | 10 | 10 | 6 | 6 |
| 1282C02_Stomorhina_cribrata_M_SouthAfrica | 1 | 1 | 1 | 1 | 1 | 1 | 1 |
| 1282C03_Stomorhina_lunata_M_SouthAfrica | 25 | 14 | 14 | 10 | 10 | 6 | 6 |
| 1282C07_Rhinia_sp5_M_SouthAfrica | 29 | 16 | 16 | 12 | 4 | 2 | 2 |
| 1282C08_Stomorhina_lunata_F_SouthAfrica | 25 | 14 | 14 | 10 | 10 | 6 | 6 |
| 1282D01_Stomorhina_rugosa_M_SouthAfrica | 30 | 7 | 7 | 6 | 6 | 1 | 1 |
| 1282D02_Stomorhina_lunata_M_SouthAfrica | 25 | 14 | 14 | 10 | 10 | 6 | 6 |
| 1282D03_Stomorhina_rugosa_F_SouthAfrica | 31 | 7 | 7 | 6 | 6 | 1 | 1 |
| 1299A01_Stomorhina_lunata_F_SouthAfrica | 32 | 14 | 14 | 10 | 10 | 6 | 6 |
| 1299A02_Stomorhina_lunata_F_SouthAfrica | 25 | 14 | 14 | 10 | 10 | 6 | 6 |
| 1299A03_Stomorhina_lunata_F_SouthAfrica | 25 | 14 | 14 | 10 | 10 | 6 | 6 |
| 1306A01_Stomorhina_lunata_F_SouthAfrica | 25 | 14 | 14 | 10 | 10 | 6 | 6 |
| 1306A06_Isomyia_pubera_F_SouthAfrica | 33 | 17 | 17 | 13 | 12 | 8 | 8 |
| 1306A07_Isomyia_tristis_F_SouthAfrica | 34 | 18 | 18 | 11 | 11 | 7 | 7 |
| 1306A08_Rhyncomya_disclusa_M_SouthAfrica | 35 | 19 | 19 | 14 | 13 | 9 | 9 |
| 1306B01_Stomorhina_lunata_F_SouthAfrica | 36 | 14 | 14 | 10 | 10 | 6 | 6 |
| 1306B03_Isomyia_trisits_F_SouthAfrica | 34 | 18 | 18 | 11 | 11 | 7 | 7 |
| 1306B04_Isomyia_tristis_F_SouthAfrica | 34 | 18 | 18 | 11 | 11 | 7 | 7 |
| 1306B05_Isomyia_natalensis_F_SouthAfrica | 37 | 20 | 20 | 15 | 14 | 10 | 10 |
| 1306B06_Stomorhina_cribrata_F_SouthAfrica | 1 | 1 | 1 | 1 | 1 | 1 | 1 |
| 1306B08_Stomorhina_guttata_F_SouthAfrica | 38 | 21 | 21 | 16 | 15 | 11 | 11 |
| 1306C01_Stomorhina_lunata_M_SouthAfrica | 25 | 14 | 14 | 10 | 10 | 6 | 6 |
| 1306C03_Stomorhina_cribrata_M_SouthAfrica | 1 | 1 | 1 | 1 | 1 | 1 | 1 |
| 1306C04_Isomyia_tristis_F_SouthAfrica | 34 | 18 | 18 | 11 | 11 | 7 | 7 |
| 1306C05_Isomyia_natalensis_F_SouthAfrica | 39 | 20 | 20 | 15 | 14 | 10 | 10 |
| 1306C06_Isomyia_tristis_M_SouthAfrica | 34 | 18 | 18 | 11 | 11 | 7 | 7 |
| 1306C08_Isomyia_tristis_M_SouthAfrica | 34 | 18 | 18 | 11 | 11 | 7 | 7 |
| 1306D03_Stormohina_lunata_F_SouthAfrica | 25 | 14 | 14 | 10 | 10 | 6 | 6 |
| 1306D04_Isomyia_natalensis_F_SouthAfrica | 39 | 20 | 20 | 15 | 14 | 10 | 10 |
| 1306D05_Stomorhina_lunata_F_SouthAfrica | 25 | 14 | 14 | 10 | 10 | 6 | 6 |
| 1306E02_Stomorhina_cribrata_F_SouthAfrica | 1 | 1 | 1 | 1 | 1 | 1 | 1 |
| 1306E03_Rhyncomya_disclusa_F_SouthAfrica | 35 | 19 | 19 | 14 | 13 | 9 | 9 |
| 1306E04_Rhyncomya_disclusa_F_SouthAfrica | 35 | 19 | 19 | 14 | 13 | 9 | 9 |
| 1306E05_Rhyncomya_disclusa_F_SouthAfrica | 35 | 19 | 19 | 14 | 13 | 9 | 9 |
| 1306F01_Isomyia_natalensis_F_SouthAfrica | 40 | 20 | 20 | 15 | 14 | 10 | 10 |
| 1306F03_Isomyia_tristis_F_SouthAfrica | 34 | 18 | 18 | 11 | 11 | 7 | 7 |
| 1306F04_Isomyia_tristis_F_SouthAfrica | 26 | 15 | 15 | 11 | 11 | 7 | 7 |
| 1306F05_Stomorhina_cribrata_F_SouthAfrica | 41 | 1 | 1 | 1 | 1 | 1 | 1 |
| 1306F06_Stomorhina_cribrata_F_SouthAfrica | 1 | 1 | 1 | 1 | 1 | 1 | 1 |
| 1306F07_Stomorhina_cribrata_F_SouthAfrica | 1 | 1 | 1 | 1 | 1 | 1 | 1 |
| 1306F08_Stomorhina_cribrata_F_SouthAfrica | 1 | 1 | 1 | 1 | 1 | 1 | 1 |
| A10_Rhyncomya_sp14_F_Tanzania | 42 | 22 | 22 | 17 | 16 | 12 | 12 |
| A12_Vanemdenia_africana_F_Tanzania | 43 | 23 | 23 | 18 | 17 | 13 | 13 |
| A3_Albaredaya_malgache_F_Madagascar | 44 | 24 | 24 | 19 | 18 | 14 | 14 |
| A5_Cosmina_margaritae_F_Tanzania | 45 | 25 | 25 | 20 | 19 | 15 | 15 |
| A8_Cosmina_gracilis_M_Namibia | 46 | 26 | 26 | 21 | 20 | 15 | 15 |
| A9_Rhyncomya_interclusa_M_SouthAfrica | 47 | 27 | 27 | 22 | 21 | 16 | 16 |
| ASMII1048_22_Stomorhina | 48 | 28 | 28 | 23 | 22 | 17 | 17 |
| ASMII10886_22_Rhiniinae | 49 | 29 | 29 | 24 | 23 | 18 | 18 |
| ASMII10905_22_Rhiniinae | 49 | 29 | 29 | 24 | 23 | 18 | 18 |
| ASMII1109_22_Rhiniinae | 50 | 29 | 29 | 24 | 23 | 18 | 18 |
| ASMII1110_22_Rhiniinae | 51 | 29 | 29 | 24 | 23 | 18 | 18 |
| ASMII1114_22_Rhiniinae | 49 | 29 | 29 | 24 | 23 | 18 | 18 |
| ASMII1136_22_Rhiniinae | 52 | 29 | 29 | 24 | 23 | 18 | 18 |
| ASMII1138_22_Rhiniinae | 49 | 29 | 29 | 24 | 23 | 18 | 18 |
| ASMII1139_22_Rhiniinae | 53 | 29 | 29 | 24 | 23 | 18 | 18 |
| ASMII1152_22_Rhiniinae | 52 | 29 | 29 | 24 | 23 | 18 | 18 |
| ASMII1170_22_Stomorhina | 48 | 28 | 28 | 23 | 22 | 17 | 17 |
| ASMII1171_22_Stomorhina | 48 | 28 | 28 | 23 | 22 | 17 | 17 |
| ASMII1192_22_Rhiniinae | 51 | 29 | 29 | 24 | 23 | 18 | 18 |
| ASMII1195_22_Stomorhina | 48 | 28 | 28 | 23 | 22 | 17 | 17 |
| ASMII12149_22_Rhiniinae | 54 | 30 | 30 | 24 | 23 | 18 | 18 |
| ASMII12153_22_Stomorhina_discolor | 55 | 31 | 31 | 25 | 24 | 19 | 19 |
| ASMII1352_22_Rhiniinae | 56 | 32 | 32 | 24 | 23 | 18 | 18 |
| ASMII2368_22_Rhiniinae | 49 | 29 | 29 | 24 | 23 | 18 | 18 |
| ASMII2442_22_Rhiniinae | 57 | 29 | 29 | 24 | 23 | 18 | 18 |
| ASMII2448_22_Rhiniinae | 49 | 29 | 29 | 24 | 23 | 18 | 18 |
| ASMII2465_22_Rhiniinae | 49 | 29 | 29 | 24 | 23 | 18 | 18 |
| ASMII3354_22_Rhiniinae | 58 | 33 | 33 | 24 | 23 | 18 | 18 |
| ASMII3356_22_Rhiniinae | 59 | 33 | 33 | 24 | 23 | 18 | 18 |
| ASMII3388_22_Rhiniinae | 49 | 29 | 29 | 24 | 23 | 18 | 18 |
| ASMII3391_22_Rhiniinae | 60 | 29 | 29 | 24 | 23 | 18 | 18 |
| ASMII3436_22_Rhiniinae | 49 | 29 | 29 | 24 | 23 | 18 | 18 |
| ASMII3439_22_Rhiniinae | 61 | 29 | 29 | 24 | 23 | 18 | 18 |
| ASMII3510_22_Rhiniinae | 62 | 34 | 34 | 26 | 25 | 18 | 18 |
| ASMII4719_22_Rhiniinae | 63 | 35 | 35 | 24 | 23 | 18 | 18 |
| ASMII4720_22_Rhiniinae | 64 | 36 | 36 | 26 | 25 | 18 | 18 |
| ASMII4747_22_Rhiniinae | 65 | 36 | 36 | 26 | 25 | 18 | 18 |
| ASMII4749_22_Rhiniinae | 63 | 35 | 35 | 24 | 23 | 18 | 18 |
| ASMII4765_22_Rhiniinae | 64 | 36 | 36 | 26 | 25 | 18 | 18 |
| ASMII5628_22_Stomorhina_discolor | 66 | 31 | 31 | 25 | 24 | 19 | 19 |
| ASMII6818_22_Rhiniinae | 57 | 29 | 29 | 24 | 23 | 18 | 18 |
| ASMII6865_22_Rhiniinae | 49 | 29 | 29 | 24 | 23 | 18 | 18 |
| ASMII7251_22_Rhiniinae | 67 | 29 | 29 | 24 | 23 | 18 | 18 |
| ASMII7252_22_Rhiniinae | 68 | 29 | 29 | 24 | 23 | 18 | 18 |
| ASMII7268_22_Rhiniinae | 49 | 29 | 29 | 24 | 23 | 18 | 18 |
| ASMII7289_22_Rhiniinae | 49 | 29 | 29 | 24 | 23 | 18 | 18 |
| ASMII7310_22_Rhiniinae | 49 | 29 | 29 | 24 | 23 | 18 | 18 |
| ASMII7478_22_Stomorhina | 48 | 28 | 28 | 23 | 22 | 17 | 17 |
| ASMII8543_22_Stomorhina | 48 | 28 | 28 | 23 | 22 | 17 | 17 |
| ASMII8544_22_Stomorhina | 48 | 28 | 28 | 23 | 22 | 17 | 17 |
| ASMII9570_22_Stomorhina_discolor | 69 | 31 | 31 | 25 | 24 | 19 | 19 |
| AUSBC1389_12_Rhinia | 70 | 29 | 29 | 24 | 23 | 18 | 18 |
| AUSBC1393_12_Rhinia | 71 | 29 | 29 | 24 | 23 | 18 | 18 |
| AUSMG383_20_Rhiniinae | 72 | 29 | 29 | 24 | 23 | 18 | 18 |
| AUSMG385_20_Rhiniinae | 49 | 29 | 29 | 24 | 23 | 18 | 18 |
| AUSMG386_20_Rhiniinae | 49 | 29 | 29 | 24 | 23 | 18 | 18 |
| B1_Trichoberia_sp1_M_SouthAfrica | 73 | 37 | 37 | 27 | 26 | 20 | 20 |
| C10_Cosmina_sp3_F_Madagascar | 74 | 38 | 38 | 28 | 27 | 21 | 21 |
| C11_Cosmina_sp2_M_Madagascar | 75 | 39 | 39 | 29 | 28 | 22 | 22 |
| C18_Cosmina_sp5_F_Kenya | 76 | 40 | 40 | 30 | 29 | 23 | 23 |
| C19_Cosmina_sp6_M_Kenya | 77 | 41 | 41 | 31 | 30 | 24 | 24 |
| C20_Cosmina_sp1_cf_fuscipennis_F_SouthAfrica | 78 | 42 | 42 | 32 | 31 | 25 | 25 |
| C3_Cosmina_aenea_M_Namibia | 79 | 43 | 43 | 33 | 32 | 26 | 26 |
| C4_Cosmina_aenea_F_Namibia | 79 | 43 | 43 | 33 | 32 | 26 | 26 |
| C8_Cosmina_sp9_cf_testaceipes_M_Madagascar | 80 | 44 | 44 | 34 | 33 | 27 | 27 |
| DIQTB231_11_Stomorhina_discolor | 55 | 31 | 31 | 25 | 24 | 19 | 19 |
| DIQTB232_11_Stomorhina_discolor | 55 | 31 | 31 | 25 | 24 | 19 | 19 |
| DIQTB251_11_Stomorhina_discolor | 81 | 45 | 45 | 25 | 24 | 19 | 19 |
| DIQTB444_12_Rhiniinae | 82 | 29 | 29 | 24 | 23 | 18 | 18 |
| DIQTB510_12_Rhinia_apicalis | 83 | 46 | 46 | 35 | 34 | 28 | 28 |
| DIQTB559_12_Rhinia_apicalis | 83 | 46 | 46 | 35 | 34 | 28 | 28 |
| DIQTB560_12_Rhinia_apicalis | 83 | 46 | 46 | 35 | 34 | 28 | 28 |
| DIQTB580_12_Stomorhina_discolor | 55 | 31 | 31 | 25 | 24 | 19 | 19 |
| DPAS1068_11_Stomorhina | 48 | 28 | 28 | 23 | 22 | 17 | 17 |
| DPAST1393_12_Rhinia | 49 | 29 | 29 | 24 | 23 | 18 | 18 |
| DPSAF028_12_Rhiniinae | 84 | 47 | 47 | 36 | 35 | 29 | 29 |
| E1_Eurhyncomyia_diversicolor_M_Mozambique | 85 | 48 | 48 | 37 | 36 | 30 | 30 |
| E2_Eurhyncomyia_diversicolor_F_Mozambique | 85 | 48 | 48 | 37 | 36 | 30 | 30 |
| ETKD1032_13_Rhiniinae | 86 | 49 | 49 | 4 | 4 | 2 | 2 |
| ETKD1044_13_Rhiniinae | 87 | 50 | 50 | 38 | 37 | 31 | 31 |
| ETKD541_12_Rhiniinae | 88 | 51 | 51 | 33 | 32 | 26 | 26 |
| ETKD613_12_Rhiniinae | 89 | 52 | 52 | 4 | 4 | 2 | 2 |
| ETKD633_12_Rhiniinae | 90 | 53 | 53 | 39 | 38 | 2 | 2 |
| ETKD757_12_Rhiniinae | 91 | 7 | 7 | 6 | 6 | 1 | 1 |
| ETKD787_13_Rhiniinae | 33 | 17 | 17 | 13 | 12 | 8 | 8 |
| ETKD788_13_Rhiniinae | 92 | 2 | 2 | 2 | 2 | 2 | 2 |
| ETKD853_13_Rhiniinae | 91 | 7 | 7 | 6 | 6 | 1 | 1 |
| F2_Fainia_elongata_F_DRCongo | 93 | 54 | 54 | 38 | 37 | 31 | 31 |
| F20_Fainia_albitarsis_M_Tanzania | 94 | 55 | 55 | 38 | 37 | 31 | 31 |
| F3_Fainia_inexpectata_F_Malawi | 95 | 56 | 56 | 38 | 37 | 31 | 31 |
| F5_Fainia_elongata_M_Malawi | 96 | 54 | 54 | 38 | 37 | 31 | 31 |
| F6_Fainia_inexpectata_M_Kenya | 97 | 56 | 56 | 38 | 37 | 31 | 31 |
| G1_Stegosoma_bowdeni_M_Togo | 98 | 57 | 57 | 40 | 39 | 32 | 32 |
| G3_Stegosoma_bowdeni_F_Togo | 99 | 57 | 57 | 40 | 39 | 32 | 32 |
| G5_Stegosoma_vinculatum_F_SouthAfrica | 100 | 10 | 10 | 8 | 8 | 5 | 5 |
| G7_Stegosoma_vinculatum_M_SouthAfrica | 101 | 10 | 10 | 8 | 8 | 5 | 5 |
| G8_Stegosoma_sp1_cf_wellmani_F_DRCongo | 102 | 58 | 58 | 41 | 40 | 33 | 33 |
| GMAEA5279_22_Stomorhina | 48 | 28 | 28 | 23 | 22 | 17 | 17 |
| GMAMC010_15_Rhiniinae | 103 | 59 | 59 | 26 | 25 | 18 | 18 |
| GMAMH009_15_Rhiniinae | 49 | 29 | 29 | 24 | 23 | 18 | 18 |
| GMAMI025_15_Rhiniinae | 104 | 33 | 33 | 24 | 23 | 18 | 18 |
| GMAMJ027_15_Rhiniinae | 49 | 29 | 29 | 24 | 23 | 18 | 18 |
| GMAMJ034_15_Rhiniinae | 105 | 33 | 33 | 24 | 23 | 18 | 18 |
| GMAMJ186_15_Rhiniinae | 57 | 29 | 29 | 24 | 23 | 18 | 18 |
| GMAML005_15_Rhiniinae | 103 | 59 | 59 | 26 | 25 | 18 | 18 |
| GMAML006_15_Rhiniinae | 49 | 29 | 29 | 24 | 23 | 18 | 18 |
| GMAMM349_15_Rhiniinae | 106 | 29 | 29 | 24 | 23 | 18 | 18 |
| GMAMN526_15_Rhiniinae | 49 | 29 | 29 | 24 | 23 | 18 | 18 |
| GMAMS685_15_Rhiniinae | 49 | 29 | 29 | 24 | 23 | 18 | 18 |
| GMAMS688_15_Rhiniinae | 49 | 29 | 29 | 24 | 23 | 18 | 18 |
| GMAMS689_15_Rhiniinae | 107 | 33 | 33 | 24 | 23 | 18 | 18 |
| GMAMS735_15_Rhiniinae | 108 | 29 | 29 | 24 | 23 | 18 | 18 |
| GMAMS750_15_Rhiniinae | 49 | 29 | 29 | 24 | 23 | 18 | 18 |
| GMAMS766_15_Rhiniinae | 49 | 29 | 29 | 24 | 23 | 18 | 18 |
| GMAMT1493_16_Rhiniinae | 103 | 59 | 59 | 26 | 25 | 18 | 18 |
| GMAMT1494_16_Rhiniinae | 103 | 59 | 59 | 26 | 25 | 18 | 18 |
| GMAMT1495_16_Rhiniinae | 103 | 59 | 59 | 26 | 25 | 18 | 18 |
| GMAMT1498_16_Rhiniinae | 107 | 33 | 33 | 24 | 23 | 18 | 18 |
| GMASF011_17_Rhiniinae | 109 | 60 | 60 | 24 | 23 | 18 | 18 |
| GMBCC1933_15_Rhiniinae | 110 | 61 | 61 | 42 | 41 | 34 | 34 |
| GMBCC1944_15_Stomorhina_discolor | 111 | 31 | 31 | 25 | 24 | 19 | 19 |
| GMBCC1947_15_Rhiniinae | 112 | 62 | 62 | 43 | 42 | 35 | 35 |
| GMBCC3273_15_Stomorhina_discolor | 111 | 31 | 31 | 25 | 24 | 19 | 19 |
| GMBCD069_15_Rhiniinae | 113 | 62 | 62 | 43 | 42 | 35 | 35 |
| GMBCD1162_15_Rhiniinae | 114 | 63 | 63 | 44 | 43 | 36 | 36 |
| GMBCD1482_15_Rhiniinae | 112 | 62 | 62 | 43 | 42 | 35 | 35 |
| GMBCD1746_15_Stomorhina_discolor | 111 | 31 | 31 | 25 | 24 | 19 | 19 |
| GMBCD1761_15_Rhiniinae | 113 | 62 | 62 | 43 | 42 | 35 | 35 |
| GMBCD1894_15_Rhiniinae | 112 | 62 | 62 | 43 | 42 | 35 | 35 |
| GMBCD3143_15_Rhiniinae | 115 | 63 | 63 | 44 | 43 | 36 | 36 |
| GMBCD3155_15_Rhiniinae | 114 | 63 | 63 | 44 | 43 | 36 | 36 |
| GMBCE1745_15_Rhiniinae | 113 | 62 | 62 | 43 | 42 | 35 | 35 |
| GMBCE2167_15_Rhiniinae | 116 | 61 | 61 | 42 | 41 | 34 | 34 |
| GMBCE2722_15_Rhiniinae | 112 | 62 | 62 | 43 | 42 | 35 | 35 |
| GMBCE2931_15_Rhiniinae | 112 | 62 | 62 | 43 | 42 | 35 | 35 |
| GMBCE2938_15_Rhiniinae | 113 | 62 | 62 | 43 | 42 | 35 | 35 |
| GMBCE3416_15_Rhiniinae | 117 | 64 | 64 | 45 | 44 | 37 | 37 |
| GMBCE3750_15_Rhiniinae | 113 | 62 | 62 | 43 | 42 | 35 | 35 |
| GMBCF1172_15_Rhiniinae | 112 | 62 | 62 | 43 | 42 | 35 | 35 |
| GMBCF2805_15_Rhiniinae | 113 | 62 | 62 | 43 | 42 | 35 | 35 |
| GMBCF3180_15_Rhiniinae | 112 | 62 | 62 | 43 | 42 | 35 | 35 |
| GMBCF3679_15_Rhiniinae | 114 | 63 | 63 | 44 | 43 | 36 | 36 |
| GMBCF5409_15_Rhiniinae | 117 | 64 | 64 | 45 | 44 | 37 | 37 |
| GMBCF5874_15_Rhiniinae | 115 | 63 | 63 | 44 | 43 | 36 | 36 |
| GMBCH2736_15_Rhiniinae | 115 | 63 | 63 | 44 | 43 | 36 | 36 |
| GMBCI160_15_Stomorhina_discolor | 111 | 31 | 31 | 25 | 24 | 19 | 19 |
| GMBCI1616_15_Stomorhina_discolor | 111 | 31 | 31 | 25 | 24 | 19 | 19 |
| GMBCI2361_15_Stomorhina_discolor | 81 | 45 | 45 | 25 | 24 | 19 | 19 |
| GMBCI2386_15_Stomorhina_discolor | 118 | 45 | 45 | 25 | 24 | 19 | 19 |
| GMBCI4695_15_Rhiniinae | 115 | 63 | 63 | 44 | 43 | 36 | 36 |
| GMBCM2533_15_Stomorhina_discolor | 111 | 31 | 31 | 25 | 24 | 19 | 19 |
| GMBCN631_15_Rhiniinae | 112 | 62 | 62 | 43 | 42 | 35 | 35 |
| GMBCN894_15_Rhiniinae | 113 | 62 | 62 | 43 | 42 | 35 | 35 |
| GMCHB388_14_Stomorhina_discolor | 119 | 65 | 65 | 25 | 24 | 19 | 19 |
| GMCHE034_14_Stomorhina_discolor | 119 | 65 | 65 | 25 | 24 | 19 | 19 |
| GMCWK043_15_Rhiniinae | 120 | 29 | 29 | 24 | 23 | 18 | 18 |
| GMCWM164_15_Rhiniinae | 51 | 29 | 29 | 24 | 23 | 18 | 18 |
| GMCWN700_15_Rhiniinae | 121 | 29 | 29 | 24 | 23 | 18 | 18 |
| GMCWO139_15_Rhiniinae | 122 | 29 | 29 | 24 | 23 | 18 | 18 |
| GMCWO140_15_Rhiniinae | 123 | 66 | 66 | 24 | 23 | 18 | 18 |
| GMCWP067_15_Rhiniinae | 124 | 29 | 29 | 24 | 23 | 18 | 18 |
| GMEGD012_14_Rhiniinae | 125 | 1 | 1 | 1 | 1 | 1 | 1 |
| GMGMN1228_14_Stomorhina_lunata | 25 | 14 | 14 | 10 | 10 | 6 | 6 |
| GMIAE017_17_Rhiniinae | 126 | 67 | 67 | 46 | 45 | 38 | 38 |
| GMIAG050_17_Rhiniinae | 127 | 68 | 68 | 47 | 46 | 39 | 39 |
| GMIAJ036_17_Rhiniinae | 126 | 67 | 67 | 46 | 45 | 38 | 38 |
| GMIAK389_17_Stomorhina_discolor | 128 | 45 | 45 | 25 | 24 | 19 | 19 |
| GMIAK420_17_Stomorhina_discolor | 129 | 31 | 31 | 25 | 24 | 19 | 19 |
| GMIBA104_17_Stomorhina_discolor | 111 | 31 | 31 | 25 | 24 | 19 | 19 |
| GMIBA251_17_Rhiniinae | 130 | 67 | 67 | 46 | 45 | 38 | 38 |
| GMIBA257_17_Rhiniinae | 131 | 68 | 68 | 47 | 46 | 39 | 39 |
| GMKMA558_15_Rhiniinae | 132 | 69 | 69 | 48 | 47 | 40 | 40 |
| GMKMA582_15_Rhiniinae | 133 | 70 | 70 | 49 | 48 | 41 | 12 |
| GMKMA584_15_Rhyncomya_soyauxi | 134 | 71 | 71 | 50 | 49 | 42 | 41 |
| GMKMA593_15_Rhiniinae | 135 | 72 | 72 | 49 | 48 | 41 | 12 |
| GMKMB601_15_Rhiniinae | 136 | 73 | 73 | 17 | 16 | 12 | 12 |
| GMKMB609_15_Rhiniinae | 137 | 73 | 73 | 17 | 16 | 12 | 12 |
| GMKMD027_15_Rhyncomya_soyauxi | 138 | 71 | 71 | 50 | 49 | 42 | 41 |
| GMKMD029_15_Rhyncomya_soyauxi | 134 | 71 | 71 | 50 | 49 | 42 | 41 |
| GMKME194_15_Rhyncomya_soyauxi | 134 | 71 | 71 | 50 | 49 | 42 | 41 |
| GMKME204_15_Rhiniinae | 139 | 74 | 74 | 51 | 50 | 43 | 42 |
| GMKMF003_15_Rhyncomya_soyauxi | 134 | 71 | 71 | 50 | 49 | 42 | 41 |
| GMKMF022_15_Rhiniinae | 135 | 72 | 72 | 49 | 48 | 41 | 12 |
| GMKMG357_15_Rhiniinae | 140 | 70 | 70 | 49 | 48 | 41 | 12 |
| GMKMH199_15_Rhiniinae | 141 | 73 | 73 | 17 | 16 | 12 | 12 |
| GMKMI691_15_Rhiniinae | 142 | 75 | 75 | 51 | 50 | 43 | 42 |
| GMKMJ917_15_Rhiniinae | 135 | 72 | 72 | 49 | 48 | 41 | 12 |
| GMKMJ926_15_Rhiniinae | 135 | 72 | 72 | 49 | 48 | 41 | 12 |
| GMKMJ950_15_Rhyncomya_soyauxi | 134 | 71 | 71 | 50 | 49 | 42 | 41 |
| GMKML237_15_Rhiniinae | 143 | 72 | 72 | 49 | 48 | 41 | 12 |
| GMKML243_15_Rhyncomya_soyauxi | 144 | 71 | 71 | 50 | 49 | 42 | 41 |
| GMKMN024_15_Rhiniinae | 145 | 72 | 72 | 49 | 48 | 41 | 12 |
| GMKMN026_15_Rhiniinae | 146 | 73 | 73 | 17 | 16 | 12 | 12 |
| GMKMN028_15_Rhiniinae | 147 | 75 | 75 | 51 | 50 | 43 | 42 |
| GMKMS030_15_Rhyncomya_soyauxi | 134 | 71 | 71 | 50 | 49 | 42 | 41 |
| GMKMT173_15_Rhiniinae | 135 | 72 | 72 | 49 | 48 | 41 | 12 |
| GMKMT188_15_Rhiniinae | 148 | 69 | 69 | 48 | 47 | 40 | 40 |
| GMKMT195_15_Rhiniinae | 146 | 73 | 73 | 17 | 16 | 12 | 12 |
| GMKMT200_15_Rhiniinae | 149 | 70 | 70 | 49 | 48 | 41 | 12 |
| GMKMU108_15_Rhiniinae | 150 | 76 | 76 | 51 | 50 | 43 | 42 |
| GMKMU731_15_Rhiniinae | 135 | 72 | 72 | 49 | 48 | 41 | 12 |
| GMKMV452_15_Rhiniinae | 151 | 72 | 72 | 49 | 48 | 41 | 12 |
| GMKMW2218_15_Rhiniinae | 152 | 70 | 70 | 49 | 48 | 41 | 12 |
| GMKMW2269_15_Rhiniinae | 153 | 70 | 70 | 49 | 48 | 41 | 12 |
| GMKMY1974_15_Rhiniinae | 136 | 73 | 73 | 17 | 16 | 12 | 12 |
| GMKMY231_15_Rhyncomya_soyauxi | 134 | 71 | 71 | 50 | 49 | 42 | 41 |
| GMMBI001_16_Rhiniinae | 154 | 77 | 77 | 52 | 51 | 44 | 43 |
| GMMBI002_16_Rhiniinae | 155 | 78 | 78 | 53 | 52 | 45 | 44 |
| GMMBR149_16_Rhiniinae | 156 | 77 | 77 | 52 | 51 | 44 | 43 |
| GMMDB131_15_Rhiniinae | 157 | 79 | 79 | 54 | 53 | 46 | 45 |
| GMMDC250_15_Rhiniinae | 158 | 79 | 79 | 54 | 53 | 46 | 45 |
| GMMDD078_15_Rhiniinae | 159 | 79 | 79 | 54 | 53 | 46 | 45 |
| GMMDD083_15_Rhiniinae | 160 | 79 | 79 | 54 | 53 | 46 | 45 |
| GMMDE039_15_Rhiniinae | 159 | 79 | 79 | 54 | 53 | 46 | 45 |
| GMMDE041_15_Rhiniinae | 161 | 79 | 79 | 54 | 53 | 46 | 45 |
| GMMDE044_15_Rhiniinae | 162 | 79 | 79 | 54 | 53 | 46 | 45 |
| GMMDF170_15_Rhiniinae | 161 | 79 | 79 | 54 | 53 | 46 | 45 |
| GMMGA479_14_Rhiniinae | 163 | 80 | 80 | 55 | 54 | 47 | 46 |
| GMPBB006_18_Rhiniinae | 164 | 81 | 81 | 56 | 55 | 34 | 34 |
| GMPBD013_18_Rhiniinae | 165 | 82 | 82 | 57 | 56 | 48 | 47 |
| GMPBH538_18_Rhiniinae | 166 | 81 | 81 | 56 | 55 | 34 | 34 |
| GMPBK065_18_Rhiniinae | 167 | 83 | 83 | 58 | 57 | 49 | 48 |
| GMPBK157_18_Rhiniinae | 168 | 81 | 81 | 56 | 55 | 34 | 34 |
| GMPBK161_18_Rhiniinae | 169 | 81 | 81 | 56 | 55 | 34 | 34 |
| GMPBK162_18_Rhiniinae | 170 | 84 | 84 | 59 | 58 | 50 | 49 |
| GMPBK2143_18_Rhiniinae | 171 | 83 | 83 | 58 | 57 | 49 | 48 |
| GMPBK2174_18_Rhiniinae | 167 | 83 | 83 | 58 | 57 | 49 | 48 |
| GMPBK2181_18_Rhiniinae | 167 | 83 | 83 | 58 | 57 | 49 | 48 |
| GMPBK2183_18_Rhiniinae | 172 | 85 | 85 | 59 | 58 | 50 | 49 |
| GMPBK2200_18_Rhiniinae | 170 | 84 | 84 | 59 | 58 | 50 | 49 |
| GMPBK2212_18_Rhiniinae | 173 | 82 | 82 | 57 | 56 | 48 | 47 |
| GMPBK2780_18_Rhiniinae | 174 | 84 | 84 | 59 | 58 | 50 | 49 |
| GMPBK2803_18_Rhiniinae | 175 | 81 | 81 | 56 | 55 | 34 | 34 |
| GMPBK3490_18_Rhiniinae | 176 | 86 | 86 | 59 | 58 | 50 | 49 |
| GMPBL070_18_Rhiniinae | 177 | 87 | 87 | 59 | 58 | 50 | 49 |
| GMPBL142_18_Rhiniinae | 178 | 84 | 84 | 59 | 58 | 50 | 49 |
| GMPBM134_18_Rhiniinae | 179 | 84 | 84 | 59 | 58 | 50 | 49 |
| GMPBN017_18_Rhiniinae | 180 | 84 | 84 | 59 | 58 | 50 | 49 |
| GMPBP099_18_Rhiniinae | 181 | 84 | 84 | 59 | 58 | 50 | 49 |
| GMPBW002_18_Rhiniinae | 182 | 87 | 87 | 59 | 58 | 50 | 49 |
| GMSAA1283_13_Rhiniinae | 183 | 88 | 88 | 11 | 11 | 7 | 7 |
| GMSAB2598_13_Rhiniinae | 184 | 88 | 88 | 11 | 11 | 7 | 7 |
| GMSAC2134_13_Rhiniinae | 184 | 88 | 88 | 11 | 11 | 7 | 7 |
| GMSAP009_13_Rhiniinae | 185 | 89 | 89 | 60 | 59 | 51 | 50 |
| GMSAP011_13_Rhiniinae | 185 | 89 | 89 | 60 | 59 | 51 | 50 |
| GMSJK001_18_Rhiniinae | 186 | 90 | 90 | 61 | 60 | 52 | 51 |
| GMSJN092_18_Rhiniinae | 187 | 90 | 90 | 61 | 60 | 52 | 51 |
| GMSJR1132_18_Rhiniinae | 186 | 90 | 90 | 61 | 60 | 52 | 51 |
| GMSJR1197_18_Rhiniinae | 188 | 90 | 90 | 61 | 60 | 52 | 51 |
| GMSJS033_18_Rhiniinae | 187 | 90 | 90 | 61 | 60 | 52 | 51 |
| GMSJT006_18_Rhiniinae | 186 | 90 | 90 | 61 | 60 | 52 | 51 |
| GMSJV004_18_Rhiniinae | 186 | 90 | 90 | 61 | 60 | 52 | 51 |
| GQ409337_1_13_606_Metallea_erinacea | 189 | 91 | 91 | 62 | 61 | 53 | 52 |
| GQ409356_1_27_620_Rhyncomya_nigripes | 190 | 92 | 92 | 63 | 62 | 54 | 53 |
| GQ409375_1_13_606_Stomorhina_discolor | 191 | 93 | 93 | 25 | 24 | 19 | 19 |
| GU681898_1_1_650_Rhiniidae_sp | 165 | 82 | 82 | 57 | 56 | 48 | 47 |
| HM375982_1_1_650_Rhinia_apicalis | 83 | 46 | 46 | 35 | 34 | 28 | 28 |
| HM399341_1_2_650_Stomorhina_pollinosa | 192 | 94 | 94 | 23 | 22 | 17 | 17 |
| HM399342_1_1_632_Metallea_incisuralis | 193 | 29 | 29 | 24 | 23 | 18 | 18 |
| HM399345_1_1_650_Metallea_incisuralis | 194 | 29 | 29 | 24 | 23 | 18 | 18 |
| HM399346_1_1_650_Metallea_incisuralis | 195 | 29 | 29 | 24 | 23 | 18 | 18 |
| HQ561046_1_1_567_Stomorhina_discolor | 55 | 31 | 31 | 25 | 24 | 19 | 19 |
| HQ561056_1_1_650_Stomorhina_discolor | 55 | 31 | 31 | 25 | 24 | 19 | 19 |
| I1_Isomyia_dubiosa_F_Togo | 196 | 95 | 95 | 64 | 63 | 55 | 29 |
| I10_Isomyia_eos_F_SouthAfrica | 197 | 96 | 96 | 65 | 64 | 56 | 54 |
| I11_Isomyia_darwini_F_SouthAfrica | 198 | 97 | 97 | 66 | 65 | 57 | 55 |
| I12_Isomyia_dubiosa_F_Togo | 199 | 95 | 95 | 64 | 63 | 55 | 29 |
| I14_Isomyia_sp1_F_Uganda | 200 | 98 | 98 | 67 | 66 | 58 | 56 |
| I15_Isomyia_sp2_M_Cameroon | 201 | 99 | 99 | 68 | 67 | 59 | 57 |
| I17_Isomyia_distinguenda_F_Malawi | 202 | 100 | 100 | 69 | 68 | 60 | 58 |
| I18_Isomyia_sp6_M_Togo | 203 | 101 | 101 | 70 | 69 | 61 | 59 |
| I19_Isomyia_distinguenda_F_Malawi | 204 | 100 | 100 | 69 | 68 | 60 | 58 |
| I2_Isomyia_dubiosa_F_Togo | 196 | 95 | 95 | 64 | 63 | 55 | 29 |
| I20B_Isomyia_tristis_M_SouthAfrica | 205 | 18 | 18 | 11 | 11 | 7 | 7 |
| I4_Isomyia_cuthbertsoni_M_SouthAfrica | 206 | 102 | 102 | 71 | 70 | 60 | 58 |
| I5_Isomyia_pubera_M_SouthAfrica | 33 | 17 | 17 | 13 | 12 | 8 | 8 |
| I6_Isomyia_sp3_cf_cuthbertsoni_F_SouthAfrica | 206 | 102 | 102 | 71 | 70 | 60 | 58 |
| I8_Isomyia_distinguenda_M_SouthAfrica | 207 | 100 | 100 | 69 | 68 | 60 | 58 |
| I9_Isomyia_sp3_cf_cuthbertsoni_F_SouthAfrica | 206 | 102 | 102 | 71 | 70 | 60 | 58 |
| ISER004_05_Rhiniinae | 208 | 103 | 103 | 72 | 71 | 62 | 60 |
| JF439553_1_27_676_Isomyia_gomezmenori | 209 | 104 | 104 | 73 | 72 | 63 | 61 |
| JQ246691_1_45_694_Cosmina_fuscipennis | 78 | 42 | 42 | 32 | 31 | 25 | 25 |
| JQ246692_1_42_691_Rhinia_sp | 210 | 105 | 105 | 4 | 4 | 2 | 2 |
| JQ246693_1_42_691_Rhyncomya_soyauxi | 211 | 71 | 71 | 50 | 49 | 42 | 41 |
| JQ246694_1_42_691_Thoracites_sp | 212 | 106 | 106 | 74 | 73 | 64 | 62 |
| K3_Fainia_albitarsis_F_Tanzania | 94 | 55 | 55 | 38 | 37 | 31 | 31 |
| K4_Rhinia_sp9_F_Tanzania | 89 | 52 | 52 | 4 | 4 | 2 | 2 |
| K5_Albaredaya_malgache_F_Madagascar | 213 | 24 | 24 | 19 | 18 | 14 | 14 |
| K7_Fainia_albitarsis_F_Tanzania | 94 | 55 | 55 | 38 | 37 | 31 | 31 |
| KMPAB4626_18_Rhiniinae | 214 | 70 | 70 | 49 | 48 | 41 | 12 |
| KMPAB4671_18_Rhiniinae | 215 | 107 | 107 | 51 | 50 | 43 | 42 |
| KMPAD2407_19_Rhiniinae | 216 | 107 | 107 | 51 | 50 | 43 | 42 |
| KMPAH001_19_Rhyncomya_soyauxi | 217 | 71 | 71 | 50 | 49 | 42 | 41 |
| KMPAH007_19_Rhyncomya_soyauxi | 218 | 71 | 71 | 50 | 49 | 42 | 41 |
| KMPAH033_19_Rhyncomya_soyauxi | 219 | 71 | 71 | 50 | 49 | 42 | 41 |
| KMPAH034_19_Rhyncomya_soyauxi | 220 | 71 | 71 | 50 | 49 | 42 | 41 |
| KMPAH035_19_Rhyncomya_soyauxi | 221 | 71 | 71 | 50 | 49 | 42 | 41 |
| KMPAH036_19_Rhyncomya_soyauxi | 222 | 71 | 71 | 50 | 49 | 42 | 41 |
| KMPAH3838_19_Rhiniinae | 223 | 70 | 70 | 49 | 48 | 41 | 12 |
| KMPAH3840_19_Rhyncomya_soyauxi | 224 | 71 | 71 | 50 | 49 | 42 | 41 |
| KMPAH3841_19_Rhiniinae | 223 | 70 | 70 | 49 | 48 | 41 | 12 |
| KMPAH3842_19_Rhiniinae | 223 | 70 | 70 | 49 | 48 | 41 | 12 |
| KMPAH3843_19_Rhyncomya_soyauxi | 225 | 71 | 71 | 50 | 49 | 42 | 41 |
| KMPAH3845_19_Rhiniinae | 226 | 70 | 70 | 49 | 48 | 41 | 12 |
| KMPAJ027_19_Rhiniinae | 223 | 70 | 70 | 49 | 48 | 41 | 12 |
| KMPAJ1372_19_Rhiniinae | 227 | 107 | 107 | 51 | 50 | 43 | 42 |
| KMPAJ193_19_Rhiniinae | 228 | 107 | 107 | 51 | 50 | 43 | 42 |
| KMPAJ212_19_Rhiniinae | 229 | 107 | 107 | 51 | 50 | 43 | 42 |
| KMPAL1228_19_Rhyncomya_soyauxi | 217 | 71 | 71 | 50 | 49 | 42 | 41 |
| KMPAL1251_19_Rhiniinae | 230 | 107 | 107 | 51 | 50 | 43 | 42 |
| KMPAL1255_19_Rhiniinae | 231 | 107 | 107 | 51 | 50 | 43 | 42 |
| KMPAL1308_19_Rhiniinae | 232 | 108 | 108 | 75 | 74 | 65 | 12 |
| KMPAL168_19_Rhyncomya_soyauxi | 233 | 71 | 71 | 50 | 49 | 42 | 41 |
| KMPAL172_19_Rhiniinae | 234 | 107 | 107 | 51 | 50 | 43 | 42 |
| KMPAL3472_19_Rhyncomya_soyauxi | 221 | 71 | 71 | 50 | 49 | 42 | 41 |
| KMPAL3474_19_Rhyncomya_soyauxi | 221 | 71 | 71 | 50 | 49 | 42 | 41 |
| KMPAL3481_19_Rhiniinae | 223 | 70 | 70 | 49 | 48 | 41 | 12 |
| KMPAL3492_19_Rhiniinae | 223 | 70 | 70 | 49 | 48 | 41 | 12 |
| KMPAL3498_19_Rhyncomya_soyauxi | 235 | 71 | 71 | 50 | 49 | 42 | 41 |
| KMPAL3499_19_Rhiniinae | 236 | 69 | 69 | 48 | 47 | 40 | 40 |
| KMPAL3503_19_Rhiniinae | 237 | 70 | 70 | 49 | 48 | 41 | 12 |
| KMPAL3513_19_Rhiniinae | 238 | 70 | 70 | 49 | 48 | 41 | 12 |
| KMPAL3514_19_Rhiniinae | 223 | 70 | 70 | 49 | 48 | 41 | 12 |
| KMPAL3515_19_Rhyncomya_soyauxi | 221 | 71 | 71 | 50 | 49 | 42 | 41 |
| KMPAL3516_19_Rhyncomya_soyauxi | 239 | 71 | 71 | 50 | 49 | 42 | 41 |
| KMPAL3523_19_Rhiniinae | 240 | 107 | 107 | 51 | 50 | 43 | 42 |
| KMPAL3530_19_Rhiniinae | 223 | 70 | 70 | 49 | 48 | 41 | 12 |
| KMPAL3533_19_Rhyncomya_soyauxi | 241 | 71 | 71 | 50 | 49 | 42 | 41 |
| KMPAL3541_19_Rhiniinae | 242 | 70 | 70 | 49 | 48 | 41 | 12 |
| KMPAL3545_19_Rhiniinae | 243 | 70 | 70 | 49 | 48 | 41 | 12 |
| KMPAL3552_19_Rhiniinae | 244 | 107 | 107 | 51 | 50 | 43 | 42 |
| KMPAL3561_19_Rhyncomya_soyauxi | 221 | 71 | 71 | 50 | 49 | 42 | 41 |
| KMPAL3568_19_Rhyncomya_soyauxi | 221 | 71 | 71 | 50 | 49 | 42 | 41 |
| KMPAL3574_19_Rhiniinae | 223 | 70 | 70 | 49 | 48 | 41 | 12 |
| KMPAL3593_19_Rhiniinae | 214 | 70 | 70 | 49 | 48 | 41 | 12 |
| KMPAL3764_19_Rhiniinae | 223 | 70 | 70 | 49 | 48 | 41 | 12 |
| KMPAL3769_19_Rhiniinae | 226 | 70 | 70 | 49 | 48 | 41 | 12 |
| KMPAL3773_19_Rhiniinae | 226 | 70 | 70 | 49 | 48 | 41 | 12 |
| KMPAL3775_19_Rhiniinae | 245 | 70 | 70 | 49 | 48 | 41 | 12 |
| KMPAL3778_19_Rhiniinae | 246 | 70 | 70 | 49 | 48 | 41 | 12 |
| KMPAN1048_19_Rhiniinae | 247 | 107 | 107 | 51 | 50 | 43 | 42 |
| KMPAN294_19_Rhyncomya_soyauxi | 221 | 71 | 71 | 50 | 49 | 42 | 41 |
| KMPAN295_19_Rhyncomya_soyauxi | 248 | 71 | 71 | 50 | 49 | 42 | 41 |
| KMPAN305_19_Rhyncomya_soyauxi | 249 | 71 | 71 | 50 | 49 | 42 | 41 |
| KMPAN323_19_Rhyncomya_soyauxi | 222 | 71 | 71 | 50 | 49 | 42 | 41 |
| KMPAN359_19_Rhyncomya_soyauxi | 250 | 71 | 71 | 50 | 49 | 42 | 41 |
| KMPBB317_18_Rhyncomya_soyauxi | 250 | 71 | 71 | 50 | 49 | 42 | 41 |
| KMPCA008_18_Rhiniinae | 251 | 107 | 107 | 51 | 50 | 43 | 42 |
| KMPCK025_19_Rhiniinae | 252 | 70 | 70 | 49 | 48 | 41 | 12 |
| KMPCL053_19_Rhiniinae | 253 | 107 | 107 | 51 | 50 | 43 | 42 |
| KMPCN128_19_Rhiniinae | 254 | 107 | 107 | 51 | 50 | 43 | 42 |
| KMPCN343_19_Rhyncomya_soyauxi | 255 | 71 | 71 | 50 | 49 | 42 | 41 |
| KMPCO422_19_Rhiniinae | 214 | 70 | 70 | 49 | 48 | 41 | 12 |
| KMPCO426_19_Rhiniinae | 256 | 70 | 70 | 49 | 48 | 41 | 12 |
| KMPCO427_19_Rhiniinae | 232 | 108 | 108 | 75 | 74 | 65 | 12 |
| KMPCP048_19_Rhiniinae | 257 | 107 | 107 | 51 | 50 | 43 | 42 |
| KMPCP049_19_Rhiniinae | 258 | 107 | 107 | 51 | 50 | 43 | 42 |
| KMPCP586_19_Rhiniinae | 223 | 70 | 70 | 49 | 48 | 41 | 12 |
| KMPCP592_19_Rhyncomya_soyauxi | 224 | 71 | 71 | 50 | 49 | 42 | 41 |
| KMPCP597_19_Rhyncomya_soyauxi | 225 | 71 | 71 | 50 | 49 | 42 | 41 |
| KMPCP599_19_Rhiniinae | 232 | 108 | 108 | 75 | 74 | 65 | 12 |
| KMPCP600_19_Rhyncomya_soyauxi | 225 | 71 | 71 | 50 | 49 | 42 | 41 |
| KMPCP602_19_Rhyncomya_soyauxi | 221 | 71 | 71 | 50 | 49 | 42 | 41 |
| KMPDA136_19_Rhyncomya_soyauxi | 259 | 71 | 71 | 50 | 49 | 42 | 41 |
| KMPDA143_19_Rhiniinae | 260 | 107 | 107 | 51 | 50 | 43 | 42 |
| KMPDA277_19_Rhiniinae | 232 | 108 | 108 | 75 | 74 | 65 | 12 |
| KMPDA343_19_Rhiniinae | 261 | 107 | 107 | 51 | 50 | 43 | 42 |
| KMPDB058_19_Rhyncomya_soyauxi | 262 | 71 | 71 | 50 | 49 | 42 | 41 |
| KMPDB060_19_Rhyncomya_soyauxi | 263 | 71 | 71 | 50 | 49 | 42 | 41 |
| KMPDB257_19_Rhiniinae | 232 | 108 | 108 | 75 | 74 | 65 | 12 |
| KMPDC024_19_Rhyncomya_soyauxi | 264 | 71 | 71 | 50 | 49 | 42 | 41 |
| KMPDC038_19_Rhyncomya_soyauxi | 265 | 71 | 71 | 50 | 49 | 42 | 41 |
| KMPDD073_19_Rhyncomya_soyauxi | 225 | 71 | 71 | 50 | 49 | 42 | 41 |
| KMPDD074_19_Rhiniinae | 223 | 70 | 70 | 49 | 48 | 41 | 12 |
| KMPDD117_19_Rhiniinae | 266 | 108 | 108 | 75 | 74 | 65 | 12 |
| KMPDD134_19_Rhiniinae | 267 | 70 | 70 | 49 | 48 | 41 | 12 |
| KMPDD145_19_Rhiniinae | 232 | 108 | 108 | 75 | 74 | 65 | 12 |
| KMPDD207_19_Rhyncomya_soyauxi | 268 | 71 | 71 | 50 | 49 | 42 | 41 |
| KMPDE072_19_Rhiniinae | 269 | 70 | 70 | 49 | 48 | 41 | 12 |
| KMPDE103_19_Rhyncomya_soyauxi | 270 | 71 | 71 | 50 | 49 | 42 | 41 |
| KMPDE117_19_Rhyncomya_soyauxi | 221 | 71 | 71 | 50 | 49 | 42 | 41 |
| KMPDE165_19_Rhiniinae | 1 | 1 | 1 | 1 | 1 | 1 | 1 |
| KMPDE167_19_Rhyncomya_soyauxi | 221 | 71 | 71 | 50 | 49 | 42 | 41 |
| KMPDE178_19_Rhyncomya_soyauxi | 271 | 71 | 71 | 50 | 49 | 42 | 41 |
| KMPDE961_19_Rhiniinae | 272 | 107 | 107 | 51 | 50 | 43 | 42 |
| KMPDF1858_19_Rhiniinae | 273 | 69 | 69 | 48 | 47 | 40 | 40 |
| KMPDF1861_19_Rhiniinae | 274 | 107 | 107 | 51 | 50 | 43 | 42 |
| KMPDF1896_19_Rhiniinae | 275 | 70 | 70 | 49 | 48 | 41 | 12 |
| KMPDF1899_19_Rhyncomya_soyauxi | 221 | 71 | 71 | 50 | 49 | 42 | 41 |
| KMPDG117_19_Rhiniinae | 226 | 70 | 70 | 49 | 48 | 41 | 12 |
| KMPDG148_19_Rhyncomya_soyauxi | 221 | 71 | 71 | 50 | 49 | 42 | 41 |
| KMPDG151_19_Rhiniinae | 236 | 69 | 69 | 48 | 47 | 40 | 40 |
| KMPDG163_19_Rhiniinae | 276 | 72 | 72 | 49 | 48 | 41 | 12 |
| KMPDG167_19_Rhiniinae | 277 | 107 | 107 | 51 | 50 | 43 | 42 |
| KMPDG703_19_Rhiniinae | 278 | 107 | 107 | 51 | 50 | 43 | 42 |
| KMPDH061_19_Rhyncomya_soyauxi | 224 | 71 | 71 | 50 | 49 | 42 | 41 |
| KMPDH063_19_Rhiniinae | 279 | 69 | 69 | 48 | 47 | 40 | 40 |
| KMPDH073_19_Rhiniinae | 223 | 70 | 70 | 49 | 48 | 41 | 12 |
| KMPDH075_19_Rhiniinae | 280 | 70 | 70 | 49 | 48 | 41 | 12 |
| KMPDI162_19_Rhiniinae | 281 | 107 | 107 | 51 | 50 | 43 | 42 |
| KMPDI163_19_Rhiniinae | 282 | 70 | 70 | 49 | 48 | 41 | 12 |
| KMPDI168_19_Rhiniinae | 283 | 108 | 108 | 75 | 74 | 65 | 12 |
| KMPDI181_19_Rhiniinae | 236 | 69 | 69 | 48 | 47 | 40 | 40 |
| KMPDI190_19_Rhiniinae | 284 | 70 | 70 | 49 | 48 | 41 | 12 |
| KMPDI202_19_Rhyncomya_soyauxi | 225 | 71 | 71 | 50 | 49 | 42 | 41 |
| KMPDI213_19_Rhiniinae | 226 | 70 | 70 | 49 | 48 | 41 | 12 |
| KMPDI220_19_Rhiniinae | 285 | 70 | 70 | 49 | 48 | 41 | 12 |
| KMPDI407_19_Rhiniinae | 232 | 108 | 108 | 75 | 74 | 65 | 12 |
| KMPDI414_19_Rhiniinae | 286 | 70 | 70 | 49 | 48 | 41 | 12 |
| KMPDJ1000_19_Rhiniinae | 287 | 107 | 107 | 51 | 50 | 43 | 42 |
| KMPDJ1460_19_Rhiniinae | 288 | 107 | 107 | 51 | 50 | 43 | 42 |
| KMPDJ182_19_Rhiniinae | 223 | 70 | 70 | 49 | 48 | 41 | 12 |
| KMPDJ186_19_Rhiniinae | 226 | 70 | 70 | 49 | 48 | 41 | 12 |
| KMPDJ188_19_Rhiniinae | 214 | 70 | 70 | 49 | 48 | 41 | 12 |
| KMPDJ189_19_Rhiniinae | 289 | 70 | 70 | 49 | 48 | 41 | 12 |
| KMPDJ192_19_Rhiniinae | 290 | 70 | 70 | 49 | 48 | 41 | 12 |
| KMPDJ197_19_Rhiniinae | 223 | 70 | 70 | 49 | 48 | 41 | 12 |
| KMPDJ208_19_Rhiniinae | 223 | 70 | 70 | 49 | 48 | 41 | 12 |
| KMPDJ214_19_Rhiniinae | 223 | 70 | 70 | 49 | 48 | 41 | 12 |
| KMPDJ2238_19_Rhiniinae | 223 | 70 | 70 | 49 | 48 | 41 | 12 |
| KMPDJ456_19_Rhiniinae | 278 | 107 | 107 | 51 | 50 | 43 | 42 |
| KMPDJ929_19_Rhiniinae | 291 | 109 | 109 | 21 | 20 | 15 | 15 |
| KMPDM072_19_Rhiniinae | 292 | 70 | 70 | 49 | 48 | 41 | 12 |
| KMPEA132_18_Rhiniinae | 293 | 70 | 70 | 49 | 48 | 41 | 12 |
| KMPEC632_18_Rhiniinae | 272 | 107 | 107 | 51 | 50 | 43 | 42 |
| KMPED003_18_Rhyncomya_soyauxi | 294 | 71 | 71 | 50 | 49 | 42 | 41 |
| KMPED012_18_Rhyncomya_soyauxi | 225 | 71 | 71 | 50 | 49 | 42 | 41 |
| KMPED013_18_Rhiniinae | 236 | 69 | 69 | 48 | 47 | 40 | 40 |
| KMPED043_18_Rhiniinae | 295 | 110 | 110 | 76 | 75 | 66 | 63 |
| KMPED049_18_Rhyncomya_soyauxi | 296 | 71 | 71 | 50 | 49 | 42 | 41 |
| KMPED088_18_Rhiniinae | 297 | 107 | 107 | 51 | 50 | 43 | 42 |
| KMPEE056_18_Rhiniinae | 298 | 107 | 107 | 51 | 50 | 43 | 42 |
| KMPEF308_19_Rhiniinae | 299 | 107 | 107 | 51 | 50 | 43 | 42 |
| KMPEG065_19_Rhyncomya_soyauxi | 225 | 71 | 71 | 50 | 49 | 42 | 41 |
| KMPEG070_19_Rhyncomya_soyauxi | 221 | 71 | 71 | 50 | 49 | 42 | 41 |
| KMPEH241_19_Rhiniinae | 295 | 110 | 110 | 76 | 75 | 66 | 63 |
| KMPEH283_19_Rhiniinae | 223 | 70 | 70 | 49 | 48 | 41 | 12 |
| KMPEH284_19_Rhiniinae | 236 | 69 | 69 | 48 | 47 | 40 | 40 |
| KMPEH285_19_Rhiniinae | 300 | 69 | 69 | 48 | 47 | 40 | 40 |
| KMPEH286_19_Rhiniinae | 236 | 69 | 69 | 48 | 47 | 40 | 40 |
| KMPEK756_19_Rhiniinae | 301 | 107 | 107 | 51 | 50 | 43 | 42 |
| KMPEL052_19_Rhiniinae | 237 | 70 | 70 | 49 | 48 | 41 | 12 |
| KMPEM1160_19_Rhyncomya_soyauxi | 302 | 71 | 71 | 50 | 49 | 42 | 41 |
| KMPEO1242_19_Rhiniinae | 303 | 70 | 70 | 49 | 48 | 41 | 12 |
| KMPFB141_18_Rhiniinae | 304 | 107 | 107 | 51 | 50 | 43 | 42 |
| KMPFE375_18_Rhiniinae | 305 | 107 | 107 | 51 | 50 | 43 | 42 |
| KMPFH092_19_Rhiniinae | 306 | 107 | 107 | 51 | 50 | 43 | 42 |
| KMPFH216_19_Rhiniinae | 307 | 107 | 107 | 51 | 50 | 43 | 42 |
| KMPFI125_19_Rhiniinae | 308 | 107 | 107 | 51 | 50 | 43 | 42 |
| KMPFI133_19_Rhiniinae | 258 | 107 | 107 | 51 | 50 | 43 | 42 |
| KMPFJ091_19_Rhiniinae | 91 | 7 | 7 | 6 | 6 | 1 | 1 |
| KMPFN007_19_Rhiniinae | 236 | 69 | 69 | 48 | 47 | 40 | 40 |
| KMPFR135_19_Rhiniinae | 214 | 70 | 70 | 49 | 48 | 41 | 12 |
| KMPFR137_19_Rhiniinae | 237 | 70 | 70 | 49 | 48 | 41 | 12 |
| KMPFR138_19_Rhiniinae | 223 | 70 | 70 | 49 | 48 | 41 | 12 |
| KMPFR141_19_Rhiniinae | 223 | 70 | 70 | 49 | 48 | 41 | 12 |
| KMPFS030_19_Rhiniinae | 309 | 70 | 70 | 49 | 48 | 41 | 12 |
| KMPFS032_19_Rhiniinae | 135 | 72 | 72 | 49 | 48 | 41 | 12 |
| KMPFS033_19_Rhiniinae | 214 | 70 | 70 | 49 | 48 | 41 | 12 |
| KMPFS037_19_Rhiniinae | 310 | 70 | 70 | 49 | 48 | 41 | 12 |
| KMPFT011_19_Rhiniinae | 223 | 70 | 70 | 49 | 48 | 41 | 12 |
| KMPFT012_19_Rhiniinae | 311 | 70 | 70 | 49 | 48 | 41 | 12 |
| KMPGA139_18_Rhiniinae | 312 | 107 | 107 | 51 | 50 | 43 | 42 |
| KMPGB135_18_Rhiniinae | 313 | 107 | 107 | 51 | 50 | 43 | 42 |
| KMPGM300_19_Rhiniinae | 223 | 70 | 70 | 49 | 48 | 41 | 12 |
| KMPGM302_19_Rhyncomya_soyauxi | 225 | 71 | 71 | 50 | 49 | 42 | 41 |
| KMPGO035_19_Rhiniinae | 314 | 107 | 107 | 51 | 50 | 43 | 42 |
| KMPGP111_19_Rhyncomya_soyauxi | 315 | 71 | 71 | 50 | 49 | 42 | 41 |
| KMPGQ161_19_Rhiniinae | 316 | 70 | 70 | 49 | 48 | 41 | 12 |
| KMPGQ432_19_Rhiniinae | 317 | 70 | 70 | 49 | 48 | 41 | 12 |
| KMPGQ433_19_Rhiniinae | 318 | 111 | 111 | 77 | 76 | 67 | 64 |
| KMPGS994_19_Rhiniinae | 92 | 2 | 2 | 2 | 2 | 2 | 2 |
| KMPGT039_19_Rhiniinae | 214 | 70 | 70 | 49 | 48 | 41 | 12 |
| KMPGU116_19_Rhiniinae | 319 | 110 | 110 | 76 | 75 | 66 | 63 |
| KMPHA111_18_Rhiniinae | 320 | 70 | 70 | 49 | 48 | 41 | 12 |
| KMPHA131_18_Rhiniinae | 223 | 70 | 70 | 49 | 48 | 41 | 12 |
| KMPHB583_18_Rhiniinae | 321 | 70 | 70 | 49 | 48 | 41 | 12 |
| KMPHC268_18_Rhiniinae | 322 | 107 | 107 | 51 | 50 | 43 | 42 |
| KMPHE323_19_Rhiniinae | 226 | 70 | 70 | 49 | 48 | 41 | 12 |
| KMPHF300_19_Rhiniinae | 323 | 107 | 107 | 51 | 50 | 43 | 42 |
| KMPHG024_19_Rhiniinae | 214 | 70 | 70 | 49 | 48 | 41 | 12 |
| KMPHG026_19_Rhiniinae | 324 | 107 | 107 | 51 | 50 | 43 | 42 |
| KMPHH035_19_Rhiniinae | 223 | 70 | 70 | 49 | 48 | 41 | 12 |
| KMPHI410_19_Rhyncomya_soyauxi | 250 | 71 | 71 | 50 | 49 | 42 | 41 |
| KMPHI411_19_Rhiniinae | 214 | 70 | 70 | 49 | 48 | 41 | 12 |
| KMPHJ075_19_Rhiniinae | 278 | 107 | 107 | 51 | 50 | 43 | 42 |
| KMPHJ076_19_Rhiniinae | 237 | 70 | 70 | 49 | 48 | 41 | 12 |
| KMPHJ078_19_Rhiniinae | 325 | 107 | 107 | 51 | 50 | 43 | 42 |
| KMPHJ080_19_Rhyncomya_soyauxi | 326 | 71 | 71 | 50 | 49 | 42 | 41 |
| KMPHL515_19_Rhiniinae | 312 | 107 | 107 | 51 | 50 | 43 | 42 |
| KMPHL516_19_Rhiniinae | 223 | 70 | 70 | 49 | 48 | 41 | 12 |
| KMPHL517_19_Rhiniinae | 236 | 69 | 69 | 48 | 47 | 40 | 40 |
| KMPHM050_19_Rhiniinae | 327 | 107 | 107 | 51 | 50 | 43 | 42 |
| KMPHM353_19_Rhiniinae | 226 | 70 | 70 | 49 | 48 | 41 | 12 |
| KMPHM360_19_Rhiniinae | 328 | 107 | 107 | 51 | 50 | 43 | 42 |
| KMPHN1942_19_Rhiniinae | 226 | 70 | 70 | 49 | 48 | 41 | 12 |
| KMPHN1943_19_Rhiniinae | 329 | 70 | 70 | 49 | 48 | 41 | 12 |
| KMPHN1951_19_Rhiniinae | 330 | 107 | 107 | 51 | 50 | 43 | 42 |
| KMPHN1955_19_Rhyncomya_soyauxi | 221 | 71 | 71 | 50 | 49 | 42 | 41 |
| KMPHN1959_19_Rhiniinae | 331 | 70 | 70 | 49 | 48 | 41 | 12 |
| KMPHN1960_19_Rhiniinae | 332 | 70 | 70 | 49 | 48 | 41 | 12 |
| KMPHN1965_19_Rhiniinae | 223 | 70 | 70 | 49 | 48 | 41 | 12 |
| KMPHN1967_19_Rhiniinae | 214 | 70 | 70 | 49 | 48 | 41 | 12 |
| KMPHN1971_19_Rhiniinae | 333 | 70 | 70 | 49 | 48 | 41 | 12 |
| KMPHN1973_19_Rhiniinae | 334 | 70 | 70 | 49 | 48 | 41 | 12 |
| KMPHN1974_19_Rhiniinae | 335 | 70 | 70 | 49 | 48 | 41 | 12 |
| KMPHN1978_19_Rhiniinae | 336 | 70 | 70 | 49 | 48 | 41 | 12 |
| KMPHN1979_19_Rhiniinae | 337 | 107 | 107 | 51 | 50 | 43 | 42 |
| KMPHO042_19_Rhiniinae | 338 | 70 | 70 | 49 | 48 | 41 | 12 |
| KMPHO043_19_Rhiniinae | 339 | 70 | 70 | 49 | 48 | 41 | 12 |
| KMPHO045_19_Rhiniinae | 92 | 2 | 2 | 2 | 2 | 2 | 2 |
| KMPHO046_19_Rhiniinae | 340 | 70 | 70 | 49 | 48 | 41 | 12 |
| KMPHO047_19_Rhiniinae | 341 | 107 | 107 | 51 | 50 | 43 | 42 |
| KMPHO057_19_Rhiniinae | 342 | 107 | 107 | 51 | 50 | 43 | 42 |
| KMPHP045_19_Rhiniinae | 309 | 70 | 70 | 49 | 48 | 41 | 12 |
| KMPHQ1269_19_Rhiniinae | 343 | 70 | 70 | 49 | 48 | 41 | 12 |
| KMPHQ1276_19_Rhiniinae | 214 | 70 | 70 | 49 | 48 | 41 | 12 |
| KMPHR034_19_Rhiniinae | 344 | 70 | 70 | 49 | 48 | 41 | 12 |
| KMPHS132_19_Rhyncomya_soyauxi | 345 | 71 | 71 | 50 | 49 | 42 | 41 |
| KMPHS134_19_Rhiniinae | 346 | 70 | 70 | 49 | 48 | 41 | 12 |
| KMPHS138_19_Rhiniinae | 285 | 70 | 70 | 49 | 48 | 41 | 12 |
| KMPHS140_19_Rhiniinae | 135 | 72 | 72 | 49 | 48 | 41 | 12 |
| KMPHS142_19_Rhiniinae | 223 | 70 | 70 | 49 | 48 | 41 | 12 |
| KMPHS143_19_Rhiniinae | 347 | 70 | 70 | 49 | 48 | 41 | 12 |
| KMPHS149_19_Rhiniinae | 348 | 107 | 107 | 51 | 50 | 43 | 42 |
| KMPHX021_19_Rhiniinae | 135 | 72 | 72 | 49 | 48 | 41 | 12 |
| KMPHY036_19_Rhiniinae | 223 | 70 | 70 | 49 | 48 | 41 | 12 |
| KMPHY042_19_Rhiniinae | 312 | 107 | 107 | 51 | 50 | 43 | 42 |
| KMPIA240_18_Rhiniinae | 223 | 70 | 70 | 49 | 48 | 41 | 12 |
| KMPIA255_18_Rhiniinae | 349 | 110 | 110 | 76 | 75 | 66 | 63 |
| KMPIA262_18_Rhiniinae | 272 | 107 | 107 | 51 | 50 | 43 | 42 |
| KMPIA267_18_Rhyncomya_soyauxi | 350 | 71 | 71 | 50 | 49 | 42 | 41 |
| KMPIC004_18_Rhiniinae | 351 | 107 | 107 | 51 | 50 | 43 | 42 |
| KMPIC013_18_Rhiniinae | 351 | 107 | 107 | 51 | 50 | 43 | 42 |
| KMPII044_19_Rhiniinae | 223 | 70 | 70 | 49 | 48 | 41 | 12 |
| KMPII045_19_Rhiniinae | 352 | 70 | 70 | 49 | 48 | 41 | 12 |
| KMPII089_19_Rhyncomya_soyauxi | 221 | 71 | 71 | 50 | 49 | 42 | 41 |
| KMPII095_19_Rhiniinae | 353 | 107 | 107 | 51 | 50 | 43 | 42 |
| KMPIJ181_19_Rhiniinae | 226 | 70 | 70 | 49 | 48 | 41 | 12 |
| KMPIJ182_19_Rhiniinae | 329 | 70 | 70 | 49 | 48 | 41 | 12 |
| KMPIK064_19_Rhiniinae | 354 | 70 | 70 | 49 | 48 | 41 | 12 |
| KMPIK142_19_Rhiniinae | 355 | 107 | 107 | 51 | 50 | 43 | 42 |
| KMPIL231_19_Rhiniinae | 236 | 69 | 69 | 48 | 47 | 40 | 40 |
| KMPIL234_19_Rhiniinae | 356 | 69 | 69 | 48 | 47 | 40 | 40 |
| KMPIL236_19_Rhiniinae | 223 | 70 | 70 | 49 | 48 | 41 | 12 |
| KMPIL238_19_Rhiniinae | 357 | 70 | 70 | 49 | 48 | 41 | 12 |
| KMPIL239_19_Rhiniinae | 358 | 70 | 70 | 49 | 48 | 41 | 12 |
| KMPIL240_19_Rhiniinae | 135 | 72 | 72 | 49 | 48 | 41 | 12 |
| KMPIL300_19_Rhiniinae | 359 | 70 | 70 | 49 | 48 | 41 | 12 |
| KMPIM021_19_Rhiniinae | 214 | 70 | 70 | 49 | 48 | 41 | 12 |
| KMPIM024_19_Rhiniinae | 226 | 70 | 70 | 49 | 48 | 41 | 12 |
| KMPIM026_19_Rhiniinae | 214 | 70 | 70 | 49 | 48 | 41 | 12 |
| KMPIM028_19_Rhiniinae | 323 | 107 | 107 | 51 | 50 | 43 | 42 |
| KMPIN001_19_Rhiniinae | 360 | 70 | 70 | 49 | 48 | 41 | 12 |
| KMPIN003_19_Rhiniinae | 361 | 107 | 107 | 51 | 50 | 43 | 42 |
| KMPIN004_19_Rhiniinae | 362 | 70 | 70 | 49 | 48 | 41 | 12 |
| KMPIN005_19_Rhiniinae | 363 | 107 | 107 | 51 | 50 | 43 | 42 |
| KMPIN006_19_Rhiniinae | 223 | 70 | 70 | 49 | 48 | 41 | 12 |
| KMPIN007_19_Rhiniinae | 364 | 70 | 70 | 49 | 48 | 41 | 12 |
| KMPIO147_19_Rhiniinae | 223 | 70 | 70 | 49 | 48 | 41 | 12 |
| KMPIO149_19_Rhiniinae | 223 | 70 | 70 | 49 | 48 | 41 | 12 |
| KMPIO150_19_Rhiniinae | 223 | 70 | 70 | 49 | 48 | 41 | 12 |
| KMPIO151_19_Rhiniinae | 226 | 70 | 70 | 49 | 48 | 41 | 12 |
| KMPIO152_19_Rhiniinae | 226 | 70 | 70 | 49 | 48 | 41 | 12 |
| KMPIO153_19_Rhiniinae | 365 | 70 | 70 | 49 | 48 | 41 | 12 |
| KMPIO154_19_Rhiniinae | 237 | 70 | 70 | 49 | 48 | 41 | 12 |
| KMPIO155_19_Rhiniinae | 149 | 70 | 70 | 49 | 48 | 41 | 12 |
| KMPIO156_19_Rhiniinae | 223 | 70 | 70 | 49 | 48 | 41 | 12 |
| KMPIO157_19_Rhiniinae | 226 | 70 | 70 | 49 | 48 | 41 | 12 |
| KMPIO158_19_Rhiniinae | 214 | 70 | 70 | 49 | 48 | 41 | 12 |
| KMPIO159_19_Rhiniinae | 284 | 70 | 70 | 49 | 48 | 41 | 12 |
| KMPIO160_19_Rhiniinae | 290 | 70 | 70 | 49 | 48 | 41 | 12 |
| KMPIO162_19_Rhyncomya_soyauxi | 366 | 71 | 71 | 50 | 49 | 42 | 41 |
| KMPIO163_19_Rhiniinae | 223 | 70 | 70 | 49 | 48 | 41 | 12 |
| KMPIO164_19_Rhiniinae | 223 | 70 | 70 | 49 | 48 | 41 | 12 |
| KMPIO165_19_Rhiniinae | 223 | 70 | 70 | 49 | 48 | 41 | 12 |
| KMPIO166_19_Rhiniinae | 359 | 70 | 70 | 49 | 48 | 41 | 12 |
| KMPIO167_19_Rhiniinae | 237 | 70 | 70 | 49 | 48 | 41 | 12 |
| KMPIO168_19_Rhiniinae | 367 | 107 | 107 | 51 | 50 | 43 | 42 |
| KMPIO170_19_Rhiniinae | 368 | 70 | 70 | 49 | 48 | 41 | 12 |
| KMPIO171_19_Rhiniinae | 223 | 70 | 70 | 49 | 48 | 41 | 12 |
| KMPIO172_19_Rhiniinae | 237 | 70 | 70 | 49 | 48 | 41 | 12 |
| KMPIO173_19_Rhiniinae | 369 | 107 | 107 | 51 | 50 | 43 | 42 |
| KMPIO174_19_Rhiniinae | 214 | 70 | 70 | 49 | 48 | 41 | 12 |
| KMPIO176_19_Rhiniinae | 214 | 70 | 70 | 49 | 48 | 41 | 12 |
| KMPIO177_19_Rhiniinae | 370 | 70 | 70 | 49 | 48 | 41 | 12 |
| KMPIO178_19_Rhiniinae | 371 | 70 | 70 | 49 | 48 | 41 | 12 |
| KMPIO179_19_Rhiniinae | 238 | 70 | 70 | 49 | 48 | 41 | 12 |
| KMPIO180_19_Rhiniinae | 214 | 70 | 70 | 49 | 48 | 41 | 12 |
| KMPIO181_19_Rhiniinae | 346 | 70 | 70 | 49 | 48 | 41 | 12 |
| KMPIO182_19_Rhiniinae | 223 | 70 | 70 | 49 | 48 | 41 | 12 |
| KMPIO183_19_Rhiniinae | 289 | 70 | 70 | 49 | 48 | 41 | 12 |
| KMPIO186_19_Rhiniinae | 372 | 112 | 112 | 51 | 50 | 43 | 42 |
| KMPIP216_19_Rhiniinae | 373 | 70 | 70 | 49 | 48 | 41 | 12 |
| KMPIP217_19_Rhiniinae | 374 | 70 | 70 | 49 | 48 | 41 | 12 |
| KMPIP222_19_Rhiniinae | 375 | 72 | 72 | 49 | 48 | 41 | 12 |
| KMPIP225_19_Rhiniinae | 376 | 70 | 70 | 49 | 48 | 41 | 12 |
| KMPIP226_19_Rhiniinae | 223 | 70 | 70 | 49 | 48 | 41 | 12 |
| KMPIP228_19_Rhiniinae | 223 | 70 | 70 | 49 | 48 | 41 | 12 |
| KMPIP230_19_Rhiniinae | 377 | 70 | 70 | 49 | 48 | 41 | 12 |
| KMPIP232_19_Rhiniinae | 223 | 70 | 70 | 49 | 48 | 41 | 12 |
| KMPIP235_19_Rhiniinae | 223 | 70 | 70 | 49 | 48 | 41 | 12 |
| KMPIP236_19_Rhiniinae | 378 | 70 | 70 | 49 | 48 | 41 | 12 |
| KMPIP237_19_Rhiniinae | 135 | 72 | 72 | 49 | 48 | 41 | 12 |
| KMPIP240_19_Rhiniinae | 316 | 70 | 70 | 49 | 48 | 41 | 12 |
| KMPIP247_19_Rhiniinae | 379 | 70 | 70 | 49 | 48 | 41 | 12 |
| KMPIP248_19_Rhiniinae | 380 | 113 | 113 | 49 | 48 | 41 | 12 |
| KMPIP249_19_Rhiniinae | 381 | 70 | 70 | 49 | 48 | 41 | 12 |
| KMPIP251_19_Rhiniinae | 382 | 70 | 70 | 49 | 48 | 41 | 12 |
| KMPIP252_19_Rhiniinae | 383 | 70 | 70 | 49 | 48 | 41 | 12 |
| KMPIP254_19_Rhiniinae | 214 | 70 | 70 | 49 | 48 | 41 | 12 |
| KMPIP256_19_Rhiniinae | 384 | 107 | 107 | 51 | 50 | 43 | 42 |
| KMPIP260_19_Rhyncomya_soyauxi | 385 | 71 | 71 | 50 | 49 | 42 | 41 |
| KMPIP262_19_Rhiniinae | 223 | 70 | 70 | 49 | 48 | 41 | 12 |
| KMPIP273_19_Rhiniinae | 386 | 70 | 70 | 49 | 48 | 41 | 12 |
| KMPIP278_19_Rhiniinae | 214 | 70 | 70 | 49 | 48 | 41 | 12 |
| KMPIP287_19_Rhiniinae | 387 | 70 | 70 | 49 | 48 | 41 | 12 |
| KMPIP288_19_Rhiniinae | 388 | 114 | 114 | 49 | 48 | 41 | 12 |
| KMPIP289_19_Rhiniinae | 389 | 70 | 70 | 49 | 48 | 41 | 12 |
| KMPIP290_19_Rhiniinae | 223 | 70 | 70 | 49 | 48 | 41 | 12 |
| KMPIP291_19_Rhiniinae | 390 | 70 | 70 | 49 | 48 | 41 | 12 |
| KMPIP292_19_Rhiniinae | 223 | 70 | 70 | 49 | 48 | 41 | 12 |
| KMPIP293_19_Rhiniinae | 391 | 70 | 70 | 49 | 48 | 41 | 12 |
| KMPIP296_19_Rhiniinae | 238 | 70 | 70 | 49 | 48 | 41 | 12 |
| KMPIP297_19_Rhiniinae | 392 | 107 | 107 | 51 | 50 | 43 | 42 |
| KMPIP299_19_Rhiniinae | 393 | 70 | 70 | 49 | 48 | 41 | 12 |
| KMPIQ020_19_Rhiniinae | 226 | 70 | 70 | 49 | 48 | 41 | 12 |
| KMPIQ021_19_Rhiniinae | 214 | 70 | 70 | 49 | 48 | 41 | 12 |
| KMPIQ022_19_Rhiniinae | 394 | 72 | 72 | 49 | 48 | 41 | 12 |
| KMPIQ023_19_Rhiniinae | 223 | 70 | 70 | 49 | 48 | 41 | 12 |
| KMPIQ024_19_Rhiniinae | 395 | 107 | 107 | 51 | 50 | 43 | 42 |
| KMPIQ025_19_Rhiniinae | 223 | 70 | 70 | 49 | 48 | 41 | 12 |
| KMPIQ030_19_Rhiniinae | 396 | 70 | 70 | 49 | 48 | 41 | 12 |
| KMPIQ037_19_Rhiniinae | 92 | 2 | 2 | 2 | 2 | 2 | 2 |
| KMPIR011_19_Rhiniinae | 226 | 70 | 70 | 49 | 48 | 41 | 12 |
| KMPIS671_19_Rhiniinae | 223 | 70 | 70 | 49 | 48 | 41 | 12 |
| KMPIS673_19_Rhiniinae | 346 | 70 | 70 | 49 | 48 | 41 | 12 |
| KMPIS675_19_Rhiniinae | 397 | 107 | 107 | 51 | 50 | 43 | 42 |
| KMPIS678_19_Rhiniinae | 398 | 107 | 107 | 51 | 50 | 43 | 42 |
| KMPIS679_19_Rhiniinae | 399 | 107 | 107 | 51 | 50 | 43 | 42 |
| KMPIS680_19_Rhiniinae | 214 | 70 | 70 | 49 | 48 | 41 | 12 |
| KMPIS684_19_Rhiniinae | 400 | 107 | 107 | 51 | 50 | 43 | 42 |
| KMPIS686_19_Rhiniinae | 327 | 107 | 107 | 51 | 50 | 43 | 42 |
| KMPIT131_19_Rhiniinae | 226 | 70 | 70 | 49 | 48 | 41 | 12 |
| KMPIT136_19_Rhiniinae | 223 | 70 | 70 | 49 | 48 | 41 | 12 |
| KMPIT138_19_Rhiniinae | 401 | 70 | 70 | 49 | 48 | 41 | 12 |
| KMPIT142_19_Rhiniinae | 402 | 70 | 70 | 49 | 48 | 41 | 12 |
| KMPIT144_19_Rhiniinae | 403 | 70 | 70 | 49 | 48 | 41 | 12 |
| KMPIT148_19_Rhiniinae | 295 | 110 | 110 | 76 | 75 | 66 | 63 |
| KMPIT154_19_Rhiniinae | 319 | 110 | 110 | 76 | 75 | 66 | 63 |
| KMPIT156_19_Rhiniinae | 319 | 110 | 110 | 76 | 75 | 66 | 63 |
| KMPIT161_19_Rhiniinae | 404 | 107 | 107 | 51 | 50 | 43 | 42 |
| KMPIT428_19_Rhiniinae | 405 | 110 | 110 | 76 | 75 | 66 | 63 |
| KMPIU181_19_Rhiniinae | 406 | 110 | 110 | 76 | 75 | 66 | 63 |
| KMPIU594_19_Rhiniinae | 365 | 70 | 70 | 49 | 48 | 41 | 12 |
| KMPIU596_19_Rhiniinae | 407 | 115 | 115 | 78 | 77 | 68 | 65 |
| KMPIU597_19_Rhiniinae | 223 | 70 | 70 | 49 | 48 | 41 | 12 |
| KMPIU603_19_Rhiniinae | 405 | 110 | 110 | 76 | 75 | 66 | 63 |
| KMPIU610_19_Rhiniinae | 284 | 70 | 70 | 49 | 48 | 41 | 12 |
| KMPIU611_19_Rhiniinae | 284 | 70 | 70 | 49 | 48 | 41 | 12 |
| KMPIU614_19_Rhiniinae | 319 | 110 | 110 | 76 | 75 | 66 | 63 |
| KMPIU620_19_Rhiniinae | 214 | 70 | 70 | 49 | 48 | 41 | 12 |
| KMPIU626_19_Rhiniinae | 408 | 110 | 110 | 76 | 75 | 66 | 63 |
| KMPIU629_19_Rhiniinae | 409 | 110 | 110 | 76 | 75 | 66 | 63 |
| KMPIV022_19_Rhiniinae | 223 | 70 | 70 | 49 | 48 | 41 | 12 |
| KMPIV025_19_Rhiniinae | 319 | 110 | 110 | 76 | 75 | 66 | 63 |
| KMPIV029_19_Rhiniinae | 410 | 110 | 110 | 76 | 75 | 66 | 63 |
| KMPIV030_19_Rhiniinae | 411 | 107 | 107 | 51 | 50 | 43 | 42 |
| KMPJB015_18_Rhiniinae | 320 | 70 | 70 | 49 | 48 | 41 | 12 |
| KMPJF263_19_Rhiniinae | 289 | 70 | 70 | 49 | 48 | 41 | 12 |
| KMPJF267_19_Rhiniinae | 405 | 110 | 110 | 76 | 75 | 66 | 63 |
| KMPJI050_19_Rhiniinae | 214 | 70 | 70 | 49 | 48 | 41 | 12 |
| KMPJI051_19_Rhiniinae | 238 | 70 | 70 | 49 | 48 | 41 | 12 |
| KMPJI052_19_Rhiniinae | 258 | 107 | 107 | 51 | 50 | 43 | 42 |
| KMPJI058_19_Rhiniinae | 214 | 70 | 70 | 49 | 48 | 41 | 12 |
| KMPJI063_19_Rhiniinae | 412 | 107 | 107 | 51 | 50 | 43 | 42 |
| KMPJI064_19_Rhiniinae | 413 | 70 | 70 | 49 | 48 | 41 | 12 |
| KMPJI067_19_Rhiniinae | 223 | 70 | 70 | 49 | 48 | 41 | 12 |
| KMPJJ480_19_Rhiniinae | 272 | 107 | 107 | 51 | 50 | 43 | 42 |
| KMPJJ692_19_Rhiniinae | 252 | 70 | 70 | 49 | 48 | 41 | 12 |
| KMPJK103_19_Rhiniinae | 414 | 116 | 116 | 51 | 50 | 43 | 42 |
| KMPJK155_19_Rhiniinae | 226 | 70 | 70 | 49 | 48 | 41 | 12 |
| KMPJK158_19_Rhiniinae | 415 | 70 | 70 | 49 | 48 | 41 | 12 |
| KMPJK160_19_Rhyncomya_soyauxi | 416 | 71 | 71 | 50 | 49 | 42 | 41 |
| KMPJK162_19_Rhiniinae | 223 | 70 | 70 | 49 | 48 | 41 | 12 |
| KMPJK227_19_Rhiniinae | 417 | 107 | 107 | 51 | 50 | 43 | 42 |
| KMPJK229_19_Rhiniinae | 418 | 107 | 107 | 51 | 50 | 43 | 42 |
| KMPJL029_19_Rhiniinae | 237 | 70 | 70 | 49 | 48 | 41 | 12 |
| KMPJL030_19_Rhiniinae | 419 | 117 | 117 | 79 | 78 | 69 | 66 |
| KMPJL040_19_Rhiniinae | 223 | 70 | 70 | 49 | 48 | 41 | 12 |
| KMPJL045_19_Rhyncomya_soyauxi | 224 | 71 | 71 | 50 | 49 | 42 | 41 |
| KMPJL584_19_Rhiniinae | 420 | 107 | 107 | 51 | 50 | 43 | 42 |
| KMPJM189_19_Rhiniinae | 236 | 69 | 69 | 48 | 47 | 40 | 40 |
| KMPJM191_19_Rhiniinae | 223 | 70 | 70 | 49 | 48 | 41 | 12 |
| KMPJM193_19_Rhiniinae | 421 | 107 | 107 | 51 | 50 | 43 | 42 |
| KMPJM194_19_Rhiniinae | 223 | 70 | 70 | 49 | 48 | 41 | 12 |
| KMPJN461_19_Rhiniinae | 223 | 70 | 70 | 49 | 48 | 41 | 12 |
| KMPJN525_19_Rhiniinae | 422 | 107 | 107 | 51 | 50 | 43 | 42 |
| KMPJO1838_19_Rhiniinae | 226 | 70 | 70 | 49 | 48 | 41 | 12 |
| KMPJP114_19_Rhiniinae | 423 | 70 | 70 | 49 | 48 | 41 | 12 |
| KMPJP2434_19_Rhiniinae | 226 | 70 | 70 | 49 | 48 | 41 | 12 |
| KMPJP2435_19_Rhiniinae | 424 | 70 | 70 | 49 | 48 | 41 | 12 |
| KMPJP2438_19_Rhiniinae | 425 | 70 | 70 | 49 | 48 | 41 | 12 |
| KMPJQ046_19_Rhiniinae | 226 | 70 | 70 | 49 | 48 | 41 | 12 |
| KMPJQ074_19_Rhiniinae | 3 | 2 | 2 | 2 | 2 | 2 | 2 |
| KMPJR294_19_Rhiniinae | 227 | 107 | 107 | 51 | 50 | 43 | 42 |
| KMPJT030_19_Rhiniinae | 319 | 110 | 110 | 76 | 75 | 66 | 63 |
| KMPJT032_19_Rhiniinae | 223 | 70 | 70 | 49 | 48 | 41 | 12 |
| KMPJT034_19_Rhiniinae | 295 | 110 | 110 | 76 | 75 | 66 | 63 |
| KMPJU057_19_Rhiniinae | 426 | 110 | 110 | 76 | 75 | 66 | 63 |
| KMPJV085_19_Rhiniinae | 92 | 2 | 2 | 2 | 2 | 2 | 2 |
| KMPJV093_19_Rhiniinae | 319 | 110 | 110 | 76 | 75 | 66 | 63 |
| KMPJV094_19_Rhiniinae | 223 | 70 | 70 | 49 | 48 | 41 | 12 |
| KMPJV096_19_Rhiniinae | 427 | 107 | 107 | 51 | 50 | 43 | 42 |
| KMPJV137_19_Rhiniinae | 240 | 107 | 107 | 51 | 50 | 43 | 42 |
| KMPJV193_19_Rhiniinae | 428 | 110 | 110 | 76 | 75 | 66 | 63 |
| KMPJV296_19_Rhiniinae | 428 | 110 | 110 | 76 | 75 | 66 | 63 |
| KMPJV556_19_Rhiniinae | 295 | 110 | 110 | 76 | 75 | 66 | 63 |
| KMPJV590_19_Rhiniinae | 429 | 107 | 107 | 51 | 50 | 43 | 42 |
| KMPKB001_18_Rhiniinae | 430 | 7 | 7 | 6 | 6 | 1 | 1 |
| KMPKB064_18_Rhiniinae | 430 | 7 | 7 | 6 | 6 | 1 | 1 |
| KMPKI026_19_Rhiniinae | 431 | 107 | 107 | 51 | 50 | 43 | 42 |
| KMPKJ001_19_Rhiniinae | 431 | 107 | 107 | 51 | 50 | 43 | 42 |
| KMPKK055_19_Rhiniinae | 223 | 70 | 70 | 49 | 48 | 41 | 12 |
| KMPKK056_19_Rhiniinae | 279 | 69 | 69 | 48 | 47 | 40 | 40 |
| KMPKK057_19_Rhiniinae | 223 | 70 | 70 | 49 | 48 | 41 | 12 |
| KMPKK058_19_Rhiniinae | 226 | 70 | 70 | 49 | 48 | 41 | 12 |
| KMPKK059_19_Rhiniinae | 236 | 69 | 69 | 48 | 47 | 40 | 40 |
| KMPKK061_19_Rhiniinae | 427 | 107 | 107 | 51 | 50 | 43 | 42 |
| KMPKK062_19_Rhiniinae | 237 | 70 | 70 | 49 | 48 | 41 | 12 |
| KMPKK063_19_Rhiniinae | 432 | 107 | 107 | 51 | 50 | 43 | 42 |
| KMPKK064_19_Rhiniinae | 237 | 70 | 70 | 49 | 48 | 41 | 12 |
| KMPKK065_19_Rhiniinae | 236 | 69 | 69 | 48 | 47 | 40 | 40 |
| KMPKK066_19_Rhiniinae | 433 | 107 | 107 | 51 | 50 | 43 | 42 |
| KMPKL079_19_Rhiniinae | 272 | 107 | 107 | 51 | 50 | 43 | 42 |
| KMPKL080_19_Rhiniinae | 223 | 70 | 70 | 49 | 48 | 41 | 12 |
| KMPKL081_19_Rhiniinae | 223 | 70 | 70 | 49 | 48 | 41 | 12 |
| KMPKL082_19_Rhiniinae | 434 | 70 | 70 | 49 | 48 | 41 | 12 |
| KMPKL083_19_Rhiniinae | 226 | 70 | 70 | 49 | 48 | 41 | 12 |
| KMPKL084_19_Rhiniinae | 435 | 107 | 107 | 51 | 50 | 43 | 42 |
| KMPKL085_19_Rhiniinae | 436 | 70 | 70 | 49 | 48 | 41 | 12 |
| KMPKL086_19_Rhiniinae | 236 | 69 | 69 | 48 | 47 | 40 | 40 |
| KMPKL087_19_Rhiniinae | 437 | 107 | 107 | 51 | 50 | 43 | 42 |
| KMPKM029_19_Rhiniinae | 223 | 70 | 70 | 49 | 48 | 41 | 12 |
| KMPKM030_19_Rhiniinae | 236 | 69 | 69 | 48 | 47 | 40 | 40 |
| KMPKM033_19_Rhiniinae | 438 | 107 | 107 | 51 | 50 | 43 | 42 |
| KMPKM035_19_Rhiniinae | 309 | 70 | 70 | 49 | 48 | 41 | 12 |
| KMPKN134_19_Rhiniinae | 439 | 70 | 70 | 49 | 48 | 41 | 12 |
| KMPKN137_19_Rhiniinae | 226 | 70 | 70 | 49 | 48 | 41 | 12 |
| KMPKN142_19_Rhiniinae | 440 | 70 | 70 | 49 | 48 | 41 | 12 |
| KMPKN147_19_Rhiniinae | 272 | 107 | 107 | 51 | 50 | 43 | 42 |
| KMPKN150_19_Rhiniinae | 237 | 70 | 70 | 49 | 48 | 41 | 12 |
| KMPKO189_19_Rhiniinae | 223 | 70 | 70 | 49 | 48 | 41 | 12 |
| KMPKP665_19_Rhiniinae | 441 | 107 | 107 | 51 | 50 | 43 | 42 |
| KMPLC105_18_Rhiniinae | 442 | 107 | 107 | 51 | 50 | 43 | 42 |
| KMPLC317_18_Rhiniinae | 443 | 107 | 107 | 51 | 50 | 43 | 42 |
| KMPLJ134_19_Rhinia | 10 | 4 | 4 | 4 | 4 | 2 | 2 |
| KMPLJ136_19_Rhiniinae | 444 | 107 | 107 | 51 | 50 | 43 | 42 |
| KMPLK071_19_Rhiniinae | 445 | 107 | 107 | 51 | 50 | 43 | 42 |
| KMPLQ1635_19_Rhiniinae | 446 | 70 | 70 | 49 | 48 | 41 | 12 |
| KMPLX033_19_Rhiniinae | 316 | 70 | 70 | 49 | 48 | 41 | 12 |
| KMPMA102_18_Rhiniinae | 447 | 107 | 107 | 51 | 50 | 43 | 42 |
| KMPMA115_18_Rhiniinae | 448 | 107 | 107 | 51 | 50 | 43 | 42 |
| KMPMA184_18_Rhiniinae | 226 | 70 | 70 | 49 | 48 | 41 | 12 |
| KMPMA186_18_Rhiniinae | 223 | 70 | 70 | 49 | 48 | 41 | 12 |
| KMPMA187_18_Rhiniinae | 223 | 70 | 70 | 49 | 48 | 41 | 12 |
| KMPMA196_18_Rhiniinae | 223 | 70 | 70 | 49 | 48 | 41 | 12 |
| KMPMA198_18_Rhiniinae | 449 | 69 | 69 | 48 | 47 | 40 | 40 |
| KMPMA223_18_Rhiniinae | 450 | 70 | 70 | 49 | 48 | 41 | 12 |
| KMPMB382_18_Rhiniinae | 214 | 70 | 70 | 49 | 48 | 41 | 12 |
| KMPMC036_18_Rhiniinae | 312 | 107 | 107 | 51 | 50 | 43 | 42 |
| KMPMC251_18_Rhiniinae | 451 | 107 | 107 | 51 | 50 | 43 | 42 |
| KMPMC259_18_Rhiniinae | 226 | 70 | 70 | 49 | 48 | 41 | 12 |
| KMPMD012_18_Rhiniinae | 309 | 70 | 70 | 49 | 48 | 41 | 12 |
| KMPMD071_18_Rhiniinae | 452 | 107 | 107 | 51 | 50 | 43 | 42 |
| KMPMD159_18_Rhiniinae | 453 | 107 | 107 | 51 | 50 | 43 | 42 |
| KMPMF080_19_Rhiniinae | 223 | 70 | 70 | 49 | 48 | 41 | 12 |
| KMPMF081_19_Rhiniinae | 226 | 70 | 70 | 49 | 48 | 41 | 12 |
| KMPMH070_19_Rhiniinae | 223 | 70 | 70 | 49 | 48 | 41 | 12 |
| KMPMH072_19_Rhiniinae | 237 | 70 | 70 | 49 | 48 | 41 | 12 |
| KMPML019_19_Rhiniinae | 226 | 70 | 70 | 49 | 48 | 41 | 12 |
| KMPML024_19_Rhiniinae | 431 | 107 | 107 | 51 | 50 | 43 | 42 |
| KMPMN584_19_Rhiniinae | 272 | 107 | 107 | 51 | 50 | 43 | 42 |
| KMPMO095_19_Rhiniinae | 226 | 70 | 70 | 49 | 48 | 41 | 12 |
| KMPMO098_19_Rhiniinae | 309 | 70 | 70 | 49 | 48 | 41 | 12 |
| KMPMO119_19_Rhiniinae | 454 | 107 | 107 | 51 | 50 | 43 | 42 |
| KMPMO129_19_Rhiniinae | 455 | 107 | 107 | 51 | 50 | 43 | 42 |
| KMPMO134_19_Rhiniinae | 237 | 70 | 70 | 49 | 48 | 41 | 12 |
| KMPMO135_19_Rhiniinae | 456 | 107 | 107 | 51 | 50 | 43 | 42 |
| KMPMO227_19_Rhiniinae | 272 | 107 | 107 | 51 | 50 | 43 | 42 |
| KMPMP121_19_Rhiniinae | 223 | 70 | 70 | 49 | 48 | 41 | 12 |
| KMPMP123_19_Rhiniinae | 457 | 70 | 70 | 49 | 48 | 41 | 12 |
| KMPMP125_19_Rhiniinae | 226 | 70 | 70 | 49 | 48 | 41 | 12 |
| KMPMQ1717_19_Rhiniinae | 240 | 107 | 107 | 51 | 50 | 43 | 42 |
| KMPMQ320_19_Rhiniinae | 214 | 70 | 70 | 49 | 48 | 41 | 12 |
| KMPMQ326_19_Rhiniinae | 458 | 107 | 107 | 51 | 50 | 43 | 42 |
| KMPMQ332_19_Rhiniinae | 316 | 70 | 70 | 49 | 48 | 41 | 12 |
| KMPMQ336_19_Rhiniinae | 223 | 70 | 70 | 49 | 48 | 41 | 12 |
| KMPMQ338_19_Rhiniinae | 223 | 70 | 70 | 49 | 48 | 41 | 12 |
| KMPMQ341_19_Rhiniinae | 237 | 70 | 70 | 49 | 48 | 41 | 12 |
| KMPMQ343_19_Stegosoma_vinculatum | 101 | 10 | 10 | 8 | 8 | 5 | 5 |
| KMPMQ347_19_Rhyncomya_soyauxi | 459 | 71 | 71 | 50 | 49 | 42 | 41 |
| KMPMQ348_19_Rhiniinae | 223 | 70 | 70 | 49 | 48 | 41 | 12 |
| KMPMQ350_19_Rhiniinae | 223 | 70 | 70 | 49 | 48 | 41 | 12 |
| KMPMQ358_19_Rhiniinae | 214 | 70 | 70 | 49 | 48 | 41 | 12 |
| KMPMQ360_19_Rhiniinae | 214 | 70 | 70 | 49 | 48 | 41 | 12 |
| KMPMQ363_19_Rhiniinae | 460 | 70 | 70 | 49 | 48 | 41 | 12 |
| KMPMQ371_19_Rhiniinae | 461 | 70 | 70 | 49 | 48 | 41 | 12 |
| KMPMQ372_19_Rhiniinae | 462 | 107 | 107 | 51 | 50 | 43 | 42 |
| KMPMQ381_19_Rhiniinae | 223 | 70 | 70 | 49 | 48 | 41 | 12 |
| KMPMQ385_19_Rhiniinae | 223 | 70 | 70 | 49 | 48 | 41 | 12 |
| KMPMQ388_19_Rhiniinae | 240 | 107 | 107 | 51 | 50 | 43 | 42 |
| KMPMQ390_19_Rhyncomya_soyauxi | 225 | 71 | 71 | 50 | 49 | 42 | 41 |
| KMPMQ394_19_Rhiniinae | 223 | 70 | 70 | 49 | 48 | 41 | 12 |
| KMPMQ397_19_Rhiniinae | 463 | 70 | 70 | 49 | 48 | 41 | 12 |
| KMPMQ398_19_Rhiniinae | 223 | 70 | 70 | 49 | 48 | 41 | 12 |
| KMPMQ406_19_Rhiniinae | 464 | 70 | 70 | 49 | 48 | 41 | 12 |
| KMPMQ411_19_Rhiniinae | 465 | 70 | 70 | 49 | 48 | 41 | 12 |
| KMPMQ412_19_Rhiniinae | 223 | 70 | 70 | 49 | 48 | 41 | 12 |
| KMPMQ418_19_Rhiniinae | 466 | 70 | 70 | 49 | 48 | 41 | 12 |
| KMPMQ422_19_Rhiniinae | 289 | 70 | 70 | 49 | 48 | 41 | 12 |
| KMPMQ423_19_Rhiniinae | 223 | 70 | 70 | 49 | 48 | 41 | 12 |
| KMPMQ427_19_Rhiniinae | 467 | 70 | 70 | 49 | 48 | 41 | 12 |
| KMPMQ438_19_Rhiniinae | 468 | 70 | 70 | 49 | 48 | 41 | 12 |
| KMPMQ451_19_Rhiniinae | 469 | 70 | 70 | 49 | 48 | 41 | 12 |
| KMPMQ461_19_Rhiniinae | 223 | 70 | 70 | 49 | 48 | 41 | 12 |
| KMPMQ466_19_Rhiniinae | 278 | 107 | 107 | 51 | 50 | 43 | 42 |
| KMPMQ475_19_Rhiniinae | 272 | 107 | 107 | 51 | 50 | 43 | 42 |
| KMPMQ481_19_Rhiniinae | 470 | 107 | 107 | 51 | 50 | 43 | 42 |
| KMPMQ496_19_Rhiniinae | 471 | 107 | 107 | 51 | 50 | 43 | 42 |
| KMPMS056_19_Rhiniinae | 472 | 107 | 107 | 51 | 50 | 43 | 42 |
| KMPNA1081_18_Rhiniinae | 473 | 107 | 107 | 51 | 50 | 43 | 42 |
| KMPNA1090_18_Rhiniinae | 91 | 7 | 7 | 6 | 6 | 1 | 1 |
| KMPNA143_18_Rhiniinae | 223 | 70 | 70 | 49 | 48 | 41 | 12 |
| KMPNA278_18_Rhiniinae | 474 | 70 | 70 | 49 | 48 | 41 | 12 |
| KMPNA288_18_Rhiniinae | 475 | 70 | 70 | 49 | 48 | 41 | 12 |
| KMPNA345_18_Rhyncomya_soyauxi | 476 | 71 | 71 | 50 | 49 | 42 | 41 |
| KMPNB1132_18_Rhyncomya_soyauxi | 459 | 71 | 71 | 50 | 49 | 42 | 41 |
| KMPNB1134_18_Rhiniinae | 477 | 70 | 70 | 49 | 48 | 41 | 12 |
| KMPNB1200_18_Rhiniinae | 346 | 70 | 70 | 49 | 48 | 41 | 12 |
| KMPNB1275_18_Rhiniinae | 478 | 107 | 107 | 51 | 50 | 43 | 42 |
| KMPND457_18_Rhiniinae | 236 | 69 | 69 | 48 | 47 | 40 | 40 |
| KMPND461_18_Rhiniinae | 479 | 70 | 70 | 49 | 48 | 41 | 12 |
| KMPNG022_19_Rhiniinae | 145 | 72 | 72 | 49 | 48 | 41 | 12 |
| KMPNH1260_19_Rhiniinae | 223 | 70 | 70 | 49 | 48 | 41 | 12 |
| KMPNH1261_19_Rhiniinae | 226 | 70 | 70 | 49 | 48 | 41 | 12 |
| KMPNH1262_19_Rhiniinae | 223 | 70 | 70 | 49 | 48 | 41 | 12 |
| KMPNH1268_19_Rhiniinae | 223 | 70 | 70 | 49 | 48 | 41 | 12 |
| KMPNH1272_19_Rhiniinae | 237 | 70 | 70 | 49 | 48 | 41 | 12 |
| KMPNH1275_19_Rhiniinae | 480 | 107 | 107 | 51 | 50 | 43 | 42 |
| KMPNI1426_19_Rhiniinae | 223 | 70 | 70 | 49 | 48 | 41 | 12 |
| KMPNI1440_19_Rhiniinae | 295 | 110 | 110 | 76 | 75 | 66 | 63 |
| KMPNI325_19_Rhiniinae | 481 | 70 | 70 | 49 | 48 | 41 | 12 |
| KMPNI328_19_Rhiniinae | 482 | 70 | 70 | 49 | 48 | 41 | 12 |
| KMPNI331_19_Rhiniinae | 309 | 70 | 70 | 49 | 48 | 41 | 12 |
| KMPNJ009_19_Rhiniinae | 92 | 2 | 2 | 2 | 2 | 2 | 2 |
| KMPNJ036_19_Rhiniinae | 483 | 107 | 107 | 51 | 50 | 43 | 42 |
| KMPNJ042_19_Rhiniinae | 484 | 107 | 107 | 51 | 50 | 43 | 42 |
| KMPNJ046_19_Rhiniinae | 223 | 70 | 70 | 49 | 48 | 41 | 12 |
| KMPNJ049_19_Rhiniinae | 485 | 107 | 107 | 51 | 50 | 43 | 42 |
| KMPNJ059_19_Rhiniinae | 278 | 107 | 107 | 51 | 50 | 43 | 42 |
| KMPNJ1382_19_Rhiniinae | 223 | 70 | 70 | 49 | 48 | 41 | 12 |
| KMPNK046_19_Rhiniinae | 223 | 70 | 70 | 49 | 48 | 41 | 12 |
| KMPNK052_19_Rhiniinae | 214 | 70 | 70 | 49 | 48 | 41 | 12 |
| KMPNK053_19_Rhiniinae | 486 | 70 | 70 | 49 | 48 | 41 | 12 |
| KMPNK215_19_Rhiniinae | 487 | 107 | 107 | 51 | 50 | 43 | 42 |
| KMPNK245_19_Rhiniinae | 488 | 107 | 107 | 51 | 50 | 43 | 42 |
| KMPNK344_19_Rhiniinae | 489 | 118 | 118 | 51 | 50 | 43 | 42 |
| KMPNL715_19_Rhiniinae | 223 | 70 | 70 | 49 | 48 | 41 | 12 |
| KMPNL716_19_Rhiniinae | 329 | 70 | 70 | 49 | 48 | 41 | 12 |
| KMPNL724_19_Rhiniinae | 272 | 107 | 107 | 51 | 50 | 43 | 42 |
| KMPNL729_19_Rhiniinae | 214 | 70 | 70 | 49 | 48 | 41 | 12 |
| KMPNL731_19_Rhiniinae | 236 | 69 | 69 | 48 | 47 | 40 | 40 |
| KMPNL732_19_Rhiniinae | 214 | 70 | 70 | 49 | 48 | 41 | 12 |
| KMPNL734_19_Rhiniinae | 223 | 70 | 70 | 49 | 48 | 41 | 12 |
| KMPNL738_19_Rhiniinae | 340 | 70 | 70 | 49 | 48 | 41 | 12 |
| KMPNM007_19_Rhiniinae | 490 | 107 | 107 | 51 | 50 | 43 | 42 |
| KMPNM112_19_Rhiniinae | 491 | 107 | 107 | 51 | 50 | 43 | 42 |
| KMPNM1244_19_Rhiniinae | 214 | 70 | 70 | 49 | 48 | 41 | 12 |
| KMPNM1249_19_Rhiniinae | 214 | 70 | 70 | 49 | 48 | 41 | 12 |
| KMPNM1250_19_Rhiniinae | 223 | 70 | 70 | 49 | 48 | 41 | 12 |
| KMPNM1253_19_Rhiniinae | 471 | 107 | 107 | 51 | 50 | 43 | 42 |
| KMPNM1254_19_Rhiniinae | 223 | 70 | 70 | 49 | 48 | 41 | 12 |
| KMPNM1255_19_Rhiniinae | 214 | 70 | 70 | 49 | 48 | 41 | 12 |
| KMPNM1256_19_Rhiniinae | 226 | 70 | 70 | 49 | 48 | 41 | 12 |
| KMPNM1257_19_Rhiniinae | 236 | 69 | 69 | 48 | 47 | 40 | 40 |
| KMPNM1261_19_Rhiniinae | 492 | 70 | 70 | 49 | 48 | 41 | 12 |
| KMPNM170_19_Rhiniinae | 493 | 107 | 107 | 51 | 50 | 43 | 42 |
| KMPNM177_19_Rhiniinae | 312 | 107 | 107 | 51 | 50 | 43 | 42 |
| KMPNO1136_19_Rhiniinae | 240 | 107 | 107 | 51 | 50 | 43 | 42 |
| KMPNO1398_19_Rhiniinae | 289 | 70 | 70 | 49 | 48 | 41 | 12 |
| KMPNO257_19_Rhiniinae | 494 | 107 | 107 | 51 | 50 | 43 | 42 |
| KMPNO831_19_Rhiniinae | 495 | 107 | 107 | 51 | 50 | 43 | 42 |
| KMPNP1547_19_Rhyncomya_soyauxi | 221 | 71 | 71 | 50 | 49 | 42 | 41 |
| KMPNP1549_19_Rhiniinae | 236 | 69 | 69 | 48 | 47 | 40 | 40 |
| KMPNP1562_19_Rhiniinae | 223 | 70 | 70 | 49 | 48 | 41 | 12 |
| KMPNP1566_19_Rhiniinae | 223 | 70 | 70 | 49 | 48 | 41 | 12 |
| KMPNP1568_19_Rhiniinae | 496 | 70 | 70 | 49 | 48 | 41 | 12 |
| KMPNQ2515_19_Rhiniinae | 223 | 70 | 70 | 49 | 48 | 41 | 12 |
| KMPNQ2523_19_Rhiniinae | 223 | 70 | 70 | 49 | 48 | 41 | 12 |
| KMPNQ2547_19_Rhiniinae | 497 | 107 | 107 | 51 | 50 | 43 | 42 |
| KMPNQ2561_19_Rhiniinae | 223 | 70 | 70 | 49 | 48 | 41 | 12 |
| KMPNQ2565_19_Rhiniinae | 498 | 107 | 107 | 51 | 50 | 43 | 42 |
| KMPNQ2577_19_Rhiniinae | 499 | 107 | 107 | 51 | 50 | 43 | 42 |
| KMPNQ455_19_Rhiniinae | 427 | 107 | 107 | 51 | 50 | 43 | 42 |
| KMPNR091_19_Rhiniinae | 223 | 70 | 70 | 49 | 48 | 41 | 12 |
| KMPNR097_19_Rhiniinae | 223 | 70 | 70 | 49 | 48 | 41 | 12 |
| KMPNR103_19_Rhiniinae | 226 | 70 | 70 | 49 | 48 | 41 | 12 |
| KMPNR659_19_Rhiniinae | 500 | 107 | 107 | 51 | 50 | 43 | 42 |
| KMPNS1504_19_Rhiniinae | 289 | 70 | 70 | 49 | 48 | 41 | 12 |
| KMPNS1511_19_Rhiniinae | 135 | 72 | 72 | 49 | 48 | 41 | 12 |
| KMPOE009_18_Rhyncomya_soyauxi | 221 | 71 | 71 | 50 | 49 | 42 | 41 |
| KMPOG215_19_Rhiniinae | 431 | 107 | 107 | 51 | 50 | 43 | 42 |
| KMPOI052_19_Rhiniinae | 223 | 70 | 70 | 49 | 48 | 41 | 12 |
| KMPOK239_19_Rhiniinae | 145 | 72 | 72 | 49 | 48 | 41 | 12 |
| KMPOK240_19_Rhiniinae | 214 | 70 | 70 | 49 | 48 | 41 | 12 |
| KMPOL085_19_Rhiniinae | 501 | 107 | 107 | 51 | 50 | 43 | 42 |
| KMPOL089_19_Rhiniinae | 214 | 70 | 70 | 49 | 48 | 41 | 12 |
| KMPOM226_19_Rhiniinae | 226 | 70 | 70 | 49 | 48 | 41 | 12 |
| KMPOO174_19_Rhiniinae | 502 | 107 | 107 | 51 | 50 | 43 | 42 |
| KMPOP042_19_Rhiniinae | 400 | 107 | 107 | 51 | 50 | 43 | 42 |
| KMPOP045_19_Rhiniinae | 214 | 70 | 70 | 49 | 48 | 41 | 12 |
| KMPOR020_19_Rhiniinae | 223 | 70 | 70 | 49 | 48 | 41 | 12 |
| KMPOR021_19_Rhiniinae | 503 | 107 | 107 | 51 | 50 | 43 | 42 |
| KMPOS021_19_Rhiniinae | 504 | 70 | 70 | 49 | 48 | 41 | 12 |
| KMPOS024_19_Rhiniinae | 223 | 70 | 70 | 49 | 48 | 41 | 12 |
| KMPOS025_19_Rhiniinae | 482 | 70 | 70 | 49 | 48 | 41 | 12 |
| KMPOS030_19_Rhiniinae | 505 | 107 | 107 | 51 | 50 | 43 | 42 |
| KMPOS078_19_Rhiniinae | 431 | 107 | 107 | 51 | 50 | 43 | 42 |
| KMPOT063_19_Rhiniinae | 223 | 70 | 70 | 49 | 48 | 41 | 12 |
| KMPOT072_19_Rhiniinae | 339 | 70 | 70 | 49 | 48 | 41 | 12 |
| KMPOT218_19_Rhiniinae | 506 | 107 | 107 | 51 | 50 | 43 | 42 |
| KMPOX007_19_Rhiniinae | 507 | 70 | 70 | 49 | 48 | 41 | 12 |
| KMPOX008_19_Rhyncomya_soyauxi | 221 | 71 | 71 | 50 | 49 | 42 | 41 |
| KMPOX009_19_Rhiniinae | 237 | 70 | 70 | 49 | 48 | 41 | 12 |
| KMPOX015_19_Rhiniinae | 508 | 107 | 107 | 51 | 50 | 43 | 42 |
| KMPPI1145_19_Rhiniinae | 482 | 70 | 70 | 49 | 48 | 41 | 12 |
| KMPQ1073_19_Rhiniinae | 509 | 70 | 70 | 49 | 48 | 41 | 12 |
| KMPQJ089_19_Rhiniinae | 510 | 110 | 110 | 76 | 75 | 66 | 63 |
| KMPQT025_19_Rhiniinae | 511 | 119 | 119 | 80 | 79 | 67 | 64 |
| KMPRF129_19_Rhiniinae | 312 | 107 | 107 | 51 | 50 | 43 | 42 |
| KMPRH082_19_Rhiniinae | 512 | 107 | 107 | 51 | 50 | 43 | 42 |
| KMPRI160_19_Rhiniinae | 223 | 70 | 70 | 49 | 48 | 41 | 12 |
| KMPRK054_19_Rhyncomya_soyauxi | 513 | 71 | 71 | 50 | 49 | 42 | 41 |
| KMPRL034_19_Rhiniinae | 514 | 107 | 107 | 51 | 50 | 43 | 42 |
| KMPRL036_19_Rhiniinae | 515 | 70 | 70 | 49 | 48 | 41 | 12 |
| KMPRL038_19_Rhiniinae | 319 | 110 | 110 | 76 | 75 | 66 | 63 |
| KMPRM088_19_Rhiniinae | 435 | 107 | 107 | 51 | 50 | 43 | 42 |
| KMPRM325_19_Rhiniinae | 516 | 107 | 107 | 51 | 50 | 43 | 42 |
| KMPRO1095_19_Rhiniinae | 223 | 70 | 70 | 49 | 48 | 41 | 12 |
| KMPRO1099_19_Rhiniinae | 223 | 70 | 70 | 49 | 48 | 41 | 12 |
| KMPRO1100_19_Rhiniinae | 135 | 72 | 72 | 49 | 48 | 41 | 12 |
| KMPRO1101_19_Rhiniinae | 223 | 70 | 70 | 49 | 48 | 41 | 12 |
| KMPRO1102_19_Rhiniinae | 223 | 70 | 70 | 49 | 48 | 41 | 12 |
| KMPRO1114_19_Rhiniinae | 223 | 70 | 70 | 49 | 48 | 41 | 12 |
| KMPRO1126_19_Rhiniinae | 223 | 70 | 70 | 49 | 48 | 41 | 12 |
| KMPRO1130_19_Rhiniinae | 316 | 70 | 70 | 49 | 48 | 41 | 12 |
| KMPRO1131_19_Rhiniinae | 316 | 70 | 70 | 49 | 48 | 41 | 12 |
| KMPRO1140_19_Rhiniinae | 223 | 70 | 70 | 49 | 48 | 41 | 12 |
| KMPRO1142_19_Rhiniinae | 517 | 70 | 70 | 49 | 48 | 41 | 12 |
| KMPRO1169_19_Rhiniinae | 226 | 70 | 70 | 49 | 48 | 41 | 12 |
| KMPRO1178_19_Rhiniinae | 214 | 70 | 70 | 49 | 48 | 41 | 12 |
| KMPRO1191_19_Rhiniinae | 518 | 70 | 70 | 49 | 48 | 41 | 12 |
| KMPRO1192_19_Rhiniinae | 519 | 70 | 70 | 49 | 48 | 41 | 12 |
| KMPRO1200_19_Rhiniinae | 520 | 70 | 70 | 49 | 48 | 41 | 12 |
| KMPRO1208_19_Rhiniinae | 521 | 70 | 70 | 49 | 48 | 41 | 12 |
| KMPRO1209_19_Rhiniinae | 223 | 70 | 70 | 49 | 48 | 41 | 12 |
| KMPRO1210_19_Rhiniinae | 522 | 70 | 70 | 49 | 48 | 41 | 12 |
| KMPRT093_19_Rhiniinae | 523 | 107 | 107 | 51 | 50 | 43 | 42 |
| KMPRU005_19_Rhiniinae | 223 | 70 | 70 | 49 | 48 | 41 | 12 |
| KMPRV076_19_Rhiniinae | 237 | 70 | 70 | 49 | 48 | 41 | 12 |
| KMPRV083_19_Rhiniinae | 391 | 70 | 70 | 49 | 48 | 41 | 12 |
| KMPRV089_19_Rhiniinae | 223 | 70 | 70 | 49 | 48 | 41 | 12 |
| KMPRV464_19_Rhiniinae | 524 | 107 | 107 | 51 | 50 | 43 | 42 |
| KMPSD032_18_Rhiniinae | 525 | 107 | 107 | 51 | 50 | 43 | 42 |
| KMPSF185_19_Rhyncomya_soyauxi | 526 | 71 | 71 | 50 | 49 | 42 | 41 |
| KMPSF186_19_Rhiniinae | 319 | 110 | 110 | 76 | 75 | 66 | 63 |
| KMPSH308_19_Rhiniinae | 527 | 107 | 107 | 51 | 50 | 43 | 42 |
| KMPSI076_19_Rhiniinae | 278 | 107 | 107 | 51 | 50 | 43 | 42 |
| KMPSI193_19_Rhiniinae | 528 | 107 | 107 | 51 | 50 | 43 | 42 |
| KMPSO042_19_Rhiniinae | 529 | 70 | 70 | 49 | 48 | 41 | 12 |
| KMPSO515_19_Rhiniinae | 530 | 70 | 70 | 49 | 48 | 41 | 12 |
| KMPSO562_19_Rhiniinae | 400 | 107 | 107 | 51 | 50 | 43 | 42 |
| KMPSP2607_19_Rhiniinae | 531 | 70 | 70 | 49 | 48 | 41 | 12 |
| KMPSP2616_19_Rhyncomya_soyauxi | 532 | 71 | 71 | 50 | 49 | 42 | 41 |
| KMPSP2639_19_Rhiniinae | 431 | 107 | 107 | 51 | 50 | 43 | 42 |
| KMPSP762_19_Rhiniinae | 533 | 107 | 107 | 51 | 50 | 43 | 42 |
| KMPSP775_19_Rhiniinae | 534 | 107 | 107 | 51 | 50 | 43 | 42 |
| KMPST066_19_Rhiniinae | 535 | 107 | 107 | 51 | 50 | 43 | 42 |
| KMPSU033_19_Rhiniinae | 536 | 107 | 107 | 51 | 50 | 43 | 42 |
| KMPSU034_19_Rhiniinae | 537 | 107 | 107 | 51 | 50 | 43 | 42 |
| KMPSV847_19_Rhiniinae | 319 | 110 | 110 | 76 | 75 | 66 | 63 |
| KMPUA165_18_Rhiniinae | 538 | 108 | 108 | 75 | 74 | 65 | 12 |
| KMPUA168_18_Rhyncomya_soyauxi | 539 | 71 | 71 | 50 | 49 | 42 | 41 |
| KMPUB2732_18_Rhyncomya_soyauxi | 540 | 71 | 71 | 50 | 49 | 42 | 41 |
| KMPUB2740_18_Rhiniinae | 236 | 69 | 69 | 48 | 47 | 40 | 40 |
| KMPUC1609_18_Rhiniinae | 541 | 108 | 108 | 75 | 74 | 65 | 12 |
| KMPUC1622_18_Rhiniinae | 542 | 70 | 70 | 49 | 48 | 41 | 12 |
| KMPUC2832_18_Rhiniinae | 214 | 70 | 70 | 49 | 48 | 41 | 12 |
| KMPUC2839_18_Rhiniinae | 232 | 108 | 108 | 75 | 74 | 65 | 12 |
| KMPUD107_19_Rhyncomya_soyauxi | 543 | 71 | 71 | 50 | 49 | 42 | 41 |
| KMPUD123_19_Rhyncomya_soyauxi | 221 | 71 | 71 | 50 | 49 | 42 | 41 |
| KMPUD130_19_Rhyncomya_soyauxi | 544 | 71 | 71 | 50 | 49 | 42 | 41 |
| KMPUD144_19_Rhyncomya_soyauxi | 545 | 120 | 120 | 50 | 49 | 42 | 41 |
| KMPUD148_19_Rhyncomya_soyauxi | 546 | 71 | 71 | 50 | 49 | 42 | 41 |
| KMPUD1639_19_Rhiniinae | 547 | 107 | 107 | 51 | 50 | 43 | 42 |
| KMPUD168_19_Rhyncomya_soyauxi | 222 | 71 | 71 | 50 | 49 | 42 | 41 |
| KMPUD211_19_Rhyncomya_soyauxi | 548 | 71 | 71 | 50 | 49 | 42 | 41 |
| KMPUD2126_19_Rhiniinae | 324 | 107 | 107 | 51 | 50 | 43 | 42 |
| KMPUD232_19_Rhiniinae | 223 | 70 | 70 | 49 | 48 | 41 | 12 |
| KMPUD236_19_Rhiniinae | 549 | 107 | 107 | 51 | 50 | 43 | 42 |
| KMPUD240_19_Rhyncomya_soyauxi | 550 | 71 | 71 | 50 | 49 | 42 | 41 |
| KMPUD260_19_Rhiniinae | 551 | 70 | 70 | 49 | 48 | 41 | 12 |
| KMPUD261_19_Rhiniinae | 552 | 107 | 107 | 51 | 50 | 43 | 42 |
| KMPUD266_19_Rhyncomya_soyauxi | 225 | 71 | 71 | 50 | 49 | 42 | 41 |
| KMPUD2740_19_Rhiniinae | 553 | 107 | 107 | 51 | 50 | 43 | 42 |
| KMPUD281_19_Rhyncomya_soyauxi | 554 | 71 | 71 | 50 | 49 | 42 | 41 |
| KMPUD309_19_Rhyncomya_soyauxi | 221 | 71 | 71 | 50 | 49 | 42 | 41 |
| KMPUD329_19_Rhyncomya_soyauxi | 221 | 71 | 71 | 50 | 49 | 42 | 41 |
| KMPUD339_19_Rhyncomya_soyauxi | 555 | 71 | 71 | 50 | 49 | 42 | 41 |
| KMPUD349_19_Rhiniinae | 227 | 107 | 107 | 51 | 50 | 43 | 42 |
| KMPUD357_19_Rhyncomya_soyauxi | 556 | 71 | 71 | 50 | 49 | 42 | 41 |
| KMPUD4445_19_Rhiniinae | 232 | 108 | 108 | 75 | 74 | 65 | 12 |
| KMPUD4446_19_Rhiniinae | 278 | 107 | 107 | 51 | 50 | 43 | 42 |
| KMPUD4497_19_Rhiniinae | 557 | 121 | 121 | 51 | 50 | 43 | 42 |
| KMPUD4511_19_Rhiniinae | 232 | 108 | 108 | 75 | 74 | 65 | 12 |
| KMPUD4516_19_Rhiniinae | 558 | 107 | 107 | 51 | 50 | 43 | 42 |
| KMPUE617_19_Rhyncomya_soyauxi | 539 | 71 | 71 | 50 | 49 | 42 | 41 |
| KMPUE627_19_Rhiniinae | 223 | 70 | 70 | 49 | 48 | 41 | 12 |
| KMPUH069_19_Rhiniinae | 559 | 107 | 107 | 51 | 50 | 43 | 42 |
| KMPUH204_19_Rhiniinae | 560 | 70 | 70 | 49 | 48 | 41 | 12 |
| KMPUH207_19_Rhyncomya_soyauxi | 225 | 71 | 71 | 50 | 49 | 42 | 41 |
| KMPUH226_19_Rhiniinae | 561 | 109 | 109 | 21 | 20 | 15 | 15 |
| KMPUH228_19_Rhiniinae | 562 | 108 | 108 | 75 | 74 | 65 | 12 |
| KMPUH247_19_Rhiniinae | 232 | 108 | 108 | 75 | 74 | 65 | 12 |
| KMPUH274_19_Rhiniinae | 435 | 107 | 107 | 51 | 50 | 43 | 42 |
| KMPUH341_19_Rhiniinae | 563 | 107 | 107 | 51 | 50 | 43 | 42 |
| KMPUI083_19_Rhiniinae | 564 | 108 | 108 | 75 | 74 | 65 | 12 |
| KMPUI085_19_Rhiniinae | 564 | 108 | 108 | 75 | 74 | 65 | 12 |
| KMPUI119_19_Rhiniinae | 565 | 70 | 70 | 49 | 48 | 41 | 12 |
| KMPUI135_19_Rhiniinae | 232 | 108 | 108 | 75 | 74 | 65 | 12 |
| KMPUI163_19_Rhyncomya_soyauxi | 366 | 71 | 71 | 50 | 49 | 42 | 41 |
| KMPUI166_19_Rhiniinae | 232 | 108 | 108 | 75 | 74 | 65 | 12 |
| KMPUI178_19_Rhiniinae | 566 | 108 | 108 | 75 | 74 | 65 | 12 |
| KMPUI198_19_Rhiniinae | 567 | 107 | 107 | 51 | 50 | 43 | 42 |
| KMPUJ014_19_Rhiniinae | 226 | 70 | 70 | 49 | 48 | 41 | 12 |
| KMPUJ016_19_Rhiniinae | 223 | 70 | 70 | 49 | 48 | 41 | 12 |
| KMPUJ046_19_Rhiniinae | 258 | 107 | 107 | 51 | 50 | 43 | 42 |
| KMPUJ101_19_Rhyncomya_soyauxi | 459 | 71 | 71 | 50 | 49 | 42 | 41 |
| KMPUJ129_19_Rhiniinae | 309 | 70 | 70 | 49 | 48 | 41 | 12 |
| KMPUJ131_19_Rhyncomya_soyauxi | 270 | 71 | 71 | 50 | 49 | 42 | 41 |
| KMPUJ1366_19_Rhiniinae | 568 | 122 | 122 | 51 | 50 | 43 | 42 |
| KMPUJ1413_19_Rhiniinae | 569 | 107 | 107 | 51 | 50 | 43 | 42 |
| KMPUJ3505_19_Rhiniinae | 570 | 107 | 107 | 51 | 50 | 43 | 42 |
| KMPUJ477_19_Rhyncomya_soyauxi | 270 | 71 | 71 | 50 | 49 | 42 | 41 |
| KMPUJ478_19_Rhiniinae | 252 | 70 | 70 | 49 | 48 | 41 | 12 |
| KMPUJ479_19_Rhyncomya_soyauxi | 224 | 71 | 71 | 50 | 49 | 42 | 41 |
| KMPUJ486_19_Rhiniinae | 223 | 70 | 70 | 49 | 48 | 41 | 12 |
| KMPUJ490_19_Rhyncomya_soyauxi | 571 | 71 | 71 | 50 | 49 | 42 | 41 |
| KMPUJ557_19_Rhiniinae | 572 | 70 | 70 | 49 | 48 | 41 | 12 |
| KMPUJ578_19_Rhiniinae | 573 | 107 | 107 | 51 | 50 | 43 | 42 |
| KMPUJ612_19_Rhiniinae | 574 | 107 | 107 | 51 | 50 | 43 | 42 |
| KMPUJ618_19_Rhiniinae | 291 | 109 | 109 | 21 | 20 | 15 | 15 |
| KMPUJ6280_19_Rhiniinae | 575 | 107 | 107 | 51 | 50 | 43 | 42 |
| KMPUJ831_19_Rhiniinae | 272 | 107 | 107 | 51 | 50 | 43 | 42 |
| KMPVA007_18_Rhiniinae | 576 | 123 | 123 | 51 | 50 | 43 | 42 |
| KMPVA152_18_Rhiniinae | 237 | 70 | 70 | 49 | 48 | 41 | 12 |
| KMPVH036_19_Rhiniinae | 577 | 107 | 107 | 51 | 50 | 43 | 42 |
| KMPVJ250_19_Rhiniinae | 578 | 108 | 108 | 75 | 74 | 65 | 12 |
| KMPVV002_19_Rhiniinae | 579 | 70 | 70 | 49 | 48 | 41 | 12 |
| KMPWA512_18_Rhiniinae | 580 | 107 | 107 | 51 | 50 | 43 | 42 |
| KMPWB072_18_Rhyncomya_soyauxi | 581 | 71 | 71 | 50 | 49 | 42 | 41 |
| KMPWC021_18_Rhyncomya_soyauxi | 221 | 71 | 71 | 50 | 49 | 42 | 41 |
| KMPWG638_18_Rhiniinae | 272 | 107 | 107 | 51 | 50 | 43 | 42 |
| KMPWL304_18_Rhiniinae | 582 | 70 | 70 | 49 | 48 | 41 | 12 |
| KMPWL309_18_Rhiniinae | 240 | 107 | 107 | 51 | 50 | 43 | 42 |
| KMPWL312_18_Rhiniinae | 583 | 107 | 107 | 51 | 50 | 43 | 42 |
| KMPWM031_18_Rhiniinae | 301 | 107 | 107 | 51 | 50 | 43 | 42 |
| KMPWN152_19_Rhiniinae | 584 | 107 | 107 | 51 | 50 | 43 | 42 |
| KMPWN154_19_Rhiniinae | 585 | 70 | 70 | 49 | 48 | 41 | 12 |
| KMPWO022_19_Rhiniinae | 309 | 70 | 70 | 49 | 48 | 41 | 12 |
| KMPWO024_19_Rhiniinae | 586 | 124 | 124 | 2 | 2 | 2 | 2 |
| KMPWO026_19_Rhiniinae | 256 | 70 | 70 | 49 | 48 | 41 | 12 |
| KMPWO028_19_Rhiniinae | 587 | 70 | 70 | 49 | 48 | 41 | 12 |
| KMPWO030_19_Rhiniinae | 588 | 107 | 107 | 51 | 50 | 43 | 42 |
| KMPWO035_19_Rhiniinae | 589 | 107 | 107 | 51 | 50 | 43 | 42 |
| KMPWO1317_19_Rhiniinae | 590 | 107 | 107 | 51 | 50 | 43 | 42 |
| KMPWO1556_19_Rhiniinae | 591 | 107 | 107 | 51 | 50 | 43 | 42 |
| KMPWR110_19_Rhiniinae | 477 | 70 | 70 | 49 | 48 | 41 | 12 |
| KMPWR112_19_Rhiniinae | 289 | 70 | 70 | 49 | 48 | 41 | 12 |
| KMPWR197_19_Rhiniinae | 498 | 107 | 107 | 51 | 50 | 43 | 42 |
| KMPWS172_19_Rhiniinae | 592 | 107 | 107 | 51 | 50 | 43 | 42 |
| KMPWS186_19_Rhyncomya_soyauxi | 221 | 71 | 71 | 50 | 49 | 42 | 41 |
| KMPWT2205_19_Rhyncomya_soyauxi | 593 | 71 | 71 | 50 | 49 | 42 | 41 |
| KMPWT2208_19_Rhiniinae | 594 | 107 | 107 | 51 | 50 | 43 | 42 |
| KMPWT2210_19_Rhiniinae | 595 | 107 | 107 | 51 | 50 | 43 | 42 |
| KMPWT411_19_Rhiniinae | 596 | 107 | 107 | 51 | 50 | 43 | 42 |
| KMPWU088_19_Rhiniinae | 597 | 107 | 107 | 51 | 50 | 43 | 42 |
| KMPWU295_19_Rhiniinae | 598 | 107 | 107 | 51 | 50 | 43 | 42 |
| KMPXH076_19_Rhyncomya_soyauxi | 225 | 71 | 71 | 50 | 49 | 42 | 41 |
| KMPXN1418_19_Rhiniinae | 580 | 107 | 107 | 51 | 50 | 43 | 42 |
| KMPXN208_19_Rhiniinae | 258 | 107 | 107 | 51 | 50 | 43 | 42 |
| KMPXO359_19_Rhiniinae | 237 | 70 | 70 | 49 | 48 | 41 | 12 |
| KMPXO361_19_Rhiniinae | 214 | 70 | 70 | 49 | 48 | 41 | 12 |
| KMPXO372_19_Rhiniinae | 599 | 107 | 107 | 51 | 50 | 43 | 42 |
| KMPXO373_19_Rhiniinae | 281 | 107 | 107 | 51 | 50 | 43 | 42 |
| KMPXP1890_19_Rhiniinae | 327 | 107 | 107 | 51 | 50 | 43 | 42 |
| KMPXP1899_19_Rhyncomya_soyauxi | 600 | 71 | 71 | 50 | 49 | 42 | 41 |
| KMPXQ040_19_Rhyncomya_soyauxi | 540 | 71 | 71 | 50 | 49 | 42 | 41 |
| KMPXQ043_19_Rhiniinae | 601 | 107 | 107 | 51 | 50 | 43 | 42 |
| KMPXR029_19_Rhiniinae | 602 | 107 | 107 | 51 | 50 | 43 | 42 |
| KMPXR061_19_Rhiniinae | 431 | 107 | 107 | 51 | 50 | 43 | 42 |
| KMPXS074_19_Rhyncomya_soyauxi | 603 | 71 | 71 | 50 | 49 | 42 | 41 |
| KMPXS075_19_Rhiniinae | 240 | 107 | 107 | 51 | 50 | 43 | 42 |
| KMPYI012_19_Rhiniinae | 604 | 107 | 107 | 51 | 50 | 43 | 42 |
| KMPZB1214_19_Rhiniinae | 449 | 69 | 69 | 48 | 47 | 40 | 40 |
| KMPZB1217_19_Rhiniinae | 605 | 69 | 69 | 48 | 47 | 40 | 40 |
| KMPZB1226_19_Rhiniinae | 431 | 107 | 107 | 51 | 50 | 43 | 42 |
| KMPZB1230_19_Rhiniinae | 606 | 119 | 119 | 80 | 79 | 67 | 64 |
| KMPZB1232_19_Rhiniinae | 236 | 69 | 69 | 48 | 47 | 40 | 40 |
| KMPZB1234_19_Rhiniinae | 236 | 69 | 69 | 48 | 47 | 40 | 40 |
| KMPZB1236_19_Rhiniinae | 236 | 69 | 69 | 48 | 47 | 40 | 40 |
| KMPZB1261_19_Rhiniinae | 236 | 69 | 69 | 48 | 47 | 40 | 40 |
| KMPZB1264_19_Rhiniinae | 607 | 69 | 69 | 48 | 47 | 40 | 40 |
| KMPZB1270_19_Rhiniinae | 236 | 69 | 69 | 48 | 47 | 40 | 40 |
| KMPZB1283_19_Rhiniinae | 236 | 69 | 69 | 48 | 47 | 40 | 40 |
| KMPZB214_19_Rhiniinae | 511 | 119 | 119 | 80 | 79 | 67 | 64 |
| KMPZC050_19_Rhiniinae | 223 | 70 | 70 | 49 | 48 | 41 | 12 |
| KMPZC052_19_Rhiniinae | 214 | 70 | 70 | 49 | 48 | 41 | 12 |
| KMPZC060_19_Rhiniinae | 488 | 107 | 107 | 51 | 50 | 43 | 42 |
| KMPZE543_19_Rhiniinae | 608 | 107 | 107 | 51 | 50 | 43 | 42 |
| KMPZE549_19_Rhiniinae | 609 | 107 | 107 | 51 | 50 | 43 | 42 |
| KMPZE551_19_Rhiniinae | 431 | 107 | 107 | 51 | 50 | 43 | 42 |
| KMPZE553_19_Rhiniinae | 610 | 107 | 107 | 51 | 50 | 43 | 42 |
| KMTTB423_18_Rhiniinae | 223 | 70 | 70 | 49 | 48 | 41 | 12 |
| KMTTC030_18_Rhiniinae | 490 | 107 | 107 | 51 | 50 | 43 | 42 |
| KMTTF027_19_Rhiniinae | 214 | 70 | 70 | 49 | 48 | 41 | 12 |
| KMTTG130_19_Rhiniinae | 223 | 70 | 70 | 49 | 48 | 41 | 12 |
| KMTTH809_19_Rhiniinae | 223 | 70 | 70 | 49 | 48 | 41 | 12 |
| KMTTH817_19_Rhiniinae | 223 | 70 | 70 | 49 | 48 | 41 | 12 |
| KMTTH825_19_Rhiniinae | 405 | 110 | 110 | 76 | 75 | 66 | 63 |
| KMTTI015_19_Rhiniinae | 214 | 70 | 70 | 49 | 48 | 41 | 12 |
| KMTTJ070_19_Rhinia | 10 | 4 | 4 | 4 | 4 | 2 | 2 |
| KMTTL904_19_Rhiniinae | 252 | 70 | 70 | 49 | 48 | 41 | 12 |
| KMTTL906_19_Rhiniinae | 449 | 69 | 69 | 48 | 47 | 40 | 40 |
| KP004766_1_9_658_Stomorhina_lunata | 25 | 14 | 14 | 10 | 10 | 6 | 6 |
| KVIS016_21_Rhiniinae | 611 | 125 | 125 | 81 | 80 | 58 | 56 |
| KVIS021_21_Rhiniinae | 612 | 126 | 126 | 82 | 81 | 70 | 67 |
| KX054612_1_1_650_Rhiniidae_sp | 613 | 127 | 127 | 25 | 24 | 19 | 19 |
| KX054613_1_1_650_Rhiniidae_sp | 13 | 2 | 2 | 2 | 2 | 2 | 2 |
| KX054615_1_1_615_Rhiniidae_sp | 614 | 31 | 31 | 25 | 24 | 19 | 19 |
| KY031766_1_1_650_Isomyia_electa | 615 | 128 | 128 | 83 | 82 | 63 | 61 |
| KY031767_1_1_650_Isomyia_electa | 616 | 128 | 128 | 83 | 82 | 63 | 61 |
| KY031768_1_1_650_Isomyia_electa | 617 | 128 | 128 | 83 | 82 | 63 | 61 |
| KY031769_1_1_650_Isomyia_pseudolucilia | 618 | 129 | 129 | 84 | 83 | 71 | 68 |
| KY031771_1_1_650_Isomyia_pseudonepalana | 619 | 130 | 130 | 85 | 84 | 72 | 69 |
| KY031773_1_1_650_Isomyia_complantenna | 620 | 131 | 131 | 86 | 85 | 73 | 70 |
| KY031774_1_1_650_Isomyia_verirecta | 621 | 132 | 132 | 87 | 86 | 74 | 71 |
| KY031819_1_1_650_Stomorhina_discolor | 622 | 45 | 45 | 25 | 24 | 19 | 19 |
| KY031820_1_1_650_Stomorhina_discolor | 111 | 31 | 31 | 25 | 24 | 19 | 19 |
| KY031821_1_1_650_Stomorhina_obsoleta | 623 | 65 | 65 | 25 | 24 | 19 | 19 |
| KY749786_1_42_635_Stomorhina_lunata | 25 | 14 | 14 | 10 | 10 | 6 | 6 |
| KY835753_1_4_588_Rhiniidae_sp | 112 | 62 | 62 | 43 | 42 | 35 | 35 |
| KY837821_1_15_579_Rhiniidae_sp | 112 | 62 | 62 | 43 | 42 | 35 | 35 |
| KY838746_1_1_573_Rhiniidae_sp | 112 | 62 | 62 | 43 | 42 | 35 | 35 |
| KY838995_1_4_593_Rhiniidae_sp | 112 | 62 | 62 | 43 | 42 | 35 | 35 |
| KY841564_1_1_645_Rhiniidae_sp | 112 | 62 | 62 | 43 | 42 | 35 | 35 |
| KY842007_1_1_588_Stomorhina_discolor | 111 | 31 | 31 | 25 | 24 | 19 | 19 |
| KY844929_1_1_546_Stomorhina_discolor | 111 | 31 | 31 | 25 | 24 | 19 | 19 |
| LC477291_1_1_650_Stomorhina_obsoleta | 624 | 65 | 65 | 25 | 24 | 19 | 19 |
| LC549079_1_1_650_Borbororhinia_bivittata | 625 | 80 | 80 | 55 | 54 | 47 | 46 |
| LC549094_1_1_650_Borbororhinia_bivittata | 626 | 80 | 80 | 55 | 54 | 47 | 46 |
| LC682331_1_1_650_Stomorhina_obsoleta | 624 | 65 | 65 | 25 | 24 | 19 | 19 |
| MADIP048_10_Rhiniinae | 627 | 133 | 133 | 88 | 87 | 48 | 47 |
| MADIP049_10_Rhiniinae | 628 | 133 | 133 | 88 | 87 | 48 | 47 |
| MADIP050_10_Rhiniinae | 628 | 133 | 133 | 88 | 87 | 48 | 47 |
| MADIP051_10_Rhiniinae | 629 | 133 | 133 | 88 | 87 | 48 | 47 |
| MADIP113_10_Rhiniinae | 629 | 133 | 133 | 88 | 87 | 48 | 47 |
| MADIP1269_13_Rhiniinae | 630 | 134 | 134 | 57 | 56 | 48 | 47 |
| MADIP567_12_Rhiniinae | 165 | 82 | 82 | 57 | 56 | 48 | 47 |
| MADIP655_12_Rhiniinae | 631 | 134 | 134 | 57 | 56 | 48 | 47 |
| MBPT074_15_Rhiniinae | 155 | 78 | 78 | 53 | 52 | 45 | 44 |
| MF804688_1_6_650_Isomyia_sp | 632 | 135 | 135 | 89 | 88 | 75 | 36 |
| MG967831_1_1_650_Alikangiella_rufithorax | 633 | 136 | 136 | 90 | 89 | 76 | 72 |
| MG967868_1_1_650_Strongyloneura_prolata | 634 | 137 | 137 | 91 | 90 | 77 | 73 |
| MG968089_1_5_650_Thoracites_abdominalis | 635 | 138 | 138 | 92 | 91 | 78 | 74 |
| MG968160_1_1_650_Stegosoma_vinculatum | 636 | 10 | 10 | 8 | 8 | 5 | 5 |
| MN411061_1_1_650_Borbororhinia_bivittata | 637 | 139 | 139 | 93 | 92 | 79 | 75 |
| MN868726_1_1_650_Rhyncomya_felina | 638 | 140 | 140 | 94 | 93 | 80 | 76 |
| MN868811_1_1_650_Stomorhina_lunata | 25 | 14 | 14 | 10 | 10 | 6 | 6 |
| MN868846_1_1_650_Rhyncomya_columbina | 639 | 141 | 141 | 95 | 94 | 81 | 77 |
| NSWHO3428_18_Stomorhina_discolor | 81 | 45 | 45 | 25 | 24 | 19 | 19 |
| NSWHO3438_18_Stomorhina_discolor | 55 | 31 | 31 | 25 | 24 | 19 | 19 |
| OL343410_1_1_650_Stomorhina_obsoleta | 640 | 65 | 65 | 25 | 24 | 19 | 19 |
| OL343411_1_1_650_Stomorhina_obsoleta | 641 | 65 | 65 | 25 | 24 | 19 | 19 |
| OL343412_1_1_650_Stomorhina_obsoleta | 642 | 65 | 65 | 25 | 24 | 19 | 19 |
| OP268186_1_1_646_Stomorhina_discolor | 643 | 142 | 142 | 25 | 24 | 19 | 19 |
| OR497843_1_1560_2209_Isomyia_nebulosa | 644 | 143 | 143 | 96 | 95 | 82 | 78 |
| PLABN218_19_Rhiniinae | 645 | 144 | 144 | 97 | 96 | 83 | 79 |
| PLLBG1418_20_Rhiniinae | 646 | 144 | 144 | 97 | 96 | 83 | 79 |
| PLRCH080_20_Rhiniinae | 647 | 145 | 145 | 97 | 96 | 83 | 79 |
| R12_Rhinia_apicalis_F_Namibia | 3 | 2 | 2 | 2 | 2 | 2 | 2 |
| R18_Rhinia_apicalis_M_Togo | 3 | 2 | 2 | 2 | 2 | 2 | 2 |
| R2_Rhinia_sp2_F_Burundi | 648 | 146 | 146 | 98 | 97 | 84 | 2 |
| R24_Rhinia_apicalis_F_Namibia | 13 | 2 | 2 | 2 | 2 | 2 | 2 |
| R3_Rhinia_coxendix_M_DRCongo | 649 | 147 | 147 | 4 | 4 | 2 | 2 |
| S13_Stomorhina_rugosa_M_Malawi | 91 | 7 | 7 | 6 | 6 | 1 | 1 |
| S14_Stomorhina_rugosa_F_Ethiopia | 650 | 7 | 7 | 6 | 6 | 1 | 1 |
| S16_Stomorhina_guttata_F_SouthAfrica | 651 | 21 | 21 | 16 | 15 | 11 | 11 |
| S17_Stomorhina_guttata_M_SouthAfrica | 38 | 21 | 21 | 16 | 15 | 11 | 11 |
| S19_Stomorhina_apta_F_Burundi | 652 | 148 | 148 | 99 | 98 | 85 | 80 |
| S2_Stomorhina_chapini_F_DRCongo | 653 | 3 | 3 | 3 | 3 | 3 | 3 |
| S20_Stomorhina_apta_F_Burundi | 654 | 148 | 148 | 99 | 98 | 85 | 80 |
| S21_Stomorhina_lunata_F_Mauritius | 25 | 14 | 14 | 10 | 10 | 6 | 6 |
| S22_Stomorhina_lunata_M_Mauritius | 25 | 14 | 14 | 10 | 10 | 6 | 6 |
| S23_Stomorhina_lunata_M_Malawi | 25 | 14 | 14 | 10 | 10 | 6 | 6 |
| S24_Stomorhina_lunata_F_Malawi | 25 | 14 | 14 | 10 | 10 | 6 | 6 |
| S26_Stomorhina_lunata_M_SouthAfrica | 25 | 14 | 14 | 10 | 10 | 6 | 6 |
| S28_Stomorhina_cf_malobana_F_Malawi | 655 | 149 | 149 | 99 | 98 | 85 | 80 |
| S3_Stomorhina_chapini_F_SouthAfrica | 656 | 150 | 150 | 100 | 99 | 3 | 3 |
| S30_Stomorhina_sp2_cf_malobana_F_Malawi | 655 | 149 | 149 | 99 | 98 | 85 | 80 |
| S31_Stomorhina_malobana_M_Malawi | 655 | 149 | 149 | 99 | 98 | 85 | 80 |
| S4_Stomorhina_chapini_M_SouthAfrica | 657 | 151 | 151 | 100 | 99 | 3 | 3 |
| S40_Stomorhina_sp2_cf_malobana_F_Tanzania | 658 | 149 | 149 | 99 | 98 | 85 | 80 |
| S42_Stomorhina_sp2_cf_malobana_F_Tanzania | 658 | 149 | 149 | 99 | 98 | 85 | 80 |
| S45_Stomorhina_cribrata_M_Tanzania | 1 | 1 | 1 | 1 | 1 | 1 | 1 |
| S5_Stomorhina_sp1_cf_armatipes_F_SouthAfrica | 659 | 14 | 14 | 10 | 10 | 6 | 6 |
| S7_Stomorhina_lunata_F_SouthAfrica | 25 | 14 | 14 | 10 | 10 | 6 | 6 |
| S8_Stomorhina_sp1_cf_armatipes_F_SouthAfrica | 659 | 14 | 14 | 10 | 10 | 6 | 6 |
| S9_Stomorhina_armatipes_M_SouthAfrica | 660 | 14 | 14 | 10 | 10 | 6 | 6 |
| SAFRA1138_18_Rhiniinae | 661 | 152 | 152 | 75 | 74 | 65 | 12 |
| SAFRA2206_18_Rhiniinae | 662 | 153 | 153 | 11 | 11 | 7 | 7 |
| SAFRA2207_18_Rhiniinae | 662 | 153 | 153 | 11 | 11 | 7 | 7 |
| SAFRA2211_18_Rhiniinae | 662 | 153 | 153 | 11 | 11 | 7 | 7 |
| SAFRA2212_18_Rhiniinae | 662 | 153 | 153 | 11 | 11 | 7 | 7 |
| SAFRA2214_18_Rhiniinae | 662 | 153 | 153 | 11 | 11 | 7 | 7 |
| SAFRA2215_18_Rhiniinae | 662 | 153 | 153 | 11 | 11 | 7 | 7 |
| SAFRA2220_18_Rhiniinae | 662 | 153 | 153 | 11 | 11 | 7 | 7 |
| SAFRA2230_18_Rhiniinae | 662 | 153 | 153 | 11 | 11 | 7 | 7 |
| SAFRA2243_18_Rhiniinae | 662 | 153 | 153 | 11 | 11 | 7 | 7 |
| SAFRA2256_18_Rhiniinae | 662 | 153 | 153 | 11 | 11 | 7 | 7 |
| SAFRA3346_18_Rhiniinae | 662 | 153 | 153 | 11 | 11 | 7 | 7 |
| SAFRA3350_18_Rhiniinae | 223 | 70 | 70 | 49 | 48 | 41 | 12 |
| T1_Thoracites_sp1_M_SouthAfrica | 663 | 154 | 154 | 74 | 73 | 64 | 62 |
| T3_Thoracites_sp1_F_SouthAfrica | 664 | 155 | 155 | 74 | 73 | 64 | 62 |
| T6_Thoracites_petersiana_F_SouthAfrica | 665 | 156 | 156 | 101 | 100 | 86 | 81 |
| T7_Thoracites_petersiana_M_SouthAfrica | 665 | 156 | 156 | 101 | 100 | 86 | 81 |
| UKMBB045_13_Stomorhina_discolor | 111 | 31 | 31 | 25 | 24 | 19 | 19 |
| USA01_Cosmina_fuscipennis_F_SouthAfrica | 666 | 42 | 42 | 32 | 31 | 25 | 25 |
| USA02_Cosmina_fuscipennis_M_SouthAfrica | 666 | 42 | 42 | 32 | 31 | 25 | 25 |
| USA03_Fainia_albitarsis_M_Kenya | 94 | 55 | 55 | 38 | 37 | 31 | 31 |
| USA04_Fainia_albitarsis_F_Kenya | 667 | 55 | 55 | 38 | 37 | 31 | 31 |
| USA05_Isomyia_nataliensis_F_SouthAfrica | 39 | 20 | 20 | 15 | 14 | 10 | 10 |
| USA06_Isomyia_nataliensis_M_SouthAfrica | 39 | 20 | 20 | 15 | 14 | 10 | 10 |
| USA07_Isomyia_pubera_F_SouthAfrica | 33 | 17 | 17 | 13 | 12 | 8 | 8 |
| USA08_Isomyia_natalensis_F_SouthAfrica | 39 | 20 | 20 | 15 | 14 | 10 | 10 |
| USA09_Isomyia_tristis_M_SouthAfrica | 34 | 18 | 18 | 11 | 11 | 7 | 7 |
| USA10_Isomyia_tristis_F_SouthAfrica | 26 | 15 | 15 | 11 | 11 | 7 | 7 |
| USA11_Rhiniaa_apicalis_F_Kenya | 668 | 2 | 2 | 2 | 2 | 2 | 2 |
| USA12_Rhyncomya_minutalis_F_SouthAfrica | 669 | 157 | 157 | 102 | 101 | 87 | 82 |
| USA13_Rhyncomya_soyauxi_F_Kenya | 670 | 71 | 71 | 50 | 49 | 42 | 41 |
| USA14_Rhyncomya_soyauxi_M_Kenya | 134 | 71 | 71 | 50 | 49 | 42 | 41 |
| USA15_Rhyncomya_soyauxi_F_Kenya | 134 | 71 | 71 | 50 | 49 | 42 | 41 |
| USA16_Rhyncomya_soyauxi_M_Kenya | 671 | 158 | 158 | 50 | 49 | 42 | 41 |
| USA18_Stomorhina_lunata_F_SouthAfrica | 25 | 14 | 14 | 10 | 10 | 6 | 6 |
| USA19_Stomorhina_lunata_M_SouthAfrica | 25 | 14 | 14 | 10 | 10 | 6 | 6 |
| Y10_Zumba_antennalis_F_SouthAfrica | 672 | 159 | 159 | 103 | 102 | 88 | 83 |
| Y13_Rhyncomya_sp16_cf_minutalis_M_SouthAfrica | 673 | 160 | 160 | 102 | 101 | 87 | 82 |
| Y15_Rhyncomya_forcipata_F_SouthAfrica | 214 | 70 | 70 | 49 | 48 | 41 | 12 |
| Y20_Rhyncomya_maculata_M_SouthAfrica | 674 | 161 | 161 | 102 | 101 | 87 | 82 |
| Y23_Rhyncomya_pruinosa_M_SouthAfrica | 236 | 69 | 69 | 48 | 47 | 40 | 40 |
| Y24_Rhyncomya_pruinosa_M_Malawi | 236 | 69 | 69 | 48 | 47 | 40 | 40 |
| Y25_Rhyncomya_pruinosa_F_Kenya | 148 | 69 | 69 | 48 | 47 | 40 | 40 |
| Y26_Rhyncomya_pruinosa_F_SouthAfrica | 236 | 69 | 69 | 48 | 47 | 40 | 40 |
| Y27_Rhyncomya_forcipata_F_SouthAfrica | 675 | 70 | 70 | 49 | 48 | 41 | 12 |
| Y28_Rhyncomya_forcipata_F_SouthAfrica | 223 | 70 | 70 | 49 | 48 | 41 | 12 |
| Y29_Rhyncomya_forcipata_M_Namibia | 223 | 70 | 70 | 49 | 48 | 41 | 12 |
| Y32_Rhyncomya_minutalis_F_SouthAfrica | 676 | 162 | 162 | 102 | 101 | 87 | 82 |
| Y35_Rhyncomya_cassotis_M_Malawi | 677 | 163 | 163 | 75 | 74 | 65 | 12 |
| Y36_Rhyncomya_sp11_cf_cassotis_M_Togo | 678 | 164 | 164 | 75 | 74 | 65 | 12 |
| Y37_Rhyncomya_sp12_cf_cassotis_M_Zambia | 679 | 152 | 152 | 75 | 74 | 65 | 12 |
| Y38_Rhyncomya_cassotis_F_Malawi | 680 | 163 | 163 | 75 | 74 | 65 | 12 |
| Y39_Rhyncomya_cassotis_F_Namibia | 681 | 152 | 152 | 75 | 74 | 65 | 12 |
| Y40_Rhyncomya_sp3_F_SouthAfrica | 682 | 165 | 165 | 104 | 103 | 89 | 84 |
| Y41_Rhyncomya_sp1_F_Cameroon | 683 | 164 | 164 | 75 | 74 | 65 | 12 |
| Y44_Rhyncomya_sp8_F_Namibia | 684 | 166 | 166 | 105 | 104 | 90 | 85 |
| Y46_Rhyncomya_paratristis_F_SouthAfrica | 685 | 167 | 167 | 106 | 105 | 91 | 86 |
| Y47_Rhyncomya_dasyops_F_SouthAfrica | 686 | 168 | 168 | 107 | 106 | 92 | 87 |
| Y56_Rhyncomya_sp19_F_Cameroon | 687 | 169 | 169 | 108 | 107 | 65 | 12 |
| Y57_Rhyncomya_sp3_F_SouthAfrica | 688 | 165 | 165 | 104 | 103 | 89 | 84 |
| Y58_Rhyncomya_trispina_M_Namibia | 689 | 107 | 107 | 51 | 50 | 43 | 42 |
| Y59_Rhyncomya_trispina_F_SouthAfrica | 690 | 107 | 107 | 51 | 50 | 43 | 42 |
| Y6_Rhyncomya_sp15_F_SouthAfrica | 691 | 170 | 170 | 109 | 108 | 93 | 88 |
| Y64_Rhyncomya_sp6_M_Togo | 692 | 171 | 171 | 110 | 109 | 94 | 89 |
| Y65_Rhyncomya_sp6_F_Togo | 692 | 171 | 171 | 110 | 109 | 94 | 89 |
| Y67_Rhyncomya_sp7_M_SouthAfrica | 693 | 172 | 172 | 102 | 101 | 87 | 82 |
| Y69_Rhyncomya_interclusa_M_SouthAfrica | 47 | 27 | 27 | 22 | 21 | 16 | 16 |
| Z2_Zumba_antennalis_F_SouthAfrica | 694 | 159 | 159 | 103 | 102 | 88 | 83 |
